# An Expanded Combined Evidence Approach to the *Gavialis* Problem Using Geometric Morphometric Data from Crocodylian Braincases and Eustachian Systems

**DOI:** 10.1371/journal.pone.0105793

**Published:** 2014-09-08

**Authors:** Maria Eugenia Leone Gold, Christopher A. Brochu, Mark A. Norell

**Affiliations:** 1 Richard Gilder Graduate School, American Museum of Natural History, New York, New York, United States of America; 2 Division of Paleontology, American Museum of Natural History, New York, New York, United States of America; 3 Department of Earth and Environmental Sciences, University of Iowa, Iowa City, Iowa, United States of America; BiK-F Biodiversity and Climate Research Center, Germany

## Abstract

The phylogenetic position of the Indian gharial (*Gavialis gangeticus*) is disputed - morphological characters place *Gavialis* as the sister to all other extant crocodylians, whereas molecular and combined analyses find *Gavialis* and the false gharial (*Tomistoma schlegelii*) to be sister taxa. Geometric morphometric techniques have only begun to be applied to this issue, but most of these studies have focused on the exterior of the skull. The braincase has provided useful phylogenetic information for basal crurotarsans, but has not been explored for the crown group. The Eustachian system is thought to vary phylogenetically in Crocodylia, but has not been analytically tested. To determine if gross morphology of the crocodylian braincase proves informative to the relationships of *Gavialis* and *Tomistoma*, we used two- and three-dimensional geometric morphometric approaches. Internal braincase images were obtained using high-resolution computerized tomography scans. A principal components analysis identified that the first component axis was primarily associated with size and did not show groupings that divide the specimens by phylogenetic affinity. Sliding semi-landmarks and a relative warp analysis indicate that a unique Eustachian morphology separates *Gavialis* from other extant members of Crocodylia. Ontogenetic expansion of the braincase results in a more dorsoventrally elongate median Eustachian canal. Changes in the shape of the Eustachian system do provide phylogenetic distinctions between major crocodylian clades. Each morphometric dataset, consisting of continuous morphological characters, was added independently to a combined cladistic analysis of discrete morphological and molecular characters. The braincase data alone produced a clade that included crocodylids and *Gavialis*, whereas the Eustachian data resulted in *Gavialis* being considered a basally divergent lineage. When each morphometric dataset was used in a combined analysis with discrete morphological and molecular characters, it generated a tree that matched the topology of the molecular phylogeny of Crocodylia.

## Introduction

Modern crocodylians are one of two extant groups of archosaurs [1], [2]. They first appear in the fossil record in the Late Cretaceous, between 80 and 90 million years ago [3]–[5]. Because of their excellent fossil record [6], the evolutionary relationships within and among many crocodylian clades are well understood. However, some areas of the tree remain controversial, particularly near the base of the crocodylian evolutionary tree. Among these is the phylogenetic position of the Indian gharial, *Gavialis gangeticus*, whose phylogenetic placement changes dramatically depending on which types of data are analyzed. Most morphological analyses place *Gavialis* as the sister taxon to all other extant members of Crocodylia with strong support (e.g. [3], [5], [7]–[14]). Conversely, phylogenetic analyses of molecular (i.e. mitochondrial and nuclear DNA) and most analyses using a combination of molecular and discrete morphological data find *Gavialis* to be most closely related to the Malayan false gharial, *Tomistoma schlegelii* (e.g. [1], [4], [15]–[21]).

A potentially confounding issue for morphological phylogenies is the hypothesized relative ecoplasticity of the crocodylian skull [22]–[24]. Even though bite force is similar in crocodylians of similar body sizes [25], differences in diet and other environmental factors [26], [27] may influence skull shape over evolutionary time. Convergence in skull shape due to these factors may cause disparate crocodylian lineages to look superficially similar [28] obfuscating true phylogenetic relationships.

Another potentially misleading factor is the substantial morphological variation that occurs throughout ontogeny [11]. Ontogenetic change in the crocodylian chondrocranium, especially with respect to the basisphenoid and basioccipital, results in a more dorsoventrally elongate braincase and Eustachian system [29]. Such ‘verticalization’ is accompanied by a reorientation of the quadrate and the jaw musculature, in addition to shifting of pneumatic passages within the braincase [30]. *Tomistoma* shares a verticalization pattern with crocodyloids and alligatoroids, but some have argued that *Gavialis* does not and has therefore been said to reflect a more plesiomorphic condition [29], [30]. The putatively plesiomorphic braincase configuration in *Gavialis* is consistent with a basal position for this species within Crocodylia.

Geometric morphometric analyses have provided an alternative for examining patterns of crocodylian diversification. This technique commonly employs homologous anatomical landmarks on specimens, digital models, or images of specimens [31] in two- or three-dimensions (2D and 3D, respectively). To visualize and analyze the underlying pattern of morphological variation, these data are subjected to a variety of multivariate techniques, including Principal Components Analysis (PCA) and other dimensionality-reducing methods [32], [33].

Multivariate statistics on landmark data have been used to model ontogenetic shape changes in four extant crocodylian species (*T. schlegelii, G. gangeticus, Mecistops cataphractus*, and *Crocodylus acutus*). The trajectory of *Gavialis* was found to differ both in rate and position in morphospace from the others [11]. These results are consistent with an outgroup position for *Gavialis* and lead the authors to support the classical phylogenetic hypothesis based on discrete morphology alone [11]. The landmarks used therein cover nearly the entire exposed surface of the skull [11]. However, many of these landmarks, especially those of the snout and palate, are from structures where size and shape may be influenced by environmental factors related to ontogenetic niche shifts (e.g. due to changes in diet) [27]. The internal anatomy of the braincase, which has yielded phylogenetically informative characters for basal crurotarsans [34], [35], has been ignored for morphometric analyses. Braincase landmarks could thus reveal phylogenetic relationships that have been obscured by ecological and functional factors in other locations of the skull.

Preliminary analyses using 2D geometric morphometric data on the braincase and Eustachian system suggested underlying similarity between *Tomistoma* and *Gavialis*, though these analyses were performed with few specimens [36]. To address the nature of phylogenetic signal within the size and shape of the braincase, we increased taxonomic and specimen sampling, and employ both 2D and 3D geometric morphometrics on the braincase and Eustachian system to more thoroughly investigate the change in shape of the crocodylian braincase, with the goal of elucidating on the phylogenetic affinities of *Gavialis* and *Tomistoma*. Both 2D and 3D landmarks were collected in order to examine congruence between the two data sets.

To address the phylogenetic position of *Gavialis* morphometric data from the braincase and Eustachian systems analyses were included in a combined analysis of discrete morphological and molecular characters for the clade. Although methods for implementing geometric morphometric data in cladistic analysis have been proposed [37]–[39], there have not been many attempts to conduct phylogenetic analyses on geometric morphometric data. Some argue that using morphometric data as phylogenetic characters is simply phenetics [40]. However, when situations arise where the two most used types of data (discrete morphology and molecules) result in disparate topologies (e.g. in Crocodylia), including geometric morphometric data into a combined analysis could add additional evidence for one topology over another in an already rooted system. Herein, internal cranial geometric morphometric data is analyzed and incorporated into a cladistic analysis for the first time. This analysis is the first of its kind for Crocodylia and could help resolve the problematic position of *Gavialis*.

## Materials and Methods

### Taxonomic Sampling and Specimen Imaging

Forty-eight specimens of 15 crocodylian species were subjected to high-resolution X-Ray Computer Tomography (CT) scanning using a GE phoenix v|tome|x CT scanner (GE Inspection Technologies, LP, Lewistown, PA.) at the American Museum of Natural History (AMNH) and an ACTIS high-resolution CT scanner (Varian Medical Systems, Inc., Palo Alto, CA.) at the University of Texas at Austin (Table S1 in File S1). Each specimen was scanned at 140–160 kV, 130–160 µA, and a resolution of 0.05–0.2 mm (Table S1 in File S1). Specimens were reconstructed using VG Studio Max v 2.2 (Volume Graphics GmbH, Heidelberg, Germany) and exported as a Stereo Lithography (.stl) file. The stl file was converted to a Stanford polygon (.ply) file using MeshLab v1.3.1 (Visual Computing Lab – ISTI – CNR, http://meshlab.sourceforge.net/) for use in analytical software

### Geometric Morphometric Analyses

**Figure 1:**
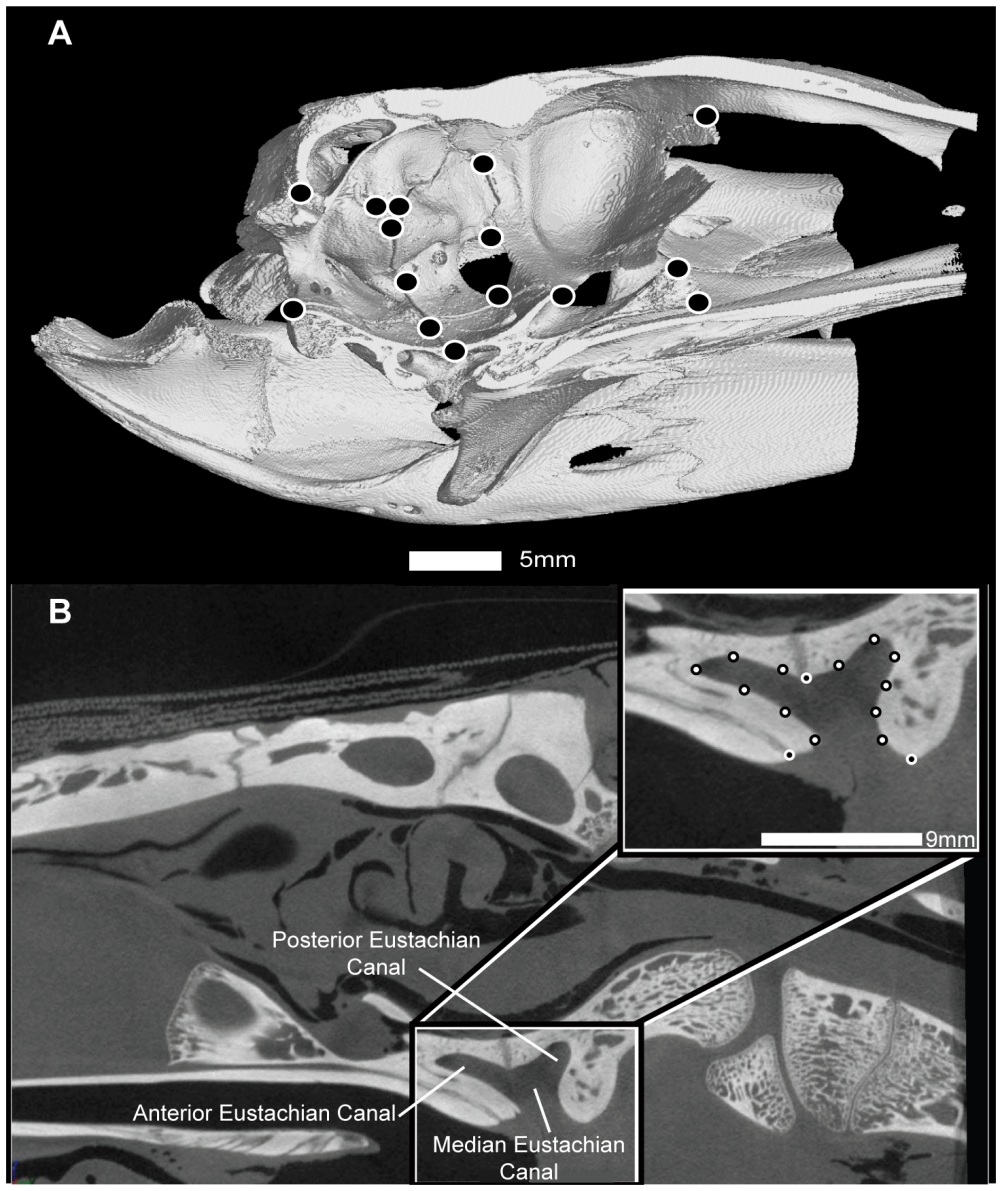
Landmark configurations for the braincase (A) and Eustachian systems (B). A: The braincase analysis used 15 Type I and Type II landmarks (Table 1). Specimen number AMNH R15163 *Crocodylus acutus*. B: Exemplary midsagittal slice and landmark placement for Eustachian system analysis. Inset diagram shows the landmark configuration. Black circles with white borders are Type I and II landmarks; white circles with black borders are sliding semi-landmarks. Specimen number AMNH R81802 *Gavialis gangeticus*.

Geometric morphometric analyses took place in three phases. In phase one, 15 Type I and Type II landmarks were sampled for 29 specimens in three-dimensions (Fig. 1A). Landmarks were placed and exported using the software Landmark v3.6 [41]. These landmarks were compiled into a tps-format file, imported into MorphoJ v1.05f [43], and aligned using generalized Procrustes superimposition [43], [44]. This alignment method was chosen over others because many of the bones of the braincase are thought to shift during ontogeny [29], [30]. Procrustes superimposition allows for the *a priori* assumption that any changes landmarks undergo would cascade across the skull as other landmarks necessarily shift in response. Other superimposition methods are best suited for cases in which a few landmarks shift more radically than others (e.g. resistant fit theta-rho; [44]). The superimposed landmarks were subjected to a Principal Components Analysis (PCA) to examine the shape changes associated with Principal Components (PC) axes. A regression of PC scores against log-transformed centroid size (a proxy for specimen size) [33] was performed for each major PC axis (containing >10% of the variation, or the first two axes if the second contained <10% of the variation). These regressions were performed in reference to family in order to look for patterns of specimen groupings.

In phase two, midsagittal images were captured from VG Studio Max. 2D landmarks identical to those used in phase one (Fig. 1A) were digitized from these midsagittal images using Image J 1.46r [42]. These landmarks were also compiled into a tps-format file and imported into MorphoJ v1.05. Alignment, PCA, and regression analyses performed in phase one were also run on the data collected in phase two.

In phase three, midsagittal images captured from VG Studio Max were used to analyze the shape differences in the Eustachian system of this clade. Fifteen 2D sliding semi-landmarks were digitized for 41 specimens using ImageJ 1.46r. These 15 landmarks comprised one Type I, two Type II, and 12 sliding semi-landmarks (Fig. 1B). The exported landmark and semi-landmark data file was converted to a sliders file with tpsUtil [45], aligned with generalized Procrustes superimposition, and analyzed by conducting a relative warps analysis (RWA: a PCA of partial warp scores, used when sliding semi-landmarks are employed) in tpsRelw [46] to examine landmark loading of component axes on each shape variable. Additionally, the superimposed landmarks were imported into MorphoJ v.1.05F [43] and subjected to a Canonical Variate analysis (CVA) to compute axes that maximally separate group means. These methods are utilized to analyze the shape similarities in braincases and the Eustachian system of crocodylians to evaluate whether these features corroborate the disparate morphological phylogenies in this clade.

Size was the most influential factor in both the braincase and Eustachian system analyses (see Results). To normalize for the overall allometric signal in the pooled shape data, the Procrustes coordinates for the braincase and Eustachian datasets were imported into R [47] and regressed against log centroid size (as a proxy for specimen size). The residuals from this regression were used in a new PCA and RWA and these new scores and coordinates were used in a separate cladistic analysis.

### Treatment of morphometric data for Cladistic Analyses

Each morphometric dataset includes negative numbers, which cannot be analyzed with standard parsimony analysis software (e.g. TNT; [48]). To adjust for this, each file was imported into Microsoft Excel and an arbitrary value of 1 was added to the data, eliminating the negative values [48]. Each of these datasets contained continuous data and were ‘analyzed as such’ (37). That is, analyses were not performed with the algorithm for raw landmark data [38], [39], but rather using each morphometric character as a continuous numerical variable.

### Cladistic Analyses

How to best incorporate these data in cladistic analyses remains debated [39], [49]. Herein, multiple types of morphometric data (e.g. Procrustes superimposed landmarks, PCA scores, RWA scores) were included in 16 cladistic analyses (Table S2 in File S1). These can be divided into two sets.

The first set of cladistic analyses were performed using morphometric data from the 2D braincase analysis. Procrustes aligned landmark coordinates and PCA scores were extracted from MorphoJ and analyzed independently and in conjunction with discrete morphological and molecular data (totaling 4 analyses). Next, size was regressed out of the morphometric data and a new set of Procrustes aligned coordinates and PCA scores were generated. These coordinates and PCA scores were also used independently in cladistic analyses and together with discrete morphological and molecular characters (totaling 4 analyses). Therefore, this first set of cladistic analyses contained eight independent analyses.

The second set of cladistic analyses used morphometric data from the Eustachian system analysis of 2D sliding semi-landmarks. These data were extracted from tpsDIG and imported into MorphoJ to perform a regression against log centroid size. The residual Procrustes coordinates and RWA values were extracted and used in their own analyses. As with the first set of cladistic analyses, Procrustes aligned coordinates and RWA scores were analyzed independently and with discrete morphological and molecular data (totaling 4 analyses). Size was then removed from these data, generating new Procrustes aligned coordinates and RWA scores were generated. These new values were used independently in cladistic analyses (totaling 4 analyses). The second set of cladistic analyses contained eight independent analyses in total. Consistency and retention indices for each of these 16 analyses was performed using the stats.run script (http://tnt.insectmuseum.org/index.php/Scripts).

For the combined analyses, the morphometric data were added to 169 morphological characters (compiled by C.A.B and available on morphobank, project #1154, and in File S2) and to 11,564 aligned base pairs (morphobank, project #1154). The molecular data comprised published sequences from GenBank for 13 mitochondrial genes: ATP6, ATP8, Cytochrome B, Cytochrome C (subunits 1, 2, and 3), NADH (subunits 1, 2, 3, 4, 4l, 5, and 6) for 16 taxa that were aligned with ClustalX [50]. A preliminary study of alignment method showed that variable alignments did not result in topology differences (Gold, pers. obs.). Because there were multiple specimens of the same species for some of the morphometric data in this study, morphological and molecular data for those species had to be duplicated so that the three datasets would agree in character number. These individuals were labeled “Genus_species”, “Genus_species_2”, as needed. A summary of character number, outgroup taxon, number of most parsimonious trees (MPTs) found, and tree length can be found in Tables S2 and S3 in File S1, for the analyses using braincase data and Eustachian data, respectively. For comparison, a cladistic analyses using only discrete morphological and molecular data was also performed (Table S4 in File S1 contains these results).

**Table 1:** Landmark number, type, and description for the 3D and 2D braincase geometric morphometric analyses.

Landmark Number	Landmark Type	Description*
0	Type 1	Lateral suture of eo and bo
1	Type 1	Intersection of medial suture of bo and bs with cutting plane
2	Type 2	Ventral maximum curvature of bsr
3	Type 2	Dorsal maximum curvature of bsr
4	Type 1	Anterior suture of bs with ls
5	Type 1	Ventral suture of pot with ls
6	Type 1	Posterior ventral point on pot above canal
7	Type 1	Lateral suture of bo and bs
8	Type 1	Dorsal intersection of pot and ls suture with cranial nerve V opening
9	Type 1	Suture of ls with f
10	Type 1	Ventral point of parietal between pot and ls
11	Type 1	Dorsal suture of pot with ex
12	Type 1	Dorsal suture of pot with so
13	Type 1	Rostrodorsal suture of ex with so
14	Type 2	Caudal most midsagittal point on skull, btwn exo and so

*Abbreviations: bo – basioccipital, bs – basisphenoid, bsr – basisphenoid rostrum, eo – exoccipital, f – frontal, ls – laterosphenoid, pot – prootic, so – supraoccipital.

Cladistic analyses were performed with TNT [51]. A traditional search was run with 1 random seed and 1000 replications of Wagner trees using tree bisection reconnection (TBR). Each replication saved 10 trees. Once these MPTs were found, a second iteration of analyses was performed using the MPTs saved in RAM from the first analysis. Strict consensus trees were created when more than one MPT was found from the second round of TBR. Nodal support was determined using bootstrap (1000 replicates) and Bremer support. Bremer support was analyzed by first running a New Technology Search using Ratchet, Fuse, Drift, and Sectorial Search, using 10 initial addition sequences and finding a minimum length 10 times. Once new MPTs were found, the bremer.run script (http://tnt.insectmuseum.org/index.php/Scripts) was run to search for suboptimal trees of 10 additional steps for the combined analyses and 5 additional steps for the morphometric data analyses. All trees were inspected by eye for congruency in relationships.

## Results

### Three-dimensional analysis of the braincase

The PCA of 3D braincase landmarks indicated that changes along the first two axes do not differentiate crocodylian clades. The PCA of 29 specimens recovered two major axes accounting for 52% and 9.9% of the variation, respectively. All the other axes accounted for less than 10% of the total variation and will not be further discussed. The landmarks that most affected PC1 include anterior shifting of the frontal–laterosphenoid suture, dorsoventral enlargement of the basisphenoid rostrum, ventral displacement of the parietal, and slight posteroventral extension of the basioccipital (Fig. S1). On PC2, the shape changes are primarily associated with posterior shifting of the frontal-laterosphenoid suture, anteroventral displacement of the parietal, and expansion of the foramen magnum via dorsal translation of the supraoccipital–exoccipital contact and posteroventral shifting of the basioccipital–exoccipital contact. Additionally, many of the prootic, basisphenoid, exoccipital, and supraoccipital elements showed torsion through anterodorsal displacement of the posterior landmarks and posteroventral displacement of the anteroventral landmarks (Fig. S1).

**Figure 2:**
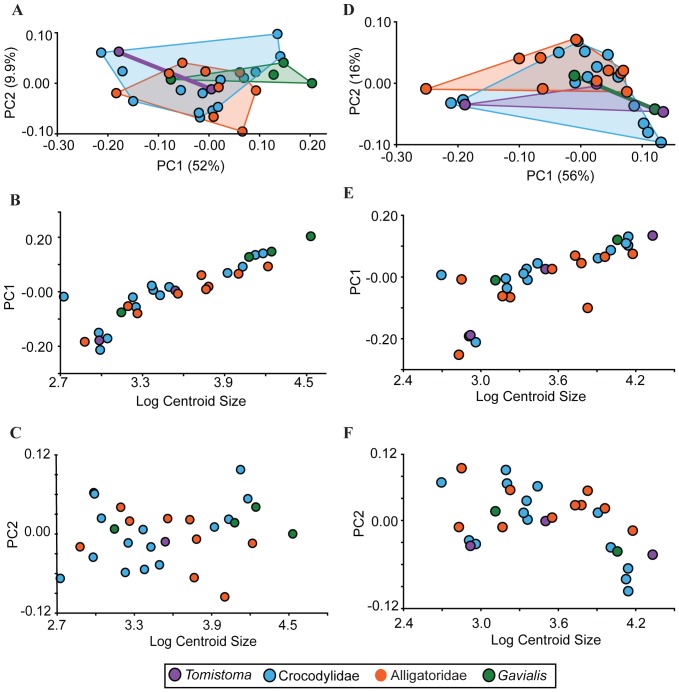
Results from 3D and 2D PCA and regressions of PC1, PC2 against log centroid size. A: Results from the 3D PCA showing broad overlap of the crocodylian families. B: Regression of 3D PC1 against log centroid size. PC1 was highly correlated with size (R^2^ = 0.201, p<0.0001). C: Regression of 3D PC2 against log centroid size. PC2 was not correlated with size (R^2^ = 0.0157, p = 0.0157). D: Results from the 2D PCA showing broad overlap of crocodylian families. E: Regression of 2D PC1 against log centroid size. PC2 was highly correlated with size (R^2^ = 0.166, p<0.0001). F: Regression of 2D PC2 against log centroid size. PC2 was not correlated with size (R^2^ = −0.0436, p = 0.0036). Members of Crocodylidae (blue circles) and Alligatoridae (orange circles) broadly overlap with *Gavialis* (green circles) and *Tomistoma* (purple circles). Color-coded polygons indicate the furthest extent of each group based on the data points.

A bivariate plot of PC2 versus PC1 showed that members of Alligatoridae and Crocodylidae broadly overlap in morphospace, as does *Gavialis* (Fig. 2A). Because there are only few specimens of *Tomistoma*, it is impossible to determine the morphospace for this species; however, generally, *Tomistoma* overlaped with the other specimens in the analysis. A regression of PC1 on log centroid size showed a significant correlation (R^2^ = 0.201, p<0.0001; Fig. 2B). Sampled species largely aligned on a common allometric trajectory: smaller specimens graded into larger ones along this regression. However, a regression analysis of PC2 on log centroid size did not imply significant correlation (R^2^ = 0.0157, p = 0.397; Fig. 2C).

### Two-dimensional analysis of the braincase

The PCA of 27 specimens also returned two major axes, accounting for greater than 60% total variation (56% and 16%, of the variation, respectively). Anteroposterior shifting of landmarks, and ventral translation of the ventral landmarks were the key shape changes occurring along PC1 (Fig. S2). The outer landmarks expanded outwards, and the inner landmarks compressed inwards. The second PC axis accounted primarily for dorsoventral compression of the basisphenoid rostrum and a ventral shift in the anterior edge of the basioccipital (Fig. S2). The plot of PC2 versus PC1 indicated broad overlap between major groups, with both *Tomistoma* and *Gavialis* positioned closer to Crocodylidae than to Alligatoridae (Fig. 2D). A regression of the first two PC axes against log centroid size showed a significant correlation for PC1 (R^2^ = 0.166, p<0.0001; Fig. 2E). Again, sampled species aligned on a common allometric trajectory. PC2 fails to find a significant correlation with centroid size (R^2^ = −0.0436, p = 0.0036; Fig. 2F).

### Two-dimensional Analysis of the Eustachian System

**Figure 3:**
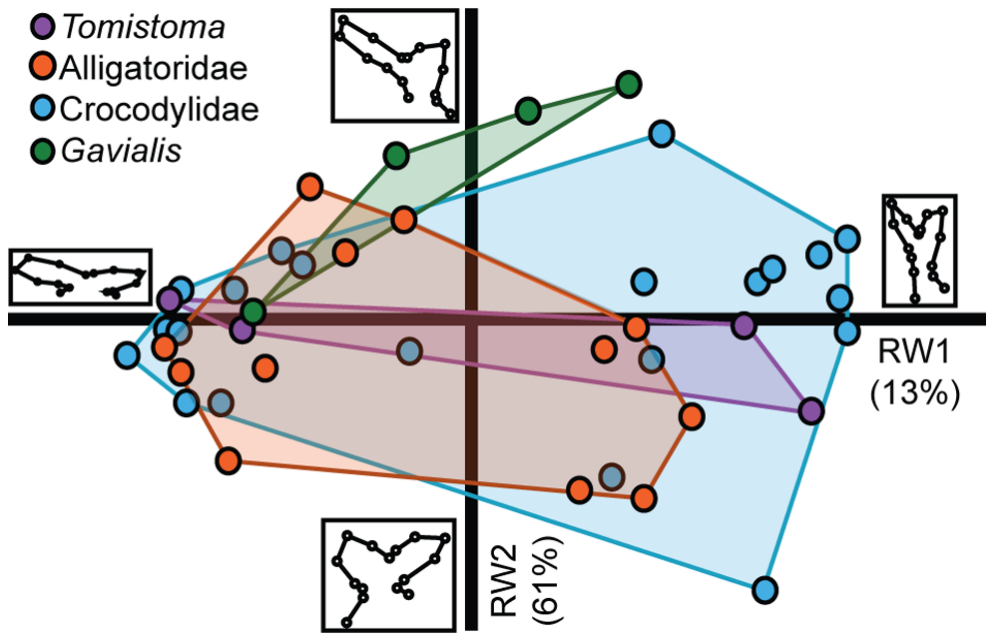
Plot of RW1 versus RW2 from the 2D analysis of the Eustachian system. Larger and smaller specimens are located towards the positive and negative ends of RW1, respectively. Members of Alligatoridae (orange) generally overlap with members of Crocodylidae (blue). *Tomistoma* (purple) overlaps with both Alligatoridae and Crocodylidae. *Gavialis* (green) occupies morphospace that is mostly unoccupied by other crocodylians, except the smallest *Gavials*, which overlaps the *Tomistoma* morphospace. Inset diagrams at the extremes of each axis show the landmark configuration for the starred location on that axis. Along RW1, Larger specimens have a more vertically oriented Eustachian canal than smaller specimens, for which the canal is more horizontal. Changes along RW2 have to do with the relative shape of the anterior and posterior Eustachian canals.

The RWA on the Procrustes shape coordinates of 41 specimens returned two significant axes that explain 61% and 13% of the variation, respectively. The RWA shows broad overlap between Crocodylidae and Alligatoridae (Fig. 3). Notably, *Tomistoma* spans morphospace that overlaped with these two groups. *Gavialis*, however, except for the smallest specimen, occupied a region of morphospace mostly separate from the others. The smallest specimen lied within the *Tomistoma* distribution.

Even though some specimens formed tight clusters with others, these clusters were not monospecific. The first RW axis described changes in the overall shape of the Eustachian system, from horizontally oriented canals in the negative morphospace to vertical canals in the positive morphospace (Fig. 3 inset diagrams). Along positive RW2, the posterior point of the medial Eustachian canal is more dorsally positioned than the anterior point, and the posterior Eustachian canal is subequal to the anterior canal. The negative scores along RW2 contained Eustachian systems that have almost equally positioned anterior and posterior points, and the anterior canal is approximately twice the size of the posterior canal.

The CVA on shape data resulted in two key CV axes (accounting for 53% and 40% of the variation, respectively; Fig. S3) maximally separating the larger clades. The specimens are clearly divided based on traditional groups, and permutation tests show significant differences between them (p<0.0005 for each pairwise comparison) except for the difference between *Tomistoma* and *Gavialis*, which had a significance value of p = 0.0254, and between *Tomistoma* and the crocodylids (p = 0.6881). *Tomistoma* fell within the crocodylids, but *Gavialis* formed its own cluster. Changes along CV1 included the ventral lengthening of the median canal, as well as a decrease in the size of the anterior and posterior canals (Fig. S4). Canonical Variate axis two accounted for changes that include ventral shifting of the anteroventral border and anterodorsal shifting of the posterior border of the median Eustachian canal. Other changes along this axis were a posterior shift in the anterodorsal landmarks (numbers 7–9) and a dorsal shift of the posterodorsal landmarks (numbers 10–14) (Fig. S4).

### Cladistic Analyses

**Figure 4:**
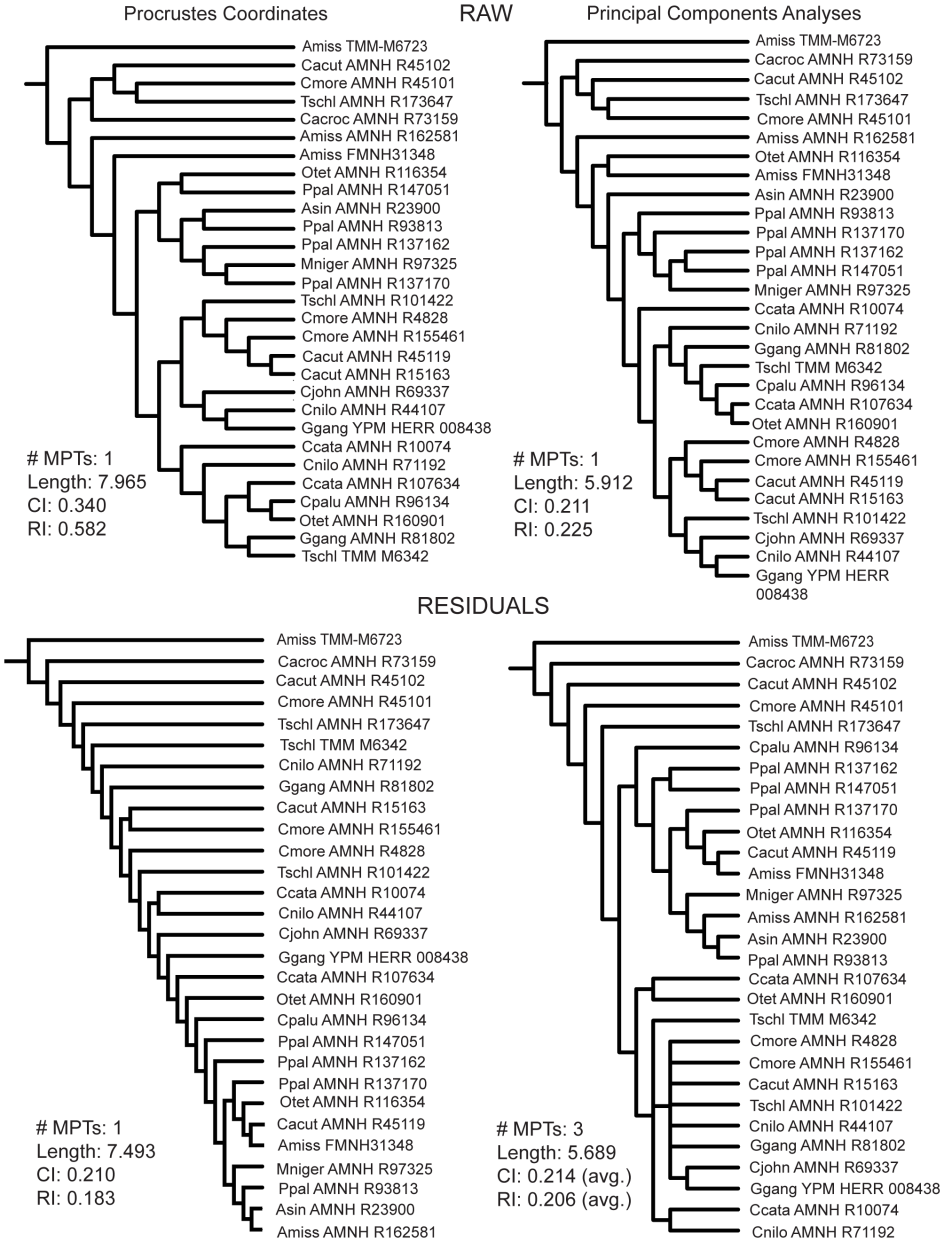
Phylogenetic results from using 2D Braincase morphometric data. Top left: Cladogram from using only Procrustes coordinates. Top right: Cladogram from using only PCA scores. Bottom left: Cladogram from using Procrustes coordinates once size was regressed out (residual Procrustes coordinates). Bottom Right: Cladogram from using PCA scores once size was regressed out (residual PCA scores). #MPTs: Number of most parsimonious trees; Length: tree length; CI: consistency index; RI: retention index.

The cladistic analyses of the braincase data showed similar results using the aligned Procrustes coordinates and the PCA scores in both raw and residual analyses (Fig. 4). All of these analyses independently resulted in the formation of a clade of non-alligatorid crocodylians. Every *Gavialis* specimen was contained within this clade. There were only two crocodylids not included in that clade - a small Morelet's crocodile (*C. moreletii*; skull length = 39 mm) and a small dwarf crocodile (*O. tetraspis*; skull length = 52 mm). Using the raw data, a clade of tiny (<5 cm skull length) specimens fell out at the base of the tree that became paraphyletic when the residuals from common allometric components were used. Additionally, in the analysis employing residual PCA scores, a small *T. schlegelii* (skull length = 49 mm) joined the paraphyletic tiny group. The residual Procrustes coordinate-only analyses produced a different topology that is comb-like (Fig. 4). Tree length decreased when the residual data are used; however, CI and RI also decreased. There was no bootstrap or Bremer support for the nodes in these analyses.

**Figure 5:**
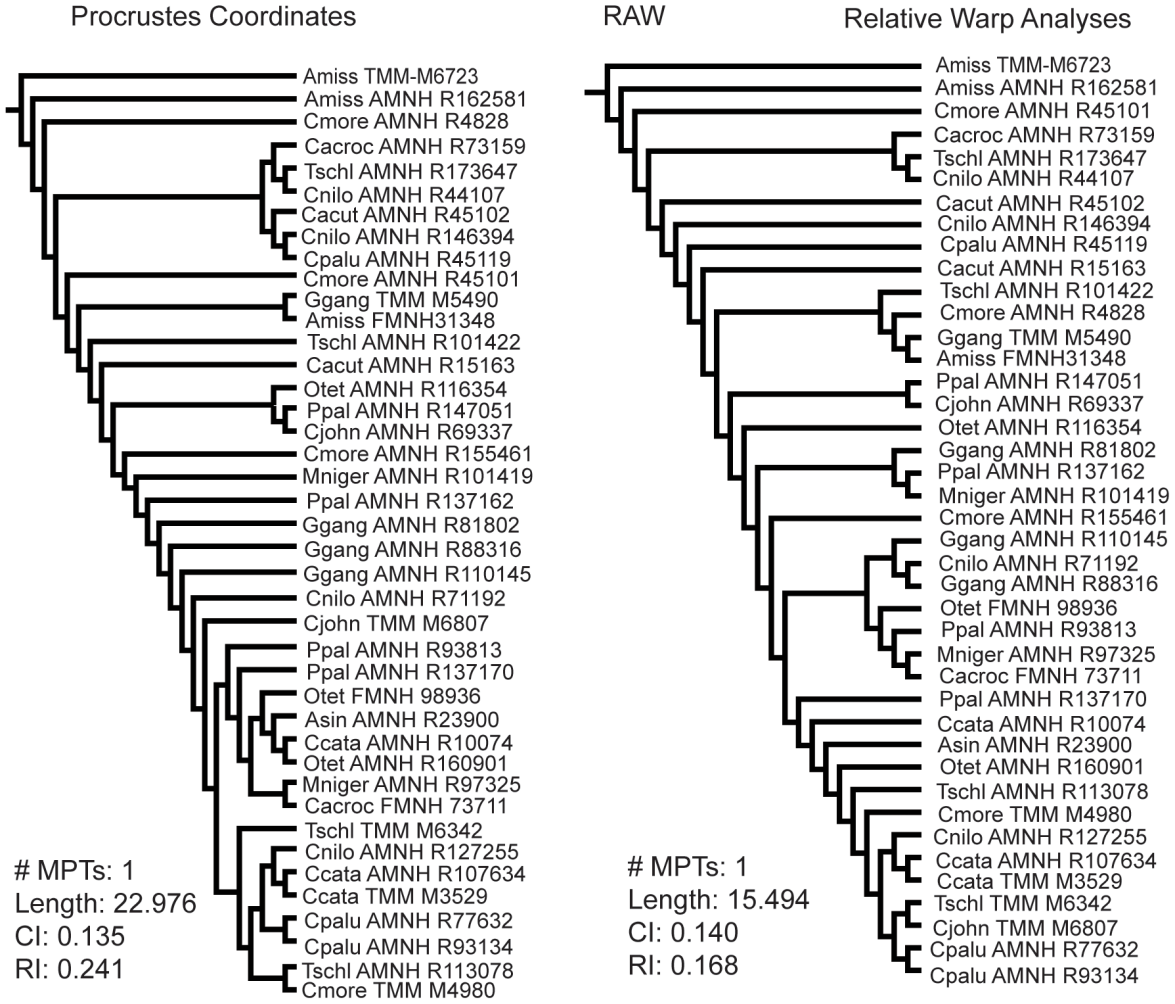
Phylogenetic results from using the raw Eustachian system morphometric data. Left: Cladogram from using only Procrustes coordinates. Right: Cladogram from using only RWA scores. #MPTs: Number of most parsimonious trees; Length: tree length; CI: consistency index; RI: retention index.

**Figure 6:**
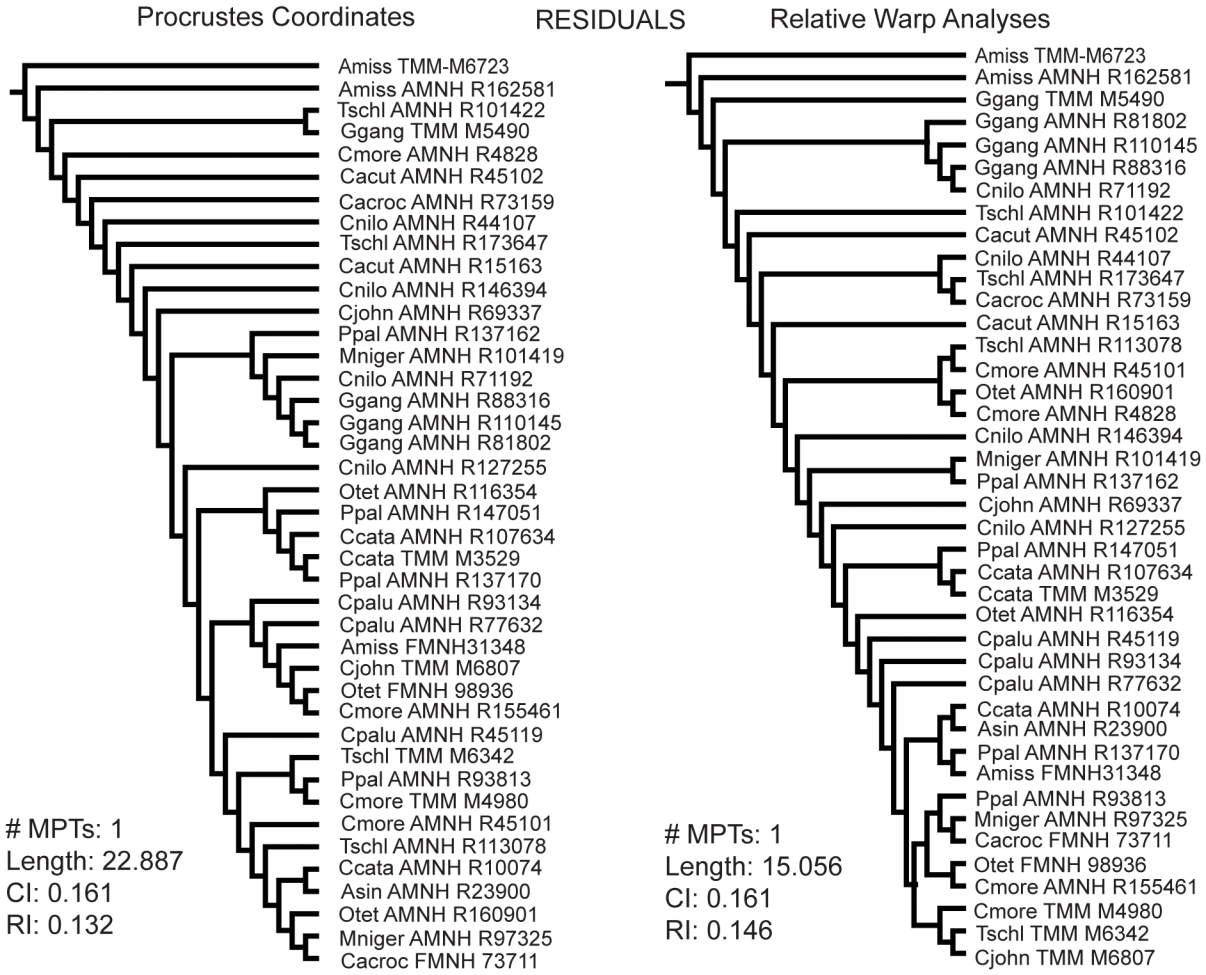
Phylogenetic results from using the residual Eustachian system morphometric data. Left: Cladogram from using Procrustes coordinates once size was regressed out (residual Procrustes coordinates). Right: Cladogram from using RWA scores once size was regressed out (residual PCA scores). #MPTs: Number of most parsimonious trees; Length: tree length; CI: consistency index; RI: retention index.

The Eustachian data produced four different topologies - one for each set of morphometric data used (Fig. 5, 6). The raw Procrustes coordinates produced a tree that had three out of four *Gavialis* specimens as a paraphyletic grade. The raw RWA scores resulted in each *Gavialis* specimen as sister to another crocodylian of lesser skull length. The residual Procrustes coordinates resulted in a clade with three of four *Gavialis* specimens that are deeply nested in the tree. This clade was reproduced with the residual RWA scores; however, in this result it also included one Nile crocodile (*C. niloticus*), and the clade lied at the base of the tree instead of deeply nested within it. Tree length decreased when the residual data are used instead of raw data for Procrustes coordinate and RWA datasets. The CI and RI values stayed low in each case. These analyses showed very low bootstrap and Bremer support.

**Figure 7:**
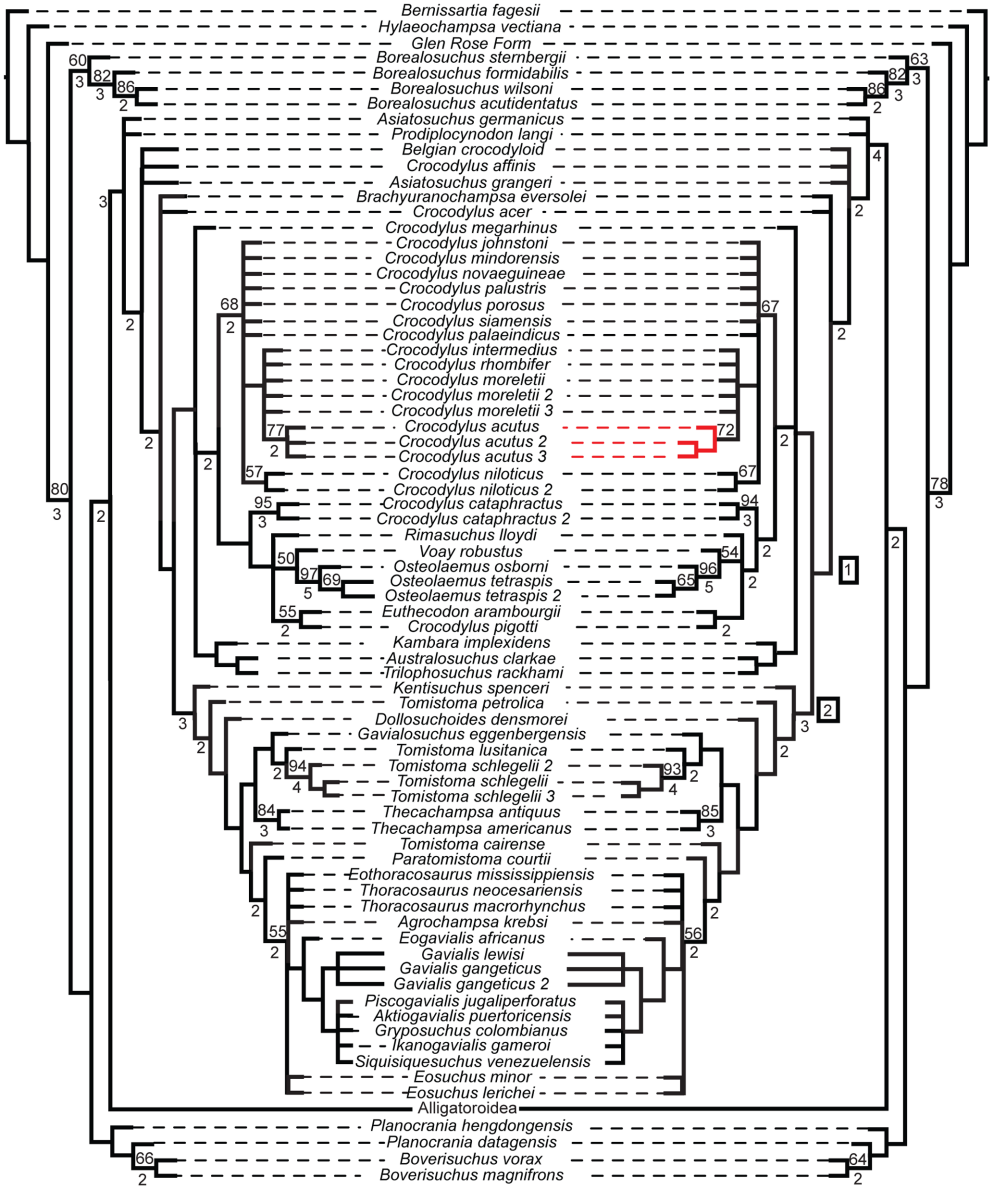
Phylogenetic tree from morphological and molecular data (left) and total evidence (right), with Alligatoroidea collapsed. Both analyses return *Gavialis* within Crocodylidae, supporting the molecular hypothesis of Crocodylia. The morphological and molecular data created 360 MPTs length of length 17534. The total evidence tree resulted in 360 MPTs length 17541.530. The strict consensus of each is shown. Numbers in boxes denote clades: 1 - Crocodylidae, 2 - Gavialidae. Bootstrap and Bremer values are shown above and below each corresponding node, respectively. Red branches indicate different relationships between the two trees.

**Figure 8:**
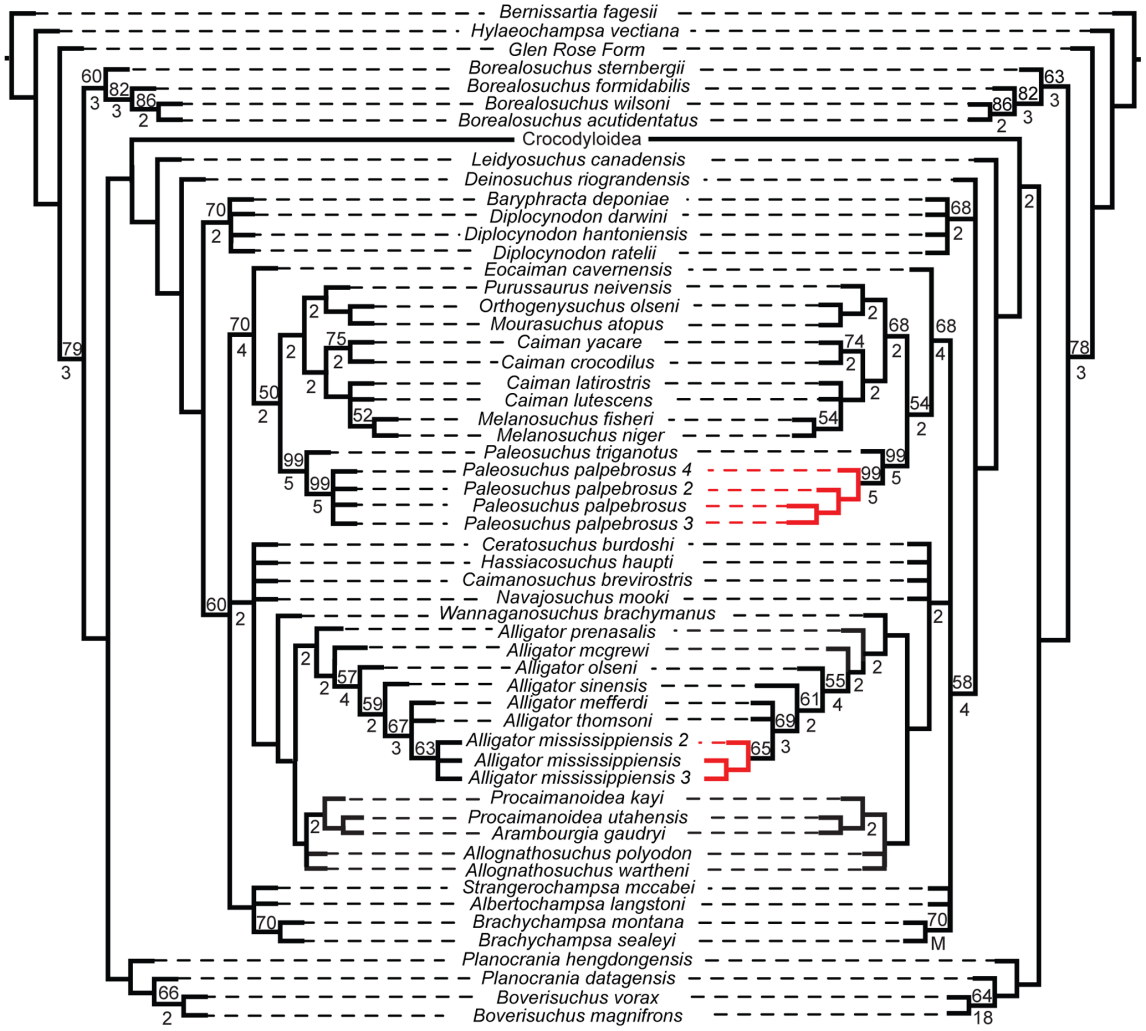
Phylogenetic tree from morphological and molecular data (left) and total evidence (right), with Crocodyloidea collapsed. Both analyses return congruent results, with very little change in supporting values. The morphological and molecular data created 360 MPTs length of length 17534. The total evidence tree resulted in 360 MPTs length 17541.360. The strict consensus of each is shown. Bootstrap and Bremer values are shown above and below each corresponding node, respectively. Red branches indicate different relationships between the two trees.

The effect of these morphometric data when added to discrete morphological and molecular characters was nearly identical regardless of which data are used. Their inclusion resulted in a reduced resolution in many clades, regardless of whether or not that particular terminal had morphometric data associated with it, when compared to the tree of morphological and molecular data (Fig. 7, 8). Nevertheless, they all produced trees consistent with those supported by molecular data in which *Gavialis* and *Tomistoma* form a monophyletic Gavialidae more closely related to Crocodylidae than to Alligatoridae (Fig. 7). The topologies only differed in intraspecific structure (e.g. the clade comprising *Paleosuchus palpebrosus*, *Paleosuchus palpebrosus*_2, *Paleosuchus palpebrosus*_3, and *Paleosuchus palpebrosus*_4). Bootstrap values supported this by showing only minute changes between the values at well-supported nodes (i.e. >50% bootstrap values).

## Discussion

Even though the braincase data alone showed no distinct groupings based on crocodylian higher taxa, when these data were regressed against log centroid size, they indicated that PC1 was significantly correlated with size. A regression along PC2 showed no significant correlation with log centroid size, and therefore phylogenetic information could be contained within subsequent principal component axes. The Eustachian system analysis resulted in *Gavialis* occupying a unique morphospace as larger individuals (>200 mm skull length), but overlapping with the crocodylids and alligatorids at a smaller size (<200 mm skull length). This shows that ontogenetic changes drive *Gavialis* away from other crocodylians morphologically, potentially showing a secondarily derived condition. *Tomistoma* also shared morphospace with crocodylids and alligatorids. Size and shape of the median Eustachian canal were most influential over RW1. Larger individuals have a more vertical canal and smaller individuals have a more horizontal canal.

This pattern of ‘verticalization’ *sensu* [29], [30] during growth is corroborated. The basisphenoid and basioccipital ventrally expand, changing the shape and orientation of the median Eustachian canal. Brochu [8] observed that derived gavialoids bear a different condition from more basal fossil relatives. Although derived gavialoids of Late Eocene age and younger, such as *Eogavialis*, the gryposuchines, and *Gavialis*, have a distinct Eustachian system morphology, more basal forms - the so-called Late Cretaceous through Paleocene “thoracosaurs” - have systems resembling those of other mature extant crocodylians [8], [52]. Closely related groups to Crocodylia also have similar Eustachian configurations [5], [6], and thus the highly derived condition in extant *Gavialis* is consistent with both the molecular and the morphological phylogenetic hypotheses. Even though both the braincase and Eustachian system have been observed to provide phylogenetic information, neither had yet been analyzed morphometrically and incorporated into a cladistic analysis.

We analyzed novel morphometric data and performed cladistic analyses using only these data. When morphometric data were analyzed alone they resulted in two findings. Firstly, the raw braincase morphometric data grouped *Gavialis* within a clade that included no alligatorids, seemingly supporting molecular and combined phylogenetic reconstructions. However, the residual braincase morphometric data showed no consistent relationships, even separating specimens of the same species. Secondly, the Eustachian system data grouped specimens of *G. gangeticus* together as a clade at the base of the tree and as sequentially paraphyletic, depending on whether the Procrustes coordinates or the RW scores were used. None of the eight analyses that used only morphometric data resulted in any well-supported nodes, in fact, all bootstrap support was under 50% for all nodes. Bremer support was also poor for these trees. The lack of nodal support and the unique relationships found with the morphometric data indicate that these many of these data may not contain phylogenetic signal. The only exception could be the braincase morphometric data, which did result in a tree that separated alligatorids from other crocodylians.

In this study, when the morphometric data were added to discrete morphological and molecular characters, they each result in the identical topology in which *Gavialis* and *Tomistoma* combined are closest relatives within Crocodylidae. Even though the two sets of morphometric data supported disparate positions for *Gavialis* among crocodylians, they each result in identical trees when combined evidence is used, further corroborating the *Gavialis* - *Tomistoma* sister relationship. These results suggest that discrete morphological features, which place *Gavialis* at the base of Crocodylia, are reversals from ancestral conditions rather than plesiomorphic characteristics [1], although extinct gavialoids need to be examined to further corroborate this idea. Therefore, we provide the first cladistic analysis of morphometric data that resulted in a tree consistent with the molecular and combined phylogeny of Crocodylia, and take another step towards reconciling competing crocodylian phylogenetic signals.

## Supporting Information

Figure S1 **Landmark changes in the 3D braincase analysis along PC1 (upper) and PC2 (lower)**. Dots represent the mean shape of the pooled data. Lines represent the direction and magnitude for changes in landmark points for +0.1 PC score. A) On PC1, the landmarks with the most change are: 1: posteroventral extension of the basioccipital; 3 and 4: dorsoventral enlargement of the basisphenoid rostrum; 10: anteroventral movement of the frontal–laterosphenoid suture; and 11: ventral displacement of the parietal. B) On PC2, the landmarks with the most changes are 10: posterior shifting of the frontal-laterosphenoid suture; 11: anteroventral displacement of the parietal; expansion of the foramen magnum via dorsal translation of the supraoccipital–exoccipital contact (landmark 15) and posteroventral shifting of the basioccipital–exoccipital contact (landmark 1).
(TIF)

Figure S2 **Landmark changes in the 2D braincase analysis along PC1 (upper) and PC2 (lower).** Dots represent the mean shape of the pooled data. Lines represent the direction and magnitude for changes in landmark points for +0.1 PC score. A) On PC1, expansion of outer landmarks (numbers 1–4, 10) and compression of inner landmarks (number 6, 7, 9, 12–15) were the key shape changes. B) On PC2, the most change occurred via dorsoventral compression of the basisphenoid rostrum (landmarks 3 and 4) and a ventral shift in the anterior edge of the basioccipital (landmarks 2 and 8).
(TIF)

Figure S3 **Plot of CV1 versus CV2 from the 2D analysis of the Eustachian system.** Clear separation is observed between Alligatoridae (orange), Crocodylidae (blue), *Gavialis* (green) and *Tomistoma* (purple) indicating that the Eustachian system contains information capable of discerning crocodylian clades.
(TIF)

Figure S4 **Landmark changes along CV1 and CV2 from the CVA of Eustachian landmarks.** Changes along CV1 include the ventral lengthening of the median canal, an increase in size of the anterior Eustachian canal and a decrease in size in the posterior Eustachian canal Along CV2, the landmarks shift to create a Eustachian system that is anteroposteriorly compressed and dorsoventrally lengthened.
(TIF)

File S1 **Four tables describing scanning parameters and the parameters and results from the cladistic analyses.**
(DOCX)

File S2 **Morphological and molecular matrices used in cladistic analyses.**
(DOCX)
