# Supplementary figures and images for: An Expanded Combined Evidence Approach to the Gavialis Problem Using Geometric Morphometric Data from Crocodylian Braincases and Eustachian Systems
Source: PLoS One. 2014 Sep 8;9(9):e105793. doi: 10.1371/journal.pone.0105793 (PMC4157744; doi:10.1371/journal.pone.0105793)

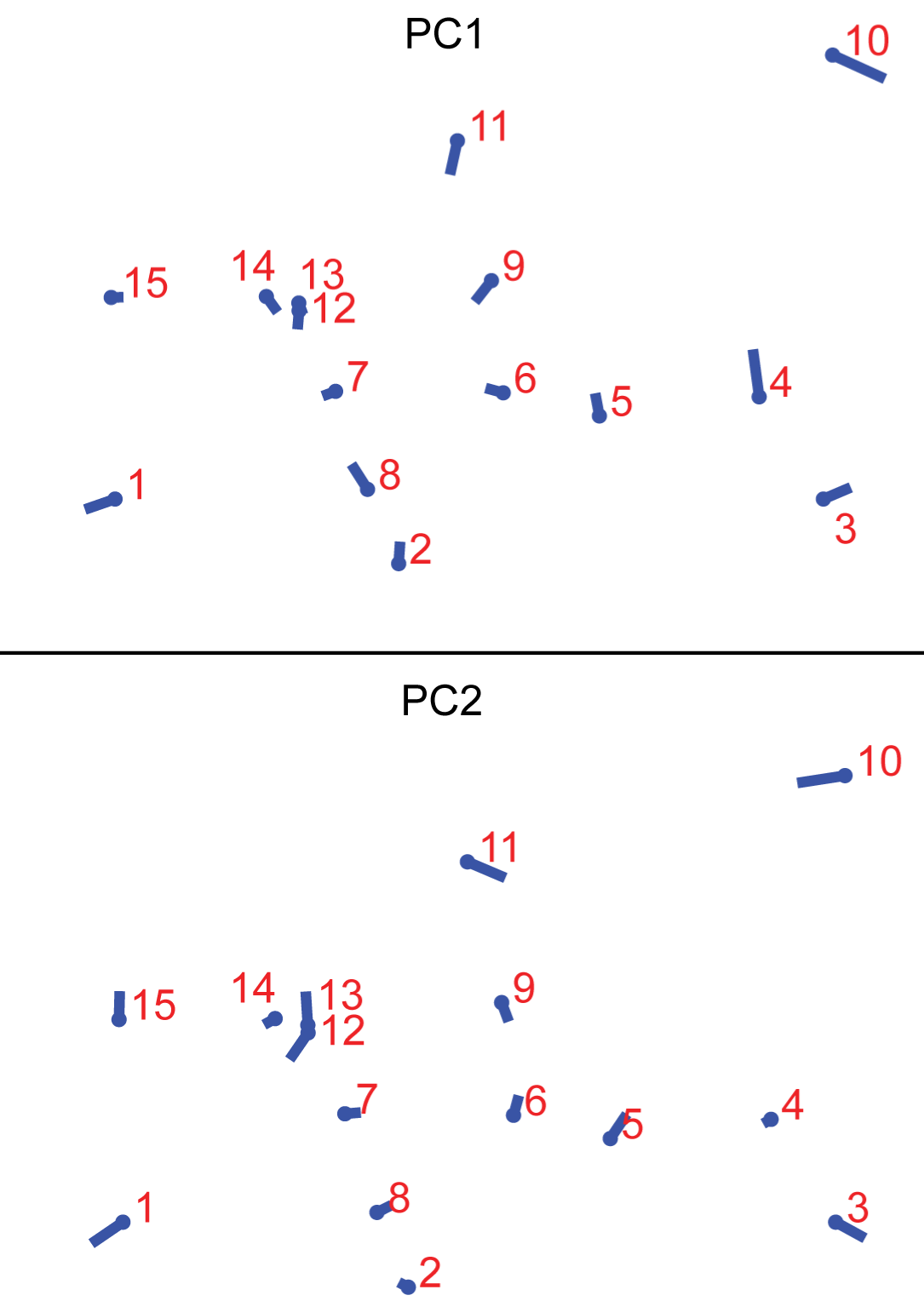

Supplement: Figure S1 — Landmark changes in the 3D braincase analysis along PC1 (upper) and PC2 (lower). Dots represent the mean shape of the pooled data. Lines represent the direction and magnitude for changes in landmark points for +0.1 PC score. A) On PC1, the landmarks with the most change are: 1: posteroventral extension of the basioccipital; 3 and 4: dorsoventral enlargement of the basisphenoid rostrum; 10: anteroventral movement of the frontal–laterosphenoid suture; and 11: ventral displacement of the parietal. B) On PC2, the landmarks with the most changes are 10: posterior shifting of the frontal-laterosphenoid suture; 11: anteroventral displacement of the parietal; expansion of the foramen magnum via dorsal translation of the supraoccipital–exoccipital contact (landmark 15) and posteroventral shifting of the basioccipital–exoccipital contact (landmark 1). (TIF) [file pone.0105793.s001.tif]

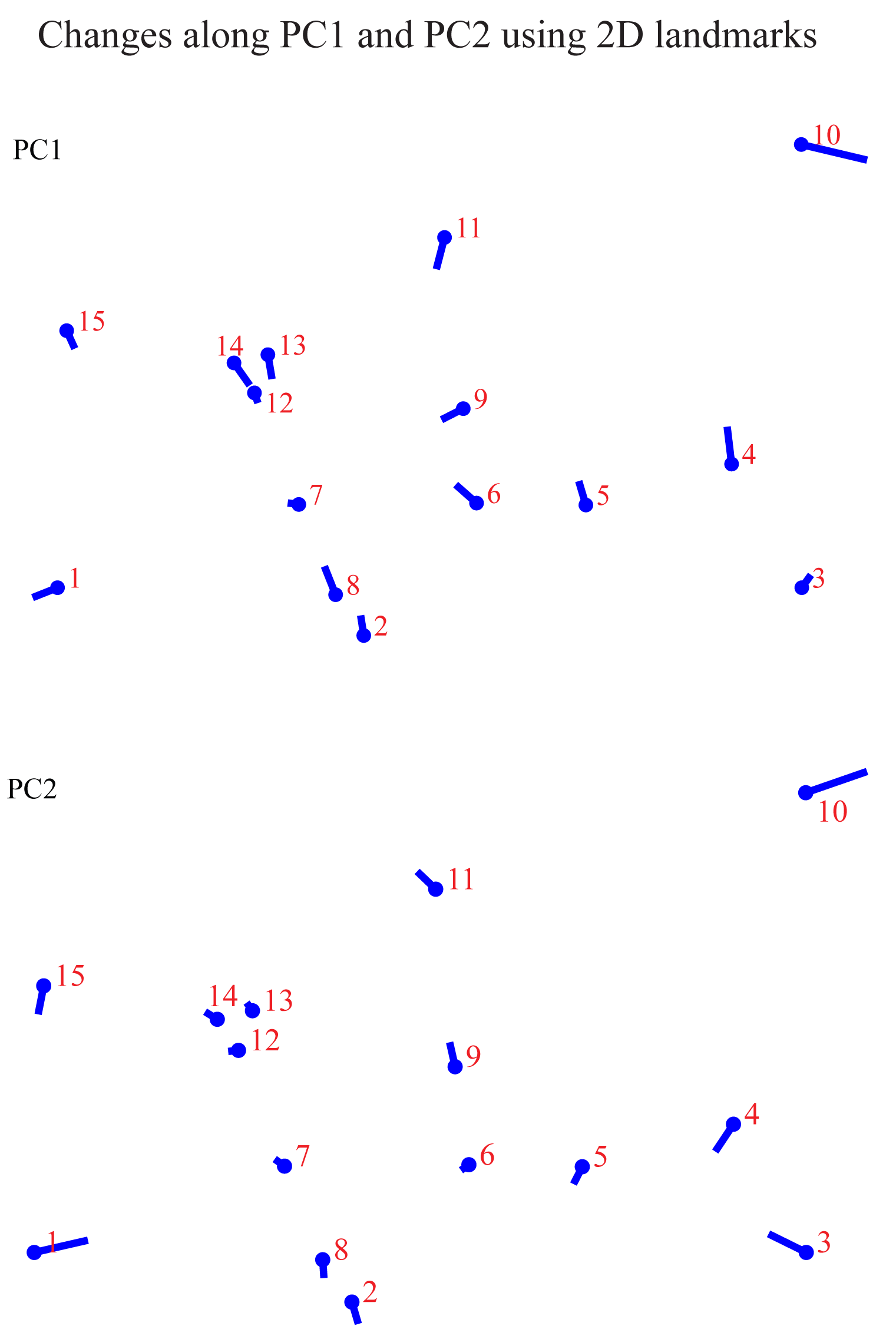

Supplement: Figure S2 — Landmark changes in the 2D braincase analysis along PC1 (upper) and PC2 (lower). Dots represent the mean shape of the pooled data. Lines represent the direction and magnitude for changes in landmark points for +0.1 PC score. A) On PC1, expansion of outer landmarks (numbers 1–4, 10) and compression of inner landmarks (number 6, 7, 9, 12–15) were the key shape changes. B) On PC2, the most change occurred via dorsoventral compression of the basisphenoid rostrum (landmarks 3 and 4) and a ventral shift in the anterior edge of the basioccipital (landmarks 2 and 8). (TIF) [file pone.0105793.s002.tif]

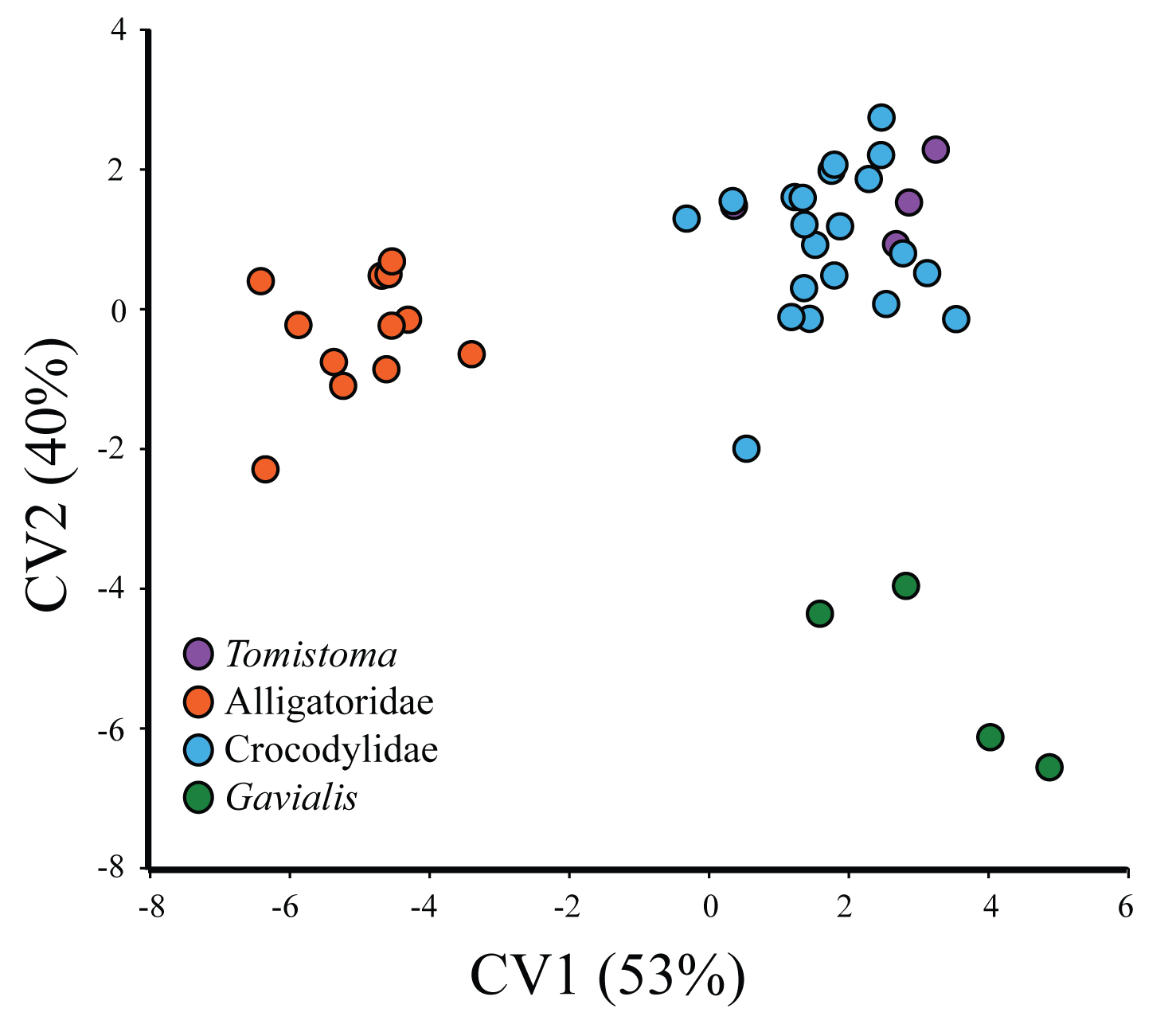

Supplement: Figure S3 — Plot of CV1 versus CV2 from the 2D analysis of the Eustachian system. Clear separation is observed between Alligatoridae (orange), Crocodylidae (blue), Gavialis (green) and Tomistoma (purple) indicating that the Eustachian system contains information capable of discerning crocodylian clades. (TIF) [file pone.0105793.s003.tif]

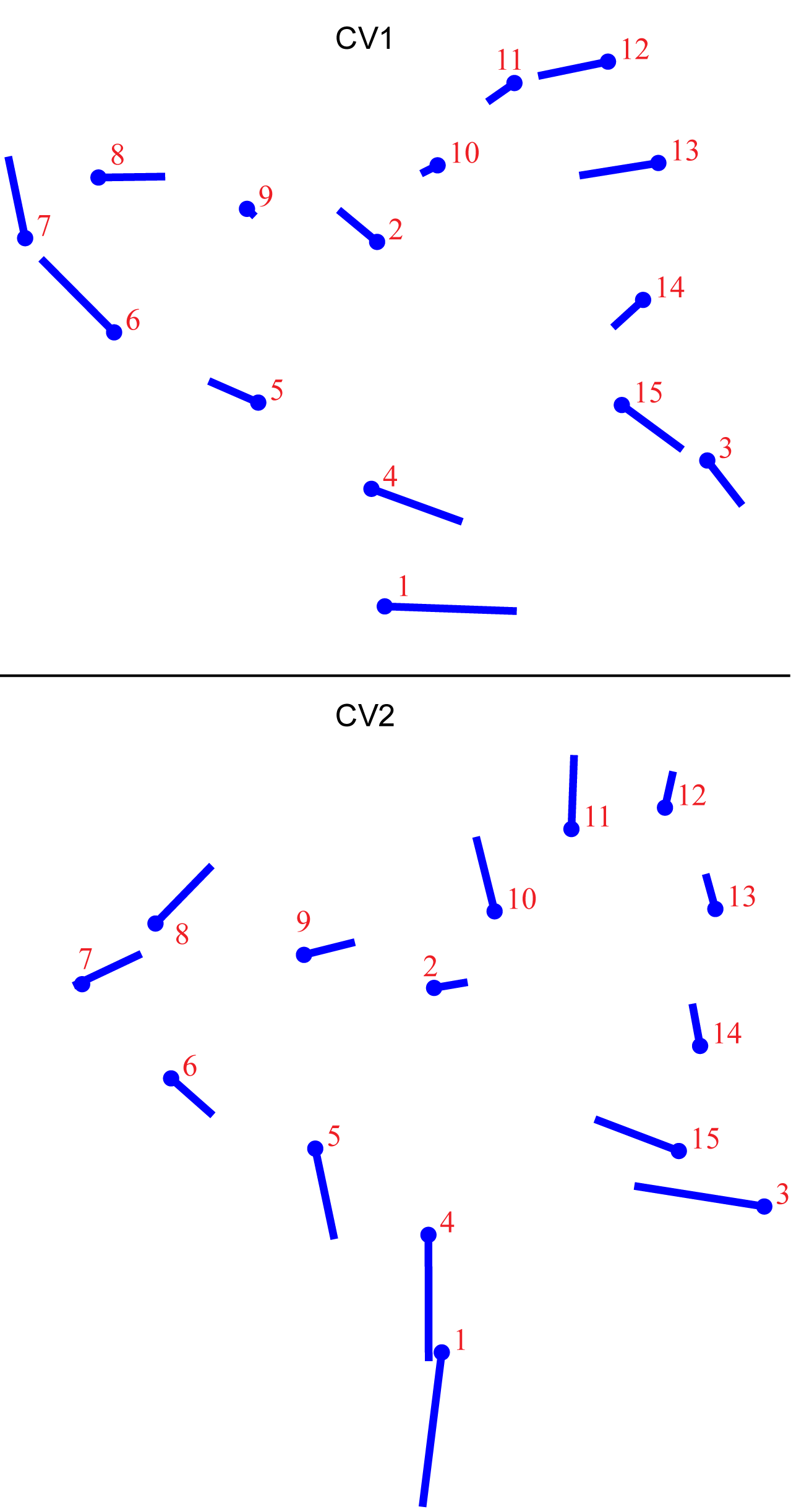

Supplement: Figure S4 — Landmark changes along CV1 and CV2 from the CVA of Eustachian landmarks. Changes along CV1 include the ventral lengthening of the median canal, an increase in size of the anterior Eustachian canal and a decrease in size in the posterior Eustachian canal Along CV2, the landmarks shift to create a Eustachian system that is anteroposteriorly compressed and dorsoventrally lengthened. (TIF) [file pone.0105793.s004.tif]
