# Supplementary material for: An Expanded Combined Evidence Approach to the Gavialis Problem Using Geometric Morphometric Data from Crocodylian Braincases and Eustachian Systems
Source: PLoS One. 2014 Sep 8;9(9):e105793. doi: 10.1371/journal.pone.0105793 (PMC4157744; doi:10.1371/journal.pone.0105793)
Supplement: File S1 — Four tables describing scanning parameters and the parameters and results from the cladistic analyses. (DOCX) [file pone.0105793.s005.docx]

Supplementary Data:

There are 4 tables included in this information:

Table S1: Scanning parameters for each specimen and in which analysis it was used.

Table S2: Parameters and results for the cladistic analysis using braincase morphometric data.

Table S3: Parameters and results for the cladistic analysis using Eustachian system morphometric data.

Table S4: Parameters and results for the cladistic analysis of only discrete morphological and molecular characters.

Table S1: Specimens, scanning parameters, and included analyses for each specimen.

|  |  |  |  |  |  | Analyses | | |
| --- | --- | --- | --- | --- | --- | --- | --- | --- |
| Specimen Number* | Species | Voltage (kV) | Current (μA) | Slices | Resolution (mm)^1^ | 3D | 2D | Eustachian |
| AMNH R73159 | *Caiman crocodilus* | 140 | 160 | 1600 | 0.035 | Y | Y | Y |
| AMNH R45102 | *Crocodylus acutus* | 140 | 160 | 1300 | 0.048 | Y | Y | Y |
| AMNH R4828 | *Crocodylus moreletii* | 140 | 160 | 1125 | 0.100 | Y | Y | Y |
| AMNH R45101 | *Crocodylus moreletii* | 140 | 160 | 1350 | 0.102 | Y | Y | Y |
| AMNH R113078 | *Tomistoma schlegelii* | 165 | 150 | 2150 | 0.135 |  |  | Y |
| AMNH R15163 | *Crocodylus acutus* | 160 | 170 | 1570 | 0.060 | Y | Y | Y |
| AMNH R45119 | *Crocodylus acutus* | 160 | 170 | 2700 | 0.076 | Y | Y | Y |
| AMNH R162581 | *Alligator mississippiensis* | 160 | 170 | 1570 | 0.088 | Y | Y | Y |
| AMNH R116354 | *Osteolaemus tetraspis* | 160 | 170 | 1570 | 0.055 | Y | Y | Y |
| AMNH R81802 | *Gavialis gangeticus* | 140 | 130 | 2320 | 0.088 | Y | Y | Y |
| AMNH R110145 | *Gavialis gangeticus* | 140 | 130 | 1790 | 0.133 | Y |  | Y |
| AMNH R137170 | *Paleosuchus palpebrosus* | 140 | 130 | 1400 | 0.086 | Y |  | Y |
| AMNH R93812 | *Paleosuchus palpebrosus* | 140 | 130 | 1450 | 0.111 | Y |  |  |
| AMNH R23900 | *Alligator sinensis* | 140 | 130 | 1550 | 0.099 | Y | Y | Y |
| AMNH R7124 | *Alligator mississippiensis* | 130 | 130 | 1500 | 0.100 |  |  |  |
| AMNH R101419 | *Melanosuchus niger* | 130 | 130 | 1100 | 0.127 | Y |  | Y |
| AMNH R88316 | *Gavialis gangeticus* | 130 | 130 | 1650 | 0.096 | Y |  | Y |
| AMNH R97325 | *Melanosuchus niger* | 130 | 130 | 1800 | 0.123 | Y | Y | Y |
| AMNH R147051 | *Paleosuchus palpebrosus* | 140 | 160 | 1300 | 0.064 | Y | Y | Y |
| AMNH R137162 | *Paleosuchus palpebrosus* | 140 | 160 | 1600 | 0.063 | Y | Y | Y |
| AMNH R10074 | *Crocodylus cataphractus* | 140 | 160 | 1300 | 0.111 | Y | Y | Y |
| AMNH R88316 | *Gavialis gangeticus* | 140 | 160 | 1650 | 0.096 | Y |  |  |
| AMNH R101422 | *Tomistoma schlegelii* | 140 | 160 | 2600 | 0.104 | Y | Y | Y |
| AMNH R107634 | *Crocodylus cataphractus* | 140 | 160 | 1900 | 0.112 | Y | Y | Y |
| AMNH R160901 | *Osteolaemus tetraspis* | 140 | 160 | 2200 | 0.097 |  | Y | Y |
| AMNH R155461 | *Crocodylus moreletii* | 150 | 160 | 1400 | 0.076 | Y | Y | Y |
| AMNH R77632 | *Crocodylus palustris* | 150 | 160 | 2200 | 0.079 |  |  | Y |
| AMNH R69337 | *Crocodylus johnstoni* | 150 | 160 | 1500 | 0.052 | Y | Y | Y |
| AMNH R71192 | *Crocodylus niloticus* | 150 | 160 | 1650 | 0.088 | Y | Y | Y |
| AMNH R96134 | *Crocodylus palustris* | 150 | 160 | 2200 | 0.096 | Y | Y |  |
| AMNH R127255 | *Crocodylus c.f. niloticus* | 150 | 160 | 2150 | 0.142 |  |  | Y |
| AMNH R44107 | *Crocodylus niloticus* | 140 | 160 | 1600 | 0.055 | Y | Y | Y |
| AMNH R146394 | *Crocodylus niloticus* | 160 | 130 | 1200 | 0.051 |  |  | Y |
| AMNH R173647 | *Tomistoma schlegelii* | 160 | 130 | 1100 | 0.064 |  | Y | Y |
| TMM-M-5490 | *Gavialis gangeticus* | - | - | 971 | 0.228 |  |  | Y |
| YPM HERR-008438 | *Gavialis gangeticus* | 200 | 180 | 1600 | 0.025 x 0.025 x 0.056 | Y | Y |  |
| FMNH-73711 | *Caiman crocodilus* | 120 | 200 | 945 | 0.082 x 0.082 x 0.142 |  |  | Y |
| FMNH-98936 | *Osteolaemus tetraspis* | 180 | 133 | 870 | 0.055 x 0.055 x 0.11 |  |  | Y |
| TMM-M-4980 | *Crocodylus moreletii* | 420 | 1800 | 663 | 0.190 x 0.190 x 0.5 |  |  | Y |
| FMNH-31348 | *Alligator mississippiensis* | 180 | 133 | 690 | 0.028 x 0.028 x 0.062 |  | Y | Y |
| TMM-M-6342 | *Tomistoma schlegelii* | 420 | 1800 | 891 | 0.165 x 0.165 x 0.46 |  | Y | Y |
| TMM-M-6723 | *Alligator mississippiensis* | 180 | 133 | 680 | 0.081 x 0.081 x 0.175 |  |  | Y |
| TMM-M-3529 | *Crocodylus cataphractus* | 420 | 1800 | 818 | 0.165 x 0.165 x 0.46 |  |  | Y |

* Abbreviations: American Museum of Natural History – AMNH; Florida Museum of Natural History - FMNH; Texas Memorial Museum – TMM; Yale Peabody Museum – YPM.

^1^ Rounded to the nearest thousandth place. Resolution has one value listed if the voxel is a cube, and three listed when it is not a cube.

**Table S2: Parameters and results for the cladistic analysis using braincase morphometric data.**

|  | 2D Braincase | | | | | | | |
| --- | --- | --- | --- | --- | --- | --- | --- | --- |
|  | Raw | | | | Residual | | | |
|  | PrCo | PrCo + all | PCA | PCA + all | PrCo | PrCo + all | PCA | PCA + all |
| # morphometric characters | 30 | 30 | 26 | 26 | 30 | 30 | 29 | 29 |
| # taxa for morphometric | 29 | 29 | 29 | 29 | 29 | 29 | 29 | 29 |
| # discrete morphological characters | - | 169 | - | 169 | - | 169 | - | 169 |
| # taxa for discrete morphological | - | 120 | - | 120 | - | 120 | - | 120 |
| # molecular characters | - | 11564 | - | 11564 | - | 11564 | - | 11564 |
| # taxa for molecular | - | 29 | - | 29 | - | 29 | - | 29 |
| Total Characters | 30 | 11763 | 26 | 11759 | 30 | 11763 | 29 | 11762 |
| Outgroup Taxon | *A.miss 6723* | *B. fagesii* | *A.miss 6723* | *B. fagesii* | *A.miss 6723* | *B. fagesii* | *A.miss 6723* | *B. fagesii* |
| # MPTs | 1 | 310 | 1 | 3140 | 1 | 2470 | 3 |  |
| MPT Length | 7.965 | 17546.073 | 5.912 | 17541.530 | 7.493 | 17540.630 |  |  |
| CI | 0.340 |  | 0.211 |  | 0.210 |  | 0.215,0.215.0.212 |  |
| RI | 0.582 |  | 0.225 |  | 0.183 |  | 0.210, 0.209, 0.199 |  |

PrCo: Procrustes coordinates; PCA: principal coordinates analysis; + all: morphometric data plus discrete morphological and molecular characters;#MPT: number of most parsimonious trees before the second round of tree searching; CI: consistency index; RI: retention index; *A. miss* 6723: *Alligator mississippiensis* specimen TMM-M6723; *B. fagesii*: *Bernissartia fagesii*

**Table S3:** **Parameters and results for the cladistic analysis using Eustachian system morphometric data.**

|  | 2D Eustachian System | | | | | | | |
| --- | --- | --- | --- | --- | --- | --- | --- | --- |
|  | Raw | | | | Residual | | | |
|  | PrCo | PrCo + all | RWA | RWA + all | PrCo | PrCo + all | RWA | RWA + all |
| # Morphometric Characters | 30 | 30 | 26 | 26 | 30 | 30 | 30 | 30 |
| # Taxa for Morphometric | 41 | 41 | 41 | 41 | 41 | 41 | 41 | 41 |
| # Discrete Morphological Characters | - | 169 | - | 169 | - | 169 | - | 169 |
| # Taxa for Discrete Morphological | - | 131 | - | 131 | - | 131 | - | 131 |
| # Molecular Characters | - | 11564 | - | 11564 | - | 11564 | - | 11564 |
| # Taxa for Molecular | - | 41 | - | 41 | - | 41 | - | 41 |
| Total Characters | 30 | 11763 | 26 | 11759 | 30 | 11763 | 30 | 11763 |
| Outgroup Taxon | *A.miss 6723* | *B. fagesii* | *A.miss 6723* | *B. fagesii* | *A.miss 6723* | *B. fagesii* | *A.miss 6723* | *B. fagesii* |
| # MPTs | 1 | 2160 | 1 | 1730 | 1 | 1970 | 1 | 980 |
| MPT Length | 22.976 | 17577.010 | 15.494 | 17556.402 | 21.887 | 17563.683 | 15.046 | 17552.587 |
| CI | 0.135 |  | 0.140 |  | 0.161 |  | 0.161 |  |
| RI | 0.241 |  | 0.168 |  | 0.132 |  | 0.146 |  |

PrCo: Procrustes coordinates; RWA: relative warp analysis; + all: morphometric data plus discrete morphological and molecular characters; #MPT: number of most parsimonious trees before the second round of tree searching; CI: consistency index; RI: retention index; *A. miss* 6723: *Alligator mississippiensis* specimen TMM-M6723; *B. fagesii*: *Bernissartia fagesii*

Supplementary Information:

The number of morphometric data reduced from 30 characters to 26 after the principal components analysis and relative warps analysis because the analysis reduces the degrees of freedom so that the last few characters become 1. As all of the specimens had the same value, we deleted those characters from the analysis. The relative warps analysis has more variation because instead of being solid 1’s, those characters became decimals approximating 1. We chose not to remove these because many of the characters were decimals approximating 1 so there would be no objective way to remove them.

**Table S4: Parameters and results for the cladistic analysis of only discrete morphological and molecular characters.**

|  | Discrete morphology and molecular data only |
| --- | --- |
| # Discrete Morphological Characters | 169 |
| # Taxa for Discrete Morphological | 119 |
| # Molecular Characters | 11563 |
| # Taxa for Molecular | 26 |
| Total Characters | 11733 |
| Outgroup Taxon | *B. fagesii* |
| # MPTs | 3160 |
| Tree Length | 17534 |
| CI | 0.570 |
| RI | 0.826 |

#MPT: number of most parsimonious trees before the second round of tree searching; CI: consistency index; RI: retention index; *B. fagesii*: *Bernissartia fagesii*
