# Supplementary material for: An Expanded Combined Evidence Approach to the Gavialis Problem Using Geometric Morphometric Data from Crocodylian Braincases and Eustachian Systems
Source: PLoS One. 2014 Sep 8;9(9):e105793. doi: 10.1371/journal.pone.0105793 (PMC4157744; doi:10.1371/journal.pone.0105793)
Supplement: File S2 — Morphological and molecular matrices used in cladistic analyses. (DOCX) [file pone.0105793.s006.docx]

**Morphological Data**

*Glen Rose Form*

????? ?1??? ????? ????? 00000 00??? 0000? 000?? 0???1 010?0 00??? ???01 0?0?? ????? 0????
?0000 ?0000 00000 00000 ?0000 ?0000 00000 10000 00100 20000 ?110? ???1? ??0?0 00000 0?000 000?? 01??? 00100 0??0

*Bernissartia fagesii*

????? ?0??? 01111 0210? 00?0? 000?? 0000? 10001 0???0 010?0 00??? ????1 0?0?0 0?001 ?1???
?000? 0000? 00030 ?00?? ???10 ????1 ?0000 ?000? ?0100 ?0??0 0000? 0?0?? 0?0?0 010?0 ??00?
?0??? ????? 00?00 000?

*Hylaeochampsa vectiana*

????? ????? ????? ????? ????? ????? ????? ????? ????? ????? ????? ????? ????? ????? 0????
?0??? ????? 0?0?? 000?0 0??00 ?0?00 00000 10010 00?0? 10100 0020? ???1? 0?000 01010 00000 00??? ?1?00 00100 00?0

*Borealosuchus formidabilis*

000?? 00?0? 11001 00101 00000 01000 0001? 000?2 0???0 11020 0000? 000?1 10000 00010 0001?
?0002 0000? 00231 00000 ?0000 10000 00010 10010 00101 00201 ?1000 000?0 0?000 01010 01010 00??? ???00 00110 0000

*Borealosuchus wilsoni*

????? ?0??? ????? ??101 00000 010?? 00?1? 000?2 0???0 1002? ?100? 0?001 10000 02010 00???
?0002 0?0?? ?0231 000?? ???00 1?0?0 ?0010 10010 00101 00201 0100? 00010 0??00 11010 01010 000?0 0???0 0011? 00?0

*Borealosuchus acutidentatus*

????? ????? ????? ????? ????? ??0?? ????? ????? ????? ?002? ????? ????? ?000? ????? 0????
?0002 0?00? ?0231 000?? ???00 ????0 ?0??0 ????? ??1?? 002?1 ?100? 0?0?0 ??0?0 11010 ?1010 00??? ????0 0??1? 00?0

*Borealosuchus sternbergii*

0000? 0000? 11001 0?101 00000 01000 0001? 00??? 0???0 11020 00000 0?000 10000 00010 0000?
?0002 00000 00131 00000 01000 1?0?0 00001 11010 00101 00001 1100? 00010 0?000 00010 01010 00000 ?1?00 00110 0000

*Eothoracosaurus mississippiensis*

????? ?0??? ????? ????1 ??000 ????? ??00? 00??? 0???? 122?? ?3??? ???0? ????0 0?011 ?0???
?0012 000?? 10250 0000? ?0000 ????0 00010 10010 00??? ?00?0 0000? ??01? ??100 00010 ?001? 00??? ????? 00010 0000

*Thoracosaurus neocesariensis*

????? ?0??? ???11 1?1?1 0???? 001?? 0??0? 00??? 0???1 122?? ?3??? ????? 10?00 0?01? 0????
?0012 0000? 10250 00000 ??000 0?0?0 00010 10010 000?0 00000 0010? 00010 ??1?0 00010 00010 00000 00?00 00?10 0000

*Thoracosaurus macrorhynchus*

????? ?0??? 0?111 1?1?1 ????? 00??? 0???? 00??? 0???1 ?22?? ?3??? ???0? 10000 00011 00???
?0012 00?0? 10250 00000 ??000 0?0?0 00010 10010 00011 00000 0010? 00010 0?100 01010 ?0010 000?? ?0??0 00010 0000

*Eosuchus minor*

????? ?0??? 0??11 1???1 ?00?0 ?01?? 0000? 000?? 0???1 122?? 0300? 0000? 10?00 00011 00???
?0012 0000? 1025? 00000 ?0000 ?0000 00010 10010 000?1 00000 0010? ??010 0?10? 10010 0001? 0100? ?1??? 01010 1301

*Eosuchus lerichei*

????? ?0??? ???01 ????1 ????? ??1?? ????? 0???? ????1 122?? ?3??? ????? ????? ????? 0????
?0012 0000? 10250 00000 ?0000 0???0 00010 10010 00001 00000 ?010? 000?0 ??10? 20010 00010
?1??? ????? 0?01? 1301

*Eogavialis africanus*

????? ???1? ????1 1???1 0???? ????? 0???? ????? 0???1 122?? 03??? ??10? 10000 0??11 01???
?0012 000?0 1025? 00000 ?0000 00000 00010 10010 00001 00000 0021? 00010 0?100 10010 00010 100?0 00??0 01010 1000

*Argochampsa krebsi*

????? ?0??? 0111? ????1 ????? 0?1?? ???0? ????? ????1 12??? ?3??? ????? ????? ????? 0????
?0012 ?000? 10250 00000 ??000 ????0 0?010 ?0?10 000?1 00001 0010? ??0?? ???00 11010 00010 0010? ?0??0 ???1? 10??

*Aktiogavialis puertoricensis*

????? ????? ????? ????? ????? ????? ????? ????? ????? ????? ????? ????? ????? ????? ?????????? ????? ????? ????? ????? ????? ????? ????0 ?0??? ????? ??2?? ????? ???00 11010 000?? 0?001 ?0??? 210?? 1???

*Piscogavialis jugaliperforatus*

????? ????? ????? ????? ????? ????? 1???? ????? ????1 1?2?? ?3??? ??000 1???? ???10 ?????
?0012 00100 10250 00000 ?0000 ????0 00010 10010 00000 ?0000 0011? 1?000 10100 20010 00020 0?0?1 ?0?0? 2??1? 100?

*Gryposuchus colombianus*

????? ?0??? 001?? ????1 ??000 ????? ????? ????? 0???1 1223? 03010 0?000 10000 00011 00???
?0012 0000? 1025? 00000 ?0?00 ??0?0 00010 10010 00001 10000 0021? 00010 0?100 11010 00020 00001 00?00 21010 1000

*Ikanogavialis gameroi*

????? ????? ????? ????? ????? ????? ????? ????? ????1 1223? ?3??? ???0? 10?0? 0?0?? ?0???
?0002 0000? 1025? 000?? ???00 ????? 0?01? ??010 ????? 100?0 0021? ????0 ????? 210?0 00020 00??? ????0 21010 100?

*Siquisiquesuchus venezuelensis*

????? ?0??? ????? ????1 ????? ????? ???0? ????? ????1 1223? ?300? 0???? ????? 0?01? ?????
?001? 000?? 1025? 000?? ???00 ????0 00010 ??01? ????? ????0 0021? ??0?? ??1?0 ??010 ?002? 00??? ????0 21010 100?

*Gavialis lewisi*

????? ????? ????? ????1 0???? ????? ????? ????? ????? ?22?? ?3??? ???0? 10000 0??1? 0????
?00?3 0???? ??2?? 0???0 ?0?00 ??0?0 000?0 ?0010 0?0?1 ???00 0021? 00010 0?100 ??0?0 00010 000?? ???00 21010 1000

*Gavialis gangeticus*

0200? 0000? 00111 10111 00000 00110 00000 ?0000 00001 12230 03000 00000 10000 00011 00010 00013 00000 1025? 00000 00000 00000 00010 10010 00001 10000 00210 00010 00100 11010 00010 00000 00000 21010 1000

*Gavialis gangeticus 2*

0200? 0000? 00111 10111 00000 00110 00000 ?0000 00001 12230 03000 00000 10000 00011 00010 00013 00000 1025? 00000 00000 00000 00010 10010 00001 10000 00210 00010 00100 11010 00010 00000 00000 21010 1000

*Boverisuchus vorax*

????? ?0??? 01001 001?1 ?0000 011?? 0100? 10??? 1???1 110?0 00??? ??0?? 10000 01?1? 0????
?2101 0000? 00030 00100 ?0000 ??0?0 00000 10010 00101 00001 1110? ??010 0?010 00010 01010 00??? 01??1 00110 0200

*Boverisuchus magnifrons*

????? ????? ???0? 0???1 ?000? 0???? 0100? 1???1 ????1 11020 00??? ???0? 1???? 0??1? 1????
?21?2 00000 00030 001?? ?0000 ????0 00000 10010 10??1 00001 1110? 0?0?0 0?0?0 10010 ?1010 0???? ????? ???10 0200

*Boverisuchus hengdongensis*

????? ????? ????? ????1 ????? ????? ????? ????? ????1 110?? ?1??? ????? 1???? 0?01? 0????
?2001 0???? ?0130 0???? ??0?0 ????0 ????0 1001? ????? ?0??1 ?100? ????? ??0?0 ?0010 0?010 0???? ?1??? ???10 ?110

*Planocrania datangensis*

????? ????? ????? ????? ????? ????? ????? ????? ????1 1???? ?0??? ????? ????? ????? ?????
?2001 000?? 10030 00000 ??0?0 ????? ??000 ??01? ????? ?0??1 ?110? ????? ??0?0 00010 010?? 0???? ????? ???10 ???0

*Leidyosuchus Canadensis*

????? ?0??? ????1 ????1 00000 11??? 10?0? 11??1 1???0 110?0 0000? 0?01? 10000 00111 01???
?0001 00000 00030 00000 00010 10001 00001 11010 00101 00001 1100? 01010 01001 00010 01010 00000 01?00 00110 0110

*Diplocynodon hantoniensis*

100?? ?1?1? 01000 01001 00001 111?? 1400? 101?2 1???0 11021 010?? ??011 10000 01111 01???
?0012 0?000 ?1130 0?000 ?1010 1?0?0 00000 10110 00101 00001 1100? 11010 0?001 01010 11010 00??0 ?1??0 0011? 0110

*Diplocynodon ratelii*

????? ?0??? ???00 0???1 0?00? 11100 1400? 10??2 1???0 10021 01??? ??011 10000 01111 01???
?0012 00000 01230 00000 01010 ??0?0 00000 11110 00101 00001 1100? ?1010 01000 00010 11010 00000 01?10 00110 0110

*Diplocynodon darwini*

1000? 1001? 01000 0?001 0000? 111?? 1400? 10112 1???0 11020 010?? 0?01? 10000 01111 0100?
?0002 0000? 01030 00000 ?0??0 ??0?0 00000 1111? 00101 000?1 1100? 11010 0?011 01010 11010 00??? ????0 00110 0110

*Baryphracta deponiae*

100?? ?0??? ????0 ????1 ?0??? 1???? 14?0? 10??2 1???? 1?02? ?0??? ??01? 10??0 ??111 0????
?001? ?00?? 01030 000?0 ????0 ????? 0?000 1?110 00?01 000?1 1100? 11010 0?0?? 011?0 ?101? 00??? ????? ???1? ?110

*Stangerochampsa mccabei*

????? 10??? 01001 0?001 00000 11100 1000? 01??1 1???1 11010 100?? ??011 11100 00?11 0????
?0011 0002? 11020 00000 ?0010 ????0 00000 01111 00101 20011 1100? 11020 0?0?1 10010 21010 00??1 ?1??0 00110 0110

*Albertochampsa langstoni*

????? ????? ????? ????? ????? ????? ????? ????? ????? ????? ????? ????? ????? ????? ??????001? 0?0?? 11020 000?0 ??010 ????0 0??00 01111 0010? 200?1 1100? ??0?0 ??0?1 10010 21010 0???? ????0 00110 001?

*Brachychampsa montana*

1010? 1001? 1100? ??001 ??000 11100 ?000? 10311 1???1 11011 01??? ??011 10?00 00111 0100?
?0011 0002? 11010 00000 ?0010 1?0?0 10000 01111 00101 20011 1100? 11020 0?011 10010 11010 20001 01?00 00110 0110

*Brachychampsa sealeyi*

????? ????? ????? ????? ????? ????? ????? 10??? 1???1 1101? ?0??? ????1 1??00 00111 ?1???
?001? 0002? 11010 0000? ??01? ????? ??00? ????? ??10? 20011 0100? ????? ????? 100?? ????? 2???? ????? ???1? ?110

*Alligator sinensis*

1010? 1101? 11001 01001 01101 11100 11000 11211 11101 10000 120?0 01011 20000 01111 01??1 10010 01000 01020 00000 00010 11001 00000 01111 00101 10111 11100 11020 01011 20010 21010 10001 11100 00110 0110

*Alligator mississippiensis*

1010? 1001? 01001 00001 01101 11100 11000 11210 11001 10001 12010 01011 20001 01111 01001 10010 01000 01020 00000 00010 11100 00000 01111 00101 10111 11100 11020 01011 21010 21010 10001 11100 00110 0110

*Alligator mississippiensis 2*

1010? 1001? 01001 00001 01101 11100 11000 11210 11001 10001 12010 01011 20001 01111 01001 10010 01000 01020 00000 00010 11100 00000 01111 00101 10111 11100 11020 01011 21010 21010 10001 11100 00110 0110

*Alligator mississippiensis 3*

1010? 1001? 01001 00001 01101 11100 11000 11210 11001 10001 12010 01011 20001 01111 01001 10010 01000 01020 00000 00010 11100 00000 01111 00101 10111 11100 11020 01011 21010 21010 10001 11100 00110 0110

*Alligator mefferdi*

????? ????? ????? ????? ????? ?1??? ????? 11??? ????1 10000 12010 00011 20001 01111 0100?
?0010 0100? 01020 00000 ?0?10 1?1?0 00000 0?111 00101 10111 1110? 11020 0?011 21010 21010 10??1 ???00 00110 0110

*Alligator thomsoni*

????? ????? ????? ????1 ????? ????? ???0? 1???? ????1 10000 ?2??? ??011 20001 00111 01???
?0010 01000 01020 0090? ?0010 1?1?0 00000 ????1 ????? 10111 1110? 1?020 0101? 21010 21010 1?001 ?11?0 00110 0110

*Alligator olseni*

????? ?0?1? ????1 0???1 ?1001 111?? ?100? 11??? ????1 10100 10??? ??011 20000 00111 01???
?0010 01000 ?1020 0??00 ???10 ??0?0 ?0001 01111 10101 10111 1110? 11020 0?011 20010 21010 10??? ???00 00?1? 0110

*Alligator mcgrewi*

1000? 0001? 01001 0?001 00001 11?10 1??0? 11??? 1???1 11100 10?10 0?011 10000 01111 01???
?0000 0100? 01020 00000 ?0010 1?001 00000 01111 00101 10111 11000 11020 0?011 20010 21010 10?01 ???00 00110 0110

*Alligator prenasalis*

????? ?0?1? ????? 0???1 00001 111?? 1000? 11211 1???1 11110 10??? ??011 10000 01111 0100?
?0000 0100? 01020 00000 ?0010 11001 00000 01111 00101 10111 1100? 11020 0?011 20010 21010 00001 ?1?00 00110 0110

*Ceratosuchus burdoshi*

????? ????? ????? ????? ????? ????? ???0? 1???? ????1 1111? ?0??? ??01? 10??0 0?11? 01???
?0001 0?01? 01020 00000 ???10 ??0?? ??00? ??01? ??1?1 ??0?1 1100? ????? ??0?? ??010 ?111?
????? ????? 0??10 0110

*Hassiacosuchus haupti*

001?? ?0?1? ????? 0???1 ?000? 111?? ???0? ?11?1 1???1 11110 ?0??? ??01? 10??0 ??111 01???
?0001 00??? 010?0 000?? ????0 1?0?? ????0 ????? ????1 100?1 1100? 1?020 0?011 20010 21010 1???? ????0 ???10 0110

*Caimanosuchus brevirostris*

????? ????? ????? ????1 ?000? 1?1?? 1000? 11??1 1???1 11110 10??? ??011 11??? ??111 ?????
?0001 00??? 010?? 000?? ????0 ????? ????? ????? ????? 000?1 1100? ??0?? ??0?? 2001? ?1010 1???? ????? ???1? ?110

*Allognathosuchus polyodon*

????? ????? ????? ????? ????? ????? ????? ????? ????1 11110 10??? ??01? 11??0 0?111 ?1???
?0001 000?? 01020 000?0 ????0 ??0?0 0?00? 0?111 001?1 ?01?1 1100? ????? ??0?? 20010 ?101?
????? ????? ???1? ?11?

*Allognathosuchus wartheni*

????? ?0??? ????? ????? 0000? 111?? 1000? 11??? 1???1 11110 10?10 0?011 11000 00111 01???
?0001 0000? 01020 00000 00010 1?0?0 00000 ?0111 001?1 10111 1100? 11020 0?011 20010 21010 000?1 ?1?00 00110 011?

*Navajosuchus mooki*

????? ?0?1? ????? 0???? 00??? 111?? 1??0? 11111 1???1 11110 10??? ??01? 10??0 0??11 0????
?0001 0001? 01020 000?? ?0?10 ??0?? ?00?0 0?111 00101 10011 1100? ??020 ??011 2?010 21010 00??? ?1??0 0??10 0110

*Wannaganosuchus brachymanus*

????? ?0??? 1?00? 0???1 0000? 11100 1000? 11??? 1???1 11110 ?0??? ??0?? 100?0 0?11? 0????
?0011 0000? ?1020 0??00 ???10 ??0?1 00000 ???1? ?01?1 101?1 1100? ????? ??0?1 2001? ?1010 00??? ????? 0011? 0110

*Procaimanoidea kayi*

????? 10?1? ????? 0???1 0?00? 111?? 10?0? 11212 1???? ?010? 1??10 0?01? 11000 00111 0????
?10?? ?0??? 010?0 000?? ?0?10 ??0?0 00000 00111 00?01 10111 11000 ??020 0?011 20010 21010 10??? ????0 00110 0110

*Procaimanoidea utahensis*

????? ????? ????? ????? ????? ????? ????? ????? ????1 10100 ?00?? 01011 110?? 00111 01???
?1011 0100? 01020 ?0000 ??010 ????0 00000 0?111 00101 101?1 1100? 1?020 0?011 20010 ?1010 00??? ????0 00110 0110

*Arambourgia gaudryi*

????? ????? ????? ????? ????? ????? ????? ????? ????1 1010? ?0??? ??01? 100?0 ??111 0????
?1001 ?10?? 01020 000?0 ???10 1000? ?000? ???11 ?0??1 101?1 1100? 1102? 0?0?1 210?0 21010 0???? ????? 0011? 0110

*Deinosuchus riograndensis*

????? ?0??? ????? ????1 ????? ????? ????? 00??? 1???0 110?? ?001? 0?11? 10??0 ??11? 0????
?00?? 1???? ??0?0 000?? ?0??0 11?00 ?0??? ????? ????? ?0001 1120? 01221 0?111 00010 01?10 00??0 00?0? ???1? ?110

*Purussaurus neivensis*

101?? 00?1? 00001 0?0?1 ????0 11??? ???0? 11??? 1???1 ?00?? 1?101 0?111 20110 00110 01???
?0011 0001? 01020 00000 ?0010 1?0?0 ?0000 01111 01211 11111 1110? 11020 1?011 2011? 21010 20001 01??0 0??10 2110

*Orthogenysuchus olseni*

????? ????? ????? ????? ????? ????? ????? ????? ????? ?00?? ????? ????? ????? ????? 0????
?0012 1?01? ?10?0 ?0??? ????0 ????0 1?000 ????? ????? ????? 1110? ????? ??0?? ??11? ?101?
?0??? ????? ????? ?110

*Mourasuchus atopus*

10??? 00?1? 00?01 0?001 ?10?0 11??? 1300? 11??? 1???1 102?1 12??? ??011 10?10 00110 00???
?0012 1000? 11040 00100 ?0010 1?0?0 10000 01111 012?1 011?1 1110? 110?0 0???? 2111? ?111? 30??? ?1??? 00110 ?110

*Eocaiman cavernensis*

????? ????? ????? ????? ????? ????? ????? ????? ????1 110?? ?2??? ??11? 1???? ????? ?????
?00?? ????? ??0?? ????? ???1? ??0?1 ??000 0???? ???0? ????1 ???0? ????0 ????? ??0?? ???1? 3???? ????? 0??1? 2???

*Caiman yacare*

1011? 1001? 10001 00001 01011 11100 11000 11122 11101 10021 12101 01110 10110 20110 01011 10011 00000 01120 00000 00010 11001 00000 01111 01211 11011 11100 11020 11011 20111 21010 30001 01100 00110 2110

*Caiman crocodilus*

1011? 1001? 10001 00001 01011 11100 11000 11122 11101 10021 12101 01110 10110 20110 01011 10011 00000 01120 00000 00010 11001 00000 01111 01211 10011 11100 11020 11011 20111 21010 30001 01100 00110 2110

*Caiman latirostris*

1011? 0001? 10001 00001 010?1 11100 11000 11122 12101 10021 12101 0111? 10110 20110 0???1 10011 00000 01020 01000 00010 11001 00000 01111 01211 10011 11100 11020 11011 21111 21010 30001 01100 00110 2110

*Caiman lutescens*

????? ????? ????? ????? ????? ????? ????? ????? ????? ?0??? ????? ????? ????? ????? ??????0011 0000? 01020 01000 ?0010 ????1 00000 01111 01211 200?1 ?110? ????0 ????? ??1?? ??0??
????? ????? ????? ?110

*Melanosuchus fisheri*

????? ?0??? ????? 0???? ????? ????? ????? ????? ????? ??02? ????? ??1?1 1011? ???10 ?1???
?001? 0000? ?1020 01010 ???10 ??0?? ?0??? ?11?? ??2?1 1???1 1110? ????? ??0?? ??11? ?1010
 ????? ????0 0??1? 21?0

*Melanosuchus niger*

1011? 1001? 1?001 00001 01011 11100 11000 11122 12101 10021 12101 01111 10110 20110 01??1 10011 00000 01020 01010 00010 11001 00000 01111 01211 10011 11100 11020 11011 21111 21010 30001 01100 00110 2110

*Paleosuchus trigonatus*

1001? 1111? 01001 01001 00011 11121 13000 11132 11201 10021 22211 11111 10110 00110 01011 11011 00100 01020 00000 00010 11000 10001 01111 01111 00011 11101 11020 11011 2111? 21010 20001 01100 00110 2110

*Paleosuchus palpebrosus*

1001? 1111? 01001 01011 00011 11121 13000 11132 11201 10021 22211 1?11? 10110 00110 0?011 11011 00100 01020 00000 00010 11000 10001 01111 01111 00011 11101 11020 11011 2111? 21010 20001 01100 00110 2110

*Paleosuchus palpebrosus 2*

1001? 1111? 01001 01011 00011 11121 13000 11132 11201 10021 22211 1?11? 10110 00110 0?011 11011 00100 01020 00000 00010 11000 10001 01111 01111 00011 11101 11020 11011 2111? 21010 20001 01100 00110 2110

*Paleosuchus palpebrosus 3*

1001? 1111? 01001 01011 00011 11121 13000 11132 11201 10021 22211 1?11? 10110 00110 0?011 11011 00100 01020 00000 00010 11000 10001 01111 01111 00011 11101 11020 11011 2111? 21010 20001 01100 00110 2110

*Paleosuchus palpebrosus 4*

1001? 1111? 01001 01011 00011 11121 13000 11132 11201 10021 22211 1?11? 10110 00110 0?011 11011 00100 01020 00000 00010 11000 10001 01111 01111 00011 11101 11020 11011 2111? 21010 20001 01100 00110 2110

*Crocodylus cataphractus*

10?0? 1001? 00001 00001 11001 11120 12000 11110 11011 11041 01010 00100 10001 11010 1?100 10012 00001 00210 00000 01000 11010 00010 11010 00101 00001 11100 00101 11001 20010 01010 00111 01011 10010 0300

*Crocodylus cataphractus 2*

10?0? 1001? 00001 00001 11001 11120 12000 11110 11011 11041 01010 00100 10001 11010 1?100 10012 00001 00210 00000 01000 11010 00010 11010 00101 00001 11100 00101 11001 20010 01010 00111 01011 10010 0300

*Crocodylus niloticus*

1010? 0001? 10101 00011 11001 11120 12000 11120 11011 10021 01010 00101 10001 11010 11100 10011 00001 00210 00000 11000 11010 01000 11010 00101 10001 11100 00101 11001 20010 01010 00111 01011 10011 0300

*Crocodylus niloticus 2*

1010? 0001? 10101 00011 11001 11120 12000 11120 11011 10021 01010 00101 10001 11010 11100 10011 00001 00210 00000 11000 11010 01000 11010 00101 10001 11100 00101 11001 20010 01010 00111 01011 10011 0300

*Crocodylus porosus*

1110? 0001? 00101 01011 11000 11120 12000 11120 11011 10021 01010 00101 10001 11010 11100 10011 00001 00210 00100 11000 11010 01000 11010 00101 00001 11100 00101 11001 20010 01010 00111 01011 10011 0300

*Crocodylus rhombifer*

0010? 0001? 10101 00011 11001 11120 11000 11120 11011 10021 01010 00101 10001 11010 11100 10011 00001 00210 10000 11000 11010 01000 11010 00101 10001 11100 00101 11001 20010 01110 00111 01011 10011 0300

*Crocodylus acutus*

0010? 0001? 10101 10011 11001 11120 12000 11020 11011 10021 01010 00101 10001 11010 11100 10011 00001 00210 10000 11000 11010 01000 11010 00101 10001 11100 00101 11001 20010 01010 00111 01011 10011 0300

*Crocodylus acutus 2*

0010? 0001? 10101 10011 11001 11120 12000 11020 11011 10021 01010 00101 10001 11010 11100 10011 00001 00210 10000 11000 11010 01000 11010 00101 10001 11100 00101 11001 20010 01010 00111 01011 10011 0300

*Crocodylus acutus 3*

0010? 0001? 10101 10011 11001 11120 12000 11020 11011 10021 01010 00101 10001 11010 11100 10011 00001 00210 10000 11000 11010 01000 11010 00101 10001 11100 00101 11001 20010 01010 00111 01011 10011 0300

*Crocodylus palustris*

1010? 0001? 00101 00111 11001 11121 12100 11120 11011 10021 01010 00101 10001 11010 11100 10011 00001 00210 00100 11000 11010 01000 11010 00101 00001 11100 00101 11001 20010 01010 00111 01011 10011 0300

*Crocodylus siamensis*

1110? 0001? 11101 00001 11001 01120 12000 11120 11011 10021 01010 00101 10001 11010 11100 10011 00001 00210 00100 11000 11010 01000 11010 00101 00001 11100 00101 11001 20010 01110 00111 01011 10011 0300

*Crocodylus intermedius*

0010? 0001? 10101 00011 11001 11120 12000 11120 11011 11021 01010 00101 10001 11010 11100 10011 00001 00210 10000 11000 11010 01000 11010 00101 00001 11100 00101 11001 20010 01010 00111 01011 10011 0300

*Crocodylus johnstoni*

1110? 0001? 00101 01011 11000 11120 12000 11120 11011 1?021 01010 00101 10001 11010 11100 10011 00001 00210 00100 11000 11010 01010 11010 00101 00001 11100 00101 11001 20010 01010 00111 01011 10011 0300

Crocodylus mindorensis

1110? 0001? 00101 01011 11000 11120 12000 11120 11011 10021 01010 00101 10001 11010 11100 10011 00001 00210 00100 11000 11010 01000 11010 00101 00001 11100 00101 11001 20010 01010 00111 01011 10011 0300

*Crocodylus novaeguineae*

1110? 0001? 00101 01011 11000 11120 12000 11120 11011 10021 01010 00101 10001 11010 11100 10011 00001 00210 00100 11000 11010 01000 11010 00101 00001 11100 00101 01001 20010 01010 00110 01011 10011 0300

*Crocodylus moreletii*

0010? 0001? 10101 00011 11001 11120 12000 11120 11011 10021 01010 00101 10001 11010 11100 10011 00001 00210 10000 11000 11010 01000 11010 00101 10001 11100 00101 11001 20010 01010 00111 01011 10011 0300

*Crocodylus moreletii 2*

0010? 0001? 10101 00011 11001 11120 12000 11120 11011 10021 01010 00101 10001 11010 11100 10011 00001 00210 10000 11000 11010 01000 11010 00101 10001 11100 00101 11001 20010 01010 00111 01011 10011 0300

*Crocodylus moreletii 3*

0010? 0001? 10101 00011 11001 11120 12000 11120 11011 10021 01010 00101 10001 11010 11100 10011 00001 00210 10000 11000 11010 01000 11010 00101 10001 11100 00101 11001 20010 01010 00111 01011 10011 0300

*Crocodylus palaeindicus*

????? ?0??? ????? ????? 1???? ????? 1???? ????? ????1 10021 0101? 00101 10001 1?01? 11???
?0011 0000? 00210 00000 ?1000 11010 01000 01010 0?101 00001 1110? 00101 1?001 20010 01010 10011 ?1?11 10011 0300

*Euthecodon arambourgii*

????? ????? ????? ????? ????? ????? ????? ????? ????? ????? ?0??? ????? ????? ????? 1????
?0002 0000? 10250 00100 ?1000 11010 00000 ??0?? ??0?? ?0001 1110? ?1101 1?001 21010 01011 00??? ?1??1 ?0?10 030?

*Osteolaemus tetraspis*

??1?? 0001? 00101 01001 11001 11120 11100 11111 11011 10021 01010 00101 10000 11010 11100 11010 00101 00210 00100 01000 11011 00101 11010 10101 00001 11101 00101 01001 21110 01011 00111 01011 10110 0300

*Osteolaemus tetraspis 2*

??1?? 0001? 00101 01001 11001 11120 11100 11111 11011 10021 01010 00101 10000 11010 11100 11010 00101 00210 00100 01000 11011 00101 11010 10101 00001 11101 00101 01001 21110 01011 00111 01011 10110 0300

*Osteolaemus osborni*

??1?? 0001? 00101 01001 11001 11120 11100 11111 11011 10021 01010 00001 10000 11010 11100 11011 00101 00210 00100 01000 11011 00100 10010 10101 00001 11101 00101 01001 21110 01011 00111 01011 10110 0300

*Voay robustus*

????? ?0??? ????? 0???1 1???? ?11?? 1110? ????? 1???1 11021 01010 00001 10001 11110 11???
?0011 0000? 00210 00100 01000 11010 00101 11010 10101 00001 1110? 00101 11001 20110 01111 10?11 01?11 10010 0300

*Rimasuchus lloydi*

????? ????? ????? ????? ????? ????? ????? ????? ????? ????? ?1??? ????? ????? ????? 1????
?0011 0000? 00210 00100 ?1000 11010 0000? ???10 ??1?? 100?1 1110? 00101 1?001 20010 01011 00111 ?1??1 10?10 030?

*Crocodylus pigotti*

????? ????? 00?01 0?001 1??01 111?? ????? 10??? 0???1 1102? ?1??? ????? 1???1 11??? 1????
?0001 0100? 00210 00100 01000 ????0 01010 11010 10001 ?0?01 1110? ??1?1 ??001 21010 01011 00??1 ?1??1 00?10 0300

*Crocodylus megarhinus*

????? ?0??? ????? ???01 ????? ????? ????? ????? ????1 11021 01??? ??001 10000 ??010 11???
?0011 0000? 00230 00000 01?00 ??0?0 00000 11010 00101 20001 1110? 002?1 1?001 2?010 01010
?0110 0???1 10010 0300

*Australosuchus clarkae*

????? ?0??? ????1 ??0?1 ????? 11??? ???0? 10??? 1???1 110?1 01??? ??001 10000 11010 11???
?0011 00001 10210 00000 01000 1?0?0 00000 ????? ??101 00001 1110? 00201 1?011 20010 01010 0011? ?1??1 ?0010 010?

*Kambara implexidens*

????? ?0??? ????? ????1 ????? 11??? 1100? 10??? 1???1 11021 01??? ??001 10000 11010 11???
?0011 00001 10210 00000 01000 1?010 00000 10010 00101 00001 1110? 00201 1?001 20010 01010 00111 01?11 10010 0100

*Trilophosuchus rackhami*

????? ????? ????? ????? ????? ????? ????? ????? ????? ????? ????? ????? ????? ????? ??????0??? ????? ??2?0 ??0?0 ?100? ??0?1 ?0000 01010 ???01 ?0?01 1110? ?1201 11011 21010 00010 20111 01?11 ?0?10 010?

*Tomistoma schlegelii*

0210? 0001? 00101 00011 10001 11110 11000 10130 11011 1?2?1 04000 00100 10000 00010 10000 10012 00001 10210 00001 01000 11010 00100 10010 00101 10001 11100 00011 01001 21010 01010 00110 01011 10010 0300

*Tomistoma schlegelii 2*

0210? 0001? 00101 00011 10001 11110 11000 10130 11011 1?2?1 04000 00100 10000 00010 10000 10012 00001 10210 00001 01000 11010 00100 10010 00101 10001 11100 00011 01001 21010 01010 00110 01011 10010 0300

*Tomistoma schlegelii 3*

0210? 0001? 00101 00011 10001 11110 11000 10130 11011 1?2?1 04000 00100 10000 00010 10000 10012 00001 10210 00001 01000 11010 00100 10010 00101 10001 11100 00011 01001 21010 01010 00110 01011 10010 0300

*Tomistoma lusitanica*

????? ?0??? ????? ????1 ????? ????? ????? 10??? 1???1 ??2?? ?4??? ??00? 10000 ??010 1????
?0012 0000? 1021? 00001 ?1000 110?0 00010 10010 00101 00001 1110? 0001? 1?101 21010 01010 00110 01??1 10010 0300

*Paratomistoma courtii*

????? ????? ????? ????? ????? ????? ????? ????? ????? ????? ????? ??0?? 10?00 00010 10???
?00?? ????? ??2?? 0?0?? ????0 1?0?? ????? ????? ????? 000?1 ??1?? ???1? ??1?? 20010 00010 00?10 00?10 ?0?10 0???

*Thecachampsa antiquus*

0200? 000?? 00101 0?001 10101 11??? 11?0? 00??? 1???1 1?2?? ?4?10 0???? 10000 31?10 1?0??
?0012 00001 10210 00000 ?1000 ??0?0 00010 1?010 00101 10000 1110? ??011 ??101 20010 01010
10??0 0???1 10010 03?2

*Thecachampsa americanus*

????? ????? ????1 ????1 ?000? 111?? 1100? 00??? 1???1 1?2?? ?4??? ??000 10?00 31?10 10???
?0012 00001 10210 00000 ?1000 ????0 00010 00010 00??? 100?0 1110? 000?1 0?101 20010 01010 1???0 ????1 10010 03?2

*Gavialosuchus eggenbergensis*

????? ????? ????? ????1 ????? ????? ????? ????? ????? ????? ????? ????? ????? ????? ??????0012 0000? 10240 00000 ??000 ????0 0?010 10010 00?01 200?0 ?110? 0???? ??1?1 21010 ?1010 0???? ????1 ???10 0300

*Tomistoma cairense*

????? ????? ????? ????? ????? ????? ????? ????? ????1 ??2?? ?4??? ????? 10000 01?10 10???
?00?2 0???? ?025? 0???0 ????0 ??0?0 00010 10010 0010? 00??0 1110? ??01? ??10? 20010 01010 001?? 0???1 ?001? 0300

*Kentisuchus spenceri*

????? ?0??? ????? ????? ????? ????? ????? ????? ????? 1???? ?4??? ??0?? 1000? 1101? 11???
?0011 0000? 10210 00000 ???00 ??0?0 10010 10010 0?101 100?1 1110? ????? 0?0?? 20010 ?1010 0???? ???11 ?0?10 03?0

Tomistoma petrolica

????? ????? ????? ????? ????? ?1??? ????? ????? ????? ????? 04??? ???00 10?0? 01?1? 10???
?00?? ????? ??2?0 0?0?0 ???00 1???0 ?0010 1001? ????? 1?0?0 ?110? 0?2?0 ??0?? 200?0 00010 0???? ????? ???1? ?3??

*Dollosuchoides densmorei*

001?? ?0??? 11101 0?001 ?0?00 111?? 1?00? ????? ????1 1?0?? ?4??? ??000 100?0 ??010 1????
?0012 00?0? 10210 00000 ??000 ????0 1?010 10010 00101 100?0 1110? ????? ??1?? 20010 ?1010 001?? ????? 0?010 030?

*Brachyuranochampsa eversolei*

????? ????? ????? ????? ????? ????? ????? ????? ????? ????? ????? ????? ????? ????? ?????
?0011 ?0??? 10210 00000 ??000 ??0?0 ?0??0 11010 00101 000?1 1100? ??0?0 0?0?1 21010 ?1010 00??? ????1 00010 0300

*Belgian crocodyloid*

???0? ?00?? 11000 01001 10000 111?? 1100? 10??? 1???1 11021 01??? ??001 10000 00010 11???
?0011 0000? 10110 00000 ?1100 1?0?0 00100 1?010 00101 ?0001 1100? 01020 0?001 20010 01010 00110 01?11 00010 0300

*Crocodylus acer*

????? ????? ????? ????? ????? ????? ????? ????? ????? ????? ????? ????? ????? ????? ??????0011 0000? 10210 00000 ??000 1???0 00100 ?1010 00101 00001 1100? 000?0 1?00? 20010 01010 001?? 0??11 00010 0300

*Crocodylus affinis*

0010? 1001? 10001 00001 10000 11100 1100? 10??? 1???1 11021 01010 00001 10000 00010 1100?
?0011 00001 00110 00000 ?0100 ??0?0 00100 11010 00101 00001 1100? 0?010 ??001 20010 01010 001?? 0???1 00010 0300

*Asiatosuchus granger*

????? ????? ????? ????? ????? ????? ????? ????? ????1 ??0?1 01??? ???0? 1000? ???10 1????
?0??? 0???? ??1?? 0???? ??1?? ????? ????? ????? ????? ?00?? ?10?? ????? ????? 20???
??0?? ????? ????? ????? ????

*Asiatosuchus germanicus*

001?? ?0?1? 00101 0?011 ?000? 111?? 1??0? ????? 1???1 11020 00??? ??001 10000 ??010 1?00?
?0001 0000? 00010 0000? ??100 ??0?0 0010? ??010 00101 000?1 1100? ??010 0?001 11010 01010 00??? ????1 00?10 0300

*Prodiplocynodon langi*

????? ????? ????? ????? ????? ????? ????? ????? ????? ????? ????? ????? ????? ????? ??????0011 0000? ?0030 00000 ??100 1??00 00100 11?10 00101 ????1 1100? ??0?0 ??0?1 1?010 ?1010 001?? 01?01 00110 0300

**Molecular Data**

*Crocodylus porosus*

------------ACAAATCTAAATTTATTTGACCAATTCTTAGTCCCCCAACTACTTGGCATACCACTACTAATCCCAGCCATGTTATTAACAACAGTACTAATCTATAACCCGCAAGATCGCTGACTATCAAACCCTTTAACAACCCTACAGTCCTGACTAATTGCAAAAGCCACCAAACAGATCATAACCCCAGTAAATAAGCCAGGACATAAGTGATCATTAATACTGATCTCACTACTAACAATGCTTATTCTCAACAACCTTTTAGGCCTTCTCCCATACACGTTTACACCAACAACTCAGCTGTCTATAAATATAGCCCTGGCCCTCCCACTGTGGCTAGCAACAGTACTAATTGGCCTACGAAACAAGCCAACCTCTTCACTAGCCCACCTCCTACCAGAAGGGACCCCAACACCCCTAATCCCAATCCTAATCCTAATCGAAACAATTAGCCTACTCATCCGACCAATCGCACTTGCCGTACGACTCACAGCCAATCTAACCGCAGGACATCTTCTATTACACCTAATCTCCACTGCGGCACTTAGCCTAATGACAACTTCCACGCTACTTGCCGGACTAACCCTAATCATTCTAACCCTATTAATGCTCCTAGAAATTGCAGTAGCAATAATTCAAGCATACGTGTTTACCCTACTACTCTCACTATACCTGCAAGAAAACGTATAAATGCCCCAATTAAACCCCGAACCTTGATTAATAATCTTATCCATTACATGGCTAGTACTCATTACTAGTTTACAGCCAAAAATTGCCTCTCTAAAGTTCATAAATAGCCCAAGCAGCCCCGCCCAAAAAACCACT-AAAACATGACCCTGACCACAAATCTAA---------------------ATGGCCCACCAACTACGAAAATCCCACCCACTCTTAAAACTAGTAAACCACTCTCTGATTGACTTGCCCACACCATCCAACATCTCCTACTGATGAAACTTTGGATCACTTCTCGGATTCACTCTATTAATCCAATTAGCATCGGGCATCCTACTAATAATGCACTTCCTAGCAGACGACTCTTTAGCTTTTATGTCCGTCGCCTACACTTCACGAGAAGTCTGATATGGTTGACTAATCCGAAGCCTCCATGCAAACGGAGCTTCCCTATTCTTCCTATGCACATTCCTCCACATCGGACGTGGAGTATACTACGGATCTTACCTAAATGAAAATACATGAAACATCGGAGTATTACTGCTACTACTACTGATAGCAACTGCCTTCATGGGCTACGTCCTACCATGAGGGCAGATGTCGTTCTGAGGGGCAACCGTGATCACCAACCTAATATCAGCCATCCCCTACGTAGGAGACTCAATTGTCACCTGAATTTGAGGAGGGCCATCCGTCAATAGCGCAACCTTAACACGATTTACTACCCTGCACTTCCTGCTCCCATTTATCCTCATAGCCACCATCCTCACACACCTCATCTTCCTCCACGAACGCGGATCATTCAACCCACTAGGATTAATCTCTAACGCCGACAAAATTCCATTCCACCCATACTTCTCAGCAAAAGACGCCATAGGCATGGCCCTGGCCACCGTCCTATTAATAACCCTTACATTCTACTTCCCAAACCTATTAGGAGACCCAGAAAACTTTACCCCTGCCGACCCCATAAAAACACCAGACCACATTAAACCAGAATGATATTTCCTATTCGCCTACACAATCTTACGATCTATTCCAAACAAACTCATAGGAGTCATTGCCATATTCGCATCCATCCTAGTATTACTACTCCTACCTGCACTACACACATCAAAACGACAATCGATAAGCCTACGTCCCCTATCCCAACTTTTATTCTGAACCCTAATCGCAGACTTCTTTATCCTCACATGAATCGGGGGCCAGCCAGTACAAAACCCATACACCTTAATCGGACAAACAGCCTCCTTCATCTACTTCTTCACCATCCTTATCTTATTACCACTGGCCGGAATAATTGAAAACCTACTAATCAAACCCCTTCGGTACCGGCCCT----------GTGAACATTAATCGTTGACTATTTTCCACTAACCACAAAGATATCGGCACCTTATATTTTATTTTCGGCGCCTGAGCTGGAATAGTAGGCACAGCCATAAGCCTATTAATCCGGACAGAGCTCAGCCAGCCTGGTCCATTCATAGGGGATGACCAAATTTATAATGTTATTGTCACAGCACATGCCTTTATCATAATTTTCTTTATAGTTATACCGATCATGATCGGCGGATTTGGAAATTGGCTACTCCCACTAATAATTGGAGCACCCGACATAGCATTTCCACGCATAAACAACATAAGCTTCTGACTGCTACCCCCATCATTTACCCTACTTCTCTCTTCAGCCTTTATTGAAACTGGAGCTGGCACCGGATGAACAGTCTACCCACCCCTAGCTGGAAACCTAGCCCACGCCGGACCATCAGTAGACTTGACCATCTTCTCCCTCCACCTTGCTGGAGTATCATCCATCCTTGGAGCAATTAACTTTATTACCACGGCCATTAATATGAAACCCCCAGCAATATCACAACAACAGACGCCTCTTTTCGTATGATCTATTCTAGTTACAGCCGTTCTCCTACTGCTCTCACTACCAGTCCTAGCTGCAGGAATTACCATATTACTCACTGATCGAAACCTGAACACCACCTTCTTTGACCCCGCAGGAGGAGGAGACCCAATCCTATACCAACACCTTTTCTGATTCTTCGGCCACCCAGAAGTATACATCCTCATCCTACCAGGGTTTGGAATAATCTCCCATGTAATCACCTTCTACTCAAGTAAAAAAGAACCATTTGGCTACATAGGAATAGTCTGAGCCATGATATCAATCGGCTTTCTCGGATTCATCGTCTGAGCCCACCACATATTTACAGTAGGAATAGACGTTGACACCCGAGCATACTTCACATCCGCCACAATAATTATCGCCATCCCCACCGGCGTAAAAGTGTTCAGCTGATTAGCCACTATTTACGGAGGAGTAGTGAAATGACAAGCCCCCATGCTCTGAGCACTCGGCTTCATTTTCTTATTCACAGTCGGAGGACTAACAGGAATTGTACTAGCTAACTCATCACTAGACATTATTCTCCACGATACCTACTACGTAGTAGCCCACTTCCACTATGTACTATCTATGGGGGCAGTATTCGCCATCATAAGCGGATTCACTCACT-GGTTCCCACTA-TTTACAGGATTTACCCTCCACCACACATGAACAAAAA-TCCAATTCATAATCATATTCACGGGTGTAAACCTAACCTTCTTCCCACAACACTTCCTGGGCCTGTCAGGGATACCACGACGATATTCCGACTACCCAGATGCATATGCCTTCTGAAATATAATCTCCTCAATCGGATCATTAGTTTCCATAGTATCAGTCGTCCTACTCACATTTATTGTATGAGAGGCATTTTCATCAAAACGAAAAGTCCAAGTGCCTGAAATAGCAAGTACAAACGTAGAATGACTAAACAACTGCCCACCGTCATACCACACCTACGAAGAGCCAGTCTTTGTTCAAGTACGAAAAAAACTAACGTAA------------------------------------ATGGCAAACCCAATACACCTAGGACTCCAAGATGCAATATCCCCGCTAATAGAAGAACTACTCTATTTTCATGACCACACACTAATAATTATTTTTTTAATCAGCATATTTGTACTATACACAATCTCAGTTTTATTACTAACAAGCCTATACCACACAAATGCAACAGATGTACAAGAAATAGAGATAATCTGAACCATTCTGCCAGCCCTAATCCTAATTACAATCGCCCTTCCATCTCTACGCACGCTATACCTTATAGACGAAACCACCAACCCCTGCCTAACCATTAAAGTTATCGGGCATCAATGGTATTGAACATATGAATATACAGACTTTTCCCAGCTGGAATTCGACTCTTACATGCTACCAACACAAGACCTGCCTCAAGGTCACTTCCGCCTTTTAGAAGTAGACCACCGCATGATTGTTCCAACAAGCTCAAGCACCCGAACATTAATCACAGCTGAAGACGTCCTACACTCATGAGCAGTACCATCCCTAGGGATCAAAATAGACGCAGTACCTGGACGACTAAACCAAACCTCACTAACATCCCCCAATCCTGGGGTATTCTATGG-CCAATGTTCTGAAATCTGCGGAGCAAACCATAGTTTTATGCCTATTGTCGTAGAAGCTGTCCCTATACAGCACTTCCAAAACTGATTAAAAACAAACTCAT----AAATGACCCACCAAACACACCTATTCCACATAGTCAACCCAAGCCCCTGGCCAATCATGGGGGCTATGGCTGCCATAATATTAACAGTCGGGCTGGTCCTATGATTCCACTGTAATTTAAACCTAATTTTACTTCTAGGACTAACCTCCACATTACTAATTATATTTCAGTGGTGACGAGATATTGTCCGAGAAAGCACCTATCTAGGCCACCATACCCCTCCAGTCCAAAAAGGACTACGATATGGCATAATCCTATTCATCACCTCAGAGGTCTTCTTTTTCCTTGGGTTCTTCTGAGCGTTCTATCACTCAAGCTTAGCACCAACCCCAGAACTAGGAGGACAATGACCACCAACGGGAATTACCACACTAGACCCATTCGAAGTCCCACTTCTCAACACAGCAGTACTACTAGCCTCAGGAGTTACAGTAACATGAGCCCATCACAGCCTAATAGAAGCCAACCGAGCATCTGCCATCCACGCCTTAATCCTCACGATTATTCTAGGGCTATACTTCACTGCCCTTCAGGCAATAGAGTACTACGAAGCCCCATTCACCATCGCAGACAGCAGCTATGGGTCAACCTTCTTTGTTGCCACAGGCTTTCACGGCCTACACGTCATTATTGGATCAACATTCCTAATAACCTGCCTCTATCGACAAACCATACACCACTTCACCTCGAACCACCATTTCGGTTTCGAAGCTGCCGCTTGATACTGACATTTCGTAGACGTAGTCTGACTGTTCCTATACATCTCAATCTATTGATGAGGCTCCTATAAGCTTTTTAACAGCCACACCCATTCTTATCTATATCGTCTCAGTCCTAATCGCAGTCGCATTCCTGACAGGGCTAGAGCGAAAAATCATTGGCTACATGCAACTACGTAAAGGCCCCAATATTGTGGGCCCTTTCGGGCTGCTACAACCATTTGCTGATGGCCTCAAGCTTATTATTAAAGAACTAACATTGCCCCTACTCGCCACCCCTGCTTTATTTATTCTGTCCCCCACAGTCGCCATTATTTTATCCCTAATCATATGAACCCCCCTACCCGTACCATTTTCTATCGCCAATCTAAACCTTGGCATATTATTTTTATTAGCCATATCCAGCTTAGCAGTTTACTCGCTACTATGGTCCGGGTGAGCATCAAACTCTAAATACGCCCTAATAGGCGCCCTACGAGCAGTAGCCCAAACCATTTCCTATGAAGTCACATTAGCCATCATTGTTTTATCTGTTGTCCTGCTTAGCGGCGGATTTTCACTTCATGCACTGGCTATTACCCAAGAACCCGCCTACCTGGCACTAACCACATGACCACTACTAATAATATGATACACCTCAACACTAGCAGAGACAAACCGCGCCCCGTTTGATCTTACAGAAGGTGAGTCAGAACTAGTATCTGGATTCAACGTTGAATACAGCGCAGGGCTGTTCACACTATTCTTCCTGGCCGAGTACGCCAACATTCTACTAATAAATACTTTAACCACCATCCTATTCCTAAACACATCAACAAACTTGCCAACACAAACACTATTCACCACCACCCTAATAAGCAAATCAATTCTACTAACCATCGGATTCCTATGAATCCGAGCATCATACCCGCGATTCCGGTATGATCAATTAATACACCTACTGTGAAAAAACTTCTTGCCAGCCACGCTGGCAATCTGCCTGTGACACTCGTCGCTCCCGATGTCAACACTAGGCCTTCCAGTAGCAA---GGATGCCCATCTTCCAACCAATCATCTTAACCACACTAACCATTACAACACTCATTTTCTTATCATCCACCCACCTGGTACTCATGTGAGTAGCACTAGAACTTAACACACTAGTGGTCCTACCATTGATTGCCAATAAATCGCACCCACGATCCATCGAAGCCTCCACAAAGTACTTCCTCACACAAGCCGCTGCTTCTGCCTTAATCATCTTCTCATGGACCTTAAACTATATCACAACTGGGGGCGGCCAAATTACAGAAGTAACAAACCAAGTCCTCACAACTATTATAACCCTGGCCCTCTTTATCAAAATTGGACTGGTGCCATTCCACTTCTGAGTACCTGAGACCATTCAAGGAATAGCCCCAACCGCCTCCATCTTCCTACTTACCTGACAAAAACTAGGCCCACTAATCATACTATACCTAATAAGCCCACTAATTAATTTCGAGGTTCTCTCTGCGGTGTCTATCCTCTCCGCCACAGTCGCCGGCTGACTTGGACTTAACCAAACCCAAATCCGGAAACTAGTAGCATTCTCCTCAATTGCCCAAATATCCTGAACACTGGTGATTATTAAATACGCACCATCACTTACAATCTTAGCCTTCTACCTATATTCAATCACAATCTCCGCTACACTTCTCACGTTAGAAAAACTATCAACAACATCCATTAACAACCTCCTACTTTCATTCCAAAAAGCCCCAGTTACTTCCTTACTGCTAACAATCTCCCTATTATCATTATCAGGCCTGCCCCCACTGGCCGGCTTCCTTCCAAAATGATTAACAATTGACCAGCTAGTGGCAGAAGGAGCAATTTGAGTCGCATTCACAATACTCATAGCCTCCCTTCTAAGCCTGTTCTTTTACCTACGACTATGATACAACTCCGCATCCACCCTTCCTCCTAACACTATTAATACCCAACGCCTATGACGCAAACCGACTCAACAAACTAACCTCACAATCAACTCCCTGGCTATAGCCGCCCTCACCCTAATCCTAGCAGCCACCATAATAAAAGCCATTACAAAACAAGAG------GCCTATTAAA----TAAACCTACTTACCGCGCTCATATTAGCCACAGCTACTGCAGTAGCCGTAATTACCCTAAATCTATTAATATCAGAGATGACCCCAGACCCAGAAAAGCTTTCACCATATGAATGCGGATTTGACCCGCTGGGGTCCGCCCGCCTGCCGCTATCTATCCGCTTTTTCATAATCGCTATTTTATTCTTACTGTTTGACTTAGAGATCGCCATCCTACTACCGCTAGCATGGGCCCTGCAACTCACAAGCCTAATAAAAAGTATCACATGGGCCATCATTGTCTTTATGCTTATATTTGCAGGCCTAACATACGAATGACTACAAGGCGGACTAGAATGAGCAGAGTAGATGTTAAAAATCATTGTACCCACAATAATACTAATTCCCTCAACCTGCCTGACAGCCACAAAAAACACCTGATTATCGCCAACAGCCTACTCAGCAGTTATTATTATCCTAGGCATACTTGTCTTAAACCCCGGAGACACCCTGATGAACACTACTGGTCTACTACTAGGAAGTGACCAAATCTCAACACCCTTACTTATACTATCCTGCTGACTACTACCACTAATATTTATGGCTAGCCAAAGCTCCATGTCACACAACCCTGCCCAACAAAAACGACTGTTTATCACAGCCCTAGCCCTCCTACAATTAGCCTTAATATTAGTATTCATGGCCTTAGACCTAATGTTATTCTACACCACCTTTGAAGCAACCCTTATTCCCACCCTAATAGTGATCGCCCGATGGGGATCCCAAACAGAACGACTCGGAGCCGGACTATATTTCCTCCTATACACCATCACTAGCTCCATACCCCTTCTAATCGCACTTCTATGGGTGTATAACATAAAAGGAACTGCGTCTATTACACTCTTACAGCTACTCCCCCCAATAACCCTAACATTCTGAACAAACACGCTACTATGAACCTCACTCATATTGGCCTTCCTAGTAAAAATCCCAATTTACGGCCTCCACCTTTGGCTACCAAAAGCCCACGTAGAAGCCCCAATTGCCGGATCCATGGTCCTTGCAGCAATCTTATTAAAACTCGGGGGTTATGGCCTGCTACGAATTACAAACCTATTAACTGAACAAACTACATCCTCTTATATTCTCCCACTGGCAGTAGCACTATGGGGTGCACTCATAACCGGCATAGTCTGCCTACGACAAACAGATTTAAAATCCCTAATTGCCTACTCCTCAGTAAGCCACATAGGACTAATAACAAGCTCGATCCTCACTCACAATCAACTAGCCCCATCAGGATCAATAATTATAATAGTGGCCCACGGCCTTACATCTTCGATACTATTCTGCCTGGCAAATATTAATTACGAACGAACACACTCACGAACCCTATTACTTACACAAGGGGTACAACTAACCACCCCAGCCATGACGTCCTGATGACTCCTAGCCTGCTTAACAAACATAGCACTTCCCCCAACAATTAATTTCATTGGGGAACTCACCCTTATAGTCTCACTATTTGACTGAGCAGACATTACTATCTTTCTAACAGGACTAAGCGCATTCATCACCTCAATCTACACCCTACACATATTCTCCTCAACCCAACAGGGAACCCTTCCAACCCATATTATTACAATAAGTCCAACCCAAACTCGAGAACATCTACTAATAACACTGCACTCCGCACCATCAATCGCTTTAATCTTTATACCTCAACTAATATACTACCAATAA---ACCTCGCCTACTAACCTGTTATTTACCTTCTCTTTCATTATCTTCACCATTGGATTTACCTTCCGCCACACTCATCTCCTCTCAGCCCTATTATGCCTGGAGGGTATGATACTATCGGTGTTTTTACTAATAGCAACATGGTCTCTAAGCTCAAACATCTCCTCTTTCATCCTTCCTTTAACAGTTCTAACACTATCAGCCTGTGAAGCTGGCATCGGCCTCGCCCTACTGATCGCCTCAGCCCGAACACACAACACAGCCAACCTCAAAAACCTAAATCTGCTCCAATGTTAAAT-AACACAGCCATTAGCCTTAATTATCATACTGTTCCTGCTCCCCCTAGCAATCTTGACACTCCCAATACTAACACCAAGCT-CAAAACTAGCCTCA--CCACTTAAAACCAAAGTATTGGCAGCTAAACTGGCCTTCCTTACCAGCCTAATCCCACTGACCTACCTCATCCATAATGACTTAACCATCACTACATACGAAGCCCAATGATCAACAATTGGTACAACCACAATTCGCGTTAGCTTCACATTAGACATTTACTCCACCTTCTTCTTACCCATCCTACTTTTTGTCGTATGAGCTATCATAGAGTTTACCGTACAATACATAGAGTCAGACCTAAAAATTAACACTTTCTTTAACCAACTCACCACCTTCACCCTAATAATAGTAATCCTTGTAACTGCTGAAAACCTATTCCAATTTTTCATCGGCTGAGAGGGAGTGGGTATCATATCCTTCATGTTAATTAACTGATGATCCTTCCGATCAAACTCGAACAAAGCTGCCATGCAAGCTGTAATCTACAACCGCCTAGCAGACATTGGCCTAGTTGTCGCCCTGGCATGAATAGTTATCAACGACCTGTCCCTAGACATTAAAAGCATACAAGTCACGCCAGACATAGCCCTCATCCCTGCACTAGGGCTGCTTCTAGCAGCTGCTGGGAAATCCGCTCAGTTCGGATTCCACCCATGACTCCCCGCAGCGATAGAAGGCCCAACCCCTGTATCAGCCCTACTACACTCAAGCACCATGGTAGTAGCCGGAGTGTTCCTACTCATCCGGACCTCAGATATGCTATACAGCAACGAAACAGCAACTACAGCTTGCTTGCTACTAGGAGCCTTTACATCCATACTAGCTGCTTCATGCGCACTAACCCAAAATGACCTAAAAAAAATCATTGCCTACTCAACTACTAGTCAACTGGGCCTAATAATGACCTCCATCGGATTAAAACAGCCTGAACTCGCATTTATACACATCTCAACACACGCGTTCTTTAAAGCAATACTCTTCCTCTGCGCAGGAACGATCATCCACAGCCTGGGCAATGAACAAGACATCCGAAAAATAGGAGGGCTTAAAAAAGCACTTCCCACCACCACCTCCTGCCTAATCATTGGCTCCCTCGCCCTATCAGGAGTGCCATTCATGGCCGGCTTTTACTCAAAAGACGCCATCATTGAAGCCATCAACACCTCCCATGTAAACTCCATATCACTGGCCATAACCCTAGTAGCAACCATCTTCACCACACTCTACAGCCTGCGCATGATTTACTATGTAACCCTAAACACCCCACGAATCCTACCACTATCCGCCGTCTCCGAGACCCCTCAAACAACCAACCCCATTCTACGACTAGCTATCGGAAGCATCGCAGCCGGACTCATAATTTCAACCACTATTATACCACCCAACATCCCACAACTAACCATGCCAGCATCAGCCAAGCTAGCCGCACTAAACATCCTAATCCTGAGTCTACTAGTCGGCTCAATCCTAATTACAATAGCAAATCAGCTTCCCAACTCCATCAAGGGCACCCAAAACCCCCTTATCTCTAAAATCATCCACTCCTACTTCATTCTACATCATACCCTACCATCTATAATCTTACAGATTAGCCAAAAACTATCAACCCACCTGATAGACCAAACACACTATGAAGCCTTGGGCCCTAAAACAGTAACCCACCTACAAACACTAATAGCAAAACTACTAACTAATTTCCATAAAGCCCAAATTAACCCGTACCTAAAAATTGCCGTTCTATCCATCACACTAATCTCCTTACTCTACTTCACCTCAATGAACGCAGGGCCCCCCGATGCCGCCCACGAATTATAA------ATGGGAATTACATTTTTTCTTCTTTGTTTTTTAATGATGGTTAGTGTGGTGTTAGTGGCGTCGGGGGTGACGATCCATTATGGGGTGGTTAGTTTGCTTTTTGCTGCAATATTTGGTAGTGGGCTGTTGGTGGCGGGGGGCGGGAGTTTTATACCGCTTGTGGTACTGCTAATTTATTTGGGGGGTTTATTAGTAGTTTTCGCTTTTTGTGTTGGATTTACTGATGATAAATATTGTGAGTTTTGAGGAGCAGGAGGATCTAAGGTGTTGGCTTATGTTTGTGGGGCCGGCTTAGGTATTGGTGGATATTGTGTATACGGCTCTGCATGA----GCAGAGGTTTTAGGGTGTTTTG--TTGATGGTGTTGAGGCTTGGGGTGATGACGTGAGCAATGAGTTTTTAGGGGTTGGGTTATTTTATTTAAGTGGTTGGGGGCTTCTCATTTTAAGTGGTTGGGCTTTATTGGTGGTGTTGTTTACTATTATAATTATAATTCGTGGGCGGCATCGGGGGGCCCTGCGTTCATTGAGG

*Crocodylus siamensis* ATGACCCTGACCACAAATCTAAATTTATTTGACCAATTCTTAGTCCCCAAACTACTTGGCATATCACTACTAATCCCAGCCATGTTATTAACAACAGTGCTAATCTATAACCCGCAAGATCGCTGACTATCAAACCCTTTAACAACCCTACAGTCCTGATTAATTGCAAAAGCCACTAAACAGATCATAACCCCAGTAAATAAGCCAGGACATAAGTGATCATTAATACTGATCTCACTACTAACAATGCTTATTCTCAACAACCTTTTAGGCCTTCTCCCATACACGTTTACACCAACAACTCAGCTGTCTATAAATATAGCCCTGGCCCTCCCACTGTGGCTAGCAACAGTACTAATTGGCCTACGAAACAACCCAACCTCTTCACTAGCCCACCTCCTACCAGAAGGGACCCCAACACCCCTAATCCCAATCCTAATCCTAATCGAAACAATTAGCCTACTCATCCGACCAATCGCACTTGCCGTACGACTCACAGCCAATCTCACCGCAGGACATCTTCTAATACACCTAATCTCCACTGCGGCACTTAGCCTAATGACAACTTCCACACTACTTGCCGGACTAACCCTGATCATTCTAACCCTATTAATGCTCCTAGAAATTGCAGTAGCAATAATTCAAGCGTACGTGTTTACCCTACTACTCTCACTATACCTGCAAGAAAACGTATAAATGCCACAATTAAACCCCGAACCTTGATTAATAATCTTATCCATTACATGGCTAGTACTCATTACTACTTTACAGCCAAAAATTGCCTCTCTAAAGTTCATAAATAGCCCAAGCAGCCCCGCCCAAAAAACCACT-AAAACATGACCCTGACCACAAATCTAA---------------------ATGGCCCACCAACTACGAAAATCCCACCCACTCTTAAAACTAGTAAACCACTCTCTGATTGACTTGCCCACACCATCCAACATTTCCTACTGATGAAACTTTGGATCACTTCTCGGATTCACCCTATTAATCCAATTAGCATCGGGCGTTATACTAATAATGCACTTCCTAGCAGACGACTCTTTAGCTTTTATGTCCGTCGCCTACACTTCACGAGAAGTCTGATATGGTTGACTAATCCGAAGCCTCCATGCAAACGGAGCTTCCCTATTCTTCCTATGCACCTTCCTCCACATCGGACGTGGAGTATACTACGGGTCCTACCTAAATGAAAATACATGAAACATCGGAGTATTATTGTTACTACTACTGATAGCAACTGCCTTCATGGGCTACGTCCTACCATGGGGGCAGATGTCGTTCTGAGGGGCAACCGTGATCACCAACCTAATATCAGCCATCCCCTACGTAGGAGACTCAATTGTCACCTGAATTTGAGGGGGGCCGTCCGTCAATAGCGCAACCTTAACACGATTTACTACCCTGCACTTCCTGCTCCCATTTATCCTCATAGCCACCATCCTCACACACCTCATCTTCCTCCACGAACGCGGGTCATTCAACCCACTAGGATTAATCTCTAACGCCGACAAAATTCCATTCCACCCATACTTCTCAGCAAAAGACGCCATAGGCATGGCCCTGGCCACCGTCCTATTAATAACCCTTACATTCTACTTCCCAAACCTATTAGGAGACCCAGAAAACTTTACCCCTGCCAACCCCATAAAAACACCAGACCACATTAAACCAGAATGATATTTCCTATTCGCCTACACAATCTTACGATCTATTCCAAACAAACTCATAGGAGTCATTGCCATATTTGCATCCATCCTAGTATTACTACTCCTACCTGCACTACACACATCAAAACGACAATCAATAAGCCTACGTCCCTTATCCCAACTTTTATTCTGAGTCCTAATCGCAGACTTCTTTATCCTCACATGAATCGGGGGCCAGCCAGTACAAAACCCATACACCTTAATCGGACAAACAGCCTCCTTCATCTACTTCTTCACCATCCTTATCTTATTACCACTGACCGGAATAATTGAAAACTTAATAATCAAACCCCTTCGGTACCGGCCGT----------GTGAACATTAATCGTTGACTATTTTCCACTAACCACAAAGATATCGGCACCTTATATTTTATTTTCGGCGCCTGAGCTGGAATAGTAGGCACAGCCATAAGCCTATTAATTCGGACAGAGCTCAGCCAGCCTGGTCCATTCATAGGGGATGACCAAATTTATAATGTCATTGTCACAGCACATGCCTTTATCATAATTTTCTTTATAGTTATACCGATCATGATCGGCGGATTTGGAAATTGGCTACTCCCACTAATAATCGGAGCACCCGACATAGCATTTCCACGCATAAACAACATAAGCTTCTGACTGCTACCCCCATCATTTACCCTACTTCTCTCCTCAGCCTTTATTGAAACTGGAGCTGGCACCGGATGAACAGTCTACCCGCCCCTAGCTGGAAACCTAGCCCACGCCGGACCATCAGTAGACTTGACCATCTTCTCCCTCCACCTTGCTGGAGTATCATCCATTCTTGGAGCAATTAACTTTATTACCACGGCCATTAATATGAAACCCCCAGCAATATCACAACAACAGACGCCTCTTTTCGTATGGTCTATTCTAGTTACAGCCGTTCTCCTACTGCTCTCACTACCAGTCCTAGCTGCAGGAATTACCATATTACTCACTGATCGAAACCTGAACACCACCTTCTTTGACCCCGCAGGAGGAGGAGACCCAATCCTATACCAACACCTTTTCTGATTCTTTGGCCACCCAGAAGTATACATCCTCATCCTACCAGGGTTTGGAATAATCTCCCATGTAATCACCTTCTACTCAAGTAAAAAAGAACCATTTGGCTACATAGGAATAGTCTGAGCCATGATATCAATCGGCTTTCTCGGATTCATCGTCTGAGCCCACCACATATTTACAGTAGGAATAGACGTTGACACCCGAGCATACTTCACATCCGCCACAATAATTATCGCCATCCCCACCGGCGTAAAAGTGTTCAGCTGATTAGCCACTATTTACGGAGGAGTAGTGAAATGACAAGCCCCCATGCTCTGAGCACTCGGCTTCATTTTCTTATTCACAGTCGGAGGACTAACAGGAATTGTACTAGCTAACTCATCACTAGACATTATTCTACACGATACCTACTACGTAGTAGCCCACTTCCACTATGTACTATCTATGGGGGCAGTATTCGCCATCATAAGCGGATTCACTCACT-GGTTCCCACTA-TTTACAGGATTTACCCTCCACCACACATGAACAAAAA-TCCAATTCATAATCATATTCACGGGTGTAAACCTAACCTTCTTCCCACAACACTTCCTGGGCCTGTCAGGGATACCACGACGATATTCCGACTACCCAGATGCATATGCCTTCTGAAATATAATCTCCTCAATCGGATCGTTAGTTTCCATAGTATCAGTCGTCCTGCTCACATTTATTGTATGAGAGGCATTTTCATCAAAACGAAAAGTCCAAGTGCCTGAAATAGCAAGTACAAACGTAGAATGACTAAACAACTGCCCACCGTCATACCACACCTACGAAGAGCCAGTCTTTGTTCAAGTACGAAAAAAACTAACATAA------------------------------------ATGGCAAACCCAATACACCTAGGACTCCAAGATGCAATATCCCCGCTAATAGAAGAACTACTCTATTTTCATGACCACACACTAATAATTATTTTTTTAATCAGTATATTTGTACTATACACAATCTCAGTTTTATTACTAACAAGTCTATACCACACAAATGCAACAGATGTACAAGAAATAGAAATAATCTGAACCATTCTGCCAGCCCTAATCCTAATTACAATCGCCCTTCCATCCCTACGCACGCTATACCTTATAGACGAAACCACCAACCCCTGCCTAACCATTAAAGTTATCGGGCATCAATGGTATTGGACATATGAATATACAGACTTTTCCCAGCTGGAATTCGACTCTTACATGCTACCAACACAAGACCTGCCTCAAGGTCACTTCCGCCTTTTAGAAGTAGACCACCGCATGATTGTTCCAACAAGCTCAAGCACCCGAACATTAATCACAGCTGAAGACGTCCTACACTCATGAGCAGTACCATCCCTAGGGATCAAAATAGACGCAGTACCTGGACGACTAAACCAAACCTCACTAACATCCCCCAATCCTGGGGTATTCTATGG-CCAATGTTCTGAAATCTGCGGAGCAAACCATAGTTTTATGCCTATTGTCGTAGAAGCTGTCCCTATACAGCACTTCCAAAACTGATTAAAAACAAACTCAT----AAATGACCCACCAAACACACCTATTCCACATAGTCAACCCAAGCCCCTGGCCAATCATGGGGGCTATGGCTGCCATAATATTAACAGTCGGGCTGGTCCTATGATTCCACTGTAATTTAAACCTAATTTTATTTCTAGGACTAATCTCCACATTACTAATTATATTCCAGTGGTGACGAGATATTGTCCGAGAAAGCACCTATCTAGGCCACCATACCCCTCCAGTCCAAAAAGGACTACGATATGGCATAATCCTATTCATCACCTCAGAGGTCTTCTTTTTCCTTGGATTCTTCTGAGCGTTCTATCACTCAAGCTTAGCACCAACCCCAGAACTAGGAGGACAATGACCACCAACGGGAATTACCACACTAGACCCATTCGAAGTCCCACTTCTCAACACAGCAGTACTACTAGCCTCAGGGGTTACAGTAACATGAGCCCATCACAGCCTAATAGAAGCCAACCGAGCATCTGCCATCCACGCCTTAATTCTCACAATTATTCTAGGGCTATACTTCACTGCCCTTCAGGCAATAGAGTACTACGAAGCCCCATTCACCATCGCAGACAGCAGCTATGGGTCAACCTTCTTTGTTGCCACGGGCTTTCACGGCCTACACGTCATTATTGGATCAACATTCCTAATAACCTGCCTTTATCGACAAACCATACACCACTTCACCTCGAACCACCATTTCGGTTTCGAAGCTGCCGCTTGATACTGACATTTCGTAGACGTAGTCTGACTGTTCCTATACATCTCAATCTATTGATGAGGCTCCTATAAGCTTTTTAACAGCCACACCCATTCTTATCTATATCGTCTCAGTCCTAATCGCAGTCGCATTCCTGACAGGGCTAGAGCGAAAAATCATTGGCTACATGCAACTACGTAAAGGCCCCAATATTGTGGGCCCTTTCGGGCTGCTACAACCATTTGCTGATGGCCTCAAGCTTATTATTAAAGAACTAACATTGCCCCTACTCGCCACCCCTGCTTTATTTATTCTGTCCCCCACAGTCGCCATTATTTTATCCCTAATCATATGAGCCCCCCTACCCGTACCATTTTCTATCGCTAATCTAAACCTTGGCATATTATTTTTATTAGCCATATCCAGCTTAGCAGTTTACTCGCTACTATGGTCCGGGTGAGCATCAAACTCTAAATACGCCCTAATAGGCGCCCTACGAGCAGTAGCCCAAACCATTTCCTATGAAGTCACATTAGCCATCATTGTTTTATCTGTTGTCCTGCTTAGCGGCGGATTTTCACTTCATGCACTGGCTATTACCCAAGAACCCGCCTACCTGGCACTAACCACATGACCACTACTAATAATATGATACACCTCAACACTAGCAGAGACAAACCGCGCCCCGTTTGATCTTACAGAGGGTGAGTCAGAACTAGTATCTGGGTTCAACGTTGAATACAGCGCAGGGCTGTTCACACTATTCTTCCTGGCCGAGTACGCCAACATTCTACTAATAAATACTTTAACCACCATCCTATTCCTAAACACATCAACAAACTTGCCAACACAAACACTATTCACCACCACCCTAATAAGCAAATCAATTCTACTAACCATCGGATTCCTATGAATCCGAGCATCATACCCACGATTCCGGTATGATCAATTAATACACCTACTGTGAAAAAACTTCTTGCCAGCCACGCTGGCAATCTGCCTATGACACTCCTCGCTCCCGATGTCAACACTAGGCCTTCCAGTAGCAA---GGATGCCCATCTTCCAGCCAATCATCTTAACCACACTAACCATTACAACACTCATTTTCTTATCATCCACACACCTAGTACTCATGTGAGTAGCACTAGAACTTAGCACACTAGTGGTCCTACCATTGATTGCCAACAAATCGCACCCACGATCCATCGAAGCCTCCACAAAGTACTTCCTCACACAAGCCGCTGCTTCTGCCTTAATCATCTTCTCATGGACCTTAAACTATATCACAACTGGGGGCGGCCAAATTACAGAAGTAACAAACCAAGCCCTCACAACTATTATAACCCTGGCCCTCTTTATCAAAATTGGATTGGTGCCATTCCACTTCTGAGTACCTGAGACCATTCAAGGAATAGCTCCAACCGCCTCCATCTTCCTACTTACCTGACAAAAACTAGGCCCACTAATCATACTATACCTAATAAGCCCACTAATTAATTTCGAGGTCCTCTCTGCAGTGTCTATCCTCTCCGCCACAGTCGCCGGCTGACTTGGACTTAACCAAACCCAAATCCGGAAACTAGTAGCATTCTCCTCAATTGCCCAAATATCCTGAACACTCGTGATTATTAAATACGCACCATCACTTACAATCTTAGCCTTCTACCTATATTCAATCACAATCTCCGCTACACTTCTCACGTTAGAAAAACTATCAACAACATCCATTAACAACCTCCTACTTTCATTCCAAAAAGCCCCAGTTACTTCCTTACTGCTAACAATCTCCCTATTATCATTATCAGGCCTGCCCCCACTGGCCGGCTTCCTTCCAAAATGATTAACAATTGACCAGCTAGTGGCAGAAGGAGCAATTTGAATCGCATTCACAATACTCATAGCCTCCCTTCTGAGCCTATTCTTTTACCTACGACTATGATACAACTCCGCATCCACCCTTCCTCCTAACACTATTAATACCCAACGCCTATGACGCAAACCGACTCAACAAACTAACCTCACAATCAACTCCCTGGCTATAGCCGCCCTCACCCTAATCCTAGCAGCCACCATAATAAAAGCCATTACAAAACAAGAG------GCCTATTAAA----TAAACCTACTTACCACGCTCATATTAGCCACAGCTACTGCAGTAGCCGTAATTGCCCTAAATCTACTAATATCAGAGATAACCCCAGACCCAGAAAAGCTTTCACCATATGAATGCGGATTTGACCCGCTGGGATCCGCCCGCCTGCCACTATCTATCCGCTTTTTCATAATCGCTATTTTATTCTTACTGTTTGACCTAGAGATCGCCATCCTACTACCGCTAGCATGAGCCCTTCAACTCACAGGCCTAATAAAAAGTATCACATGGGCCATCATTGTCTTTATGCTTATATTTGCAGGCCTAACATACGAATGACTACAAGGCGGGCTAGAATGAGCAGAGTAAATGTTAAAAATCATTGTACCCACAATAATACTAATTCCCTCAACCTGTCTGACAGCCACAAAAAACACCTGACTATCGCCAACAGCCTACTCAGCAGTTATTATTATCCTAGGCATACTTGTCTTAAACCCCGGAGACACCCTGATGAACACTACTGGTCTATTACTAGGAAGTGACCAAATCTCAACGCCCTTACTTATACTATCCTGCTGACTACTACCACTAATATTTATGGCTAGCCAAAGCTCCATGTCACACAACCCCGCCCAACAAAAACGACTGTTTATCACAGCCCTAGCCCTCCTACAATTAGCCCTAATATTAGTATTCATGGCCTTAGACCTAATATTATTCTACACCACCTTTGAAGCAACCCTTATTCCCACCCTAATAGTGATCGCCCGATGAGGCTCCCAAACAGAACGACTCGGAGCCGGACTATATTTCCTCCTATACACCATCACTAGCTCCATACCCCTTCTAATTGCACTTCTATGAGTGTATAACATAAAAGGAACTGCGTCTATTACACTCTTACAACTACTGCCCCCAATAGCCCTAACATTCTGAACAAACACGCTACTATGAACCTCACTCATATTGGCCTTCCTAGTAAAAATCCCAATTTACGGCCTCCACCTTTGGCTACCAAAAGCCCACGTAGAAGCCCCAATTGCCGGATCCATGGTCCTTGCAGCAATCTTATTAAAACTCGGGGGTTATGGCCTGCTACGAATTACAAACCTATTAACTGAACAAACTACATCCTCTTATATTCTCCCACTGGCAGTAGCACTATGGGGTGCACTCATAACCGGCATAGTCTGCCTACGACAAACAGATTTAAAATCCCTAATTGCCTACTCCTCAGTAAGCCACATAGGACTAATAACAAGCTCGATCCTCACTCACAATCAACTAGCCCCATCAGGATCAATAATTATAATAGTAGCCCACGGCCTTACATCTTCGATACTATTCTGCCTGGCAAATATTAACTACGAACGAACACACTCACGAACCCTATTACTTACACAAGGAGTACAACTAACTGCCCCAGCCATGACATCCTGATGACTCCTAGCCTGCTTAACAAACATAGCACTTCCCCCAACAATTAATTTCATTGGAGAGCTCACCCTTATAGTCTCACTATTTGACTGAGCAGACATTACTATCTTTCTAACAGGGCTAAGCGAATTCATCACCTCAATCTACACCCTACACATATTCTCCTCAACCCAACAGGGAACCCTTCCAACCCATATTATTACAATAAGTCCAACCCAAACTCGAGAACATCTACTAATAACACTGCACTCCGCACCATCAATCGCTTTAATCTTTATACCTCAACTAATATACTGCCAATAA---ACCTCGCCCACTAATCTGTTATTCACCTTCTCTTTCATTATCTTCACCATTGGGTTTACCTTCCGCCACACCCATCTCCTCTCAGCCCTATTATGCCTGGAAGGTATGATACTATCAGTATTTTTACTAATAGCAACATGGTCTCTAAGCTCAAACATCTCCTCTTTCATCCTTCCTTTAACAGTACTAACACTATCAGCCTGTGAAGCTGGCATCGGCCTCGCCCTACTGATCGCCTCAGCCCGAACACACAACACAGCCAACCTCAAAAACCTAAATCTGCTCCAATGTTAAAT-AACACAGCCATTAGCCTTAATTATTATACTGTTCCTGCTCCCCCTAGCAATCTTGACACTCCCAATACTAACACCAAGCT-CAAAACTGGCCTCA--CCACTTAAAACCAAAGTATTGGCAGCTAAACTGGCCTTCCTTACCAGCCTAATCCCGCTGACCTACCTCATCCATAATGACTTAACCATCACTACATACGAAGCCCAATGATCAACAATTGGTACAACCACAATTCGCGTTAGCTTCACATTAGACATTTACTCCACCTTCTTCTTACCCATCCTACTTTTTGTCGTATGAGCTATCATAGAGTTTACCGTACAATACATAGAGTCAGACCTAAAAATTAACACTTTCTTTAACCAACTCACCACCTTCACCCTAGTAATAGTAATCCTTGTAACTGCTGAAAACCTATTCCAATTTTTCATCGGCTGAGAGGGGGTGGGTATCATATCCTTCATGTTAATTAACTGATGATCCTTCCGATCAAACTCGAACAAAGCTGCCATGCAAGCTGTAATCTACAACCGCCTAGCAGACATTGGCCTAGTTGTCGCCCTGGCATGAATAGTTATCAACGACCTGTCCCTAGACATTAAAAACACACAAGTCACGCCAGACATAGCCCTCATCCCTGCACTAGGGCTGCTTCTAGCAGCTGCTGGGAAATCCGCTCAGTTCGGATTCCACCCATGACTCCCCGCAGCGATAGAAGGCCCAACCCCTGTATCAGCCCTATTACACTCAAGCACCATGGTAGTAGCCGGAGTGTTCCTACTCATCCGGACCTCAAATATGCTATACAGCAACGAAACAGCAACTACAGCTTGCTTGCTACTAGGAGCCTTTACATCCATACTAGCTGCTTCATGCGCACTAACCCAAAATGACCTAAAAAAAATCATTGCCTACTCAACTACTAGTCAACTAGGCCTAATAATGACCTCCATCGGATTAAAACAACCTGAGCTCGCATTTATACACATCTCAACACACGCGTTCTTTAAAGCAATGCTCTTCCTCTGCGCAGGAACGATCATCCACAGCCTAGGCAATGAACAAGACATCCGAAAAATAGGAGGGCTTAAAAAGGCACTTCCCACCACCATCTCCTGCCTAATCATTGGCTCCCTCGCCCTATCAGGAATGCCATTCATAGCCGGCTTTTACTCAAAAGACGCCATCATTGAAGCCATCAACACCTCCCATGTAAACTCCCTATCACTGGCCATAACCCTTGTAGCAACCACCTTCACCACACTCTACAGCCTGCGCATGATTTACTATGTAACCCTAAACACCCCACGAATCCTATCACTATCAGCCGTCTCCGAGGCCCCTCAAACAACCAGCCCCATTCTACGACTAGCTATCGGAAGCATCGCAGCCGGACTCATAATTTCAGCCACTATTATACCACCCAACATCCCACAGCTAACCATGCCAACATCAGCCAAGCTAGCCGCACTAAACATCCTAATCCTGAGTCTACTAGTCGGCTCAATCCTAATTACAATAGCAAATCAGCTTCCCAACTCCATCAAGGGCACCCAAAACCCCCTTATCTCTAAAATCATCCACTCCTACTTCATTCTACATCATACCCTGCCATCTATAATCTTACAGATTAGCCAAAAACTGTCAACCCACCTGATAGACCAAACACACTATGAAGCCTTGGGCCCTAAAACAGTAACCCACCTACAAACACTAATAGCCAAACTGCTAACTAACTTCCATAAAGCTCAAATTAACCCGTACCTAAAAATTGCCATTCTATCCATCACACTAATCTCCTTACTCTACTTCACCTCAATGAACGCAGAGCCCCCCGATGCCGCCCACGAATTATAA------ATGGGAATTACATTTTTTCTTCTTTGTTTTTTAATGATGGTTAGTGTGGTGTTAGTGGCGTCGGGGGTGACGATCCATTATGGGGTGGTTAGTTTGCTTTTTGCTGCAATATTTGGTAGTGGGCTGTTGGTGGCGGGAGGTGGGAGTTTTATACCGCTTGTGGTATTGCTAATTTATTTGGGGGGTTTATTGGTAGTTTTCGCTTTTTGTGTCGGATTTACTGATGATAAATATTGTGAGTTTTGAGGGGTGGGAGGATCTAAGGTGTTGGCTTATGTTTGTGGGGCCGGTTTAGGTATTGGTGGATATTGTGTATACGACTCTGCATGA----GCAGAGGTTTTAGGGTGTTTTG--TTGATGGTGTTGAGGCTTGGGGTGATGATGTGAGTAATGAGTTTTTAGGGGTTGGGCTATTTTATTTAAGTGGTTGGGGGCTTCTCGTTTTAAGTGGTTGGGCTTTATTGGTGGTGTTGTTTACTATTATAATTATAATTCGTGGGCGGCATCGGGGGGCTCTGCGTTCATTGAGG

*Crocodylus palustris*

------------ACAAATCTAAACTTATTTGACCAATTCTTAGTACCCAAACTACTTGGCATATCACTATTAGCCCCAGCCATATTATTAACAGCAATACTAATCTACAACCCACAAGACCGCTGACTATCGAACCCATTAACAACCCTACAATCCTGATTAATTGCAAAAGCCACTAAACAAATCATAACCCCAGTAAACAAACCAGGACATAAGTGATCATTAATACTGATCTCATTACTAACAATGCTTATTCTCAACAACCTTTTAGGCCTTCTCCCATACACATTTACACCAACAACTCAGCTGTCTATAAACATAGCCCTGGCCCTGCCACTATGGCTGGCAACAGTGCTGGTTGGCCTGCGAAGCAAGCCGGCCTCTTCACTAGCCCACCTCCTGCCAGAAGGAACCCCAACACCCCTGATCCCTATCCTAATCTTAATCGAAACAATTAGTCTATTAATCCGACCAATCGCACTTGCCGTGCGACTCACAGCCAACCTCACCGCAGGACATCTTTTGATGCACTTAATTTCTACTGCAGCACTTAGCCTAATGACAACTTCCACACTACTTGCCGGGCTAACCCTAACCATCCTAGCCATACTGATCCTTCTAGAAATTGCAGTAGCAATAATCCAAGCATATGTATTCACCCTACTACTCTCACTATACCTGCAAGAAAACGTATAAATGCCACAATTAAACCCAGAACCTTGATTAATGATCTCATCTATCACATGGCTAGTATTCATTGTTACTTTACAGCCAAAAATTGCTTCTCTAAAGTTCATAAATAACCCGAGCAGCCCAGACCAAAAAACCACT-AAAACATGACCCTGGCCACAAATCTAA---------------------ATGACCCACCAACTACGAAAATCCCACCCACTCCTAAAGCTAGTAAACCACTCTTTAATTGACTTACCCACACCATCCAACATCTCCTACTGATGAAACTTCGGATCACTTCTTGGATTCACCCTATTAATCCAACTGGCATCGGGCATCCTACTAATAATACACTTCCTAGCAGATGACTCCTTGGCCTTCATATCCGTCGCCTACACCTCACGAGAAGTCTGATATGGATGACTAATCCGAAGCCTACATGCAAACGGGGCTTCTCTATTCTTCCTATGCATTTTCCTCCACATCGGACGTGGACTATACTACGGATCATATCTAAACGAAAATACATGAAACATTGGAGTACTGCTACTACTATTACTGATAGCAACTGCCTTCATAGGCTACGTCCTACCATGAGGTCAGATATCATTCTGAGGGGCAACCGTAATCACCAACCTAATATCAGCTATCCCCTACGTGGGAGACTCAATCGTGGCTTGAATCTGAGGAGGACCATCCATCAACAGCGCAACCCTAACACGATTTACTACCCTACACTTCCTGCTCCCGTTTATCCTCACAGCCACTGTCGTCACACACCTCATCTTCCTCCACGAACGCGGATCATTCAACCCACTAGGATTAATCTCTAACGCCGACAAAATTCCGTTCCACCCATACTTCTCGGCAAAAGACGCCATAGGCATAGCCCTAGCCACCGTTTTATTAATGACCCTTTCATTCTACTTCCCAAACCTGTTAGGAGACCCAGAAAACTTTACCCCTGCCGACCCAATAAAAACACCAGACCACATTAAACCAGAATGATACTTCCTATTCGCCTACACAATCTTACGGTCTATTCCAAACAAACTCGCAGGAGTTCTTGCCATGTTCGCATCCATCCTAGTATTATTACTTCTACCCGCACTACACACATCAAAACGACAATCAATAAGCCTGCGCCCCCTATCCCAACTCCTATTCTGAACCCTAACCGCAGACCTCCTCATTCTCACATGAATCGGAGGACAGCCAGTACAAGACCCATACATTCTAATTGGACAAATAGCCTCCTTCATCTACTTCTTTACCATCCTTATCCTATTACCACTGGCTGGAATAATTGAAAACATACTATTCAAACCCCTTCGGTACCGACCAT----------GTGAATATCAATCGTTGACTTTTTTCCACCAACCACAAAGATATCGGCACCTTGTATTTTATTTTCGGCGCCTGAGCCGGAATAGTAGGCACAGCCATAAGCCTGTTAATCCGAACAGAACTCAGCCAACCTGGCCCCTTCATAGGAGATGACCAAATTTACAATGTTATTGTTACAGCACATGCCTTTATCATAATCTTCTTCATAGTTATACCAATCATGATCGGCGGATTTGGAAATTGACTACTTCCATTAATAATTGGAGCACCCGACATAGCATTCCCTCGCATAAACAACATAAGCTTCTGACTACTACCCCCATCATTCACCCTACTTCTCTTTTCCGCCTTTATTGAAACCGGAGCTGGCACTGGATGAACAGTCTACCCACCCCTAGCTGGAAACCTAGCCCATGCCGGACCGTCAGTAGACTTAACCATTTTCTCCCTTCACCTTGCCGGAGTATCATCCATCCTTGGAGCAATTAACTTCATTACCACGGCTATCAACATAAAACCCCCAGCAATATCACAACAACAAACACCCCTTTTCGTATGGTCTGTTCTAGTTACAGCTGTTCTCCTGCTACTATCACTACCAGTTCTAGCCGCAGGAATTACTATATTACTCACCGACCGAAACTTGAACACAACCTTCTTTGACCCAGCAGGAGGAGGTGACCCAATCCTATACCAGCACCTTTTCTGATTTTTTGGCCACCCAGAAGTGTACATCCTTATCCTGCCAGGATTTGGAATAATCTCCCACGTAATTACCTTCTACTCAAGTAAAAAAGAACCATTTGGTTACATAGGAATAGTCTGAGCCATGATATCAATCGGCTTTCTTGGCTTCATTGTCTGAGCCCACCACATATTCACAGTTGGAATAGATGTTGATACTCGAGCATATTTCACATCCGCCACAATAATTATCGCCATCCCCACTGGCGTAAAAGTATTCAGCTGATTAGCCACTATTTACGGAGGAGTAGTGAAATGACAAGCCCCCATACTCTGGGCACTCGGCTTCATTTTCTTATTCACAGTTGGAGGACTAACAGGAATTGTACTAGCTAACTCATCACTAGACATTATTCTCCACGACACCTACTACGTAGTAGCCCACTTCCACTATGTATTATCTATAGGGGCAGTATTCGCTATCATAAGCGGATTTACCCACT-GATTCCCACTA-TTTACAGGATTTACCCTCCACCAAACATGAACAAAAA-TTCAATTCATAATTATGTTTACAGGCGTAAACCTAACCTTCTTCCCACAGCACTTCCTAGGTCTATCAGGAATACCACGACGATACTCAGACTACCCAGACGCATATGCCTTCTGAAATATAATCTCCTCAATTGGGTCATTAATCTCCATAGTATCAGTTATTCTACTCACATTTATTGTATGAGAAGCATTTTCATCAAAACGAAAAGTTCAAGTACCCGAAATAGCAAGCACAAATGTAGAGTGACTAAACAATTGCCCACCATCATACCACACCTACGAAGAACCAGTCTTTGTTCAAGTACGACCAAAACTAATGTAA------------------------------------ATGGCAAACCCAATACACCTAGGACTCCAAGATGCAATATCCCCACTAATAGAAGAACTCCTCTATTTTCATGACCACACACTAATAATTATTTTTTTAATCAGCATGTTTGTACTATACACAATCTCAGTTTTACTACTAACAAACCTATACCACACAAATGCAACAGATGTACAAGAAATAGAAATAATCTGAACCATTCTGCCAGCCCTAATCCTCATCACCATCGCCCTTCCATCCCTGCGCACATTGTACCTCATAGACGAAACCACCAACCCCTGCCTAACCATTAAAGTCATCGGGCATCAATGATATTGAACATATGAATACACAGACTTCTCCCAACTGGAATTCGACTCTTATATGCTTCCAACACAAGATCTGCCTCAGGGTCACTTCCGCCTTTTAGAAGTAGACCACCGCATGATTGTTCCAACAAACTCAAGCACACGAACACTAATCACAGCCGAAGACGTCCTGCACTCATGAGCAGTACCATCCTTGGGAATTAAAATAGACGCAGTACCAGGACGACTAAACCAAACCTCACTAACATCCCCCAACCCTGGAGTCTTCTATGGGCCAATGTTCCGAAATCTGCGGAGCAA-CCATAGCTTCATGCCCATTGTCGTAGAAGCTGTCCCAATACAGCACTTCCAAAGCTGATTAAAAACAAGCTCAT----AAATGATCCACCAAACACACCTATTTCACATAGTTAACCCAAGCCCATGGCCAATTATAGGAGCTATAGCTGCCATAATACTAACAGCCGGATTAGTCCTATGATTCCATTGTAATCTAGGCTTAATTCTGCTTCTGGGACTAATCTCAACCCTGCTAATTATATTTCAATGATGACGAGATATCGTCCGAGAAAGCACCTACATAGGCCACCACACCCCACCAGTCCAAAAAGGACTACGATATGGCATAATCCTATTTATCACCTCAGAGGTCTTCTTTTTCCTCGGGTTCTTCTGAGCGTTCTATCACTCAAGCTTGGCCCCAACCCCAGAACTAGGAGGACAGTGACCACCAACCGGAATTACCACACTAGACCCATTCGAAGTTCCACTCCTCAACACAGCTGTGCTACTAGCCTCGGGGGTCACAGTAACATGGGCCCACCACAGCCTAATAGAAGCCAACCGAGCATCCACCATCCACGCCTTAATTCTTACAATTATCCTAGGACTATACTTCACTGCTCTTCAAGCAATAGAATACTACGAAGCCCCATTCACCATCGCAGACAGCAGCTATGGATCAACCTTCTTTGTTGCCACAGGCTTTCACGGCCTACACGTCATTATTGGATCAACATTCCTAATAACCTGCCTCTATCGACAAATCATACACCACTTCACCTCAAATCACCACTTCGGTTTCGAAGCTGCCGCTTGATACTGACACTTCGTAGATGTAGTCTGACTATTCCTGTACATCTCAATCTACTGATGAGGCTCCTATAAGTTTTTTAACAGTCGCACCCATTCTTATCTATATCATCTCAGTCCTAATTGCAGTCGCATTCCTGACAGGACTAGAACGAAAAATCATTGGCTACATGCAACTACGTAAAGGCCCTAATATCGTGGGCCCCCTTGGGCTGCTGCAACCATTTGCTGACGGCCTCAAGCTTATTATCAAAGAGCTGACACTACCCCTGCTCGCCACCCCCGCTTTATTTGTTCTATCCCCAGCAGTCGCCCTCATTTTAGCCCTAATTATGTGGACCCCCCTGCCCGTACCATTTTCTATCGCCAACCTAAACCTTGGCATGTTATTTTTACTAGCCATATCCAGCTTAGCAGTTTATTCACTACTATGGTCCGGGTGAGCATCAAACTCTAAATACGCCCTAATGGGCGCCCTACGAGCTGTGGCCCAAACCATTTCTTATGAAGTCACACTAGCCATCATTGTTCTATCTATTGTCCTACTCAGTGGCGGATTCTCACTACACGCCCTAGCTGTTACCCAAGAGCCCATCTACTTGGCACTAACCACATGACCTCTACTGATAATATGATACACCTCAACACTAGCAGAGACAAACCGCGCCCCGTTTGACCTCACAGAAGGTGAGTCAGAACTAGTATCTGGATTCAACGTTGAATACAGCGCAGGACTATTCACACTATTTTTCCTAGCCGAATACGCCAACATTCTACTAATAAACACCCTAACCACCATCCTCTTCCTAAACACCTCAACAAACCTGCCAACACAAACATTATTCACCACCGCCCTAATAAGTAAATCAATCCTATTAACCATCGGATTCCTATGGGTCCGAGCATCATACCCACGATTCCGGTATGATCAATTAATACACCTACTGTGAAAAAACTTCTTGCCGGCCACGCTGGCAATCTGTCTGTGACACTCGTCGCTCCCAGTATCAACATTCGGTCTTCCAGTAACAA---GGATGCCCATCTTCCAACCAATTATCCTAACTACACTAACCATCACAACACTAATTTTTCTGTCCTCCACCCACCTAGTGTTAATGTGGGTAGCACTAGAACTCAGCACATTAGTAGTCCTACCGCTAATCGCTAATAAGTCGCACCCACGATCCATCGAAGCCTCCACAAAATACTTCCTCACACAGGCCACCGCTTCTGCCCTAATCATCTTCTCATGAACCTTAAACTATATCACAACCGGAGGCGGCCAAATCTCAGAAGTAACAAACCAAACCCTTACAACTATTATAGCCATAGCCCTATTTATTAAAATTGGATTAGTGCCATTCCACTTCTGAGTGCCTGAAACCATTCAAGGAATAACTCCAACCGCCTCCATCTTCCTACTCACCTGACAGAAACTAGGCCCACTAATCATATTATACCTAATGAGTCCACTAATTAATTTCGAGGTCCTCTCTGCAGTATCTATCCTGTCCGCCACAGTTGCCGGCTGACTTGGGCTTAACCAGACCCAAATCCGAAAGCTAGTAGCATTCTCTTCAATCGCCCAAATATCTTGAACCCTAGTGATTATTAAATACGCACCATCACTTACAATCCTAGCCTTCTACTTATATTCAATCACAATTTCCACTACCCTTCTCACACTAGAAAAATTATCAACAACATCCGTTAACAGCCTCTTACTTTCGTTCCAAAAAGCCCCAATTACTTCCTTATTGCTGACAATCTCTCTATTATCCTTATCAGGCCTACCCCCACTAGCCGGCTTCCTCCCAAAATGATTAACAATTGACCAGCTCGTGGCAGAAGGAGCAATTTGAGTAGCATTCACAATACTCATGGCCTCTCTCCTAAGCCTATTCTTCTACCTACGACTATGATACAACTCCGCATCCACCCTTCCTCCCAACACTGCTAATACCCAACGCCTATGACGCAAACCAGTCCAACAAACCAACCTTACAATCAACTCCCTGGCCATAGCCGCCTTCACCCTAATCCTAGCAGCCACCATAATAAAAGCCATCACAAAACAAGAG------GCATATTAAA----TAAACCTACTTACCGCATTCATGTTAGCCACAGCCACCGCAGTAGCCGTAATTACCCTAAATCTACTAATGTCAGAAATGGCCCCAGACCCAGAAAAACTTTCACCATACGAGTGCGGATTTGACCCGCTAGGGTCAGCCCGCCTGCCATTATCTATCCGCTTTTTCATAATCGCTATTTTATTCTTACTCTTTGACTTAGAGATCGCCATTCTACTACCGCTAGCATGGGCCCTCCAACTCACAAACCTGATCAAAAGCATCACATGGGCCATCATTATCTTCTTACTTATATTTGCAGGCCTAACATACGAATGACTACAAGGCGGACTAGAATGGGCAGAATAAATGTTAAAAATCGTTGTACCTACAATAATACTAATCCCCTCAACCTGCCTAACAGCCACAAAAAACACCTGACTATCACCGACAGCTTACTCAGCAGTCATTATTATCCTAGGCATGCTTGTCTTAAACCCCGGAGACATTCTGATAAACACTAGCGGTTTATTATTAGGAAGCGACCAGATTTCAACACCCCTGCTCATATTGTCCTGCTGACTATTACCACTGATATTCATGGCCAGCCAAAGCTCCATGTCACACAACCCAACCCAACAAAAACGACTTTTTATTACAGCCCTGGCCCTCCTTCAATTAGCCTTAATGTTGGTATTCATAGCCTTAGACCTAATGTTATTTTACACCGCCTTTGAAGCAACCCTTATTCCCACCTTAATAGTGATCGCCCGATGAGGCTCACAAACAGAACGACTCGGAGCCGGACTATATTTCCTCCTATACACCATCACTAGCTCTATGCCCCTTCTAATTGCACTTCTATGGGTATATAACATAAAAGGAACTGCGTCCATTACACTCTTACAACTATTACCACAAATAACCCTAACATTCTGAACAAACACATTACTATGAACTTCACTCATACTAGCCTTCCTAGTAAAAATCCCAATTTACGGCCTTCACCTTTGGCTCCCAAAAGCCCACGTAGAGGCCCCAATTGCCGGATCCATGGTCCTTGCAGCAATCTTATTAAAACTCGGAGGCTACGGCTTGCTACGAGTTACAAATCTACTAACTGAACAAACCACATCCTCTTACATCCTGCCCCTAGCAGTAGCATTATGGGGTGCACTCATAACCGGCATAATCTGCTTACGACAAACAGATTTAAAATCCCTAATCGCCTACTCCTCAGTAAGCCACATAGGACTAATAACAAGCTCAATCCTCACTCGTAATCAACTGGCCCCCTCGGGCTCAATAATTATAATAGTGGCCCACGGCCTTACATCCTCAATACTATTCTGCCTGGCAAACATTACTTACGAACGAACACACTCACGAACTCTACTACTAACACAAGGAGTACAACTAACCACCCCAATCATAACATCCTGATGACTCCTGGCCTGCTTAACAAACATAGCACTCCCCCCAACAATTAATTTTATTGGAGAACTCACCCTGATAGTATCACTATTTGACTGAGCGGACATTACCATCTTTCTAACAGGACTAAGCGCATTCATCACCTCAGTCTACACCCTACACATATTCTCCTCAACCCAGCAAGGAACCCTTCCAAACCACATCATCACAATAAGCCCAACCCAAACGCGAGAACATCTACTAATAACACTACACTCTGCGCCATCAATCGCTTTAATCTTTATCCCTCAACTAATGTATTACCAATAA---ACCTCCCCCACCAACCTACTATTTTCCCTCTCTTTCATTATCTTCACCATTGGATTTACCTTCCGCCACACTCATCTACTCTCAGCCCTACTATGCCTGGAAGGCATGATGCTATCGGTCTTCCTACTAATAGCAACATGGTCTCTAAACTCAAACATCTCATCTTTCATCCTGCCCTTAACAGTATTGACATTATCAGCCTGTGAAGCCGGTATTGGCCTCGCCCTATTGATCGCCTCAGCCCGAACACACAACACAGCCAATCTCAAAAACCTAAATCTACTCCAATGTTAAAT-AACACAGCCATTAGCCTTAATTATTATACTTTTCCTGCTCCCCCTAGCAATCTTGACACTCCCAATACTGATACCAAACT-CAAAATTAACCTCA--CCACTTAAAACCAAAGTACTAGCAGCAAAACTAGCCTTCCTTACCAGCCTAATTCCACTTACCTACCTCATCTACAATGACTTAACCATTACTACATACGAAACCCAATGATCAACAATTGGCACAACCACAATTCGCACTAGCTTCACATTAGACATCTACTCCGTCTTCTTCCTGCCTATTCTCCTTTTTGTTGTATGGTCCATCATAGAATTTACTGCACAATACATGGAATCAGACCTAAAAATTAACACTTTCTTTAACCAGCTCACCACCTTCACCCTAATAATGATAATCCTTGTAACTGCTGAAAACCTCTTCCAATTTTTCATTGGTTGAGAGGGAGTAGGTATCATATCCTTCATGTTAATCAACTGATGATCATTCCGATCAAACTCGAACAAAGCTGCTATGCAAGCCGTAATTTACAACCGCCTAGCAGACATTGGCCTAATTATTACTCTAGCATGAATAGCCATCAATGACCTGTCACTAAATATTAAAAGCATACAAATCACACCAGACATAGCCCTCATCCCCGCACTCGGGTTGCTCCTAGCAGCTGCCGGAAAATCTGCCCAATTTGGATTCCACCCATGACTCCCCGCAGCAATAGAAGGACCGACCCCTGTTTCAGCCCTACTGCACTCAAGCACCATGGTGGTAGCTGGAGTGTTCCTACTCATCCGAACCTCTGACCTGCTATACAGCAGCGAAACAGCAACTACAGCCTGCTTATTACTAGGAGCCTTTACATCCATACTAGCTGCTTCATGCGCATTAACCCAAAATGACCTAAAAAAAATTATTGCCTACTCAACTACTAGCCAGCTAGGCCTAATAATGACCTCCATCGGACTAAAACAACCCGAACTCGCATTCATACACATCTCGACACATGCATTCTTTAAGGCAATATTATTCCTCTGCGCAGGAACAATCATCCACAGCCTAAACAATGAACAAGACATCCGAAAAATGGGAGGACTCAAAAAAGCCCTCCCTACTACCTCCTCCTGCTTAATCATTGGCTCCCTTGCCCTATCAGGAATGCCATTCATGGCTGGTTTTTACTCAAAAGACGCCATCATTGAATCCATCAACACCTCCAATGTAAACTCCCTATCTCTGGCCATAACCCTAGTAGCAACCATCTTCACCACGCTCTACAGCTTACGCATGATCTACTATGTAGCCTTAAGCACTCCACGAATCCTACCCTTGTCAGCCATCTCCGAGACCCCTCAAATAACTAACCCTGTTATACGGCTAGCTATTGGAAGCATCGCAGCTGGGCTCATAATTTCAACCACTATCCTACCGCCTAATATTCCACAACTAACCATGCCGGCATCAGCCAAACTGGCCGCACTAAGCACCCTAATCTTGGGCCTACTAGTCGGCTCAATCCTGATTACAGTAGCAGATCAGTTTCCCAGCTCCACCAAAGGCACCCAAAACCCACTTATCTCTAAAATCATCCACTCCTACTTCATTCTACATCACACCCTATCATCCATAGTCCTACAAATTAGCCAAAAACTATCAACCCACCTGATAGACCAAACACACTATGAAGCCTTGGGCCCTAAAACAATAACCTACCTACAAATACTAATAGCCAAACTTTTAACTAACTTTCACAAAGCCCGAATTAACCCATACCTAAAAATTACCATTCTGTCCATCACACTAATTTCCCTATTCTACTTCACCTCAGTGAACGTAGAGCCCCCCGATGCCGCCCACGAACTAGAAT-----ATGGGAATTACATTTTTTCTTCTTTGTTGCTTAATGATAATTAGTGTGGTGTTAGTGGCGTCTGGGGTGACGATCCATTATGGGGTGGTTAGTTTGCTTTTTGCGGCAATATTTGGTAGTGGGTTGTTGGTAGCAGGGGGTGGAAGTTTTATGCCACTTGTGGTGCTACTAATTTACTTGGGTGGGCTGTTGGTAGTTTTTGCTTTTTGTGTTGGGTTCACTGATGATAAGTATTGTGAGCTCTGGGGGGCAGGGGGGTCTAAGGTGTCGGCTTATGTTTGTGGGGCTGGCCTAGGTATCGGTGGATACTGTATGTATAATTCTACATGA----GCTGAGGCTTTAGGGTGTTTTG--TTGATTCCGTTGAGACTTGGGGTGGCGATATAAACAATGAGTTTTTAGGAGCTGGGTTGTTTTATCTAAGTGGTTGGGGGCTCCTCATTTTGAGTGGTTGGGCTTTATTAGTGGTATTGTTTACTATTATAATTCTAGTTCGTGGGCGGCATCGGGGGGCTCTACGTTCACTGAGG

*Crocodylus niloticus*

------------ACAAATCTAAATCTATTTGACCAATTCTTAGTGCCTAAACTATTTGGCATATCACTATTAATCCCAGCCATACTGCTAACAACGCTGCTAATTTATAACCCGCAAGACCGCTGGCTATCAAACCCTTTAACAACCCTACAAGCCTGACTAATTGCAAAAACCACCAAACAAATCATATCCCCAGTAAACAAACCAGGACATAAGTGATCATTAATACTGATCTCACTACTAACAATGCTTATTCTAAACAACCTTTTAGGCCTTCTCCCATATACATTTACACCAACAACTCAACTGTCTATAAACATAGCCCTGGCCCTACCACTATGACTGGCGACAGTACTAATTGGCCTACGAAACAAACCGGCTTCTTCACTAGCCCACCTCCTGCCAGAAGGGACCCCAACACCCCTTATCCCAATCTTAATCTTAATCGAGACGATCAGCCTGCTCATCCGACCAATCGCACTCGCCGTGCGGCTTACAGCCAACCTCACCGCAGGCCATCTTCTGATACACCTAATCTCTACTGCGGCACTCAACCTAGTGACAACTTCCACACTACTTGCCGGACTTACCCTATCCATCCTAGCCCTGTTAATACTCCTAGAAATTGCAGTAGCAATGATCCAGGCATATGTATTTACTCTACTACTCTCACTATACCTGCAAGAAAACGTATAAATGCCACAATTAAACCCAGAACCCTGATTAATGATCTCATCCATTACATGGCTAGTATTCGTTATTACTTTACAGCCAAAAATTGCCTCTATAAAATTCATAAATAACCCAAGCAATCCAAACCAAAAAACCACT-AAAACATGACCCTGGCCACAAATCTAA---------------------ATGACCCACCAACTACGAAAATCCCACCCACTTTTAAAACTAGTAAACCACTCTTTAATTGACCTACCCACGCCATCCAACATCTCCTACTGATGAAACTTTGGATCACTTCTTGGATTCACCCTATTAGTCCAGCTGGCGTCGGGCATCCTACTAATAATGCATTTCCTAGCAGATGACTCCCTAGCTTTTATATCTGTCGCTTATACTTCACGAGAAGTTTGATACGGCTGACTAATTCGAAGCCTCCATGCAAACGGAGCCTCCCTATTCTTCCTGTGTATTTTCCTACACATTGGACGCGGAGTGTACTACGGATCATACCTCAACGAAAATACATGAAACATCGGAGTGCTACTACTGCTGCTACTGATAGCAACTGCCTTCATAGGCTACGTCCTACCATGGGGACAGATATCATTCTGAGGGGCAACCGTAATCACCAACCTAATATCAGCCATCCCATACGTGGGGAACACAATTGTAACCTGAATTTGAGGGGGACCATCAGTCAACAGCGCAACCCTAACACGATTCACCACTTTGCACTTCCTACTACCATTTATCCTCCTGGCCGCAGTTATCACGCACCTCATCTTCCTTCACGAACGCGGATCATTCAACCCGCTAGGACTGACCTCCAACGCCGACAAAATTCCGTTCCACCCGTATTTCTCAGCAAAAGACGCCATAGGCATAGCTATAGCCGCCGTCCTGTTAACAACCCTTACATTCTACTTCCCAAACCTATTAGGAGACCCAGAAAACTTCACCCCTGCCGACCCCATAAAAACACCGGACCACATTAAACCAGAATGATACTTCCTATTTGCCTACACGATCCTACGATGTATCCCAAACAAACTCACAGGAGTCCTTGCCATGTTCGCATCCATCCTAGTACTATTACTCCTACCTACACTACACACATCAAAACGGCAATCAATAAGCCTACGCCCCCTATCCCAACTCCTATTCTGAGCCCTAATCGCAGATTTCTTTATTCTTACATGAATCGGAGGACAACCAGTACAGGACCCCTACATCCTAATTGGGCAAGTAGCCTCCTTCATATACTTCTTCACCATCCTTATCCTATTACCACTAGCTGGAATAATTGAAAACCTATTAATCAAACCCCTTCGGTACCCGACCT----------GTGAATATTAATCGTTGACTTTTTTCCACTAACCACAAAGATATCGGCACCTTGTATTTTATTTTCGGCGCCTGAGCCGGAATAGTAGGCACAGCCATAAGCCTATTAATCCGAACGGAGCTCAGCCAGCCAGGCCCCTTCATAGGAGATGACCAAATTTACAATGTTATTGTTACAGCACATGCCTTTATCATAATTTTCTTTATAGTTATACCAATTATGATCGGAGGATTTGGAAATTGACTACTCCCATTAATAATTGGAGCACCCGACATAGCATTCCCTCGCATAAACAACATAAGCTTCTGATTGCTACCCCCATCATTTACCCTACTTCTCTTTTCCGCCTTTATTGAAACCGGGGCTGGCACCGGATGAACAGTCTACCCACCGCTAGCTGGAAACCTAGCCCACGCCGGACCATCAGTAGACCTCACTATTTTCTCCCTTCACCTTGCTGGGGTGTCATCCATCCTTGGAGCAATTAACTTTATCACCACGGCTATCAACATAAAACCACCAGCAATGTCACAACAACAAACGCCCCTCTTTGTGTGATCTGTTCTAGTTACAGCTGTCCTCCTACTGCTCTCACTACCCGTCCTAGCTGCAGGAATTACTATACTACTAACTGACCGAAACTTGAACACCACTTTCTTTGACCCCGCAGGAGGAGGAGACCCAATCCTATACCAACACCTCTTCTGATTTTTCGGCCACCCAGAGGTATATATCCTTATCCTACCAGGGTTTGGAATAATCTCCCACGTAATTACCTTCTACTCAAGCAAAAAAGAACCATTTGGCTATATAGGAATAGTCTGAGCCATAATGTCAATCGGCTTCCTTGGCTTCATTGTCTGAGCCCACCATATGTTTACAGTAGGAATAGATGTTGACACTCGAGCATATTTCACATCCGCCACAATAATTATCGCCATCCCCACTGGTGTAAAAGTATTCAGTTGATTAGCCACTATTTACGGAGGAGTAGTAAAATGACAAGCCCCCATACTCTGAGCACTCGGCTTCATTTTCTTATTCACAGTCGGAGGACTTACAGGAATTGTACTAGCTAACTCGTCACTAGACATTATTCTCCACGATACCTACTACGTAGTAGCCCACTTCCACTATGTGCTATCTATAGGAGCAGTATTCGCCATCATAAGCGGATTCACCCACT-GATTCCCATTA-TTTACAGGATTCACCCTACACAGCACATGAACAAAAA-TTCAATTCATAATTATGTTTACAGGCGTAAACCTAACCTTCTTCCCACAGCACTTCCTAGGCCTATCAGGAATACCACGACGATATTCAGACTACCCGGATGCATATGCCTTCTGAAATATAATTTCCTCGATCGGGTCATTAATCTCCATAGTGTCAGTTATCCTACTCACATTTATTGTATGAGAAGCATTTTCATCAAAACGAAAAGTTCAAGTACCTGAAATAGCAAGCACAAATGTAGAATGACTAAATAATTGTCCACCATCATACCACACCTACGAAGAACCGGTCTTTGTTCAAGTACAGCCAAAACTAACGTAA------------------------------------ATGGCAAATCCAATACACCTAGGACTCCAAGATGCAATATCCCCATTAATAGAAGAACTCCTCTATTTTCATGACCACACACTAATAATTATTTTTCTAATCAGCATGTTTGTACTTTATACAATCTCAGTTCTACTGCTAACAAACCTATACCACACAAATGCAACAGACGTACAAGAAATAGAAATAATCTGAACCATTCTGCCAGCCCTAATCCTGATTACCATCGCCCTTCCATCTCTACGCACGCTATACCTCATAGACGAAACCACCAACCCCTGCCTAACCATTAAAGTCATCGGACATCAGTGATACTGAACATATGAATATACAGACTTTTCACAGCTAGAGTTCGACTCTTATATACTACCAACACAAGACCTACCCCAAGGTCATTTCCGCCTCTTAGAAGTAGACCACCGCATGATTGTCCCAACAAACTCAAGCACCCGAACATTAATCACAGCTGAAGACGTCCTACACTCATGAGCGGTACCCTCCTTAGGAATCAAAATAGACGCAGTGCCAGGACGACTAAATCAAACCTCACTAACATCCCCCAACCCTGGAGTTTTCTATGG-CCAATGCTCTGAAATCTGCGGGGCAAACCATAGTTTCATGCCTATTGTCGTAGAAGCTGTACCAATACAGCACTTCCAAAGCTGATTAAAAACAAACTCAT----AAATGACCCACCAAATACACCTATTTCACATAGTCAACCCAAGCCCGTGACCAATTTTAGGGGCTATGGCTGCCATAATACTAACAGCCGGATTAGTCCTATGATTCCACTGTAATATAAACCTAGTTTTACTCATAGGACTAATCTCAACCTTACTGATTATATTTCAATGATGACGAGACGTCGTCCGAGAAAGCACCTATTTAGGCCACCATACCCCGCCAGTCCAAAAAGGCCTACGATACGGTATAATCCTCTTTATCACCTCAGAAGTCTTCTTCTTCCTCGGGTTCTTTTGAGCGTTCTATCACTCAAGCTTGGCTCCAACCCCAGAACTGGGAGGACAATGACCGCCAACCGGAATTACCACACTAGACCCATTCGAAGTTCCACTCCTCAACACAGCAGTTCTACTAGCCTCTGGGGTTACAGTGACATGAGCCCACCACAGCCTAATGGAAGCCAACCGAGCACCCGCCATCCACGCCTTAACTCTCACAATTATTCTAGGGCTATACTTCACTGCTCTTCAAGCAATAGAATATTACGAAGCCCCATTCACCATCGCAGACAGCAGCTATGGCTCAACCTTCTTTGTTGCCACAGGCTTTCACGGCCTACATGTCATTATTGGCTCAACATTCCTAATGACCTGCCTATATCGACAAATCATACACCACTTCACCTCAAATCACCATTTCGGCTTCGAAGCCGCCGCTTGATATTGACATTTCGTAGATGTAGTCTGACTGTTCCTATACATCTCAATCTATTGATGAGGCTCCTATAAGTTTTTTAACAACCACGCCCATTCTTATCTACATCATCTCAGTCCTAATCGCAGTCGCATTCCTGACAGGACTAGAACGAAAAATCATTGGCTATATACCACTGCGCAAAGGCCCTAACATTGTCGGCCCTCTTGGGCTGCTACAGCCATTCGCCGACGGCCTCAAGCTTATTATCAAAGAGCTAACACTGCCCCTACTCGCCACCCCTGCTTTATTTGTTTTTTCCCCCGCAGTCGCCCTCATTTTATCCCTAATTATGTGGGCCCCCCTGCCCGTACCATTTTCTATCGCCAATCTAAACCTTGGCATGCTATTTTTATTAGCCATATCCAGCTTAGCAGTCTACTCACTACTGTGATCCGGATGAGCATCAAACTCTAAATACGCCCTAATAGGCGCCTTACGAGCGGTAGCCCAAACCATCTCCTACGAAGTCACATTAGCCATCATTGTTCTATCTATCGTCTTACTTAGCGGTGGATTTTCACTGCACGCACTAGCTGTAACCCAAGAGCCTATCTACTTGGCACTAACCACATGACCACTACTGATAATATGATACACCTCAACATTAGCAGAAACAAACCGCGCCCCATTTGACCTCACAGAAGGTGAATCAGAACTAGTATCTGGCTTCAACGTTGAGTACAGCGCAGGGCTGTTCACACTATTTTTTCTAGCTGAATACGCTAACATCCTACTAATAAACATTTTAACCACCATCCTCTTCCTAAACACAACAACAAACCTGCCAACACAAACACTATTCACCACCACCCTAATAAGTAAATCGATTCTACTGACCATCGGATTCCTATGAATCCGAGCATCATACCCACGATTCCGGTATGATCAATTAATACACCTGCTGTGAAAAAACTTCTTGCCAGCCACCCTGGCAATCTGCCTATGACACTCGTCGTTCCCTGTGTCAACATTCGGTCTTCCAGTAGCAA---GGATGCCCATCTTCCAACCCGTCATCTTAACCACACTAACTATTACAACACTCATTTTCCTGTCCTCCACCCATCTGGTGCTTATGTGGGTGGCACTAGAACTTAGCACATTAGTAGTTCTGCCATTAATTGCCAATAAGTCGCACCCACGATCCATTGAAGCCTCCACAAAATACTTCCTCACACAAGCCACCGCTTCTGCCCTAATCATCTTCTCATGGACCTTAAACTATATCACAACCGGGGGCGGCCAAATTACAGAGGTAACAAACCAAACACTTACAACCATTACAGCCCTAGCCCTGTTTATCAAAATTGGATTAGTGCCATTCCACTTCTGAGTGCCCGAAACCATTCAAGGAATAACCCCAACCGCCTCCATCTTCCTACTTACCTGACAAAAACTAGGCCCACTAATTATATTATACCTAATGAGCCCACTAATTAATTTCGAAGTCCTCTCTGCAGTATCTATCCTATCCGCCACAGTTGCCGGCTGGCTTGGACTTAATCAAACCCAAATCCGAAAACTAGTGGCATTCTCTTCAATCGCCCAAATAGCCTGGACCCTAGTGATTATTAAATACGCACCATCACTTACAATCCTAGCCTTCTACCTGTATTCAATCACAATCTCCACCACGCTTCTCACACTAGAAAAACTATCAACAACATCCATTAACAACCTCCTACTTTCGTTCCAAAAAGCCCCAATTACTTCCTTACTACTAATGGTCTCTTTATTATCCCTATCAGGACTACCCCCACTAGCCGGCTTCCTACCAAAATGACTAACAATCAACCAGCTCGTAGCAGAGGGGGCAATTTGAGTCGCATTCACAATACTCATGGCCTCTCTTCTAAGCCTTTTCTTTTACCTCCGGCTATGATACAATTCCGCATCCACCCTTCCCCCAAACACTACTAATACCCAACGACTATGACGCAAACCAGTTCAACAAACCAACCTCACAATCAACTCCCTAGCTATAGCCGCCCTCACCCTAATCCTAGCAGCCACCATAATAAAAGCCACTACAAAACAAGAG------ACCCATTAAA----TAAACCTTCTCACTGCATTCATATTAGCCACAGCCACTGCAGTAGCCGTAATTACCCTAAACCTACTGATATCGGAAATGGCCCCAGACCCCGAAAAACTCTCACCATACGAGTGCGGATTCGACCCCCTAGGGTCCGCCCGCCTGCCACTATCTATCCGCTTTTTCATGGTCGCTATCTTATTCCTACTATTTGACCTAGAAATCGCCATCCTACTACCACTAGCATGGGCCCTTCAACTCACAAACCTGATCAAAAGTGTCACATGGGCCATCATTGTCTTCTTACTCATATTTGCAGGTCTAACATACGAATGATTACAGGGCGGACTAGAATGGGCAGAATAGATGTTAAAAATCATTGTACCAACAATAATACTAATCCCATCAACCTGCCTAACAACCACAAAAAACACCTGACTATCACCTACAGCCTACTCAGCAGTTATTATTATCCTAGGCATACTTGTCTTAAACCCCGGGGATATTCTGATAAATACTAGCGGTTTATTACTAGGAAGCGACCAAATCTCAACACCCCTACTTATATTATCCTGCTGACTACTACCATTAATATTTATAGCCAGCCAAAGTTCCATGTCACACGACCCAACTCAACAAAAACGACTATTTATTACAGCCCTAGCCCTCCTGCAATTAGCCCTAATATTAGTATTCATGGCCTTAGACCTAATATTATTTTACACCGCCTTTGAAGCAACCCTCATTCCCACCCTAATAGTAATCGCCCGATGGGGCTCCCAAACAGAACGGCTCGGAGCCGGACTATACTTCCTCCTATACACCATCACTAGCTCCATACCGCTCCTAATTGCACTTCTATGGGTATATAACATAAAAGGAACTACATCCATCACACTCTTACAACTATTACCCCAAATAACCCTAATATTCTGAACAAGCACATTACTATGAGTCTCCCTTATATTAGCCTTCCTAGTAAAAATCCCAATTTACGGCCTTCACCTCTGGCTACCAAAAGCCCACGTAGAAGCCCCCATTGCCGGATCCATGGTCCTTGCAGCGATCCTGTTAAAACTTGGGGGCTATGGCCTGCTACGAATTACAAATACACTAACTGAACAAGCCACATTCTCCTACATCCTACCCCTAACAGTAGCACTATGAGGTGCACTCATAACTGGCATAATCTGCTTACGACAAACAGACTTAAAATCCCTAATTGCCTACTCCTCAGTAAGCCACATAGGATTAATAACAAGCTCAATCCTTACTCGCAATCAACTAGCCCCCTCAGGCTCAATAATTATAATAGTAGCTCACGGCCTTACATCCTCAATACTATTCTGCTTGGCAAATATTACTTACGAACGAACACACTCACGAACCCTGCTACTAACGCAAGGAGTACAACTCACCACCCCAATCATGACATCCTGGTGGCTTCTGGCCTGCTTAACAAACATAGCACTCCCCCCAACAATTAACTTTATTGGAGAGCTCACCCTTATAGTATCACTATTTGACTGAGCGGACATTACTATTTTTCTAACAGGACTAAGCGCATTCATCACCTCGATTTACACCCTACACACATTCTCCTCCACCCAACAAGGAACCCTACCAAACCACATTATTACGATAAGCCCAACCCAAACGCGAGAACATCTACTAATAGCACTACACTCTGCACCATCAGTTGCTTTAATTATTGTCCCCCAACTGATGTATTACCAATAA---ACCTCCCCCACTAATCTACTGTTCACCATTTCTTTCATCATCTTCACCATTGGGTTCACCTTCCGTCACACTCATCTCCTCTCAGCCCTATTATGCCTGGAAGGCATGATGTTGTCGGTATTTCTATTAATGGCAACATGATCTATAAACTCAAACATCTCATCGTTCATCCTGCCTTTAACAGTCCTAACACTATCAGCCTGTGAAGCCGGCATTGGCCTCGCCCTACTGATCGCCTCAGCCCGAACACATAATACAGCCAACCTCAAAAACCTAAACCTACTCCAATGTTAAAT-AACACAGCCCTCAGCCTTAATTATTATACTTTTCCTGCTACCCCTAGCAATCTTAACACTCCCAATACTAGCCCCAAGCT-CAAAATTGACCTCG--CCACTTAGCACCAAAGTATTGATAGCAAAACTAGCCTTCCTTACTAGCCTAATCCCACTGGCCTATCTCATCTACAATGACCTAACCATTACTACATACGAAACCCGATGATCAGAAATTAGCACAACCACAATTCGCATTAGCTTCACGCTAGACATTTACTCCACCTTCTTCTTACCAATCCTCCTTTTTGTCGTATGGTCCATCATAGAATTTACAGTACAATACATGGAGTCAGACATAAAAATTAACACTTTCTTTAACCAACTCACCACCTTCACCCTAATAATAATAATTCTTGTGACCGCTGAAAACCTCTTCCAATTTTTCATCGGCTGAGAAGGAGTTGGCATCATGTCCTTTATGCTAATTAACTGGTGATCATTCCGATCAAGCTCGAACAAAGCGGCCATGCAAGCCGTAATCTATAACCGCTTAGCAGACATTGGCCTAGTTATCACCCTGGCATGAATAGTCATCAACAACCTGTCCCTAAGTATTAAAAGCATACAAATCACACCAGACATAGCCCTCATCCCCGCACTCGGGCTGCTTCTAGCAGCGGCCGGAAAGTCTGCTCAATTCGGATTCCACCCATGACTCCCCGCAGCAATAGAGGGTCCAACCCCCGTCTCGGCCTTACTGCACTCAAGCACTATAGTAGTAGCCGGAGTATTCCTACTCATCCGAACCTCAGACCTCCTATACAGCAGCGAAACAGCAACTACAGCCTGCCTACTACTGGGGGCCTTTACATCCATACTAGCCGCCTCCTGCGCACTAACCCAAAATGACCTAAAAAAAATTATTGCCTACTCAACTACTAGCCAGCTAGGCCTAATAATGACCTCCATCGGGTTAAAACAACCTGAGCTCGCATTCATACACATCTCAACACACGCATTCTTTAAAGCAATGCTATTCCTATGCGCAGGGACAATTATCCACAGCCTAGACAACGAACAAGACATCCGAAAGATGGGGGGACTCAAAAAAGCGCTTCCTACTACATCCTCCTGCTTAATTATTGGCTCCCTTGCCCTCTCAGGAATACCATTCATGGCTGGCTTTTACTCAAAAGACGCCATCATCGAGTCAATCAACACCTCCAACGTAAACTCCCTATCACTAGCCATGACCCTAGTGGCAACCACCTTCACCACACTTTACAGCCTGCGTATGATCTACTATGTGGCCCTAAGCACCCCACGAATCCTACCACTATCCGCCATCTCCGAAACCCATCAGACAACCAACCCAATTCTACGGCTGGCCATTGGAAGCATCGCAGCCGGACTCATAATTTCAACCACTATTTTACCGCCAAATATTCCACAACTGACCATACCAACATCAACCAAGCTAACCGCACTAAGTATCCTAGTCCTAGGATTACTAGTCGGCTCAATCCTAATCACAGTAGCAGACCAACTTCCCAGCTCCACCAAGGGCACCCAAAACCCCCTTATCTCTAAGATCATCCACTCTTACTTCATTCTGCACCACACCCTATCATCCATAGTCTTACAAATTAGCCAAAAATTATCAACCCACCTAATAGACCAAACACACTATGAAGCCTTAGGCCCTAAAACAATAACCTATCTACAAATACTAATAGCCAAACTATTAACTAGCCTCCATAAAGCCCGAATTAACCCGTACTTAAAAATCATTATCCTATCCATCATATTAATCTCCCTATTCTACTTCACCTCGATGAACGCAGGGCCCCTCGATGCCGCCCACGAACTAGAAT-----ATGGGAATTACATTTTTTCTTATTTGTTGTTTAATGATAATTAGTGTGGTGTTAGTGGCGTCTGGGGTGACGATCCACTATGGGGTGGTTAGTTTGCTTTTTGCTGCAATATTTGGTAGTGCATTGTTAGTAGTAGGGGGTGGAAGTTTTATGCCGCTTGTGGTACTACTAATTTATTTGGGCGGCTTGTTGGTGGTTTTTGCTTTTTGTGTGGGGTTCACTGATGATAAATATTGTGAGTTCTGGGGAGCAGGGGGGTCTAAGGTGTCGGCTTGTGTTTGTGGGGTTGGCTTAGGCGTTGGTGGATATTATATATATGACTCTGCGTGA----GCTGAGGTTTTAGGGTGTTTTG--TTGATGCCGTTGAGACCTGGAGTGGTGATGTTAGTAATGAGTTTTTAGGGGTTGGGCTGTTTTATATGAGTGGCTGGGGGTTTCTCATTTTAAGTGGCTGAGCCTTATTGGTGGTATTGTTTACTATTATGATTCTAGTTCGTGGGCGGCATCGAGGGGCCCTGCGTTCATCGAGG

*Crocodylus niloticus 2*

------------ACAAATCTAAATCTATTTGACCAATTCTTAGTGCCTAAACTATTTGGCATATCACTATTAATCCCAGCCATACTGCTAACAACGCTGCTAATTTATAACCCGCAAGACCGCTGGCTATCAAACCCTTTAACAACCCTACAAGCCTGACTAATTGCAAAAACCACCAAACAAATCATATCCCCAGTAAACAAACCAGGACATAAGTGATCATTAATACTGATCTCACTACTAACAATGCTTATTCTAAACAACCTTTTAGGCCTTCTCCCATATACATTTACACCAACAACTCAACTGTCTATAAACATAGCCCTGGCCCTACCACTATGACTGGCGACAGTACTAATTGGCCTACGAAACAAACCGGCTTCTTCACTAGCCCACCTCCTGCCAGAAGGGACCCCAACACCCCTTATCCCAATCTTAATCTTAATCGAGACGATCAGCCTGCTCATCCGACCAATCGCACTCGCCGTGCGGCTTACAGCCAACCTCACCGCAGGCCATCTTCTGATACACCTAATCTCTACTGCGGCACTCAACCTAGTGACAACTTCCACACTACTTGCCGGACTTACCCTATCCATCCTAGCCCTGTTAATACTCCTAGAAATTGCAGTAGCAATGATCCAGGCATATGTATTTACTCTACTACTCTCACTATACCTGCAAGAAAACGTATAAATGCCACAATTAAACCCAGAACCCTGATTAATGATCTCATCCATTACATGGCTAGTATTCGTTATTACTTTACAGCCAAAAATTGCCTCTATAAAATTCATAAATAACCCAAGCAATCCAAACCAAAAAACCACT-AAAACATGACCCTGGCCACAAATCTAA---------------------ATGACCCACCAACTACGAAAATCCCACCCACTTTTAAAACTAGTAAACCACTCTTTAATTGACCTACCCACGCCATCCAACATCTCCTACTGATGAAACTTTGGATCACTTCTTGGATTCACCCTATTAGTCCAGCTGGCGTCGGGCATCCTACTAATAATGCATTTCCTAGCAGATGACTCCCTAGCTTTTATATCTGTCGCTTATACTTCACGAGAAGTTTGATACGGCTGACTAATTCGAAGCCTCCATGCAAACGGAGCCTCCCTATTCTTCCTGTGTATTTTCCTACACATTGGACGCGGAGTGTACTACGGATCATACCTCAACGAAAATACATGAAACATCGGAGTGCTACTACTGCTGCTACTGATAGCAACTGCCTTCATAGGCTACGTCCTACCATGGGGACAGATATCATTCTGAGGGGCAACCGTAATCACCAACCTAATATCAGCCATCCCATACGTGGGGAACACAATTGTAACCTGAATTTGAGGGGGACCATCAGTCAACAGCGCAACCCTAACACGATTCACCACTTTGCACTTCCTACTACCATTTATCCTCCTGGCCGCAGTTATCACGCACCTCATCTTCCTTCACGAACGCGGATCATTCAACCCGCTAGGACTGACCTCCAACGCCGACAAAATTCCGTTCCACCCGTATTTCTCAGCAAAAGACGCCATAGGCATAGCTATAGCCGCCGTCCTGTTAACAACCCTTACATTCTACTTCCCAAACCTATTAGGAGACCCAGAAAACTTCACCCCTGCCGACCCCATAAAAACACCGGACCACATTAAACCAGAATGATACTTCCTATTTGCCTACACGATCCTACGATGTATCCCAAACAAACTCACAGGAGTCCTTGCCATGTTCGCATCCATCCTAGTACTATTACTCCTACCTACACTACACACATCAAAACGGCAATCAATAAGCCTACGCCCCCTATCCCAACTCCTATTCTGAGCCCTAATCGCAGATTTCTTTATTCTTACATGAATCGGAGGACAACCAGTACAGGACCCCTACATCCTAATTGGGCAAGTAGCCTCCTTCATATACTTCTTCACCATCCTTATCCTATTACCACTAGCTGGAATAATTGAAAACCTATTAATCAAACCCCTTCGGTACCCGACCT----------GTGAATATTAATCGTTGACTTTTTTCCACTAACCACAAAGATATCGGCACCTTGTATTTTATTTTCGGCGCCTGAGCCGGAATAGTAGGCACAGCCATAAGCCTATTAATCCGAACGGAGCTCAGCCAGCCAGGCCCCTTCATAGGAGATGACCAAATTTACAATGTTATTGTTACAGCACATGCCTTTATCATAATTTTCTTTATAGTTATACCAATTATGATCGGAGGATTTGGAAATTGACTACTCCCATTAATAATTGGAGCACCCGACATAGCATTCCCTCGCATAAACAACATAAGCTTCTGATTGCTACCCCCATCATTTACCCTACTTCTCTTTTCCGCCTTTATTGAAACCGGGGCTGGCACCGGATGAACAGTCTACCCACCGCTAGCTGGAAACCTAGCCCACGCCGGACCATCAGTAGACCTCACTATTTTCTCCCTTCACCTTGCTGGGGTGTCATCCATCCTTGGAGCAATTAACTTTATCACCACGGCTATCAACATAAAACCACCAGCAATGTCACAACAACAAACGCCCCTCTTTGTGTGATCTGTTCTAGTTACAGCTGTCCTCCTACTGCTCTCACTACCCGTCCTAGCTGCAGGAATTACTATACTACTAACTGACCGAAACTTGAACACCACTTTCTTTGACCCCGCAGGAGGAGGAGACCCAATCCTATACCAACACCTCTTCTGATTTTTCGGCCACCCAGAGGTATATATCCTTATCCTACCAGGGTTTGGAATAATCTCCCACGTAATTACCTTCTACTCAAGCAAAAAAGAACCATTTGGCTATATAGGAATAGTCTGAGCCATAATGTCAATCGGCTTCCTTGGCTTCATTGTCTGAGCCCACCATATGTTTACAGTAGGAATAGATGTTGACACTCGAGCATATTTCACATCCGCCACAATAATTATCGCCATCCCCACTGGTGTAAAAGTATTCAGTTGATTAGCCACTATTTACGGAGGAGTAGTAAAATGACAAGCCCCCATACTCTGAGCACTCGGCTTCATTTTCTTATTCACAGTCGGAGGACTTACAGGAATTGTACTAGCTAACTCGTCACTAGACATTATTCTCCACGATACCTACTACGTAGTAGCCCACTTCCACTATGTGCTATCTATAGGAGCAGTATTCGCCATCATAAGCGGATTCACCCACT-GATTCCCATTA-TTTACAGGATTCACCCTACACAGCACATGAACAAAAA-TTCAATTCATAATTATGTTTACAGGCGTAAACCTAACCTTCTTCCCACAGCACTTCCTAGGCCTATCAGGAATACCACGACGATATTCAGACTACCCGGATGCATATGCCTTCTGAAATATAATTTCCTCGATCGGGTCATTAATCTCCATAGTGTCAGTTATCCTACTCACATTTATTGTATGAGAAGCATTTTCATCAAAACGAAAAGTTCAAGTACCTGAAATAGCAAGCACAAATGTAGAATGACTAAATAATTGTCCACCATCATACCACACCTACGAAGAACCGGTCTTTGTTCAAGTACAGCCAAAACTAACGTAA------------------------------------ATGGCAAATCCAATACACCTAGGACTCCAAGATGCAATATCCCCATTAATAGAAGAACTCCTCTATTTTCATGACCACACACTAATAATTATTTTTCTAATCAGCATGTTTGTACTTTATACAATCTCAGTTCTACTGCTAACAAACCTATACCACACAAATGCAACAGACGTACAAGAAATAGAAATAATCTGAACCATTCTGCCAGCCCTAATCCTGATTACCATCGCCCTTCCATCTCTACGCACGCTATACCTCATAGACGAAACCACCAACCCCTGCCTAACCATTAAAGTCATCGGACATCAGTGATACTGAACATATGAATATACAGACTTTTCACAGCTAGAGTTCGACTCTTATATACTACCAACACAAGACCTACCCCAAGGTCATTTCCGCCTCTTAGAAGTAGACCACCGCATGATTGTCCCAACAAACTCAAGCACCCGAACATTAATCACAGCTGAAGACGTCCTACACTCATGAGCGGTACCCTCCTTAGGAATCAAAATAGACGCAGTGCCAGGACGACTAAATCAAACCTCACTAACATCCCCCAACCCTGGAGTTTTCTATGG-CCAATGCTCTGAAATCTGCGGGGCAAACCATAGTTTCATGCCTATTGTCGTAGAAGCTGTACCAATACAGCACTTCCAAAGCTGATTAAAAACAAACTCAT----AAATGACCCACCAAATACACCTATTTCACATAGTCAACCCAAGCCCGTGACCAATTTTAGGGGCTATGGCTGCCATAATACTAACAGCCGGATTAGTCCTATGATTCCACTGTAATATAAACCTAGTTTTACTCATAGGACTAATCTCAACCTTACTGATTATATTTCAATGATGACGAGACGTCGTCCGAGAAAGCACCTATTTAGGCCACCATACCCCGCCAGTCCAAAAAGGCCTACGATACGGTATAATCCTCTTTATCACCTCAGAAGTCTTCTTCTTCCTCGGGTTCTTTTGAGCGTTCTATCACTCAAGCTTGGCTCCAACCCCAGAACTGGGAGGACAATGACCGCCAACCGGAATTACCACACTAGACCCATTCGAAGTTCCACTCCTCAACACAGCAGTTCTACTAGCCTCTGGGGTTACAGTGACATGAGCCCACCACAGCCTAATGGAAGCCAACCGAGCACCCGCCATCCACGCCTTAACTCTCACAATTATTCTAGGGCTATACTTCACTGCTCTTCAAGCAATAGAATATTACGAAGCCCCATTCACCATCGCAGACAGCAGCTATGGCTCAACCTTCTTTGTTGCCACAGGCTTTCACGGCCTACATGTCATTATTGGCTCAACATTCCTAATGACCTGCCTATATCGACAAATCATACACCACTTCACCTCAAATCACCATTTCGGCTTCGAAGCCGCCGCTTGATATTGACATTTCGTAGATGTAGTCTGACTGTTCCTATACATCTCAATCTATTGATGAGGCTCCTATAAGTTTTTTAACAACCACGCCCATTCTTATCTACATCATCTCAGTCCTAATCGCAGTCGCATTCCTGACAGGACTAGAACGAAAAATCATTGGCTATATACCACTGCGCAAAGGCCCTAACATTGTCGGCCCTCTTGGGCTGCTACAGCCATTCGCCGACGGCCTCAAGCTTATTATCAAAGAGCTAACACTGCCCCTACTCGCCACCCCTGCTTTATTTGTTTTTTCCCCCGCAGTCGCCCTCATTTTATCCCTAATTATGTGGGCCCCCCTGCCCGTACCATTTTCTATCGCCAATCTAAACCTTGGCATGCTATTTTTATTAGCCATATCCAGCTTAGCAGTCTACTCACTACTGTGATCCGGATGAGCATCAAACTCTAAATACGCCCTAATAGGCGCCTTACGAGCGGTAGCCCAAACCATCTCCTACGAAGTCACATTAGCCATCATTGTTCTATCTATCGTCTTACTTAGCGGTGGATTTTCACTGCACGCACTAGCTGTAACCCAAGAGCCTATCTACTTGGCACTAACCACATGACCACTACTGATAATATGATACACCTCAACATTAGCAGAAACAAACCGCGCCCCATTTGACCTCACAGAAGGTGAATCAGAACTAGTATCTGGCTTCAACGTTGAGTACAGCGCAGGGCTGTTCACACTATTTTTTCTAGCTGAATACGCTAACATCCTACTAATAAACATTTTAACCACCATCCTCTTCCTAAACACAACAACAAACCTGCCAACACAAACACTATTCACCACCACCCTAATAAGTAAATCGATTCTACTGACCATCGGATTCCTATGAATCCGAGCATCATACCCACGATTCCGGTATGATCAATTAATACACCTGCTGTGAAAAAACTTCTTGCCAGCCACCCTGGCAATCTGCCTATGACACTCGTCGTTCCCTGTGTCAACATTCGGTCTTCCAGTAGCAA---GGATGCCCATCTTCCAACCCGTCATCTTAACCACACTAACTATTACAACACTCATTTTCCTGTCCTCCACCCATCTGGTGCTTATGTGGGTGGCACTAGAACTTAGCACATTAGTAGTTCTGCCATTAATTGCCAATAAGTCGCACCCACGATCCATTGAAGCCTCCACAAAATACTTCCTCACACAAGCCACCGCTTCTGCCCTAATCATCTTCTCATGGACCTTAAACTATATCACAACCGGGGGCGGCCAAATTACAGAGGTAACAAACCAAACACTTACAACCATTACAGCCCTAGCCCTGTTTATCAAAATTGGATTAGTGCCATTCCACTTCTGAGTGCCCGAAACCATTCAAGGAATAACCCCAACCGCCTCCATCTTCCTACTTACCTGACAAAAACTAGGCCCACTAATTATATTATACCTAATGAGCCCACTAATTAATTTCGAAGTCCTCTCTGCAGTATCTATCCTATCCGCCACAGTTGCCGGCTGGCTTGGACTTAATCAAACCCAAATCCGAAAACTAGTGGCATTCTCTTCAATCGCCCAAATAGCCTGGACCCTAGTGATTATTAAATACGCACCATCACTTACAATCCTAGCCTTCTACCTGTATTCAATCACAATCTCCACCACGCTTCTCACACTAGAAAAACTATCAACAACATCCATTAACAACCTCCTACTTTCGTTCCAAAAAGCCCCAATTACTTCCTTACTACTAATGGTCTCTTTATTATCCCTATCAGGACTACCCCCACTAGCCGGCTTCCTACCAAAATGACTAACAATCAACCAGCTCGTAGCAGAGGGGGCAATTTGAGTCGCATTCACAATACTCATGGCCTCTCTTCTAAGCCTTTTCTTTTACCTCCGGCTATGATACAATTCCGCATCCACCCTTCCCCCAAACACTACTAATACCCAACGACTATGACGCAAACCAGTTCAACAAACCAACCTCACAATCAACTCCCTAGCTATAGCCGCCCTCACCCTAATCCTAGCAGCCACCATAATAAAAGCCACTACAAAACAAGAG------ACCCATTAAA----TAAACCTTCTCACTGCATTCATATTAGCCACAGCCACTGCAGTAGCCGTAATTACCCTAAACCTACTGATATCGGAAATGGCCCCAGACCCCGAAAAACTCTCACCATACGAGTGCGGATTCGACCCCCTAGGGTCCGCCCGCCTGCCACTATCTATCCGCTTTTTCATGGTCGCTATCTTATTCCTACTATTTGACCTAGAAATCGCCATCCTACTACCACTAGCATGGGCCCTTCAACTCACAAACCTGATCAAAAGTGTCACATGGGCCATCATTGTCTTCTTACTCATATTTGCAGGTCTAACATACGAATGATTACAGGGCGGACTAGAATGGGCAGAATAGATGTTAAAAATCATTGTACCAACAATAATACTAATCCCATCAACCTGCCTAACAACCACAAAAAACACCTGACTATCACCTACAGCCTACTCAGCAGTTATTATTATCCTAGGCATACTTGTCTTAAACCCCGGGGATATTCTGATAAATACTAGCGGTTTATTACTAGGAAGCGACCAAATCTCAACACCCCTACTTATATTATCCTGCTGACTACTACCATTAATATTTATAGCCAGCCAAAGTTCCATGTCACACGACCCAACTCAACAAAAACGACTATTTATTACAGCCCTAGCCCTCCTGCAATTAGCCCTAATATTAGTATTCATGGCCTTAGACCTAATATTATTTTACACCGCCTTTGAAGCAACCCTCATTCCCACCCTAATAGTAATCGCCCGATGGGGCTCCCAAACAGAACGGCTCGGAGCCGGACTATACTTCCTCCTATACACCATCACTAGCTCCATACCGCTCCTAATTGCACTTCTATGGGTATATAACATAAAAGGAACTACATCCATCACACTCTTACAACTATTACCCCAAATAACCCTAATATTCTGAACAAGCACATTACTATGAGTCTCCCTTATATTAGCCTTCCTAGTAAAAATCCCAATTTACGGCCTTCACCTCTGGCTACCAAAAGCCCACGTAGAAGCCCCCATTGCCGGATCCATGGTCCTTGCAGCGATCCTGTTAAAACTTGGGGGCTATGGCCTGCTACGAATTACAAATACACTAACTGAACAAGCCACATTCTCCTACATCCTACCCCTAACAGTAGCACTATGAGGTGCACTCATAACTGGCATAATCTGCTTACGACAAACAGACTTAAAATCCCTAATTGCCTACTCCTCAGTAAGCCACATAGGATTAATAACAAGCTCAATCCTTACTCGCAATCAACTAGCCCCCTCAGGCTCAATAATTATAATAGTAGCTCACGGCCTTACATCCTCAATACTATTCTGCTTGGCAAATATTACTTACGAACGAACACACTCACGAACCCTGCTACTAACGCAAGGAGTACAACTCACCACCCCAATCATGACATCCTGGTGGCTTCTGGCCTGCTTAACAAACATAGCACTCCCCCCAACAATTAACTTTATTGGAGAGCTCACCCTTATAGTATCACTATTTGACTGAGCGGACATTACTATTTTTCTAACAGGACTAAGCGCATTCATCACCTCGATTTACACCCTACACACATTCTCCTCCACCCAACAAGGAACCCTACCAAACCACATTATTACGATAAGCCCAACCCAAACGCGAGAACATCTACTAATAGCACTACACTCTGCACCATCAGTTGCTTTAATTATTGTCCCCCAACTGATGTATTACCAATAA---ACCTCCCCCACTAATCTACTGTTCACCATTTCTTTCATCATCTTCACCATTGGGTTCACCTTCCGTCACACTCATCTCCTCTCAGCCCTATTATGCCTGGAAGGCATGATGTTGTCGGTATTTCTATTAATGGCAACATGATCTATAAACTCAAACATCTCATCGTTCATCCTGCCTTTAACAGTCCTAACACTATCAGCCTGTGAAGCCGGCATTGGCCTCGCCCTACTGATCGCCTCAGCCCGAACACATAATACAGCCAACCTCAAAAACCTAAACCTACTCCAATGTTAAAT-AACACAGCCCTCAGCCTTAATTATTATACTTTTCCTGCTACCCCTAGCAATCTTAACACTCCCAATACTAGCCCCAAGCT-CAAAATTGACCTCG--CCACTTAGCACCAAAGTATTGATAGCAAAACTAGCCTTCCTTACTAGCCTAATCCCACTGGCCTATCTCATCTACAATGACCTAACCATTACTACATACGAAACCCGATGATCAGAAATTAGCACAACCACAATTCGCATTAGCTTCACGCTAGACATTTACTCCACCTTCTTCTTACCAATCCTCCTTTTTGTCGTATGGTCCATCATAGAATTTACAGTACAATACATGGAGTCAGACATAAAAATTAACACTTTCTTTAACCAACTCACCACCTTCACCCTAATAATAATAATTCTTGTGACCGCTGAAAACCTCTTCCAATTTTTCATCGGCTGAGAAGGAGTTGGCATCATGTCCTTTATGCTAATTAACTGGTGATCATTCCGATCAAGCTCGAACAAAGCGGCCATGCAAGCCGTAATCTATAACCGCTTAGCAGACATTGGCCTAGTTATCACCCTGGCATGAATAGTCATCAACAACCTGTCCCTAAGTATTAAAAGCATACAAATCACACCAGACATAGCCCTCATCCCCGCACTCGGGCTGCTTCTAGCAGCGGCCGGAAAGTCTGCTCAATTCGGATTCCACCCATGACTCCCCGCAGCAATAGAGGGTCCAACCCCCGTCTCGGCCTTACTGCACTCAAGCACTATAGTAGTAGCCGGAGTATTCCTACTCATCCGAACCTCAGACCTCCTATACAGCAGCGAAACAGCAACTACAGCCTGCCTACTACTGGGGGCCTTTACATCCATACTAGCCGCCTCCTGCGCACTAACCCAAAATGACCTAAAAAAAATTATTGCCTACTCAACTACTAGCCAGCTAGGCCTAATAATGACCTCCATCGGGTTAAAACAACCTGAGCTCGCATTCATACACATCTCAACACACGCATTCTTTAAAGCAATGCTATTCCTATGCGCAGGGACAATTATCCACAGCCTAGACAACGAACAAGACATCCGAAAGATGGGGGGACTCAAAAAAGCGCTTCCTACTACATCCTCCTGCTTAATTATTGGCTCCCTTGCCCTCTCAGGAATACCATTCATGGCTGGCTTTTACTCAAAAGACGCCATCATCGAGTCAATCAACACCTCCAACGTAAACTCCCTATCACTAGCCATGACCCTAGTGGCAACCACCTTCACCACACTTTACAGCCTGCGTATGATCTACTATGTGGCCCTAAGCACCCCACGAATCCTACCACTATCCGCCATCTCCGAAACCCATCAGACAACCAACCCAATTCTACGGCTGGCCATTGGAAGCATCGCAGCCGGACTCATAATTTCAACCACTATTTTACCGCCAAATATTCCACAACTGACCATACCAACATCAACCAAGCTAACCGCACTAAGTATCCTAGTCCTAGGATTACTAGTCGGCTCAATCCTAATCACAGTAGCAGACCAACTTCCCAGCTCCACCAAGGGCACCCAAAACCCCCTTATCTCTAAGATCATCCACTCTTACTTCATTCTGCACCACACCCTATCATCCATAGTCTTACAAATTAGCCAAAAATTATCAACCCACCTAATAGACCAAACACACTATGAAGCCTTAGGCCCTAAAACAATAACCTATCTACAAATACTAATAGCCAAACTATTAACTAGCCTCCATAAAGCCCGAATTAACCCGTACTTAAAAATCATTATCCTATCCATCATATTAATCTCCCTATTCTACTTCACCTCGATGAACGCAGGGCCCCTCGATGCCGCCCACGAACTAGAAT-----ATGGGAATTACATTTTTTCTTATTTGTTGTTTAATGATAATTAGTGTGGTGTTAGTGGCGTCTGGGGTGACGATCCACTATGGGGTGGTTAGTTTGCTTTTTGCTGCAATATTTGGTAGTGCATTGTTAGTAGTAGGGGGTGGAAGTTTTATGCCGCTTGTGGTACTACTAATTTATTTGGGCGGCTTGTTGGTGGTTTTTGCTTTTTGTGTGGGGTTCACTGATGATAAATATTGTGAGTTCTGGGGAGCAGGGGGGTCTAAGGTGTCGGCTTGTGTTTGTGGGGTTGGCTTAGGCGTTGGTGGATATTATATATATGACTCTGCGTGA----GCTGAGGTTTTAGGGTGTTTTG--TTGATGCCGTTGAGACCTGGAGTGGTGATGTTAGTAATGAGTTTTTAGGGGTTGGGCTGTTTTATATGAGTGGCTGGGGGTTTCTCATTTTAAGTGGCTGAGCCTTATTGGTGGTATTGTTTACTATTATGATTCTAGTTCGTGGGCGGCATCGAGGGGCCCTGCGTTCATCGAGG

*Crocodylus moreletii*

ATGACCCTGACCACAAATCTAAACCTATTTGACCAATTCTTAGTGCCTAAACTACTTGGCATATCACTGTTAATCCCAGCCATATTGCTAACAACACTGTTAATTTATAACCCGCAAGACCGCTGACTGTCGAACCCTTTAACAACCCTACAAGCCTGACTAATTGCAAAAACCACTAAACAAATCATAATCCCAGTAAACAAGCCAGGACATAAGTGATCATTAATACTGATCTCATTACTAACAATGCTCATCCTCAACAACCTTTTAGGCCTTCTCCCATATACATTTACACCAACAACTCAACTGTCTATAAACATAGCCCTGGCCCTGCCACTATGATTGGCAACAGTACTAATTGGCCTACGAAACAAACCGGCTTCTTCTCTAGCCCACCTCCTGCCAGAAGGGACCCCGACACCCCTCATCCCAATCTTAATCTTAATCGAGACAATCAGCCTGCTCATCCGACCAATCGCGCTCGCCGTGCGACTTACAGCCAACCTCACCGCAGGACATCTTTTGATACACCTAATCTCTACTGCAGCACTCAACCTAATGACAACCTCCACGCTACTTGCAGGACTCACCCTATTCATCCTAGCCCTGTTAATACTCCTAGAAATTGCAGTAGCAATGATCCAAGCATATGTATTTACCCTACTACTCTCATTGTACCTGCAAGAAAACGTATAAATGCCGCAATTAAACCCAGAACCTTGATTAATGATCTCATCTATTACATGGCTAGTATTCATTATTACTTTACAGCCAAAAATTGCTTCTATGAAATTCATAAATAACCCAAGCAACCCAAACCAAAAAACCACT-AAAACATGACCCTGACCACAAATCTAA---------------------ATGGCCCACCAACTACGAAAATCCCACCCACTTTTAAAACTAGTAAACCACTCTTTAATTGACTTACCCACGCCATCCAACATCTCCTACTGATGAAACTTTGGATCACTTCTTGGGTTCACCCTATTAATCCAACTAGCATCTGGCATCCTACTAATAATGCACTTCCTAGCAGATGACTCCCTAGCTTTTATATCTGTTGCTTATACCTCACGAGAAGTTTGATATGGTTGACTAATCCGAAGCCTCCATGCAAACGGAGCCTCCCTGTTCTTCCTATGTATTTTCCTACACATTGGACGCGGAGTATACTACGGATCATACCTCAATGAAAACACATGAAACATCGGAGTACTGCTACTATTACTACTGATAGCAACTGCCTTCATAGGCTACGTCTTACCGTGAGGACAAATATCATTCTGAGGGGCAACCGTAATCACCAACCTAATATCAGCCATTCCATACGTAGGAGACACAATTGTAACCTGAATTTGAGGGGGGCCATCAGTCAACAGCGCAACCCTAACACGATTCACCACCTTACATTTCCTACTCCCATTTATCCTCATAGCCGCAGTCATCACACACCTCATCTTCCTCCACGAACGCGGATCATTCAACCCACTTGGACTAGCCTCCAACGCCGACAAAATTCCGTTCCACCCATACTTCTCAGCAAAAGACGCCATAGGCATAGCTATAGCCGCCGTCCTATTAACAACCCTTACATTCTACTTTCCAAACCTATTAGGAGACCCAGAAAACTTCACCCCTGCCGACCCCATAAAAACACCAGACCACATTAAACCAGAATGATACTTCCTATTTGCCTACACAATCCTACGATCTATCCCAAACAAGCTCACAGGAGTCCTTGCCATATTCGCATCCATCCTAGTACTATTACTTCTACCTGCACTACACACATCAAAACGGCAATCAATAAGCCTACGCCCCCTATCACAACTCCTATTCTGGGCCCTAATCGCAGACTTCTTTATTCTTACATGAATCGGAGGACAACCAGTACAAGACCCATACATCTTAATTGGACAAATGGCCTCCTTCATCTACTTCTTCACCATCCTTATCTTATTGCCACTGGCTGGAATAATTGAAAACCTATTAATCAAACCCCTTCGATACCGACCCT----------GTGAATATTAATCGTTGACTTTTTTCCACTAACCACAAAGATATCGGCACCTTGTATTTTATTTTCGGCGCCTGAGCCGGAATAGTAGGCACAGCCATAAGCCTATTAATCCGAACAGAGCTCAGCCAGCCAGGTCCCTTCATAGGAGATGACCAAATTTATAATGTTATTGTTACAGCACATGCCTTTATCATAATTTTCTTTATAGTTATACCAATTATGATCGGGGGATTTGGAAATTGACTACTCCCATTAATAATTGGGGCACCCGACATAGCATTCCCTCGCATAAACAACATAAGCTTCTGATTGCTGCCCCCATCATTTACCCTACTTCTCTTTTCCGCCTTTATTGAAACTGGGGCTGGCACCGGATGAACAGTCTACCCACCACTAGCTGGAAACCTAGCCCACGCCGGACCGTCAGTAGACCTCACTATCTTCTCCCTTCACCTTGCTGGGGTATCATCCATCCTTGGAGCAATCAACTTTATTACCACAGCTATCAACATAAAACCCCCAGCAATGTCACAACAACAAACACCCCTTTTTGTATGGTCTGTTCTAGTTACAGCTGTTCTCCTACTGCTCTCACTACCCGTCCTAGCTGCAGGAATTACCATATTACTTACTGACCGAAACTTGAACACCACTTTCTTTGACCCGGCAGGAGGAGGAGACCCAATCCTATACCAACACCTTTTCTGATTTTTCGGCCACCCAGAAGTATACATCCTCATCCTCCCAGGGTTTGGAATAATCTCCCACGTAATTACCTTCTACTCAAGCAAAAAAGAACCATTTGGTTATATAGGAATARTCTGGGCCATAATGTCAATCGGCTTCCTTGGCTTCATTGTCTGAGCCCACCATATGTTTACAGTAGGAATAGATGTTGATACTCGAGCATATTTCACATCCGCCACAATAATTATCGCCATCCCCACTGGTGTAAAAGTATTCAGCTGATTAGCCACTATTTACGGGGGAGTAGTAAAATGACAAGCCCCCATGCTCTGAGCACTCGGCTTCATTTTCTTATTCACAGTTGGAGGACTAACAGGAATTGTACTAGCTAACTCGTCACTAGACATTATTCTCCACGATACCTACTATGTAGTAGCCCACTTCCACTATGTATTATCTATAGGAGCAGTGTTCGCCATCATAAGCGGGTTCACCCACT-GATTCCCATTA-TTTACAGGATTCACCCTACACAGCACATGAACAAAAA-TTCAATTCATAATCATATTTACAGGTGTAAACCTAACCTTCTTCCCACAGCACTTCCTAGGCCTATCAGGAATGCCACGACGATATTCAGACTACCCAGATGCATATGCCTKCTGAAATATAATTTCCYCAATTGGGTCATTAAYCTCCATAGTATCAGTTATCCTACTCACATTTATTGTATGAGAAGCATTTTCATCAAAACGAAAAGTTCAAGTACCTRAAATGGCAAGCACAAATGTAGAATGACTAAACAATTGTCCACCATCATACCACACCTACGAAGAACCAGTCTTTGTTCAAGTACAACCAAAACTAAAGTAA------------------------------------ATGGCAAACCCAATACACCTAGGACTCCAAGATGCAATATCCCCATTAATAGAGGAACTCCTCTATTTTCATGACCATACACTAATAATTATTTTTCTAATCAGCATGTTTGTACTCTATACGATCTCAGTTTTATTGCTAACAAACCTGTACCACACAAACGCAACAGACGTACAAGAAATAGAGATAATCTGAACCATTCTACCAGCCCTAATCCTAATTACCATCGCCCTTCCATCTCTACGCACACTATACCTCATAGACGAAACCACCAACCCCTGCCTAACCATTAAAGTCATTGGACATCAATGGTATTGAACATATGAATATACAGACTTTTCACAGCTAGAGTTCGACTCTTACATACTACCAACACAAGACCTACCTCAGGGTCACTTCCGCCTATTAGAAGTAGACCACCGCATGATTGTCCCCACAAACTCAAGCACTCGAACATTAATCACAGCTGAAGACGTCCTACACTCATGAGCAGTACCATCCTTAGGAATCAAAATAGACGCGGTGCCGGGACGACTAAATCAAACCTCACTAACATCTCCCAACCCCGGAATTTTCTATGG-CCAATGCTCTGAAATCTGCGGGGCAAACCATAGTTTTATGCCTATTGTCGTAGAAGCTGTACCAATACAGCACTTCCAAAGCTGATTAAAAACAAACTCAT----AAATGACACACCAAATACACCTATTCCACATAGTCAACCCAAGCCCATGACCAATTTTAGGGGCTATAGCTGCCATAATACTAACAGCCGGATTAGTCCTATGATTCCACTGTAATCTAAGCCTAATTTTACTCATAGGACTAATCTCAACCTTACTGATTATATTTCAATGATGACGAGACGTTGTCCGAGAAAGCACCTATTTAGGCCACCATACCCCTCCAGTCCAAAAAGGCCTACGATATGGTATAATCCTCTTTATCACCTCAGAAGTCTTCTTTTTCCTCGGGTTCTTCTGGGCGTTCTATCACTCAAGCCTGGCTCCAACCCCTGAACTAGGTGGACAGTGGCCACCAACTGGAGTTACCACACTAGACCCATTCGAAGTTCCACTCCTCAACACAGCAGTACTACTAGCCTCAGGGGTTACAGTAACATGAGCCCACCACAGCCTAATAGAAGCCAACCGAACACCCGCCATCCACGCCTTAACTCTTACAATTATTCTAGGGCTATACTTCACTGCCCTTCAAGCAATAGAGTATTACGAAGCCCCATTCACCATCGCAGACAGCAGCTATGGCTCAACCTTCTTTATTGCCACAGGCTTTCACGGCCTACATGTCATTATTGGCTCAACATTCCTAATAACCTGCCTCTATCGACAAATCATACACCACTTCACCTCAAATCACCACTTCGGTTTCGAAGCCGCCGCCTGATACTGACACTTCGTAGATGTAGTCTGACTGTTCCTATACATCTCAATCTATTGATGAGGCTCCTATAAGTTTTTTAACAATCACGCCCATTATTATCTACATCATCTCAGTCCTAATCGCAGTTGCATTCCTAACGGGACTAGAACGAAAAATCATTGGCTATATACAGCTACGTAAAGGCCCTAACATTGTCGGCCCCCTTGGACTGCTGCAGCCATTCGCCGACGGCCTCAAACTTATTATCAAAGAGCTAACACTGCCCCTACTCGCCACCCCTGCTTTATTTGTCTTATCCCCAGCAGTCGCCCTCATTTTATCCCTAATTATATGAGCCCCCCTGCCCGTACCATTTTCTATCGCCAATCTAAACCTTGGCATACTATTTTTATTAGCCATATCTAGCTTAGCAGTCTACTCACTACTGTGATCCGGATGAGCATCAAACTCTAAATACGCCCTAATAGGCGCCTTACGAGCAGTAGCCCAAACCATCTCCTACGAGGTCACATTAGCCATCATTGTTCTATCTGTTGTCTTACTTAGCGGCGGGTTTTCACTACACGCACTAGCTGTGACCCAAGAACCCGTCTACCTGGCACTAACCACATGACCACTACTGATAATATGATATACCTCAACATTAGCAGAAACAAACCGCGCCCCGTTTGACCTCACAGAAGGTGAATCAGAACTAGTATCTGGCTTCAACGTTGAGTACAGCGCAGGACTATTCACACTATTTTTCCTAGCTGAATACGCTAACATCCTACTAATAAACATTTTAACCACCATCTTGTTCCTAAACACAACAACAAACCTGCCAACACAAACATTATTCACCACCGTCCTAATAAGTAAATCCATTCTACTGACCATCGGGTTCCTATGAATCCGAGCATCATACCCGCGATTCCGGTATGATCAATTAATACACCTACTGTGAAAAAACTTCTTGCCAGCCACGCTGGCAATCTGCCTGTGACACTCGTCGTTCCCAGTGTCAATATTCGGTCTTCCAGTAGCAA---GGATGCCCATCTTCCAACCCATCATCTTAACTACACTAACTATTACGACACTCATTTTCCTATCCTCCACCCAGCTGGTGCTCATATGGGTAGCACTAGAACTTAGCACACTAGTAATTCTGCCATTAATTGCCAATAAATCGCACCCACGATCCATTGAAGCCTCCACAAAATACTTCCTCACACAGGCCACCGCTTCTGCCTTAATCATCTTCTCATGGACCTTAAACTATATCACAACCGGGGGCGGCCAAATTACAGAAATAACAAACCAGACACTTACAACCATTACGGCCCTAGCCTTGTTTATTAAAATTGGATTAGTGCCATTCCACTTCTGAGTGCCCGAAACCATCCAAGGAATAACCCCAACCGCCTCCATCTTCCTACTTACCTGACAAAAACTAGGCCCACTAGTTATATTATACCTAATGAGCCCACTAATTAATTTCGAGGTCCTCTCTGCAGTATCTATCCTATCCGCCACAATTGCTGGCTGACTTGGACTTAACCAAACCCAAATCCGAAAACTAGTAGCATTCTCTTCGATCGCCCAAATAGCCTGAACCCTGGTGATTATTAAATACGCACCACCACTTACAATCCTAGCCTTCTATTTGTATTCAATCACAATTTCCACCACGCTTCTCACACTAGAAAAACTATCAACAACATCCATTAACAACCTCCTACTTTCATTCCAAAAAGCCCCAATTACTTCCTTACTACTAATGGTCTCTTTATTATCCCTATCAGGACTACCCCCACTAGCCGGCTTCCTACCAAAATGACTAACAATTGACCAACTAGTAGCAGAGGGGGCAATTTGAGTCGCATTCACAATACTCATAGCCTCTCTTCTAAGCCTTTTCTTTTACCTCCGACTATGATACAACTCCGCATCCACCCTTCCCCCCAACACTGCTAATACCCAACGACTATGGCGCAAACCAGTTCAACAAACAAGCCTCACAATCAACTCCCTAGCTATAGCCGCCCTCACCCTAATCCTAGCGGCCACCATAATAAAAGCCATTACAAAACAAGAG------GCCTATTAAA----TAAACCTACTTACTGCATTCATATTAGCCACAGCCACTGCAGTAGCCGTAATCACCCTAAACCTACTGATATCAGAAATGACCCCAGACCCCGAAAAACTCTCACCATATGAGTGCGGATTCGACCCACTAGGATCCGCCCGCCTGCCACTATCTATCCGCTTTTTCATGGTCGCTATCTTATTCCTACTATTTGACCTAGAGATCGCCATCCTGCTACCACTAGCATGAGCCCTTCAACTCACAAGCCTGATCAAAAGCATCACATGGGCCATCATTATCTTCTTACTTATATTTGCAGGTCTAACATACGAATGACTACAAGGCGGACTAGAATGAGCAGAGTAGATGTTAAAAATCATTGTACCTACAATAATACTAATCCCCTCAGCCTGCCTAACAACCACAAAAAACACCTGACTATCACCAACAGCCTATTCAACGATTATTATTATCCTAGGCATGCTTGTCTTAAACCCCGGAGACATCCTAGTAAATACTAGTGGTTTGTTGCTAGGAAGCGACCAAATTTCAACACCCCTACTTATATTATCCTGCTGACTACTGCCACTAATATTTATAGCCAGCCAAAGCTCCATGTCACACGACCCAACCCAACAAAAACGACTATTTATTACGGCCCTAGCCCTCCTACAATCAGCCCTAATATTAGTATTCATGGCCCTAGACCTAATATTATTTTACATCGCCTTTGAAGCAACCCTCATTCCCACCCTAATAGTAATCGCCCGATGAGGCTCCCAGACAGAACGGCTCGGGGCCGGTCTATACTTCCTCCTATACACCATCACTAGCTCTATACCGCTTCTAATTGCGCTTCTATGAGTCTACAACATAAAAGGAACTACATCTATTACACTCTTACAACTATTGCCCCAGATAACCCTAACATTCTGAACAAGCACGTTACTGTGAACCTCCCTTATATTAGCCTTCCTAGTAAAAATCCCAATTTACGGCCTTCACCTCTGGCTACCAAAAGCCCACGTAGAGGCCCCCATTGCCGGATCCATGGTCCTTGCAGCGATCCTATTAAAACTCGGGGGCTATGGTCTGCTACGAGTTACGAACCCACTAACTGAACAAACCACATCCTTTTATATCCTACCCCTAACAGTAGCACTATGAGGTGCACTCATAACCGGCATAATCTGCTTACGACAAACAGACTTAAAATCCCTAATTGCCTACTCCTCAGTGAGCCACATGGGATTAATAACAAGCTCGATCCTTACTCGCAATCAACTAGCCCCTTCAGGCTCAATAATCATAATAGCAGCCCACGGCCTTACATCCTCAATACTATTCTGCTTGGCAAATATTACTTACGAACGAACACACTCACGAACCCTCCTACTAACGCAAGGAGTACAACTCACCACCCCAGTCATGTCATCCTGATGGCTTCTGGCCTGCTTAACAAACATAGCACTCCCCCCAACAATCAACTTTATTGGAGAACTCACCCTTRTAGTTTCACTATTTGACTGAGCGGACATTACTATCTTCCTAACAGGACTAAGCGCATTCATCGCCTCGATTTACACCCTACACATATTCTCCTCCACCCAGCAAGGAACCCTCCCAAGCCACATTATCACGATAAGCCCAACCCAAACGCGAGAACATCTACTAATAACACTACACTCTGCACCATCAGTTGCTTTAATTATTGTCCCTCAACTAATGTATTACCAATAA---ACCTCCCCCACTAACCTACTGTTCACCCTTTCCTTCATCATCTTCACCATTGGGTTTACCTTCCGCCACACTCATCTCCTCTCAGCCCTATTATGCCTGGAGGGCATGATATTATCGGTATTTCTATTAATGGCAACATGGTCTCTAAACTCAAACATCTCATCCTTCATCCTGCCTTTAACAGTCCTAACATTATCAGCATGTGAAGCTGGCATTGGCCTTGCCCTACTGATTGCCTCAGCCCGAACACATAATACAGCCAACCTCAAAAACCTAAACCTACTCCAATGTTAAAT-AACACAACCCTCAGCCTTAATTATTATACTTTTCCTACTACCCCTAGCAATCTTAACACTCCCAATACTAATCCCAAACT-CAAAATTAACTTCA--CCACTCAACACCAAAGTATTGACAGCAAAACTAGCCTTCCTTACTAGCCTAATCCCACTGACCTATCTCATCTATAATGACTTAACCATTACTACATACGAAACCCAATGATCAACAATCAGCACAACCACAATTCGCATTAGCTTCACGCTAGACATTTACTCCACCTTCTTCTTACCCATCCTTCTTTTTGTTGTATGATCTATCATAGAATTCACAGTACAATACATAGACTCAGACATAAAGATTAACACTTTCTTTAACCAACTCACCACCTTCACCCTAATAATAATAATTCTTGTAACTGCCGAAAACCTCTTCCAATTTTTCATTGGCTGAGAAGGAGTGGGCATCATATCCTTTATACTAATTAACTGGTGATCATTCCGATCAAACTCGAATAAAGCTGCCATGCAAGCCGTAATTTATAACCGCTTAGCAGACATTGGCCTAGTTATCACCCTGGCATGAATAGTCATTAACAACCTGTCCCTAAGCATTAAAGGCATACAAATCACACCAGACATGGCCCTCATCCCCGCACTCGGATTGCTTCTAGCAGCTGCTGGAAAATCTGCTCAATTCGGATTCCACCCATGACTCCCAGCAGCAATAGAAGGTCCAACCCCCGTCTCAGCCTTACTGCACTCAAGCACTATGGTAGTAGCCGGAGTATTCCTACTCATCCGAGCCTCAGACCTCCTATACAGCAGCGAAACAGCAACTACAACCTGCTTACTACTGGGGGCCTTTACATCCATACTGGCCGCCTCCTGTGCACTAACCCAAAATGACCTAAAAAAAATTATTGCCTACTCAACTACTAGCCAGCTAGGCCTAATAATGACCTCCATCGGGTTAAAACAACCTGAACTCGCATTTATACACATCTCAACACACGCATTCTTTAAAGCAATACTGTTTCTATGCGCAGGAACAATTATCCACAGCCTAGACAACGAACAAGACATCCGAAAGATGGGGGGACTCAAAAAAGCACTTCCTACTACATCCTCCTGCTTAATTATTGGTTCCCTTGCCCTCTCAGGAATACCATTTATAGCTGGCTTTTACTCAAAAGACGCCATCATTGAGTCAATCAACACCTCCAACGTAAACTCCCTGTCACTAGCCATAACCCTAGTAGCAACCACCTTCACTACACTTTACAGCCTGCGCATGATCTACTATGTAGCCCTAAGCACCCCACGAATCCTGTCACTATCCGCCATCTCCGAAACTCCTCAGACAACCAACCCAATTCTACGGCTAGCCATTGGAAGCATCACGGCCGGACTCATAATTTCAACCACTATTCTGCCACCAAATACGCCACAACTAACCATACCAACATCAGCCAAGCTAGCCGCACTAAGTATCCTAATCCTAGGACTACTAGTCGGCTCAATCCTAATTACAGTAGCAGACCAACTTCCCAACTCCACCAAAGGCACCCAAAACCCCCTTATCTCTAAAATCATCCACTCTTACTTCATTCTGCACCACACCATATCATCCCTGGTCTTACAAATTAGCCAAAAATTATCAACCCACCTGATAGACCAAACACACTATGAAGCCTTAGGCCCTAAAACAATAACCTACCTACAAATACTAATAGCCAAGCTATTAACCAACCTCCATAAAGCCCGAATTAACCCGTACTTAAAAATCATTATCCTATCCGTCATACTAGTCTCCCTGTTCTACTTCACCTCAATGAACGCAGAGCCCCTCGATGCCGCCCACGAACTAGAATCATAAATGGGAATTACATTTTTTCTTCTTTGTTGCTTAATGATAATCAGTGTGGTGTTAGTGGCGTCTGGGGTGACGATCCACTATGGGGTGGTTAGTTTGCTTTTTGCTGCAATATTTGGCAGCGCATTATTAGTAGTAGGGGGTGGAAGTTTTATGCCGCTTGTGGTACTACTAATTTATTTGGGCGGCTTGTTGGTGGTTTTTGCTTTTTGTGTGGGATTCACTGATGATAAATATTGTGAGTTCTGGGGAGCAGGGGGGTCTAAGGTGTCGGCCTGTGTTTGTGGGGTTGGCTTAGGCATTGGTGGGTATTACATGTATGAGTCTGCATGA----GCTGAGGTTTTAGGGTGTTTTG--TTGATGTCGTTGAGACTTGGGGTGGTGATGTGAGTAATGAGTTTTTAGGAGTTGGGCTGTTTTATATGAGTGGCTGGGGGTTCCTCATTTTAAGCGGTTGGGCTCTATTGGTGGTGTTGTTTACCATTATGATTCTAGTTCGTGGGCGGCATCGAGGGGCTCTGCGTTCATTGAGG

*Crocodylus johnstoni*

ATGACCATGGCCACAAATTTAAACTTATTTGACCAATTCTTAATGCCTAAATTACTCGGCATGTCCTTATTAATTCCAGCTATATTATTAACAACAACCCTAATCTATAACCCACAAGACCGCTGACTATCGAACCCCTCAACAACCTTACAGTCCTGATTAATTGCAAAAGCCACTAAACAAATCATAACCCCAGTAAACAAACCAGGTCACAACTGAGCATTAATGCTGATATCACTACTAACAATACTTCTTCTCAACAACCTTCTAGGCCTTCTCCCGTATACATTCACACCAACAACTCAGCTGTCCATAAACATAGCCCTGGCCCTACCACTATGACTGGCAACTGTACTAGTTGGCCTACGAAACAAACCAGCCTCTTCACTAGCCCACCTCCTGCCAGAAGGGACCCCAACACCCCTAATCCCAATCTTAATCCTAATCGAAACAATCAGCCTGCTTATCCGACCAATCGCACTTGCCGTGCGACTCACAGCCAACCTCACCGCAGGACACCTCTTGATGCATCTAATCTCCACTACGGCACTTAACCTAATGACAACCTCCACACTACTTGCCGGACTCACCCTAGCCATCCTAACCCCGCTAATACTCCTAGAAATCGCAGTAGCAATAATCCAAGCATATGTGTTCACCCTACTACTATCACTATACCTGCAAGAAAACGTATAAATGCCACAATTAAACCCAGAACCTTGACTAATAATCTCATCTATCACATGGCTAGTATTCATTCTCGCTTTACAGCCAAAAATTGCTTCCCTAAAATTCATAAATAACCCAAGCAGCCCAGACCAAAAAACCACT-AAAACATGACCATGGCCACAAATTTAA---------------------ATGACCCACCAACTACGAAAATCCCACCCGCTCTTAAAATTAATAAACCACTCTTTGATCGACTTGCCCACACCATCCAACATCTCCTACTGATGAAACTTTGGATCACTTCTTGGGTTCACCCTACTAATCCAACTAGCATCGGGCATCCTACTAATAATACACTTCCTAGCAGATGACTCTTTAGCTTTTATATCCGTCGCCTACACCTCACGAGAAGTCTGATACGGCTGGCTGATCCGAAGCCTCCATGCAAACGGGGCCTCACTATTCTTCCTGTGCATTTTCCTACACATCGGGCGCGGACTATACTACGGATCATACCTCAACGAAAACACATGAAACATCGGAGTACTACTACTACTATTACTGATAGCAACTGCCTTCATAGGCTACGTCCTACCATGAGGGCAAATATCATTCTGAGGGGCAACCGTAATCACCAACCTGATATCGGCCATCCCATATGTGGGAGACTCAATTGTGACCTGAATTTGAGGAGGACCATCCGTCAATAGCGCAACCCTAACACGATTTACTACCCTACACTTCCTGCTCCCATTTATCCTCACAGCCGCTGTCATCACACACCTTATCTTCCTCCACGAACGCGGATCATTCAACCCATTAGGACTGGTCTCCAACGCCGACAAAGTTCCGTTCCACCCATATTTCTCAGCAAAAGACGCTATAGGCATAGCCCTGGCCACCACCCTACTAATAACCCTCACATTCTACTTCCCAAACCTACTAGGAGACCCAGAAAACTTCACCCCAGCCGACCCCATAAAGACACCAGACCACATTAAACCAGAATGATACTTCCTATTCGCCTACACAATCTTACGGTCTATCCCAGACAAGCTTACGGGGGTCCTTGCCATATTCGCATCCATCCTAGTATTATTGCTCCTGCCTGCACTACACACATCAAAACGACAATCAATAGGCCTACGCCCCCTATCCCAGCTTCTATTCTGAACCTTAGTCGCAGACTTCTTTATTCTCACATGAGTCGGAGGACAGCCAGTACAAGACCCATACATCCTAATTGGACAAATAGCCTCATTCATCTATTTCTTCAACATCCTTATCTTATTACCACTAGCCGGAATAATTGAAAACTTGCTAATCAAACCCCTTCAATACCGACACT----------GTGAATATTAATCGTTGACTTTTTTCCACTAACCACAAAGATATCGGCACCTTGTACTTTATCTTCGGCGCCTGAGCCGGAATAGTGGGCACAGCCATAAGCCTATTAATCCGAACAGAGCTCAGCCAGCCTGGCCCCTTCATAGGAGATGACCAAATTTATAATGTTGTTGTTACAGCACATGCCTTTATCATAATTTTCTTTATAGTCATACCAATCATGATCGGTGGATTTGGAAATTGACTACTCCCACTAATAATTGGAGCACCCGACATAGCATTCCCTCGCATAAACAACATAAGCTTCTGACTGCTGCCCCCATCATTTACCCTACTTCTCTTTTCCACCCTTATTGAAACTGGAGCTGGCACCGGATGAACAGTTTACCCCCCACTAGCTGGAAACCTAGCCCACGCCGGACCATCAGTAGATTTGACTATCTTCTCCCTTCACCTTGCTGGAGTATCCTCCATCCTTGGAGCAATTAACTTCATTACCACAGCCATTAACATAAAACCCCCGGCAATATCACAACAACAAACGCCCCTTTTTGTGTGATCCGTCCTAGTCACAGCCATTCTCCTGCTGCTCTCACTGCCCGTCCTAGCTGCAGGAATTACCATACTACTAACTGACCGAAACTTGAACACCACCTTCTTCGACCCCGCAGGAGGGGGGGACCCAATCCTATACCAGCACCTTTTCTGATTTTTCGGCCACCCGGAAGTGTACATCCTCATCCTACCAGGATTTGGAATAATCTCCCATGTAATTACTTTCTACTCAAGTAAAAAAGAACCATTCGGCTACATAGGAATAGTCTGAGCCATGATGTCAATCGGCTTTCTTGGCTTTATTGTCTGAGCCCACCATATGTTTACAGTAGGAATAGACGTTGATACTCGAGCATATTTCACATCCGCCACAATAATTATCGCCATCCCCACTGGCGTAAAAGTATTCAGCTGATTGGCCACCATTTACGGAGGAGTAATGAAATGACAAGCCCCCATGCTATGAGCACTTGGCTTCATTTTCTTATTCACAGTCGGAGGGCTAACAGGAATCGTACTAGCCAACTCATCACTAGACATTATCCTACACGATACCTACTACGTAGTAGCCCACTTCCACTACGTATTATCTATAGGAGCAGTATTCGCTATCATAAGCGGATTCACCCACT-GATTCCCACTA-TTTACAGGGTTTACCCTCCACCAAACATGAACAAAAA-TCCAATTCATAATCATATTTACAGGTGTAAACCTAACCTTCTTCCCACAACACTTCCTAGGCCTATCAGGAATACCACGACGATATTCAGATTACCCAGACGCATATGCCTTCTGAAATATGGTCTCCTCAATTGGGTCATTAATTTCCATAGTATCAGTTATCCTACTCACATTTATTGTATGAGAGGCATTTTCATCAAAACGAAAAGTTCAAGTGCCTGAAATAGCAAGCACAAACGTAGAATGACTAAACAATTGCCCACCACCATACCACACCTACGAAGAGCCAGTCTTTGTTCAAGTACAGCCTAAACCAACGTAG------------------------------------ATGGCAAACCCAATACACCTAGGACTCCAAGATGCAATATCCCCATTAATGGAAGAGCTCCTCTACTTTCACGACCACACACTAATAATTATTTTCTTAATTAGTACATTCGTACTATACACAATCTCAGTTCTGCTGCTAACAAGCCTTTACCACACAAATGCAACAGATGTACAAGAAATAGAAATAATCTGAACCATTCTACCAGCCCTAATCCTGATTACCATCGCCCTTCCATCTCTGCGCACATTATACCTCATAGACGAAACCACCAACCCCTGCCTGACCATTAAAGTTATTGGCCATCAATGATACTGAACATATGAATATACAGACTTCTCCCAGCTAGAATTCGACTCTTATATACTACCAACACAAGACCTACCCCAAGGCCACTTCCGCCTTTTAGAAGTAGACCACCGCATGATTGTCCCAACAAGCTCAAGCACCCGAACACTAATCACAGCTGAAGACGTCCTACACTCATGAGCGGTACCATCCTTAGGGATCAAAATAGACGCAGTGCCAGGACGACTAAATCAAACCTCACTAACTTCCCCCAACCCTGGGGTTTTCTATGG-CCAATGTTCTGAAATCTGTGGAGCAAACCATAGCTTTATACCTATCGTAGTGGAAGCTGTCCCAATACAGCACTTCCAAAACTGATTAAAAACAAACTCAT----AAATGACCCACCAAACACACCTGTTTCACATAGTCAACCCAAGCCCATGGCCAATTACAGGAGCCATAGCCGCCATAATATTAACAACTGGATTAATCCTATGATTCCACTATAATTCAAGCCTAATTCTAATTCTAGGACTAATCTCCACCTTACTGACTATATTTCAATGATGACGAGACGTTGTCCGAGAAAGCACCTATCTAGGCCACCATACCCCACCAGTCCAAAAAGGACTACGATATGGCATAATCCTCTTTATCATCTCAGAGGTTTTCTTTTTCCTCGGATTCTTCTGAGCGTTCTATCACTCAAGCTTGGCCCCAACCCCAGAACTAGGAGGACAGTGACCACCAACTGGAATTTCCACACTAGACCCATTCGAAGTCCCGCTCCTCAACACAGCAGTACTATTAGCCTCAGGAGTTACAGTAACATGAGCCCACCACAGCCTAATAGAAGCCAACCGAGCACCCGCCATCCACGCCTTAATTCTTACAATTGCCCTAGGACTATACTTCACTGCTCTTCAAGCAATAGAATACTACGAAGCCCCATTCACCATCGCAGACAGCAGCTATGGGTCAACCTTCTTTGTTGCCACAGGCTTTCACGGCCTACACGTTATTATCGGATCAACATTCCTAATAACCTGCCTCTATCGACAAATCATGCACCACTTCACCTCAAATCACCATTTCGGTTTCGAAGCTGCCGCTTGATACTGACACTTCGTAGATGTAGTCTGACTGTTCCTGTACATCTCAATCTATTGATGAGGCTCCTATAAGTTTTTTAACAATCACGCCAATCCTCATTTACATCCTCTCAGTCCTAATCGCAGTCGCATTCCTAACAGGGCTAGAGCGAAAAATCATCGGCTATATGCAGCTACGTAAAGGCCCTAATATTGTAGGCCCACTTGGACTGCTGCAGCCGTTTGCCGACGGCCTCAAGCTTATTATTAAAGAGCTAACACTCCCCATACTAGCCACCCCCGCTTTATTTATTTTATCCCCCGCAGTCGCCATTATTTTATCCTTGATTATATGAGCCCCCCTGCCTGTACCATTTTCTATCGCCAATCTAAACCTTGGCATGTTATTTTTACTAGCCATATCCAGCCTAGCAGTCTATTCACTACTATGATCTGGATGGGCATCAAACTCTAAATACGCCCTAATGGGCGCCTTACGAGCAGTAGCCCAAACCATTTCCTACGAAGTCACATTAGCTATCATTGTCCTATCTATCGTCCTACTTAGCGGGGGATTTTCACTGCACGCATTAGCTGTTATTCAAGAACCAATCTACTTAGCGCTAGCCACATGACCACTGCTGATAATATGATACACCTCCACAGTAGCAGAGACAAACCGTGCCCCGTTCGACCTCACAGAAGGGGAGTCAGAGCTAGTATCTGGGTTCAACGTTGAATACAGCGCAGGGCTATTCACACTATTCTTCCTAGCCGAATACGCCAACATTCTACTAATAAACATTTTAACCACCATCCTATTCCTAAACACATCAACAAACCTGCCGACACAAACACTATTCACCACCACCCTAATAAGTAAATCAATTCTGCTAACCGTTGGATTCCTATGAATCCGAGCATCATACCCACGATTTCGGTATGATCAACTAATACACCTACTGTGAAAAAACTTCTTGCCAGCCACGCTGGCAATCTGCCTGTGACACTCGTCCCTCCCAGTGTCAACATTCGGCCTTCCAGTAGCAA---GGATGCCCATCTTCCAGCCGATCATCTTAACCACACTAACTATCACAACACTCATTTTCCTATCCTCCACCTATTTGGTGCTCATATGGGTAGCACTAGAACTTAGTACACTAGTAGTCCTTCCATTAATTGCCAATAAATCTCACCCACGATCCATCGAAGCCTCCACAAAATACTTCCTCACACAGGCCACAGCCTCCGCCTTAATCATCTTCTCATGGGCCTTAAACCATATCACAGCAGGGAGTGGCCAAATTACAGAAGTAACAAACCAAGCCCTCACAACCATTATAGCCCTAGCCCTGTTTATTAAAATTGGGTTAGTGCCATTCCACTTCTGAGTGCCTGAAACCATTCAAGGAATAACTCCAACCGCCTCAATCTTCCTACTCACCTGACAAAAGCTAGGCCCACTAATTATATTATACCTAATGAGCCCACTAATTAATTTCGAGATCCTCTCTGTAGTGTCCATCCTGTCCACCACAGTTGCTGGATGACTTGGACTTAACCAAACCCAAATCCGAAAACTAGTAGCATTCTCTTCAATTGCCCAAATGGCTTGAACCCTGGTGATTATTAAATACGCACCATCACTTACAATCTTGGCCTTCTATTTATACTCAATCACAATTTCCACCACCCTCCTCACACTAGAAAAATTATCAACAACATCCATCAACAACCTCCTACTTTCGTTCCAAAAAGCCCCAATTACTTCCTCCCTACTAACGGTCTCTCTACTATCACTATCAGGACTGCCCCCACTAGCCGGCTTCCTCCCAAAATGACTAACAATTGACCAACTCGTGGCAGAAGGGGCAATTTGAGTCGCATTCACAATGCTCATAGCCTCCCTTCTAAGCCTGTTCTTCTACTTACGACTATGATACGACTCCGCATCCACACTTCCCCCTAACACTGCCAACACCCAACGCCTATGACGCAAACCAACCCAGCAAACCAACCTTGCAATCAACTCCATGGCTATAGCCGCCCTCACCCTAATCCTGGCAGCCACCATAATAAAAGCCATTACAAAACAAGAG------GGCTACTAAA----TAAACCTACTTACCACATTCATATTGGCCACAGCCACTGCAGTAGCCGTAATCACCCTAAACCTGCTGATATCAGAAATAACCCCAGACCCAGAAAAACTCTCACCATACGAGTGCGGATTTGACCCGCTAGGATCCGCCCGCCTGCCACTATCAATCCGCTTTTTCATAGTAGCCATCCTATTCTTACTATTTGACTTAGAGATCGCCATTCTACTACCGCTAGCATGAGCCCTCCAACTCACAAGCCTGATCAAAAGCATAACATGGGCCATCATTATCTTCTTGCTCATATTCGCTGGCCTGACGTACGAATGATTACAAGGCGGACTAGAATGAGCAGAATAGATGTTAAAAATCGTCGTACCCACAATAATACTAATCCCCGCAACCTGCCTGACAACCACAAAAAATACCTGACTATCACCAACAGCCTACTCAACAGTTATTATTATCCTAGGCATACTTATCTTAAACCCCGGAGATATTCTGATAAACGCTAGTGGTTTACTATTAGGAAGCGACCAAATCTCAACACCCCTGCTCATATTATCCTGCTGACTACTACCATTAATGTTTATAGCTAGCCAAAGCTCCATACCACACAACCAAACCCAACAAAAACAACTGTTTATTACAACCCTGGCCCTCCTACAATTAGCCTTAATACTAGTATTCATAGCCTTAGACCTGATATTATTATACACCGCCTTTGAAGCAACCCTTATTCCCACCCTAATAGTGATCGCCCGGTGGGGCTCCCAAACAGAACGACTCGGAGCCGGACTATATTTCCTCCTATACACCATCACTAGCTCCATACCCCTTCTAATCGCACTTCTATGGGTATACAACATAAAAGGAACTACATCTATTGCCATCCTACAACTATTGCCCACAACAACCCTAACATTCTGAACAGACACGTTACTATGGGTCTCGCTTATATTAGCCTTCCTGGTAAAAATCCCAATTTATGGCCTTCACCTTTGACTACCAAAAGCCCACGTAGAAGCCCCAATTGCCGGATCCATGGTCCTTGCAGCAATCCTATTAAAACTCGGAGGCTACGGCTTACTACGGGTCACAAACTTATTAACTGAACAAACTACATCCTCTTATATCATGCCCTTAACAGTAGCACTATGGGGCGCACTCATAACCGGCATAATCTGCTTACGACAAACAGACTTAAAGTCCCTAATTGCCTACTCCTCAGTAAGCCACATAGGACTAATAACAGGCGCTATCCTCACTCATAATCAACTGGCCCCCTCAGGCTCAATAATTATAATAGTAGCCCACGGCCTCACATCCTCAATACTATTCTGCCTGGCAAACATTACTTACGAACGAACACACTCACGAACCCTACTACTAACGCAAGGAGTACAACTAGCCACACCGGCCATGACATCCTGGTGGCTTCTAGCCTGCTTAACAAACATAGCACTCCCACCAACAATTAACTTTATTGGAGAACTCACCCTTATAGTATCACTATTTGACTGAGCGGACATTACCATCTTTCTAACGGGACTAAGCGCATTCGTCACCTCAACTTATACCCTACACATATTCTCCTCCACCCAACAAGGAACCCTACCAAATCACATCATAATGATGAGCCCGACCCAAACTCGAGAACACCTACTAATGGCCCTACACTCCGCGCCATCAATCGCTTTAATTTTTATCCCTCAACTGATATACTACCAATAA---ACCTCCCCAACCAATCTATTGTTCACTTTCTCTTTTATCATCTTCACCATTGGATTTACCTTCCGCCACACTCACCTCCTCTCGGCTCTACTATGCTTGGAAGGCATGATGCTATCAGTGTTCCTACTAATAACAACATGGTCCCTAAACTCAAATATCTCATCTTTCATCCTACCTTTAACAGTCCTGACGCTATCAGCATGCGAGGCCGGTATTGGCCTCGCCCTACTGGTTGCCTCAGCCCGAACACACAATACAGCCAACCTCAAAAACCTAAATCTACTCCAATGTTAAAT-AACACACCCCTCAACCCTAATTATTATACTGTTCCTGCTCCCCCTAGCAATCTTAACACTCCCAATACTAGCACCAAGCT-CAAAATTAACCTCG--CCACTTGGCACCAAAGTACTAGCAGCAAAGCTGGCCTTCCTTACTAGCCTAATCCCACTAGCCTACCTTATCCATAATGACTTAACCATCACTACATACGAAACCCAATGATCAACAATTGGCACAACCACAATTCGCGTTAGCTTCACACTAGACATCTACTCTGCCTTCTTCTTGCCCACCCTCCTTTTTGTTGTATGGTCAATCATAGAATTTACCACGCAATACATGGAATCAGACCCAAAAATTAACACTTTCTTCAACCAGCTCATCACCTTCACCCTAATAATAATAATCCTTGGAACCGCTGAAAACCTCTTTCAGTTTTTCATCGGTTGAGAAGGGGTAGGTATTATATCATTCATGCTAATCAACTGATGGTCATTCCGATCAAACTCGAACAAAGCCGCCATGCAAGCCGTAATTTATAACCGCCTAGCAGATATCGGCCTAATTATTACCCTGGCATGAATAGTTACCAACGACCTATCACTAAACATTAAAGGCGCACAAATCGCGCCAGACACAGCCCTAATCCCCGCACTCGGACTACTTCTAGCATCTGCTGGAAAATCCGCTCAATTCGGGTTCCACCCGTGACTCCCCGCAGCGATAGAGGGCCCAACCCCCGTCTCAGCCCTACTGCACTCAAGCACCATAGTAGTAGCCGGAGTGTTCCTGCTCATCCGAACCTCCGACCTCCTATACAGCAGCGAAACAGCAACCACAACCTGCCTACTACTCGGGGCCTTTACATCCGTACTAGCTGCTTCATGCGCACTATCCCAAAATGACCTAAAAAAAATTATTGCCTACTCAACTACCAGCCAACTAGGCCTTATAATGACCTCCGTAGGATTAAAACAACCTGAACTCGCATTCATACACATCTCAACACATGCGTTCTTTAAGGCAATGCTATTCCTCTGCGCAGGAACAATTATCCACAGCCTAAACAATGAACAAGACATCCGAAAAATAGGAGGACTCAACAAAATGCTTCCTACCACCTCCTCCTGCTTAATCATTGGCTCCCTCGCCCTATCAGGAATGCCATTCATGGCTGGCTTTTACTCAAAAGACGCCATCATTGAATCTATCAACACCTCCAATGTAAACTCCCTATCATTAGCCACAACCTTAATAGCAACCATCTTCACCACACTTTATAGCCTACGCATAATCTACTACGTAGCCCTAAGCTCCCCACGAACCCCACCATTACCCGCCATCTCCGAAACCCCCCAGATAACCAACCCCATCCTACGACTAGCCATCGGGAGCATCGCAGCCGGGCTCATGATTTCAACAACCATTCTGCCACCCAACACTCCACAACTAACCATGCCAACATCAACCAAACTAGCCACACTAAGCATCCTAATCCTTGGATTACTGATCGGCTCAATCCTAATTACAGTAGCAAACCAACTACCCTACTCCATCAAAGGCACCCAAAACCCCCTTACCTACAAAATTACCCACTCCTACTTCATCCTACATCACATCCTATCATCCATAGTCTTACAAATTAGCCAAAAACTATCAACCCACCTGATAGACCAGACACACTATGAAGCCTTGGGCCCTAAAACAATAACCTACCTGCAAATACTAATGGCCAAGATATTAACCAACTTTCATAAAGCTCGAATTAACCCATACTTAAAAGCCATTGTCCTATCCATCATGCTAATTTCCCTACTCTACTTCACCTCAATGAACGTAGAGCCCCCCGATGCCGGCCACGAACTAGGGCCATAAATGGGAATTACATTTTTTCTTCTTTGTTGTTTAATGATGGCTAGTGTAGTATTAGTGGCGTCGGGGGTGGCGATCCATTATGGGGTGGTTAGTTTGCTTTTTGCTGCAATATTTGGAAGTGGGCTACTAGCAATGGGGGGTGGAAGTTTTATGCCGCTTGTGGTACTTCTGATTTATTTAGGTGGCCTGCTGGTAGTTTTTGCTTTTTGTGTGGGGTTCACTGATGATAAATACTGTGAGTTCTGGGGAGCATGGGGGTCTAAGGTGTCGGCTTATGTTTGTGGGGTTGGCTTAGGTATTGGTGGATATTGTGTATATGACTCTGCGTGA----GCCGCGGTTTTGGGGTGTTTTG--TTGATGCCGTTGGGACCTGGAGTGGTGATGTTAGCGACGAGTTTTTAGGGGTCGGGTTGTTTTATTCAAGTGGTTGGGGGCTGCTTATTTTGAGCGGTTGGGCCTTATTGGTGGTGTTGTTTACTATTATGGCCCTAGTTCGTGGCCGGCATCGGGGGGCTCTACGTTCATTGAGG

*Crocodylus mindorensis*

------------ACAAACCTAAATTTATTTGACCAATTCTTAATGCCTAAACTTCTTGGCATATCCCTATTAATCCCAGCCATATTATTAACAACAGTGCTAATCTATAACCCACAAGACCGCTGACTATCAAACCCTATAACAACCCTACAATCCTGACTAATTGCAAAAGCCACTAAACAAATCATAACCCCAGTAAACAAACCGGGGCACAAATGATCATTAATGCTGATCTCATTACTAACAATGCTCGTTTTAAATAACCTCTTAGGCCTCCTCCCATATACATTTACACCAACAACTCAACTATCTATAAACATAGCCCTAGCCCTACCACTATGATTGGCAACAGTATTAATTGGCCTACGAAATAAGCCAACTTCTTCACTGGCCCACCTCCTGCCAGAAGGGACCCCAACACCCCTAATCCCAATCTTAGTCTTAATCGAGACAATCAGTCTACTTATCCGACCAATCGCACTTGCCGTGCGACTCACAGCCAACCTCACCGCAGACCATCTTTTAATACACCTAGTTTCTACTGCAGCACTTAGCCTAATGACAACTTCCACGCTACTCGCCGGACTAACCCTAACCATCCTAGCCCTTCTAATACTCCTAGAAATTGCAGTAGCAATAATTCAGGCATATGTATTCACCCTACTACTATCACTATACCTGCAAGAAAACGTATAAATGCCACAATTAAACCCAGAACCTTGACTAATAATCTCTTCTATTACATGGCTAGTATTCATCTTTACCCTTCAGCCAAAAATTGCTTCTCTAAAATTCATAAATGCCCCAAGCAGCCCAAACCAAAAAACCACC-AAAACATGACCATGGCCACAAACCTAA---------------------ATGGCCCACCAACTACGAAAATCCCACCCGCTCTTAAAACTAGTAAACTACTCTTTAATCGACTTACCCACACCGTCCAACATCTCCTACTGATGAAACTTTGGATCACTTCTTGGATTCACCCTATTAATCCAACTAGCATCGGGCATCCTACTAATAATGCACTTCCTAGCAGATGACTCTTTGGCCTTTATATCCGTCGCTTACACCTCACGAGAAGTCTGATACGGCTGACTAATCCGAAGCCTTCATGCAAACGGGGCTTCTCTATTCTTCCTATGCATTTTCCTACACATCGGACGTGGACTATACTACGGATCATACCTTAATGAAAACACATGGAACATTGGAGTACTGCTACTACTATTACTGATAGCAACTGCCTTCCTGGGCTACGTCTTACCATGAGGACAAATATCATTCTGAGGAGCAACTGTAATCACCAACCTAATATCAGCCATCCCCTACGTAGGTAACTCAATTGTGACCTGAATTTGAGGAGGACCATCCGTCAATAGCGCAACCCTAACACGATTTACCACACTACACTTCCTCCTCCCATTCATCCTCATGGCCGCTGTCATCACACACCTCATCTTCCTCCACGAGCACGGATCATTCAACCCCCTGGGACTAGTCCCTAACGCCGATAAAATTCCGTTCCACCCATATTTCTCAGCAAAAGACGCCATAGGAGTAGCCCTAGCCACCCTCCTACTAATGACTCTCACATTATACCTCCCAAACCTATTAGGAGACCCAGAAAACTTCACCCCAGCCGACCCCATAAAAACACCAGACCACATTAAACCAGAATGATACTTCCTATTCGCCTACACAATCTTACGGTCCATTCCAAACAAGCTCACAGGGGTACTTGCCATATTCGCATCCATCCTAGTATTATTGCTTCTACCTGCACTACACACATCAAAACGACAATCAATAAGCCTACGCCCCCTATCCCAACTCCTATTCTGAACCCTAATCGCAGATTTCTTTATCCTTACATGAATCGGAGGACAGCCAGTACAAGACCCATACACCCTAATTGGACAAATAGCCTCATTCATCTACTTCTTCATCATCCTTATCTTATTACCACTGGCTGGAATAATCGAAAACTTGTTAATCAAACCCCTCCAATACCGACCAT----------GTGAATATTAATCGTTGACTTTTTTCCACTAACCACAAAGACATTGGCACCTTGTATTTTATTTTCGGCGCCTGAGCCGGGATAGTAGGCACAGCCATAAGCCTATTAATCCGAACGGAGCTCAGCCAGCCTGGCCCCTTCATAGGAGATGACCAAATTTATAACGTCATTGTTACAGCACATGCCTTTATCATAATTTTCTTTATAGTTATACCAATCATGATCGGCGGATTTGGAAATTGACTACTCCCGCTAATAATTGGGGCACCCGACATAGCATTCCCTCGCATGAACAACATAAGCTTCTGACTACTACCCCCATCATTCACCCTACTTCTCTTTTCCGCCTTCATTGAGACTGGGGCTGGAACCGGATGAACAGTTTACCCACCGCTAGCTGGAAACCTAGCCCACGCCGGGCCATCAGTAGACCTAACCATCTTCTCCCTTCACCTTGCTGGAGTATCATCGATCCTTGGGGCAATTAACTTCATTACCACGGCTATTAATATAAAACCCCCAGCAATGTCACAACAACAAACACCCCTATTTGTGTGATCTGTCCTAGTCACAGCTGTCCTACTACTACTCTCACTGCCCGTCCTAGCTGCGGGAATTACCATACTACTAACTGATCGAAACTTAAACACCTCCTTCTTTGACCCCGCAGGAGGAGGAGACCCAATCCTATACCAACACCTTTTCTGATTTTTCGGACACCCAGAAGTATACATCCTTATCCTGCCAGGATTTGGAATAATCTCACATGTAATTACATTCTACTCAAGTAAAAAAGAACCATTCGGTTACATAGGGATAGTCTGAGCCATAATGTCAATCGGCTTTCTTGGCTTCATTGTCTGAGCCCACCACATGTTTACAGTGGGGATAGATGTTGACACTCGAGCATACTTCACATCCGCCACAATAATTATCGCCATCCCCACTGGCGTAAAAGTGTTCAGCTGACTGGCCACTATTTATGGGGGAGTAGTGAAATGACAAGCCCCAATGCTCTGAGCACTCGGCTTCATTTTCTTATTCACAGTTGGAGGACTAACAGGAATTGTACTAGCTAACTCATCACTAGACATTATTCTACACGATACCTACTACGTAGTAGCCCACTTCCACTACGTGTTATCCATAGGAGCAGTATTCGCCATCATAAGCGGACTCACCCACT-GATTCCCATTA-TTTACAGGATTTACCCTCCACCACACATGAACAAAAA-TTCAATTCATAATCATGTTTACAGGTGTAAACCTAACCTTCTTCCCACAACATTTCCTGGGCCTATCAGGAATACCACGACGATACTCAGACTACCCAGACGCATATGCCTTTTGAAATATAATCTCATCGATCGGATCGCTAATTTCCATAGTATCAGTTATCCTACTCACATTTATCGTATGAGAAGCATTTTCATCAAAACGAAAAGTTCAAGTACCGGAAATAGCAAGCACAAACGTAGAGTGACTAAACAATTGCCCGCCATCATACCACACCTACGAAGAACCAGTCTTTGTTCAAGTACAACCGAAACTAGCGTAA------------------------------------ATGGCAAACCCAATACACCTAGGACTCCAAGATGCAATATCCCCACTAATAGAAGAACTCCTCTATTTTCATGACCACACACTAATAATTATTTTTTTAATCAGCATGTTTGTACTATACACAATCTCAGTTTTACTACTAACAAACCTATACCACACAAATGCAACAGATGTACAAGAAATAGAAATAATCTGAACCATTCTGCCAGCCCTAATCCTCATCACCATCGCCCTTCCATCCCTGCGCACATTGTACCTCATAGACGAAACCACCAACCCCTGCCTAACCATTAAAGTCATCGGGCATCAATGATATTGAACATATGAATACACAGACTTCTCCCAACTGGAATTCGACTCTTATATGCTTCCAACACAAGATCTGCCTCAGGGTCACTTCCGCCTTTTAGAAGTAGACCACCGCATGATTGTTCCAACAAACTCAAGCACACGAACACTAATCACAGCCGAAGACGTCCTGCACTCATGAGCAGTACCATCCTTGGGAATTAAAATAGACGCAGTACCAGGACGACTAAACCAAACCTCACTAACATCCCCCAACCCTGGAGTCTTCTATGG-CCAATGTTCCGAAATCTGTGGAGCAAACCATAGCTTTATGCCTATTGTCGTGGAAGCTGTCCCAATACAGCACTTCCAAAACTGATTAAAAACAAACTCAT----AAATGACTCACCAAACACACCTATTTCACATAGTCAACCCAAGCCCATGGCCAATTATAGGGGCCATAGCTGCCACAATACTAACAACCGGATTAGTCCTATGATTCCACTGCAACTTAAGCCTAGTCTTGCTTCTGGGACTAATATCCACCTTGCTGATTATATTTCAATGATGACGAGACATTGTCCGAGAAAGCACCTATCTAGGCCACCACACCCCGCCAGTCCAAAAAGGACTACGATACGGTATAATCCTTTTTATCACCTCAGAGGTCTTCTTTTTCCTCGGATTCTTCTGAGCGTTCTATCACTCAAGCTTGGCCCCAACCCCAGAACTGGGAGGACAATGGCCCCCAACCGGAATTACCACACTGGACCCATTCGAGGTCCCACTACTCAACACAGCAGTTCTGCTAGCCTCAGGAGTCACAGTAACATGAGCCCACCACAGCCTAATAGAAGCCAACCGAACACCCGCCATCCACGCCCTAATTATCACAATTATTCTAGGACTATACTTCACTGCTCTTCAAGCAATAGAATACTACGAAGCCCCATTCACTATCGCAGACAGCAGCTATGGGTCAACTTTCTTTGTCGCCACAGGCTTTCACGGCCTACACGTTATTATTGGATCAACATTCCTAATAGCCTGCCTCTATCGACAAATCATACACCACTTCACCTCAAACCACCATTTCGGTTTCGAAGCTGCCGCTTGATACTGACATTTCGTAGATGTAGTCTGACTATTCCTATACATCTCAATCTATTGATGAGGCTCCTATAAGCTTTTTAATAGTCACACCCATTCTTATTTATATCATCTCAGTCCTAATCGCAGTCGCATTCCTGACAGGGCTAGAACGAAAAATCATTGGCTATATGCAACTACGTAAAGGCCCCAATATTGTGGGCCCTCTTGGGCTGCTACAGCCATTTGCTGACGGCCTCAAGCTTATCATTAAAGAGCTAACACTACCCCTACTCGCCACCCCTGCCCTATTTATTTTGTCCCCCGCAGCCGCCCTTATTCTATCCCTAATCATATGGGCCCCCCTACCCGTACCATTTTCTATCGCCAATCTAAACCTTGGCATGCTATTTTTACTAGCCATATCCAGCTTAGCAGTCTATTCACTACTATGGTCCGGCTGAGCATCAAACTCCAAATACGCCCTAATAGGCGCCTTACGAGCCGTGGCCCAGACCATCTCCTATGAAGTCACATTAGCCATCATCGTTCTGTCTATTGTCCTCCTTAGCGGCGGATTTTCACTACACGCGCTAGCTATTACCCAAGAGCCTGTCTACTTAGCACTAACCACATGACCACTACTGATAATATGATACACCTCAACACTAGCAGAGACAAACCGTGCCCCTTTCGACCTTACAGAAGGTGAGTCAGAGCTAGTATCTGGGTTCAACGTTGAATACAGCGCAGGGCTGTTCACACTGTTTTTTCTAGCCGAATACGCCAACATTCTACTAATAAACACCTTAACCACCATCCTATTCCTAAACACATCAACAAATTTGCCAACACAGACACTATTCACCACCACCCTAATAACCAAATCAACTCTATTAACCATCGGATTCCTATGAATCCGAGCATCATACCCGCGATTCCGGTATGATCAACTAATACACCTGCTGTGAAAAAACTTCTTGCCAGCCACGCTGGCAATCTGCCTGTGACACTCGTCGCTCCCAGTGTCAACATTCGGTCTTCCAGTAGCAA---GGATGCCCATCTTCCAACCAGTCATCTTAACCACACTAGCCATCACAACACTCATTTTTCTGTCCTCCACCCATCTAGTGCTCATGTGGGTAGCACTAGAACTTAGCACACTAGTAGTTATCCCACTAGTTGCCAACAAATCGCACCCACGATCCATCGAAGCTTCCACAAAATATTTCCTCACACAAGCCACGGCTTCTGCCTTAGTCATCTTCTCATGAACCTTAAACTATATCACAACCGGGGGCGGCCAAATTACAGAAGTAACAAACCAAACCCTTACAACTATTATAGCCCTAGCCCTCTTTATTAAAATTGGACTAGTGCCATTCCACTTCTGAGTGCCTGAAACCATTCAAGGAATAACCCCAACCGCCTCAATTTTCCTACTCACCTGACAAAAGCTAGGCCCGCTAATTATATTGTACCTAATGAGTCCGCTAATTAACTTCGAGGTCCTCTCCACAGTATCTATCCTATCCGCCACAATTGCTGGCTGGCTTGGACTTAACCAGACCCAGATCCGAAAACTAGTAGCACTCTCCTCAATTGCCCAAATAGCTTGAACCCTGGTGATTGTTAAATACGCACCATCGCTTACAATCCTTGCCTTCTATCTATATTCAATTACAATTTCCACTACACTTCTCACATTAGAAAAACTATCAACAACATCCATTAATAACCTCCTACTCTCCTTCCAAAAAGCCCCAATCACTTCTTTACTACTGACAGTCTCTCTGCTATCCCTATCAGGCCTACCACCACTAGCCGGCTTCCTCCCAAAGTGGCTAACAATTGACCAACTCGTAGCAGAAGGGGCAATCTGAGTCGCATTCACAATACTCATAGCCTCCCTTCTAAGCCTATTCTTCTACCTACGACTATGATATAACTCCGCATCTACACTTCCCCCTAATACTGCTAACACCCAACGCCTATGACGCAAACCAATTCAACAAACCAACCTTACAATCAACTCCCTAGCTATAGCCGCCCTCACCCTAATCCTGGCAGCTACCATAATAAAAGCCATTACAAAACAAGAG------GCCTATTAAA----TAAACCTACTTACCACACTCATATTAGCCACAGCCACCGCAGTAGCCGTAATCACCCTAAACCTGCTGATGTCAGAAATAGCCCCGGACCCAGAAAAACTCTCACCATATGAATGCGGATTTGACCCACTAGGGTCCGCCCGCCTACCACTATCCATCCGCTTTTTCATAGTAGCCATTCTATTCTTACTATTTGACTTAGAAGTCGCCATCCTACTACCACTAGCATGAGCTCTCCAACTCACAAGCCTGATCAAAAGCATCACATGAGCCATCACTATCTTCTTACTCATATTTGCAGGCCTAACATACGAGTGGCTACAAGGAGGACTAGAATGAGCAGAATAGATGTTAAAAATCGTTGTACCAACAATAATATTAATCCCCTCATCCTGCCTAACAACCGCAAAAAACACCTGATTATCGCCTACAGCCTACTCAACAGCCATTATTATCCTAGGCATGCTTGTCTTAAACCCCGGAGATATTCTGATAAACACTAGCGGTCTATTATTAGGAAGTGACCAAATTTCTACACCATTACTTATATTATCCTGCTGACTATTACCACTAATATTTATAGCCAGCCAAAGCTCCATGTCGCACAACCCAGCCCAACAAAAACGACTTTTCATTACGGCCCTGGCCCTCCTACAACTAGCCCTAATGCTAGTATTCATGGCCTTAGACCTAATGCTATTTTACACCGCTTTTGAGGCAACCCTCATTCCCACCCTAATAGTGATCGCCCGATGGGGCTCCCAAGCAGAACGACTCGGAGCCGGACTATACTTCCTCCTATACACTATCACTAGCTCTATGCCCCTTTTAGTTGCACTTCTATGGGTATATAACATAAAAGGAACTACGTCCATTACGCTCTTACAACTACTGCCCCCAATAACCCTAATATTCTGAACAAACACGCTACTATGGACCTCCCTCATATTAGCCTTCCTAGTAAAGATCCCAATTTACGGCCTTCACCTTTGACTACCAAAAGCCCACGTAGAGGCCCCAATTGCCGGATCCATGGTCCTTGCAGCGATTCTATTAAAACTCGGAGGCTATGGCTTGCTACGAATCACAAACCTACTAACTGAACAAACCACAGCCTCTTATATCCTACCCCTAGCAGTAGCACTGTGGGGCGCACTCATAACCAGCATAATCTGCTTACGGCAAACAGACTTAAAATCCCTAATTGCCTACTCCTCAGTAAGCCACATAGGACTAATAACAAGCTCAATCCTCACTCGGAACCAATTGGCCCCCTCAGGATCAATAATTATAATAGTGGCCCACGGCCTTACATCCTCAATGCTATTCTGCCTGGCAAATATCACCTACGAACGAACACACTCACGAACCCTACTACTAACGCAAGGAGTACAACTAACCACCCCGGCCATGACATCCTGATGGCTTCTAGCCTGCTTAACAAACATAGCACTCCCCCCAACAATTAACTTTATTGGAGAGCTCACCCTCATAGTATCACTATTTGACTGAGCGGACATTACTGTTTTTTTAACAGGGCTAAGCGCATTCATCACCTCAATCTACACCCTACACATATTCTCCTCAACCCAACAAGGAACCCTTCCAACCCACATTATCACAATAAGCCCAACCCAAACACGAGAACACCTACTAATAGCGCTACACTCCGCACCATCAGTCGCTTTAATTTTTATTCCTCAGCTAATATATCACCAATAA---ACCTCCCCCACTAACCTGTTGTTCACCCTCTCTTTCATCATCTTCACCATCGGGTTTACCTTCCGCCACACCCACCTCCTCTCAGCTCTACTATGCCTGGAAGGCATGATATTATCCGTATTCCTACTGATGGCGGCATGGTCCCTAAACTCAAACATCTCATCTTTCATCCTACCTTTAACGGTCCTAACACTATCAGCATGTGAGGCTGGTATTGGCCTCGCCCTACTGATTGCCTCAGCCCGGACACACAATACAGCCAACCTCAAAAACCTAAATCTACTCCAATGTTAAAT-AACACAGCCCTCAGCCTTAATTATTATACTTTTCCTACTCCCCCTAGCAATCTTGACACTCCCAATGCTAATACCAAGCT-CAAAATTAACCTCA--CCACTTAGCACCAAAGTGTTGACAACAAAATTGGCCTTCCTTACTAGTCTAGTCCCACTAACCTACCTCATCTACAACGATCTAACCATCATTACATACGAGGCCCAATGGTCAACAATTGGCACAACCACAATTCACATTAGCTTCACATTAGACACTTACTCCACCCTATTCTTGCCCATCCTCCTTTTTGTTGTATGATCCATCATAGAATTTACCGTACAATACATAGAATCCGACCTAAAAATTAATACTTTCTTTAACCAACTCACCACCTTCACCCTAATAATAATAGTCCTTGTGACCGCTGAAAACCTATTCCAGTTTTTCATCGGCTGAGAAGGAGTAGGCATTATATCATTCATGTTAATCAACTGATGATCATTCCGATCAGACTCAAACAAAGCTGCTATACAAGCCGTAATTTACAACCGCTTGGCAGACATTGGCCTAGTCATCACTCTAGCATGAATAGTCACTAACAACCTATCACTAAATATTAAAGGCATGCAAACCACACCAGACATGGCCCTCATCCCCGCACTCGGACTACTTCTAGCAGCTGCTGGAAAATCTGCTCAATTCGGGTTCCACCCATGACTTCCAGCAGCAATAGAAGGCCCAACCCCTGTCTCAGCCCTGCTTCACTCAAGCACTATAGTGGTAGCTGGAGTATTCCTGCTCATCCGAACCTCAGACCTGCTATACAGCAGCAAAACAGCAACCACAGCCTGCTTACTACTAGGAGCCTTCACATCTATATTAGCCGCTTCATGTGCACTAACCCAAAAAGACCTAAAAAAAATTATTGCCTACTCTACTACCAGCCAACTAGGCCTAATAATGACCTCCATCGGATTAAAACAGCCTGAACTCGCATTTATACACATCTCAACACACGCATTCTTTAAAGCAATATTATTCCTATGCGCAGGGACAATTATCCACAGCCTAAACAATGAACAAGACATCCGAAAAATAGGAGGACTCAAAAAAGCTCTTCCCGCTACCTCTTCCTGCTTAATTATTGGCTCCCTCGCCCTATCAGGAATGCCATTCATGGCTGGCTTTTACTCAAAAGACGCCATCATTGAGTCCATCAGCACCTCCAATGTAAACTCCCTATCACTGGCCATAACCCTAGTAGCAACCATCTTCACTACCCTTTATAGCCTACGCATGATCTATTATGTAGCCCTAAACACCACACGGACCCTACCACTATCCGCCACTTCCGAGACCCCTCAGATAACCAACCCCGTTCTACGACTAGCAATTGGGAGCATCGCAGCCGGACTCATAATTTCAACTGCCATTCTACCACCTAATATTCCACAACTAACCATGCCTACATCAACCAAACTAGCCGCACTAGGCATCCTAATACTAGGATTACTAGTCGGCTCAATTCTAATTACAACAGCAAATCAACTACCCAGCTCCATCAAAGGCACCCAAAACCCCCTTATCTCTAAAATCCTCCACTCATACTTCATTCTACATCACACCCTATCATCCACAGCACTACAAATTAGCCAAAAATTATCAACCCACCTGATAGACCAAACACACTATGAAGCCTTGGGCCCTAAAACAACAACCTTCCTACAAATGCTGATGGCCAAACTGCTAACTAACTTTCATAAAGCCCGAATTAACCCGTACTTAAAAGCCATTATTCTATCCATCGTACTAATTTCCCTACTCTACTTCACCTCAATGAACGCAGAGCCCCCCGACGCCGACCACGAACTAGAAT-----ATGGGAATTACATT---TCTTCTTTGTTTTTTAATGATAATTAGTGTGGTGCTAGTGGCGTCGGGGGTGACGATCCACTATGGGGTGGTTAGTCTGCTTTTTGCTGCAATATTTGGTAGTGGGTTGTTGGCAGTGGGGGGTGGAGGTTTTATGCCGCTTGTAGTATTATTAATTTATTTGGGTGGATTGTTGGTAGTTTTTGCTTTTTGTGTGGGGTTCACTGATGATAAATATTGTGAGTTCTGGGGAGCAGGTGGGTCTAAGGTGTCGGCTTATGTTTGCGGGTCTGGCTTGGGTGTTGGTGGGTATTATATATACGACTCTGCATGA----GCCGAGGCTTTGGGGTGTTTTG--TTGATGCCGTTGAGATCTGGGGCGGTGATGTGAGCAGTGAGTTTTTAGGGGTTGGGCTATATTATTTAAGTGGTTGGGGATTTCTCGTTTTGAGTGGTTGGGCTTTATTGGTGGTACTATTTACTATTATAATTCTAGTTCGTGGTCGGCGTCGGGGGGCTCTGCGTTCATTGAGG

*Osteolaemus tetraspis*

------------ACAAACCTAAATTTATTTGATCAATTCTCAATGCCTAAAATCCTTGGCATATCATTATTAACACCAGCCATATTATTAACAGCAATACTACTATATAATCCACAAGATCGTTGACTGTCAAACCCATTAACTACCCTACAATCATGATTAGTTGCGTATACTACTAAGCAAATTATAACTCCAGTGAGTAAACCAGGACACAAATGATCCCTGATACTGATATCCTTACTAACCCTTCTTATTCTTAACAACCTTCTCGGGCTCCTTCCATATACCTTTACACCAACAACTCAGCTATCCATAAACTTGGCCCTAGCCTTACCATTATGGCTGGCAACAGTATTAGTCGGCCTACGAAACAAGCCAACTTCCTCATTAGCCCACCTCCTGCCAGAAGGAACCCCAACACCACTAATCCCAATCTTAATTTTAATTGAAACAATTAGCCTACTTATCCGACCAATCGCACTCGCCGTGCGACTTACAGCCAACCTGACTGCAGGACATCTCTTAATTCACCTAATCGCTGCCACAGCCCTTAACTTGATAACAACATTCCCACTACTTACTGGACTAACCCTAATTGTCTTAACTTTACTAATACTCCTGGAAATTGCAGTAGCAATAATCCAGGCATACGTATTTACCCTATTACTCTCACTATACCTACAAGAAAACGTATAAATGCCACAATTAAACCCAGAGCCATGATTAACAATTCTATCTGTCACATGACTAGTACTTATTGTCATCTTACAACCAAAAATTGCCTCCTTAAAATTCATAAATAGCCCCACCCACCCAGACCAAAAAACTACT-AAACCATGACCCTGGCCACAAACCTAA---------------------ATGATCCACCAACTACGAAAATCCAACCCGCTATTAAAATTGATAAATAATTCCCTAATTGATCTGCCAACACCATCCAATATCTCCTATTGATGAAATTTCGGATCCCTGTTAGGATTCACCTTATTAATCCAACTAGCATCAGGCATTCTACTAATAATACACTTCCTAGCCGATGACACCATAGCTTTTATATCCGTGGCTTACACCTCACGAGAAGTATGATACGGATGACTAATTCGCAGCCTCCACGCAAACGGCGCCTCCCTATTTTTCCTATGCATTTTCCTTCACATGGGACGAGGACTATACTACGGATCATACCTACATGAAAACACATGAAATATTGGAGTACTACTACTATTATTACTAATAGCAACAGCCTTCATAGGATACGTCCTCCCATGAGGACAAATATCATTCTGAGGGGCAACTGTAATCACCAACCTTATATCAGCCATCCCATACATAGGAGACTCAATCGTAACCTGAATTTGAGGCGGACCATCTGTTAACAGCGCAACCCTAACACGATTCACCACCCTACACTTCCTACTCCCATTTATCCTAATAGCCGCCATCATCACACATCTAATCTTCCTTCACGAGCACGGGTCATTCAACCCACTAGGATTAACTTCCAACGCCGACAAAATTCCATTCCATCCATACTTCTCAACAAAAGACGCAGTAGGTGCAATCATAGCCACCATTATATTAACAACCCTGGCATTCTACTTCCCAAACCTTCTAGGAGACCCAGAAAACTTTACCCCAGCCGACCCCATAAAAACACCTGACCACATCAAGCCAGAGTGATATTTTCTGTTCGCCTACACAATCTTACGATCTATCCCAAACAAACTAATAGGAGTCATTGCCATATTCATGTCAATCTTGGTACTTCTACTCATACCCATACTTCACACATCAAAACAACAATCTATAAGCCTACGCCCACTATCACAACTTCTATTCTGAACTCTTACCGCAGACTTCCTCATTCTCACATGAATCGGGGGCCAACCAGTACAAGACCCATACATCTTAATCGGACAGATAGCCTCCTCCGTCTACTTCTTCACCATCCTCATCCTTCTACCACTAGCCGGGACAATTGAAAATATATTAATCAAACCACTTCAATACCGGCCAT----------GTGAATATTAACCGTTGACTTTTTTCCACCAACCATAAAGACATTGGCACCTTATACTTTATCTTCGGCGCATGAGCTGGAATAGTGGGAACAGCCCTAAGCCTTCTAATCCGGACAGAACTAAGCCAACCTGGCCCTCTTATAGGAGATGATCAGATTTACAATGTCATTGTCACAGCACATGCCTTTATTATAATTTTCTTTATGGTAATACCAATCATAATCGGAGGATTTGGAAACTGGCTACTGCCACTAATAATCGGAGCACCCGATATAGCATTTCCCCGAATAAATAACATAAGCTTCTGATTATTACCGCCCTCATTTATCTTACTTCTATTTTCCGCCTTCATTGAAACAGGCGCCGGTACAGGATGAACTGTTTACCCACCACTAGCTGGGAATCTGGCCCACGCTGGGCCATCAGTAGATTTAACTATCTTCTCCCTTCATCTTGCTGGGGTTTCATCAATTTTAGGGGCAATCAACTTTATCACCACAGCCCTTAATATAAAACCACCATCAATGTCACAACAACAAACACCACTTTTCGTATGATCCGTACTAGTCACGGCTGTACTCCTACTGCTCGCCCTACCGGTCCTAGCGGCAGGAATTACTATATTACTCACTGACCGAAACTTAAACACTACCTTTTTCGATCCTGCTGGAGGAGGGGATCCAATCCTATACCAACACCTCTTCTGATTCTTTGGCCACCCAGAAGTCTACATCCTCATCCTACCTGGATTTGGAATAATCTCTCATGTAATCACTTTCTACTCAAGTAAAAAAGAACCTTTCGGATATATAGGAATAGTGTGAGCCATAATATCAATTGGCTTCTTAGGCTTCATTGTATGAGCCCACCACATATTCACAGTAGGAATGGACGTGGACACTCGAGCATACTTTACATCCGCCACAATAATCATCGCCATCCCTACAGGTGTAAAAGTATTTAGCTGATTAGCCACCATTTATGGAGGAGTAGTAAAATGACAAGCCCCAATGCTCTGGGCACTTGGCTTTATTTTCCTATTTACAATTGGTGGATTAACAGGGATTGTTCTAGCCAACTCATCACTAGATATTATTCTCCACGACACTTACTACGTAGTAGCTCACTTCCACTATGTGTTATCCATAGGAGCAGTATTCGCCATCATAAGCGGATTCACCCATT-GATTCCCATTA-TTTACAGGGTTCACCCTTCATCACACATGAACCAAAA-TTCAATTCATGATTATGTTTGTAGGTGTAAACCTAACCTTCTTCCCACAACATTTTCTAGGCCTATCGGGGATGCCACGACGATACTCAGACTATCCAGACGCATATTCCTTCTGAAATACAACCTCCTCAATTGGCTCACTAATCTCTATAATTTCAGTTATTCTTCTCATATTTATTGTATGAGAGGCATTTTCATCGAAACGAAAAATTCAACTGCCAGAAATATCAACCACAAATGTAGAATGGCTAAACAACTGCCCGCCATCATACCACACCTACGAAGAGCCGGTATTTGTTCAAGTACAACCAAGCCTAATCTAT--------------------------------------GGCAAACCCAATACACCTGGGACTTCAAGATGCAATATCCCCATTAATAGAAGAACTCCTCTATTTCCACGACCACACACTAATAATCATTTTCTTAATCAGCATGTTTGTATTATACACAATTTCATTTTTACTGTTAACAAAACTATACAATACAAACGCCACAGACGTGCAAGAGATAGAAATAATTTGAACTATCCTGCCTGCCCTCATCTTAATCACCATTGCCCTACCATCCCTACGCACATTATACCTTATAGACGAGACCACCAACCCATGCCTGACCGTAAAAATTATTGGGCATCAATGATATTGAACCTACGAATACACAGATTTTTCCCCACTAGAGTTTGACTCTTACATACTTCCCACACAAGACCTTCCCCAAGGCCATTTTCGTCTCTTAGAAGTAGACCACCGCATAGTTATCCCAACAAATTCAAGCACTCGAGCCCTAGTCACAGCCGAAGATGTCCTACACTCTTGAGCAGTACCATCATTAGGAGTCAAAATGGACGCAGTACCAGGCCGCCTAAATCAAACCTCACTAACATCTCCAAATCCTGGAGTTTTCTATGG-CCAATGCTCAGAAATTTGTGGGGCAAATCATAGTTTTATGCCCGTTGTTGTAGAAGCTGTCCCAATACGACACTTCCAAAACTGATTAAAAACAAACACCT----AAATGACTCACCAAATACACCTTTTTCACATAGTTAACCCAAGCCCGTGACCCATCATAGGAGCCATAGCCGCCATAATATTAACAACCGGGTTAATCTTATGATTCCACTGTAATTCAAACCTAATCCTGCTACTGGGGTTGGTATCAACCATAGCAATTATATCTCAATGATGACGAGACATTATTCGAGAAAGCACCTACCTAGGCCACCACACACCGCCAGTACAAAAAGGACTACGATATGGTATAATCCTATTTATTACCTCAGAGGTCCTATTCTTCCTCGGGTTCTTCTGAGCATTCTATCATTCAAGCTTATCCCCAACCCCCGAGCTAGGAGGACAATGACCACCAACTGGAATCACCACCCTAGACCCATTTGAAGTCCCCCTGCTAAACACGGCAGTACTACTCGCCTCAGGAGTTACAGTAACGTGGGCCCACCACAGCCTAATAAAAGCCGACCGAAAATCCGCCATCCACGCCCTAATACTCACAATTATTTTAGGGTTATATTTTACTGCCCTTCAAGCAATAGAGTATTACGAAGCCCCATTCACCATCGCAGACAGCAGCTATGGGTCAACATTCTTTGTTGCCACAGGATTTCACGGCCTACATGTTATTATTGGATCGACATTCCTAACAGCCTGCCTATACCGACAAATCATATACCACTTCACCTCAAACCACCACTTTGGATTTGAAGCCGCCGCCTGATATTGACATTTCGTTGATGTAGTCTGACTATTCCTATATATTTCGATCTATTGATGAGGCTCTTATAAGCTTCCAAACAGCTGCCCCTATCATACTTTATATTATCTTAGTCCTGATTGCAGTTGCATTCCTAACAGGCCTGGAACGAAAAATCATCGGGTATATACAACTACGAAAAGGCCCTAACATTGTAGGACCACTTGGCTTGCTACAGCCATTTGCTGACGGACTCAAACTTATTATTAAAGAACTAACATTCCCCCTACTTGCCACCCCTGCTTTATTTATTTTATCCCCTGCAGTAGCCCTCATCCTATCATTAATTATATGAGCCCCCCTACCAGTGCCATTCTCCATTGCTGACCTAAACCTTGGCATATTATTTTTATTAGCAATATCAAGCTTGGCAGTCTATTCACTATTATGGTCCGGTTGAGCATCAAATTCTAAATACGCGCTAATAGGGGCCTTACGGGCAGTAGCCCAAACTATTTCATATGAGGTAACATTAGCTATTATTATTCTGTCTATTGCACTGCTGAGCGGTGGGTTTTCATTACACGCTCTCACCATCACCCAAGAACCCATCTTCATAGCATTAACCACATGGCCGCTACTAATAATATGATACACCTCAACACTAGCAGAGACAAACCGAGCTCCATTTGACCTAACAGAAGGAGAATCAGAACTAGTTTCTGGATTCAACGTTGAATATAGCGCAGGACTATTTACACTGTTTTTTCTAGCTGAGTATGCCAACATCTTACTAATAAACATTTTAACCACTGTCCTATTCCTCAATACATCAACAAATCTAACTACACAAGCACTATTTACTACCGCCTTAATGAGCAAATCTATTTTACTTACTATAGGCTTCCTATGAGTTCGAGCATCCTACCCGCGGTTCCGCTACGACCAATTAATACACCTATTGTGAAAAAATTTCCTACCAGCTACATTGGCAATCTGCCTATGACACTCATCACTGCCGACATCAATATTTGGTTTACCAGTAATAA---GGATGCCTCTCTTCCAACCTATTATTCTAACCACATTAACTATCACCACATTTATTTTTTTATCCTCCACCCACCTAGTGATTATATGAGTAGCACTAGAACTCAGTACACTAGTAGTCTTACCATTAATTGCTAACAAATCGCACCCTCGAGCAATTGAAGCCTCCACAAAGTATTTCCTAGCACAGGCCACCGCTTCTGCTCTGATCATCTTCTCAGGCACATTAAACTATATCGCCACAGGAAACTGTCAAATTACAGAAATAACAAACCAAACCCTAATAATTACCATAACTTTAGCTATGTTTATTAAAGTTGGATTAGTGCCGTTCCACTTCTGAGTGCCCGAAATTATTCAAGGAATAACCCCTACCGCCTCCATCTTTCTACTAACTTGGCAAAAACTAGGACCACTAATCTTACTTTTCTTAATAAGCCCGTTAATTTGCTTTGAAGTGATCTCTACGGTCTCTATTCTATCCGCCACAGTTGCTGGATGGCTAGGCCTAAACCAAACCCAAATTCGAAAGCTGGTAGCACTGTCTTCAATCGCCCAAATAGCTTGAACCATTGTGATTATTAAATATGCACCATCACTTACAATCCTGGCTTTCTATTTATACTCAATCACTATCTCCACAACACTTCTTACATTAGAAAAACTATCAACAACGTCAATTAATAGTCTTCTACTCTCATTCTCAAAAGCCCCAATCACCTCATCCCTACTAACCATTTCCTTAGTGTCTTTATCAGGCCTGCCACCACTAGCCGGATTCTTGCCAAAGTGGTTAACCATTGACCAGCTAGTGGCGGAAGGAGCAATTTGGGTTGCATTCACAATACTTATAGCCTCCCTTCTAAGCCTGGCCTTTTATTTACGTCTATGATATAACTCTGCATCAACCATCCCCCCAAACACCGTTAATACAAAACGCCTATGACGTAAATCTACTCAACAGACCAACCTTACAATCAACTTCATGGCCACAGCTGCCCTGACTCTAATCCTAGCAGCCACCCTAATAAAAGCTGTTACAAAACAAGAA------ACCAATTAAA----TAAACTTACTCACCCTGTTTATATTAGCCATGGCTACTGCAACAGCTGTAATTGCCCTAAACCTATTAATATCAGAACCCACCCCAGATTCAGAAAAACTCTCACCGTACGAGTGTGGGTTTGACCCATTAGGGTCAGCTCGCCTTCCACTATCTATCCGCTTTTTTATAATTGCCATTCTATTCTTATTGTTCGACCTAGAAATCGCCATCCTACTGCCACTAGCATGGGCACTCCAACTATCAAACCTGATTAAAACTACTGTATTAACCATCACTATCTTCTCACTCATATTTACGGGTCTAGCATATGAGTGACTACAAGGAGGTTTAGAATGAGCAGAATAAATGTTAAAAATTATTGTACCAACAGTAATATTAATTCCGTCCACCTGTTTAACCTCCACAAAAATTACCTGATTATCCCCAACAGCCTACTCAACAACTATTATTATCCTAAGTATAATCGTATTAAACCCAGGAGACTTCCTAGTAAGCACTAGCGGATTACTGCTAGGAAGTGACCAAATCTCAACACCACTGCTAATACTATCCTGCTGACTACTACCACTAATATTTATAGCTAGTCAAAGTTCAATATTACGTAACCCAACCTCACAAAAGCGGCTATTTATTACAGCCCTAGCCCTACTCCAACTAACATTGATATTAGTGTTTATGTCTTCAGACCTAATACTGTTTTATACAGCTTTCGAAGCAACCCTTATCCCCACCTTAATAGTAATCGCTCGATGAGGCTCCCAAACAGAACGACTAGGAGCAGGAATATATTTTTTATTATATACTATTACCAGCTCAATACCACTACTAATTGCAGTTTTGTGGATATATAACATAAAGGGCACTCTTTCCATTATTCTACTTCAACTTCTACCCCCAGTAGCCCTTATATTTTGAGCAAACACACTGCTATGAACCTCACTTATATTAGCCTTCTTAGTAAAAATCCCAATTTACGGGCTTCACCTATGACTACCTAAAGCCCACGTAGAGGCCCCAATTGCCGGATCCATAGTCCTAGCCGCCATCCTATTAAAACTAGGAGGTTATGGACTACTACGGGTCACAAACTTATTAACTGAACAAACCACATCCTATTATGTTCTCCCAATAACAGTAGCATTATGGGGTTCACTAATAACTGGTATAATCTGCCTACGACAAACAGACTTAAAGTCACTAATTGCATACTCCTCAGTTAGCCATATAGGATTAATAACAAGCTCGATTCTCACTCGTAACCAACTAGCCATGCCAGGTTCAATAACTGTAATAGTAGCCCACGGCCTTACATCCTCAATACTATTCTGCTTAGCAAATACTACTTATGAGCGGACACATTCACGAACCCTATTAATAGCACAAGGAGTACAACTCACTACCCCCACCATAGCCTCTTGATGATTACTAGCCTGCTTAATAAATATAGCACTACCCCCAACAATTAACTTTATTGGAGAATTTACCCTTATAGTATCACTATTTAATTGAGCTGATATTACCGCCATCTTAGCAGGAATAAGCGCGTTTATCACCTCAATTTATAGCCTACATATATTCTCCTCAACCCAACAAGGAACTCTTCCAAGCCACATCATCACACTAATCCCAACCCAAACACGAGAACACCTACTAATAACTATACACTCTGCGCCATCACTCGCCTTAATAACCACACCCCAGCTAATATATAACCAATAA---TTGACATCCACCCACCTTTTATTTGCCTTCTCCTTTATCTTATTTACCATTGGATTTACTTTCCGCCATACTCACCTTCTCTCCGCTCTTTTATGCTTGGAAGGCATAATACTATCAGCATTTATAATACTAGCAACATGATCCATAAACTCTCACACCTCCTCATTTATTTTGCCTTTAACAATCCTAACATTGTCAGCTTGCGAAGCTGGTATCGGCCTCGCACTCCTCATCTCCTCCGCCCGAACACATGACACAGCCAACCTCAAGAACCTAAACTTGCTTTTATGTTAAAT-AACCAAGCCTTCACCGCTCATT------CTATTTTTAATTCCGATACTAATCCTATCACTACCCGCACTACTGCCTACAT-CAAAATTGATCCGA--TACCCTATCATCAATGTGATAACAGCAAAGCTAGCTTTTTTCGCTAGCTTACTACCAACAGCCTATTTCATCTATACTGGTATAATCACCACTACACACGAGTTCCAACTGCTAACTATTGGTATAACCACAATCCAAATTAGTTTCGTACTAGATAGCTACTCCACCTTTTTCTTACCTATTCTCCTTTTTATTGTATGGACCATTATAGAATTTACCATACAATATATAAAATCAGACCTAGAAATTAACATCTTCTTTAATCAACTTACTATTTTTACCACAATGATAATAATCCTTTTAACTGCTGAAAACATTTTCCAGCTCTTCGTTGGTTGGGAAGGAGTAGGCATCATATCCTTTATGCTAATTAACTGATGATCCTTTCGATCAAATTCGAATAAAGCTGCCATACAAGCCATAATCTACAATCGCTTAGCTGACATTGGCTTAATAGTTACTATAGCATGAATAGTCATTAACAACATATCCCTAAGCATCAACGTAGTTCAAACCTCGCCAAAACTTGCCCTCATCCCCGCGCTAGGATTAGTCCTAGCAGCAGCAGGAAAATCCGCCCAATTTGGCTTCCACCCATGACTTCCTGCAGCAATAGAAGGCCCAACCCCTGTATCAGCCCTATTACACTCAAGCACTATAGTAGTAGCAGGCGTATTCCTGCTTATCCGAACATCAACCCTCCTATATAGCAGCGAAACGGCAACCATAATCTGCCTATTACTAGGAGGCATCACATCACTGCTGGCAGCCACATGCTCACTAGCCCAAAATGACCTAAAAAAAATCATTGCCTACTCAACCACCAGCCAACTGGGACTAATAATAGTTGCTATCGGGTTAAAACAACCTGAACTTGCATTTATGCACATCTCAACACACGCATTTTTTAAAGCAATACTATTCCTTTGCGCAGGGATAATTATCCACGGGTTAAACAACGAACAGGATGTCCGTAAAATAGGAGGACTCAAAAAAGCACTACCTATTACTTCCTCCTGTCTAGTTATTGGCTCCCTAGCCCTATCAGGTATGCCGTTCATAACTGGGTTTTACTCAAAAGATGCTATCATTGAATCTATTGGCACCTCCTATGTGAACTCACTGTCATTAGCTATAACCCTAATAGCAACTACCTTCACCACACTCTACAGTTTACGAATGGTTTACTATGTAGCCCTAAATACACCACGAATTTTAACATTATCTACCCTCCACGAAGATCCACAAATAGTCAACCCAATCCTACGACTAGCTCTTGGAAGCATCACAGCAGGCCTTCTAATCTCAGCCACTATTTTACCAACAAATATCCAACAACTAACAATGCCAGCATCAACCAAATTAGTCGCATTAAGTATCCTAGCATTAGGATTACTAATCGGATCAGCCCTCATCATAATAGCCAACCAACTCCCAACACCAATTAAGGGCGCCCAAACTCCCCTTCTTTTTAAAATCTTCCACTTCTACTTCATCCTACACCACACCATACCAGCCGCAATCCTAAAAATTAGCCAAAAGCTATCAACCCACCTGATGGACCAAACGCACTATGAAAACTTAGGCCCTAAAACAGCAGCCTATTTACAAATACTAGCAGCTAAAATATTAACTAGCTTTCACAAGGCCCAAATTAGCCCATATTTAAAAACCTTCATTACATTTATTGTACTGATTTTAATCATCTATTTTTCCTCAACGAACGCAAGG---------------------------------ATGGGAAATATATTTTTTATTCTTTGTTGCTTAATAATAATCAGTGTTGTATTGGTGGCCTTTGGGGTTACGATTCATTATGGGGTGGTTAGTTTGCTTTTTGCTACAATACTGGGTAGTAGTTTGCTGGTGGCAGGAGGGGGAAGTTTTATGCCGCTTGTAGTGTTGTTAATTTATTTAGGGGGGATGTTGGTGGTTTTTGCCTTTTGCGTGGGATTCACTGATGACAAGTATTGCGAGTTTTGAGGGTCGGGGGGGTCCAAGGGGCTGGCCTACATTTGATGGGCAGGGCTAGGTTTTGGGTGTTGTTGCGTGTATGATTCTATATGG----GTTGAATTTTTAGGACATCTCG--TTGATGTAGTAAATACTTGGAACAGTGAAGTAAGCAGTGAATCTTTGGGGGTCGGGCAATTTTATTTAAGTGGATGAGGGTTTCTTGTTTTAAGCGGTTGGGCGTTGTTGGTTGTATTATTTACTATTATGGCCCTAGTTCGGGGACAATATCAAGGGGCCTTGCGTTCGTTGAGG

*Osteolaemus tetraspis 2*

------------ACAAACCTAAATTTATTTGATCAATTCTCAATGCCTAAAATCCTTGGCATATCATTATTAACACCAGCCATATTATTAACAGCAATACTACTATATAATCCACAAGATCGTTGACTGTCAAACCCATTAACTACCCTACAATCATGATTAGTTGCGTATACTACTAAGCAAATTATAACTCCAGTGAGTAAACCAGGACACAAATGATCCCTGATACTGATATCCTTACTAACCCTTCTTATTCTTAACAACCTTCTCGGGCTCCTTCCATATACCTTTACACCAACAACTCAGCTATCCATAAACTTGGCCCTAGCCTTACCATTATGGCTGGCAACAGTATTAGTCGGCCTACGAAACAAGCCAACTTCCTCATTAGCCCACCTCCTGCCAGAAGGAACCCCAACACCACTAATCCCAATCTTAATTTTAATTGAAACAATTAGCCTACTTATCCGACCAATCGCACTCGCCGTGCGACTTACAGCCAACCTGACTGCAGGACATCTCTTAATTCACCTAATCGCTGCCACAGCCCTTAACTTGATAACAACATTCCCACTACTTACTGGACTAACCCTAATTGTCTTAACTTTACTAATACTCCTGGAAATTGCAGTAGCAATAATCCAGGCATACGTATTTACCCTATTACTCTCACTATACCTACAAGAAAACGTATAAATGCCACAATTAAACCCAGAGCCATGATTAACAATTCTATCTGTCACATGACTAGTACTTATTGTCATCTTACAACCAAAAATTGCCTCCTTAAAATTCATAAATAGCCCCACCCACCCAGACCAAAAAACTACT-AAACCATGACCCTGGCCACAAACCTAA---------------------ATGATCCACCAACTACGAAAATCCAACCCGCTATTAAAATTGATAAATAATTCCCTAATTGATCTGCCAACACCATCCAATATCTCCTATTGATGAAATTTCGGATCCCTGTTAGGATTCACCTTATTAATCCAACTAGCATCAGGCATTCTACTAATAATACACTTCCTAGCCGATGACACCATAGCTTTTATATCCGTGGCTTACACCTCACGAGAAGTATGATACGGATGACTAATTCGCAGCCTCCACGCAAACGGCGCCTCCCTATTTTTCCTATGCATTTTCCTTCACATGGGACGAGGACTATACTACGGATCATACCTACATGAAAACACATGAAATATTGGAGTACTACTACTATTATTACTAATAGCAACAGCCTTCATAGGATACGTCCTCCCATGAGGACAAATATCATTCTGAGGGGCAACTGTAATCACCAACCTTATATCAGCCATCCCATACATAGGAGACTCAATCGTAACCTGAATTTGAGGCGGACCATCTGTTAACAGCGCAACCCTAACACGATTCACCACCCTACACTTCCTACTCCCATTTATCCTAATAGCCGCCATCATCACACATCTAATCTTCCTTCACGAGCACGGGTCATTCAACCCACTAGGATTAACTTCCAACGCCGACAAAATTCCATTCCATCCATACTTCTCAACAAAAGACGCAGTAGGTGCAATCATAGCCACCATTATATTAACAACCCTGGCATTCTACTTCCCAAACCTTCTAGGAGACCCAGAAAACTTTACCCCAGCCGACCCCATAAAAACACCTGACCACATCAAGCCAGAGTGATATTTTCTGTTCGCCTACACAATCTTACGATCTATCCCAAACAAACTAATAGGAGTCATTGCCATATTCATGTCAATCTTGGTACTTCTACTCATACCCATACTTCACACATCAAAACAACAATCTATAAGCCTACGCCCACTATCACAACTTCTATTCTGAACTCTTACCGCAGACTTCCTCATTCTCACATGAATCGGGGGCCAACCAGTACAAGACCCATACATCTTAATCGGACAGATAGCCTCCTCCGTCTACTTCTTCACCATCCTCATCCTTCTACCACTAGCCGGGACAATTGAAAATATATTAATCAAACCACTTCAATACCGGCCAT----------GTGAATATTAACCGTTGACTTTTTTCCACCAACCATAAAGACATTGGCACCTTATACTTTATCTTCGGCGCATGAGCTGGAATAGTGGGAACAGCCCTAAGCCTTCTAATCCGGACAGAACTAAGCCAACCTGGCCCTCTTATAGGAGATGATCAGATTTACAATGTCATTGTCACAGCACATGCCTTTATTATAATTTTCTTTATGGTAATACCAATCATAATCGGAGGATTTGGAAACTGGCTACTGCCACTAATAATCGGAGCACCCGATATAGCATTTCCCCGAATAAATAACATAAGCTTCTGATTATTACCGCCCTCATTTATCTTACTTCTATTTTCCGCCTTCATTGAAACAGGCGCCGGTACAGGATGAACTGTTTACCCACCACTAGCTGGGAATCTGGCCCACGCTGGGCCATCAGTAGATTTAACTATCTTCTCCCTTCATCTTGCTGGGGTTTCATCAATTTTAGGGGCAATCAACTTTATCACCACAGCCCTTAATATAAAACCACCATCAATGTCACAACAACAAACACCACTTTTCGTATGATCCGTACTAGTCACGGCTGTACTCCTACTGCTCGCCCTACCGGTCCTAGCGGCAGGAATTACTATATTACTCACTGACCGAAACTTAAACACTACCTTTTTCGATCCTGCTGGAGGAGGGGATCCAATCCTATACCAACACCTCTTCTGATTCTTTGGCCACCCAGAAGTCTACATCCTCATCCTACCTGGATTTGGAATAATCTCTCATGTAATCACTTTCTACTCAAGTAAAAAAGAACCTTTCGGATATATAGGAATAGTGTGAGCCATAATATCAATTGGCTTCTTAGGCTTCATTGTATGAGCCCACCACATATTCACAGTAGGAATGGACGTGGACACTCGAGCATACTTTACATCCGCCACAATAATCATCGCCATCCCTACAGGTGTAAAAGTATTTAGCTGATTAGCCACCATTTATGGAGGAGTAGTAAAATGACAAGCCCCAATGCTCTGGGCACTTGGCTTTATTTTCCTATTTACAATTGGTGGATTAACAGGGATTGTTCTAGCCAACTCATCACTAGATATTATTCTCCACGACACTTACTACGTAGTAGCTCACTTCCACTATGTGTTATCCATAGGAGCAGTATTCGCCATCATAAGCGGATTCACCCATT-GATTCCCATTA-TTTACAGGGTTCACCCTTCATCACACATGAACCAAAA-TTCAATTCATGATTATGTTTGTAGGTGTAAACCTAACCTTCTTCCCACAACATTTTCTAGGCCTATCGGGGATGCCACGACGATACTCAGACTATCCAGACGCATATTCCTTCTGAAATACAACCTCCTCAATTGGCTCACTAATCTCTATAATTTCAGTTATTCTTCTCATATTTATTGTATGAGAGGCATTTTCATCGAAACGAAAAATTCAACTGCCAGAAATATCAACCACAAATGTAGAATGGCTAAACAACTGCCCGCCATCATACCACACCTACGAAGAGCCGGTATTTGTTCAAGTACAACCAAGCCTAATCTAT--------------------------------------GGCAAACCCAATACACCTGGGACTTCAAGATGCAATATCCCCATTAATAGAAGAACTCCTCTATTTCCACGACCACACACTAATAATCATTTTCTTAATCAGCATGTTTGTATTATACACAATTTCATTTTTACTGTTAACAAAACTATACAATACAAACGCCACAGACGTGCAAGAGATAGAAATAATTTGAACTATCCTGCCTGCCCTCATCTTAATCACCATTGCCCTACCATCCCTACGCACATTATACCTTATAGACGAGACCACCAACCCATGCCTGACCGTAAAAATTATTGGGCATCAATGATATTGAACCTACGAATACACAGATTTTTCCCCACTAGAGTTTGACTCTTACATACTTCCCACACAAGACCTTCCCCAAGGCCATTTTCGTCTCTTAGAAGTAGACCACCGCATAGTTATCCCAACAAATTCAAGCACTCGAGCCCTAGTCACAGCCGAAGATGTCCTACACTCTTGAGCAGTACCATCATTAGGAGTCAAAATGGACGCAGTACCAGGCCGCCTAAATCAAACCTCACTAACATCTCCAAATCCTGGAGTTTTCTATGG-CCAATGCTCAGAAATTTGTGGGGCAAATCATAGTTTTATGCCCGTTGTTGTAGAAGCTGTCCCAATACGACACTTCCAAAACTGATTAAAAACAAACACCT----AAATGACTCACCAAATACACCTTTTTCACATAGTTAACCCAAGCCCGTGACCCATCATAGGAGCCATAGCCGCCATAATATTAACAACCGGGTTAATCTTATGATTCCACTGTAATTCAAACCTAATCCTGCTACTGGGGTTGGTATCAACCATAGCAATTATATCTCAATGATGACGAGACATTATTCGAGAAAGCACCTACCTAGGCCACCACACACCGCCAGTACAAAAAGGACTACGATATGGTATAATCCTATTTATTACCTCAGAGGTCCTATTCTTCCTCGGGTTCTTCTGAGCATTCTATCATTCAAGCTTATCCCCAACCCCCGAGCTAGGAGGACAATGACCACCAACTGGAATCACCACCCTAGACCCATTTGAAGTCCCCCTGCTAAACACGGCAGTACTACTCGCCTCAGGAGTTACAGTAACGTGGGCCCACCACAGCCTAATAAAAGCCGACCGAAAATCCGCCATCCACGCCCTAATACTCACAATTATTTTAGGGTTATATTTTACTGCCCTTCAAGCAATAGAGTATTACGAAGCCCCATTCACCATCGCAGACAGCAGCTATGGGTCAACATTCTTTGTTGCCACAGGATTTCACGGCCTACATGTTATTATTGGATCGACATTCCTAACAGCCTGCCTATACCGACAAATCATATACCACTTCACCTCAAACCACCACTTTGGATTTGAAGCCGCCGCCTGATATTGACATTTCGTTGATGTAGTCTGACTATTCCTATATATTTCGATCTATTGATGAGGCTCTTATAAGCTTCCAAACAGCTGCCCCTATCATACTTTATATTATCTTAGTCCTGATTGCAGTTGCATTCCTAACAGGCCTGGAACGAAAAATCATCGGGTATATACAACTACGAAAAGGCCCTAACATTGTAGGACCACTTGGCTTGCTACAGCCATTTGCTGACGGACTCAAACTTATTATTAAAGAACTAACATTCCCCCTACTTGCCACCCCTGCTTTATTTATTTTATCCCCTGCAGTAGCCCTCATCCTATCATTAATTATATGAGCCCCCCTACCAGTGCCATTCTCCATTGCTGACCTAAACCTTGGCATATTATTTTTATTAGCAATATCAAGCTTGGCAGTCTATTCACTATTATGGTCCGGTTGAGCATCAAATTCTAAATACGCGCTAATAGGGGCCTTACGGGCAGTAGCCCAAACTATTTCATATGAGGTAACATTAGCTATTATTATTCTGTCTATTGCACTGCTGAGCGGTGGGTTTTCATTACACGCTCTCACCATCACCCAAGAACCCATCTTCATAGCATTAACCACATGGCCGCTACTAATAATATGATACACCTCAACACTAGCAGAGACAAACCGAGCTCCATTTGACCTAACAGAAGGAGAATCAGAACTAGTTTCTGGATTCAACGTTGAATATAGCGCAGGACTATTTACACTGTTTTTTCTAGCTGAGTATGCCAACATCTTACTAATAAACATTTTAACCACTGTCCTATTCCTCAATACATCAACAAATCTAACTACACAAGCACTATTTACTACCGCCTTAATGAGCAAATCTATTTTACTTACTATAGGCTTCCTATGAGTTCGAGCATCCTACCCGCGGTTCCGCTACGACCAATTAATACACCTATTGTGAAAAAATTTCCTACCAGCTACATTGGCAATCTGCCTATGACACTCATCACTGCCGACATCAATATTTGGTTTACCAGTAATAA---GGATGCCTCTCTTCCAACCTATTATTCTAACCACATTAACTATCACCACATTTATTTTTTTATCCTCCACCCACCTAGTGATTATATGAGTAGCACTAGAACTCAGTACACTAGTAGTCTTACCATTAATTGCTAACAAATCGCACCCTCGAGCAATTGAAGCCTCCACAAAGTATTTCCTAGCACAGGCCACCGCTTCTGCTCTGATCATCTTCTCAGGCACATTAAACTATATCGCCACAGGAAACTGTCAAATTACAGAAATAACAAACCAAACCCTAATAATTACCATAACTTTAGCTATGTTTATTAAAGTTGGATTAGTGCCGTTCCACTTCTGAGTGCCCGAAATTATTCAAGGAATAACCCCTACCGCCTCCATCTTTCTACTAACTTGGCAAAAACTAGGACCACTAATCTTACTTTTCTTAATAAGCCCGTTAATTTGCTTTGAAGTGATCTCTACGGTCTCTATTCTATCCGCCACAGTTGCTGGATGGCTAGGCCTAAACCAAACCCAAATTCGAAAGCTGGTAGCACTGTCTTCAATCGCCCAAATAGCTTGAACCATTGTGATTATTAAATATGCACCATCACTTACAATCCTGGCTTTCTATTTATACTCAATCACTATCTCCACAACACTTCTTACATTAGAAAAACTATCAACAACGTCAATTAATAGTCTTCTACTCTCATTCTCAAAAGCCCCAATCACCTCATCCCTACTAACCATTTCCTTAGTGTCTTTATCAGGCCTGCCACCACTAGCCGGATTCTTGCCAAAGTGGTTAACCATTGACCAGCTAGTGGCGGAAGGAGCAATTTGGGTTGCATTCACAATACTTATAGCCTCCCTTCTAAGCCTGGCCTTTTATTTACGTCTATGATATAACTCTGCATCAACCATCCCCCCAAACACCGTTAATACAAAACGCCTATGACGTAAATCTACTCAACAGACCAACCTTACAATCAACTTCATGGCCACAGCTGCCCTGACTCTAATCCTAGCAGCCACCCTAATAAAAGCTGTTACAAAACAAGAA------ACCAATTAAA----TAAACTTACTCACCCTGTTTATATTAGCCATGGCTACTGCAACAGCTGTAATTGCCCTAAACCTATTAATATCAGAACCCACCCCAGATTCAGAAAAACTCTCACCGTACGAGTGTGGGTTTGACCCATTAGGGTCAGCTCGCCTTCCACTATCTATCCGCTTTTTTATAATTGCCATTCTATTCTTATTGTTCGACCTAGAAATCGCCATCCTACTGCCACTAGCATGGGCACTCCAACTATCAAACCTGATTAAAACTACTGTATTAACCATCACTATCTTCTCACTCATATTTACGGGTCTAGCATATGAGTGACTACAAGGAGGTTTAGAATGAGCAGAATAAATGTTAAAAATTATTGTACCAACAGTAATATTAATTCCGTCCACCTGTTTAACCTCCACAAAAATTACCTGATTATCCCCAACAGCCTACTCAACAACTATTATTATCCTAAGTATAATCGTATTAAACCCAGGAGACTTCCTAGTAAGCACTAGCGGATTACTGCTAGGAAGTGACCAAATCTCAACACCACTGCTAATACTATCCTGCTGACTACTACCACTAATATTTATAGCTAGTCAAAGTTCAATATTACGTAACCCAACCTCACAAAAGCGGCTATTTATTACAGCCCTAGCCCTACTCCAACTAACATTGATATTAGTGTTTATGTCTTCAGACCTAATACTGTTTTATACAGCTTTCGAAGCAACCCTTATCCCCACCTTAATAGTAATCGCTCGATGAGGCTCCCAAACAGAACGACTAGGAGCAGGAATATATTTTTTATTATATACTATTACCAGCTCAATACCACTACTAATTGCAGTTTTGTGGATATATAACATAAAGGGCACTCTTTCCATTATTCTACTTCAACTTCTACCCCCAGTAGCCCTTATATTTTGAGCAAACACACTGCTATGAACCTCACTTATATTAGCCTTCTTAGTAAAAATCCCAATTTACGGGCTTCACCTATGACTACCTAAAGCCCACGTAGAGGCCCCAATTGCCGGATCCATAGTCCTAGCCGCCATCCTATTAAAACTAGGAGGTTATGGACTACTACGGGTCACAAACTTATTAACTGAACAAACCACATCCTATTATGTTCTCCCAATAACAGTAGCATTATGGGGTTCACTAATAACTGGTATAATCTGCCTACGACAAACAGACTTAAAGTCACTAATTGCATACTCCTCAGTTAGCCATATAGGATTAATAACAAGCTCGATTCTCACTCGTAACCAACTAGCCATGCCAGGTTCAATAACTGTAATAGTAGCCCACGGCCTTACATCCTCAATACTATTCTGCTTAGCAAATACTACTTATGAGCGGACACATTCACGAACCCTATTAATAGCACAAGGAGTACAACTCACTACCCCCACCATAGCCTCTTGATGATTACTAGCCTGCTTAATAAATATAGCACTACCCCCAACAATTAACTTTATTGGAGAATTTACCCTTATAGTATCACTATTTAATTGAGCTGATATTACCGCCATCTTAGCAGGAATAAGCGCGTTTATCACCTCAATTTATAGCCTACATATATTCTCCTCAACCCAACAAGGAACTCTTCCAAGCCACATCATCACACTAATCCCAACCCAAACACGAGAACACCTACTAATAACTATACACTCTGCGCCATCACTCGCCTTAATAACCACACCCCAGCTAATATATAACCAATAA---TTGACATCCACCCACCTTTTATTTGCCTTCTCCTTTATCTTATTTACCATTGGATTTACTTTCCGCCATACTCACCTTCTCTCCGCTCTTTTATGCTTGGAAGGCATAATACTATCAGCATTTATAATACTAGCAACATGATCCATAAACTCTCACACCTCCTCATTTATTTTGCCTTTAACAATCCTAACATTGTCAGCTTGCGAAGCTGGTATCGGCCTCGCACTCCTCATCTCCTCCGCCCGAACACATGACACAGCCAACCTCAAGAACCTAAACTTGCTTTTATGTTAAAT-AACCAAGCCTTCACCGCTCATT------CTATTTTTAATTCCGATACTAATCCTATCACTACCCGCACTACTGCCTACAT-CAAAATTGATCCGA--TACCCTATCATCAATGTGATAACAGCAAAGCTAGCTTTTTTCGCTAGCTTACTACCAACAGCCTATTTCATCTATACTGGTATAATCACCACTACACACGAGTTCCAACTGCTAACTATTGGTATAACCACAATCCAAATTAGTTTCGTACTAGATAGCTACTCCACCTTTTTCTTACCTATTCTCCTTTTTATTGTATGGACCATTATAGAATTTACCATACAATATATAAAATCAGACCTAGAAATTAACATCTTCTTTAATCAACTTACTATTTTTACCACAATGATAATAATCCTTTTAACTGCTGAAAACATTTTCCAGCTCTTCGTTGGTTGGGAAGGAGTAGGCATCATATCCTTTATGCTAATTAACTGATGATCCTTTCGATCAAATTCGAATAAAGCTGCCATACAAGCCATAATCTACAATCGCTTAGCTGACATTGGCTTAATAGTTACTATAGCATGAATAGTCATTAACAACATATCCCTAAGCATCAACGTAGTTCAAACCTCGCCAAAACTTGCCCTCATCCCCGCGCTAGGATTAGTCCTAGCAGCAGCAGGAAAATCCGCCCAATTTGGCTTCCACCCATGACTTCCTGCAGCAATAGAAGGCCCAACCCCTGTATCAGCCCTATTACACTCAAGCACTATAGTAGTAGCAGGCGTATTCCTGCTTATCCGAACATCAACCCTCCTATATAGCAGCGAAACGGCAACCATAATCTGCCTATTACTAGGAGGCATCACATCACTGCTGGCAGCCACATGCTCACTAGCCCAAAATGACCTAAAAAAAATCATTGCCTACTCAACCACCAGCCAACTGGGACTAATAATAGTTGCTATCGGGTTAAAACAACCTGAACTTGCATTTATGCACATCTCAACACACGCATTTTTTAAAGCAATACTATTCCTTTGCGCAGGGATAATTATCCACGGGTTAAACAACGAACAGGATGTCCGTAAAATAGGAGGACTCAAAAAAGCACTACCTATTACTTCCTCCTGTCTAGTTATTGGCTCCCTAGCCCTATCAGGTATGCCGTTCATAACTGGGTTTTACTCAAAAGATGCTATCATTGAATCTATTGGCACCTCCTATGTGAACTCACTGTCATTAGCTATAACCCTAATAGCAACTACCTTCACCACACTCTACAGTTTACGAATGGTTTACTATGTAGCCCTAAATACACCACGAATTTTAACATTATCTACCCTCCACGAAGATCCACAAATAGTCAACCCAATCCTACGACTAGCTCTTGGAAGCATCACAGCAGGCCTTCTAATCTCAGCCACTATTTTACCAACAAATATCCAACAACTAACAATGCCAGCATCAACCAAATTAGTCGCATTAAGTATCCTAGCATTAGGATTACTAATCGGATCAGCCCTCATCATAATAGCCAACCAACTCCCAACACCAATTAAGGGCGCCCAAACTCCCCTTCTTTTTAAAATCTTCCACTTCTACTTCATCCTACACCACACCATACCAGCCGCAATCCTAAAAATTAGCCAAAAGCTATCAACCCACCTGATGGACCAAACGCACTATGAAAACTTAGGCCCTAAAACAGCAGCCTATTTACAAATACTAGCAGCTAAAATATTAACTAGCTTTCACAAGGCCCAAATTAGCCCATATTTAAAAACCTTCATTACATTTATTGTACTGATTTTAATCATCTATTTTTCCTCAACGAACGCAAGG---------------------------------ATGGGAAATATATTTTTTATTCTTTGTTGCTTAATAATAATCAGTGTTGTATTGGTGGCCTTTGGGGTTACGATTCATTATGGGGTGGTTAGTTTGCTTTTTGCTACAATACTGGGTAGTAGTTTGCTGGTGGCAGGAGGGGGAAGTTTTATGCCGCTTGTAGTGTTGTTAATTTATTTAGGGGGGATGTTGGTGGTTTTTGCCTTTTGCGTGGGATTCACTGATGACAAGTATTGCGAGTTTTGAGGGTCGGGGGGGTCCAAGGGGCTGGCCTACATTTGATGGGCAGGGCTAGGTTTTGGGTGTTGTTGCGTGTATGATTCTATATGG----GTTGAATTTTTAGGACATCTCG--TTGATGTAGTAAATACTTGGAACAGTGAAGTAAGCAGTGAATCTTTGGGGGTCGGGCAATTTTATTTAAGTGGATGAGGGTTTCTTGTTTTAAGCGGTTGGGCGTTGTTGGTTGTATTATTTACTATTATGGCCCTAGTTCGGGGACAATATCAAGGGGCCTTGCGTTCGTTGAGG

*Crocodylus cataphractus*

ATGACCTTGGCCACAAACCTAAATTTATTTGACCAGTTCTTAATACCCAAACTACTTGGCGTATCATTATTAATTCCAGCCATACTACTAACAGCAACACTACTATATAATCCACAAGATCGCTGACTATCAAACCCTTCAACAACCCTACAATCCTGATTAATTGCAAAAACCACTAAACAAATTATAACCCCAGTAAATAAAACAGGGCACAAATGATCATTAATATTAATATCTCTACTGACACTACTCATTCTTAACAACCTTTTAGGACTTCTTCCATATACATTTACACCAACAACCCAACTATCCATAAACCTATCCCTAGCCCTACCACTATGGCTGGCAACAGTACTGATAGGCCTGCGAAACAAACCAACTACCTCATTAGCCCATCTTCTCCCAGAAGGTACCCCAACACCCCTAATCCCAATCCTGATTTTAATCGAAACAATCAGCCTATTCATCCGACCAATCGCACTCGCCGTACGACTCACAGCTAACCTAACTGCAGGACACCTATTAATGCACCTTATCTCTGCTGCAGCACTCAACTTAATAACAATATCCCCACTACTCACTGGACTAACCTTAATTACCCTAACCCTATTAGTATTACTAGAAGTTGCAGTAGCAATAATCCAAGCATACGTCTTCACCCTCTTGCTCTCCTTATACCTGCAAGAAAACGTATAAATGCCACAACTAAACCCAGAACCCTGATTAATAGTATTATCTGTCACATGATTAGTACTTATCATTATCTTACAACCAAAAATTGCCTCATTAAAATTTACAAATAACCCAACCAACCCAGACCAAAAAACCACC-AAAGCATGACCTTGGCCACAAACCTAA---------------------ATGGCCCACCAACTACGAAAATCCCACCCGCTACTAAAACTAATAAACCACTCCCTAATCGACCTACCAACACCATCAAACATCTCCTATTGGTGAAACTTCGGGTCCCTGCTTGGATTCACCCTACTAATCCAACTAACATCCGGAATCTTACTAATAATACACTTCCTAGCTGACGACACACTAGCTTTCACATCAGTTGCCTACACCTCACGAGAAGTTTGATATGGCTGACTAATCCGCAGCCTTCACGCAAACGGAGCCTCTCTATTCTTCTTATGCATCTTCCTTCATATTGGACGAGGACTATACTACGGATCATACCTCAATGAAAACACATGAAATATCGGAGTACTGCTACTATTACTACTAATAGCAACAGCCTTCATAGGCTATGTCCTACCATGAGGACAAATATCATTCTGAGGGGCAACCGTAATTACTAACCTCATATCAGCCATCCCGTACATTGGAAACTCAATCGTAGCCTGAATTTGAGGGGGGCCATCTGTCAGCAGTGCAACCCTTACACGATTCACCACCCTACACTTCCTCCTTCCATTTATCCTAATAGCTGCCGTCATCACCCACCTTATTTTTCTTCACGAACGCGGATCATTCAACCCATTAGGATTAACCCCCAACGCTGACAAAATTCCGTTCCACCCATACTTCTCAACAAAAGATGCCATAGGGGTAATTCTAGCCACCACTCTATTAATAACACTAGCATTATACCTCCCAAACTTACTAGGAGACCCACAAAACTTCACCCCGGCCGACCCCATAAAAACACCAGACCACATTAAACCAGAATGATACTTCCTATTCGCCTACACAATCCTACGATCAATCCCAAACAAACTCACAGGAGTTACTGCCATATTCATATCAATCCTAGTACTACTACTCCTACCAGCACTACACACATCAAAACGACAATCTATAAGCCTACGCCCACTATCCCAACTCCTATTCTGAACCCTAATCGCAGATTTCTTTATTCTCACATGAATTGGGGGACAACCAGTACAAGACCCATACATTTTAATTGGACAAATAGCCTCCTCACTCTATTTCTCAACCATCCTTATCCTCCTACCACTAGCAGGAACAATTGAAAACCTAATGATCAAACCTCTTCAATACCGACCATACGGGCCATGGTGAATATTAATCGTTGACTTTTTTCCACCAACCACAAAGATATCGGCACCTTGTATTTTATCTTCGGCGCCTGAGCTGGAATAGTGGGGACAGCCCTAAGCCTCCTAATCCGGACAGAACTTAGCCAGCCAGGTCCATTTATGGGAGATGACCAAATCTACAATGTCGTCGTTACAGCACACGCCTTTATCATAATCTTTTTTATAGTAATACCAATCATGATCGGAGGATTTGGAAACTGACTACTACCATTAATGATTGGAGCACCAGACATAGCATTCCCTCGAATAAATAACATGAGCTTCTGATTACTCCCCCCTTCATTCACCTTACTCTTATTTTCCGCCTTTATTGAAACCGGTGCCGGCACCGGTTGAACAGTCTACCCACCACTAGCTGGAAACCTGGCCCACGCAGGACCATCAGTAGACCTAACTATCTTCTCCCTTCACCTTGCTGGAGTATCATCAATCTTAGGGGCAATTAACTTCATTACCACTGCTATTAACATAAAACCCCCATCAATATCACAACAACAAACACCACTTTTTGTGTGATCTGTATTAGTCACTGCTGTACTTCTACTGCTAGCCCTACCAGTCCTAGCTGCAGGCATTACTATACTGCTTACTGACCGAAACTTGAACACCACCTTCTTTGACCCTGCAGGAGGAGGAGACCCAATCTTATACCAACACCTTTTCTGATTTTTCGGCCACCCAGAAGTCTACATCCTCATCCTACCAGGGTTTGGAATGATCTCCCATGTTATTACTTTCTACTCAAGTAAAAAAGAACCATTTGGATATATGGGGATAGTATGAGCTATGATATCAATTGGATTCTTAGGTTTCATTGTCTGAGCCCACCACATATTTACAGTAGGAATAGATGTCGATACCCGCGCATACTTCACATCCGCCACAATAATTATCGCCATCCCTACAGGAGTAAAAGTATTTAGCTGATTAGCCACCATTTATGGGGGAGTAGTGAAATGACAAGCCCCAATGCTTTGAGCACTTGGCTTCATCTTCCTATTCACAGTTGGGGGGCTAACAGGAATTGTCTTAGCCAATTCATCACTAGACATTATTCTCCACGACACTTACTACGTAGTAGCCCACTTCCACTATGTATTATCAATAGGAGCAGTCTTCGCCATCATAAGCGGATTTACCCACT-GATTCCCACTA-TTTACAGGATTTACCCTTCACCACACATGAACAAAAA-TTCAATTCATAATTATATTTGTAGGAGTAAACCTAACCTTCTTCCCACAGCACTTCCTTGGCCTATCAGGAATGCCACGACGATACTCAGACTACCCAGATGCATACTCTTTCTGAAACATAATCTCCTCAATTGGGTCCCTGATCTCTATAATCTCAGTTATTCTACTTATATTTATTGTATGAGAAGCATTTTCATCAAAACGAAAAGTCCAAGTGCCTGAAATATCAACCACAAATGTAGAATGGCTAAATAACTGCCCACCATCATATCACACATACGAAGAGCCAGTATTTGTTCAAGTACAACCAAGCCTAATTTATCGATGATTGTACATAAAAAACAAAGACCTATACTAAATGGCAAACCCAATACACTTAGGACTCCAAGACGCAATATCCCCACTAATAGAAGAACTCCTATACTTCCACGACCACACGCTAATAATTATTTTCTTAATCAGCATATTTGTACTATATACAATCTCAGCTCTACTACTAACAAAACTATACAACACAAACGCCACAGACGTACAAGAAATAGAAATAGTTTGAACCATCCTGCCAGCCCTCATTTTAATTACTATTGCCCTACCATCCCTGCGCACACTTTATCTTATAGATGAAACCACCAACCCGTGCCTAACCATTAAAGTCATTGGGCACCAGTGGTATTGAACCTATGAATACACAGATTTTTCTCAATTGGAATTTGACTCTTATATACTCCCCACACAAGACCTGCCCCAGGGTCATTTCCGCCTTCTAGAAGTAGACCACCGCATGATCGTTCCAACAAACTCAAGCACCCGAACACTAATCACAGCTGAAGATGTCCTCCACTCATGAGCAGTCCCATCATTAGGAATTAAAATGGACGCAGTACCAGGCCGCCTAAATCAAACCTCACTAACATCTCCAAACCCTGGAATTTTCTACGG-CCAATGTTCTGAAATTTGTGGAGCAAACCACAGCTTTATGCCTATCGTCGTAGAAGCTGTCCCAATACAACACTTCCAAAACTGATTAAAAACAAACACCT----AAATGATCCACCAAACACACCCGTTTCATATAGTTAACCCAAGCCCATGGCCCATCATGGGGGCTATAGCTGCCATAATATTAACAACAGGATTAGTCCTATGATTTCACTGCAATTTAAGCCTGGTTTTACTTATAGGACTAGTATCCACCATAACAATTATGTTTCAATGATGGCGGGATATTATCCGAGAAAGCACCTACCTAGGCCACCACACCCCACCAGTGCAAAAGGGGCTACGATACGGCATAATCCTATTCATCACCTCAGAAGTCTTCTTCTTCATCGGGTTCTTCTGGGCATTCTATCACTCAAGCCTTTCACCAACCCCAGAGCTTGGAGGACAGTGGCCACCAACTGGAATTACCACACTAGACCCATTCGAAGTACCACTCCTAAACACAGCAGTACTACTAGCCTCGGGAGTTACAGTAACATGAGCCCACCACAGCCTAATGGAAGCAAACCGAACACCAACCACCCACGCCCTAATACTTACAATTATTCTAGGGTTGTATTTCACTGCCCTACAAGCAATAGAATATTATGAGGCCCCATTTACCATCGCAGACAGTAGTTATGGGTCTACCTTCTTTGTCGCCACAGGCTTTCACGGCCTACATGTTATTATCGGATCAACTTTTCTAATAACCTGCCTATACCGACAGATTATACACCACTTCACCTCAAACCACCACTTTGGATTCGAAGCCGCCGCCTGATATTGACATTTCGTAGATGTAGTCTGACTATTCCTATACATCTCTATCTATTGATGAGGATCTTATTAGTTTTATAACGGCTGCCCCAATCATACTTTATATCATTTTAATCCTAGTTGCAGTTGCATTCCTTACTGGCCTAGAACGAAAAATCATTGGTTACATACAACTACGAAAGGGGCCTAATATTGTAGGGCCACTGGGCCTACTACAACCATTTGCTGATGGCCTCAAACTTATTATCAAAGAACTAACACTCCCCCTACTCGCCACCCCAACCCTATTTGTTTTGTCCCCTGCAGTGGCCCTCATCCTATCCTTAATTATGTGAGCACCCTTACCCGTACCATTCTCCATCGCCAACCTAAACCTTGGCATACTGTTCTTACTAGCCATATCTAGCTTAGCAGTCTATTCCTTATTATGATCCGGCTGAGCATCAAACTCTAAATACGCACTAATAGGGGCATTACGGGCAGTAGCCCAAACTATCTCATATGAAGTTACACTAGCCATCATTGTCCTATCCATTGTGCTGCTCAGTGGCGGATTTTCACTACACGCACTCACCATTACCCAAGAACCGCTCTTCCTAGCATTAACCACATGACCTTTACTAATAATATGGTATACCTCAACACTAGCAGAAACTAACCGCGCCCCATTTGACTTAACAGAAGGTGAGTCAGAACTAGTATCCGGATTCAACGTAGAGTACAGCGCAGGATTATTCACACTGTTCTTTTTGGCCGAATACGCCAACATCTTATTGATAAATGTTTTAACCACCATCCTGTTCCTAAACATACCTATAAATCTACCAACACAAACACTATTCACCGCAGCTTTAATAGGCAAAGCTATCACGCTAACCGTGGGTTTTCTCTGGGTACGAGCATCATACCCACGATTCCGCTATGACCAGCTAATACACCTCCTATGAAAAAGCTTCTTACCCGTCACATTAGCAATATGCCTATGACACTCAACACTACCAATATCAATATTTGGTCTACCGGTAACAA---GGATGCCCATCTTCCAACCCATTATTTTAACCACACTAACCATCACCACATTCATTTTTCTATCCTCCACCCACCTAGTACTTATGTGAGTATCACTGGAACTTAGCACGCTAGTAGTCTTACCACTAATTGCCAATAAATCACACCCCCGAGCAATTGAAGCCTCCACAAAATACTTCCTGACACAAGCCACCGCTTCTGCACTAATAATCTTCTCAGGGACATTAAACTATATTACCACCGGAAACTGTCAAATTATAGAAATAACAAACCAAACCCTAATAATCATTATAATCCTAGCCATGTTTATTAAAGTCGGATTAGTGCCTTTCCACTTCTGGGTGCCAGAAACTATTCAAGGAATATCCCCAACCGCCTCAATCTTCCTGCTAACCTGGCAAAAGCTGGGCCCACTAATCTTATTATTCCTAATAAGCCCATTAGTTAATTTTGAAGTAATCTCTGTGGTATCTATCCTATCTGTTACAGTTGCTGGGTGACTCGGACTCAACCAAACCCAGATCCGTAAACTGGTAGCACTCTCCTCAATCGCTCAGATAGCTTGAACCGTTATTATCATCAAATATGCACCATCACTTACAATTCTAGCTTTCTATGTATACTCAATTACCATTTCCACAACCCTACTCACGCTAGAAAAGCTATCAACAACCTCTGTTAGCAGCCTGCTACTTTCATTCTCAAAAGCCCCAATTACCTCATCATTACTGACAATCTCCCTACTATCCCTGTCAGGACTTCCACCACTAGCCGGATTCCTCCCAAAATGATTAACCATTGACCAACTAGTGGCAGAAGGGGCAATTTGAATTACATTCGTAATGCTCATGGCTTCCCTTCTAAGCCTATTCTTCTACCTACGTTTGTGATACAACTCCGCATCCACCCTGCCACCAAACGCTACCAACACCAAACGACTATGACGTAAATCAACCCAACAAACCAACCTTACAATCAACTCAATAGCCACAGCCGCTATAACCCTAATCCTAGCAGCCACCTTAATAAAAGCCATTACAAAACAAGAC------ATTAATTAAA----TAAATTTACTTACCTTATTTATACTAGCCATGGCTACTGCAGTAGTTGTAATTACCATAAACCTATTAATATCAGAGCCCGCCCCAGACTCAGAAAAACTCTCACCATACGAATGTGGATTTGACCCATTAGGATCAGCCCGCCTACCGCTATCTATTCGTTTTTTCATGGTCGCCATTCTATTTTTACTATTCGACTTAGAAATCGCTATCCTACTACCACTAGCATGAGCACTTCAACTCTTAAACCTTATTAAAACTGTCACACTAGCAATTATCATCTTCTCACTTATATTTATAGGACTAGCATATGAATGACTACAAGGCGGATTAGAGTGGGCAGAGTAAATGCTAAAAATCATCGTACCAACAATAATATTAATTCCATCCACCTGTTTAACAGCCACAAAAAACACCTGACTGTCACCAACAGCCTACTCGGCAATTATTATCGTCATAAGCATGCTTGTATTAAATCCAGGAGATGCCCTAACAAACACTAGCGGATTACTACTGGGAAGCGACCAAATCTCAACACCTCTACTAATTTTATCCTGCTGACTACTGCCAATAATATTCATAGCCAGTCAAAGCTCAATATTACACAACCCAACCCCACAAAAACGACTTTTTATTACAGCCCTAGCCCTTCTACAATTAGCATTAATATTAGTATTCATAGCTTCAGACCTAATACTGTTTTATACTGCTTTCGAAGCAACCCTTATCCCTACCCTATTAGTAATTGCTCGGTGAGGCTCCCAAACAGAACGATTAGGGGCGGGGATATACTTTCTTCTATATACCATTACCAGCTCTATGCCTCTCCTAATCGCAATTCTATGGGTATATAATATAAAGGGAACACTTTCTATTATCCTACTACAACTCATACCCCAAGTAACCATCATATTCTGAACAAATACATTATTATGAACATCACTTATATTAGCCTTCTTAGTAAAAATTCCAATTTACGGACTTCACCTATGACTACCAAAAGCCCATGTAGAAGCCCCAATTGCCGGATCCATAGTCCTAGCCGCGATCTTATTAAAACTAGGGGGCTATGGACTTTTACGAGTCACAAACCTATTAACCGAACAAACCACTCCCTCATATATCCTGCCACTAACAGTGGCACTATGAGGTGCTCTCATAACTGGAATAATCTGCCTTCGACAAACAGACTTAAAATCACTAATTGCCTACTCTTCAGTTAGTCATATAGGACTAATAACAAGCTCAATCCTCACTCGTAACCAACTAGCCCCGTCTGGTTCGATAATCATAATAGTAGCCCATGGCCTCACATCCTCAATACTATTCTGTTTAGCAAACATCACTTACGAACGAACACACTCACGAACCCTTCTACTAACACAAGGAGTACAGCTCACCACCCCCGCCATAACCTCCTGATGACTGCTAGCCTGTTTGACAAACATAGCACTACCCCCAACAATCAACTTCATTGGAGAACTCACTCTTATAGTGTCCCTATTTAACTGAGCAGACATTACCGTCTTTCTAACAGGACTAAGTGCATTCATCACCTCAATTTACACCCTCCACATATTCTCATCAACCCAACAAGGAACCCTACCAAGCCACATCATTACAATAAGCCCAACCCAGACACGAGAACACTTAATCATAGCCATGCACTCAGCACCGTCACTCGCCTTGATCGCCACACCCCAACTAATATACAACCAATAA---ACCTCTCCAACTGACTTCTTATTCGCCCTATCCTTCATTTTATTCACCATCGGATTTACTTTCCGCCATACTCATCTTCTCTCGGCCCTTCTATGTCTAGAAGGCATAATATTGTCAGCATTCATAATATTAGCAACATGGTCTATAAACTCCCACACCTCCTCTTTTGTTCTGCCACTAATAATCCTAACACTATCAGCATGCGAAGCCGGTATTGGACTAGCTTTACTAATTGCCTCGGCCCGAACGCACAATACAGCTAATCTCAAAAACCTAAATTTACTCCAATGCTAAAT-AGCACAGACATCAATGTTAACCATAACACTATTTATACTCCCACTATTTATCCTAGCGTTACCCACACTAACTCCAACAT-CAAAACTAATCCCA--CATCCAAGTACCAATGTACTTGCAGCAAAACTAGCTTTCTTCACCAGCCTATTTCCCCTAGCCCACTTCATCCACACTGGCATAACCACCACCACATGCGAAACCCAACTATTAACAATAGGCACAACCACAATTCAAGTCACCCTAACGCTAGACAGCTACTCCACATTCTTCATACCAATCCTCCTTTTTGTCGTGTGGTCTATTATAGACTTCACCGTACAATACATAAAACCAGACCTAAAAGTCAGCATCTTCTTTAGTTATCTAATCACTTTCACCATAATAATAATAATTCTTGTAACTGCCGAAAACATGTTCCAGTTCTTCATTGGTTGAGAAGGAGTGGGTATTATATCATTTATATTAATTAACTGATGATCTTTTCGATCAAGCTCGAACAAAGCCGCCATACAAGCCGTGGTCTATAATCGCCTAGCTGATATTGGCCTAATCATTACTATAGCGTGAATAGTCATTAACAACACCTCCTTAAGCATTAAAGGAGTACAAGCCCCACCAGACCTCGCCCTAATCCCAGCACTTGGATTAGTCCTAGCAGCCGCAGGAAAATCCGCCCAATTCGGATTCCACCCCTGGCTCCCCGCAGCAATAGAAGGCCCAACCCCTGTATCAGCCCTACTACACTCAAGCACCATGGTGGTAGCCGGCGTATTCCTGCTTATCCGAACCTCCGACCTCCTATATAGCAGCGAAACAGCAACTACAATCTGTTTATTCCTAGGGGCCATTACATCATTGCTAGCAGCCACATGTGCGCTAGCCCAAAATGACCTAAAAAAAATTATCGCCTACTCAACCACCAGCCAACTAGGACTTATAATGATTTCAATTGGACTAAAACAACCCGAACTCGCATTCATGCACATCTCAACACACGCATTCTTTAAAGCAATACTATTCCTCTGTGCAGGAACAATCATTCACAACCTAGACAATGAACAAGACATCCGAAAAATAGGAGGACTCAAAAAAGCACTACCAATTACCTCCTCCTGCTTAGTCATTGGATCTCTAGCCCTATCAGGAATGCCTTTCATGGCCGGGTTCTATTCAAAAGACGCTATTATTGAATCCATTAACACCTCCAATGTAAACTCCCTATCACTAGCCATGACCCTAATAGCAACCATCCTTACCACATTCTACAGTCTACGAATAATCTACTATGTAGCCCTAAATACACCACGAATCTTGCCACTATCCTCAATTTCCGAAAACCCACAAACAACCAACCCAATCCTACGATTAGCCGTCGGAAGCATCGCAGCCGGTCTTCTAATTTCATCCACTATTTTACCAACAAACATTCCACAGCTAACCATACCAGCATCAGCCAAACTAGCCGCATTAGGTATCCTAATACTAGGCCTACTAGTTGGATCAGCCCTAGTTATAATAGCTAACCAATTACCAACATCAATCAAAGGCTCCCAAACCCCCCTTCTCTTTAAAATTATACACTTCTACTTTATTTTACATCACACCCTACCGACCGCAATCTTACAAATTAGCCAAAAACTATTAACCCACCTAATAGACCAAACACATTACGAAGCCTTGGGCCCTAAAGCAGCAACCTACCTACAAATACTAATATCTAAAATCCTAACCAACTTCCACAAGGCCCGAATCAACCCATACTTAAAAATTATTATTATATCTATTATACTAATCTCAATACTATACTTTTCCTCAATGAGCGTAGAGCCCCCTGATATCGGCCACGAACCAGGGCCATAAATGGGAACTATGCTTTTTTTTCTTTGTTGTTTAATAATGGTAAGTGTGGTACTGGTGGCTTTTGGGGTTACAATCCACTATGGAGTGGTTAGTTTGTTGTTTGCAGCAATGTTTGGTAGTGGTTTATTGGTGGCAGGTGGAGGGAGTTTTGTGCCTCTTGTAGTCCTATTAATCTATTTAGGGGGCATGTTGGTGGTTTTTGCTTTTTGTGTGGGGTTTACTGATGATGAGTACTGTGAGTTTTGGGGGGCAGGAGGCTCTAAGGGGTTGGCCTGTGTTTGTTTAGTGGGATTGAGTATTGGGTATTATTATGTATATGATTTTACATGA----TTGGGGTTTATGGGATATTTCG--TTGATGTGTTTGAGATTTGAAGTGGTGGGGTGAGTGGTGAGTCATTAGGCGTTGGCCTGTTTTATTTAAGTGGGTGGGGATTTCTTGTTCTGAGCGGTTGGGCTTTATTGGTTGTCTTATTTACTATTATGGCCCTGGTTCGTGGCCGATATCAGGGGGCTCTACGCTCATTGAGG

*Gavialis gangeticus*

ATGACCTTGGCCACAAATCTAAGCTTATTTGATCAATTTTCAATCCCTAAACTCCTAGGAATCCCCATACTAATCCCCGCCATATTAATAACAAGCATGTTATTATATAACCCAGAAGACCGATGATTGTCTAACCCAACAACAACCATCCAATCCTGATTAATTGCAAAAACAACTAAACAAATTATAGCCCCAGTGAATCAACCAGGACACAAATGATCCCTAATATTGATTTCGCTACTAACCTTTCTTCTTATCAATAACCTCCTAGGACTACTCCCATATACTTTTACACCAACAACGCAACTATCTATAAACCTAGCCTTAGCCCTACCCTTATGAATGGCAACAGTATTAATTGGGTTACGAAATAAACCAACCGCCTCCTTAGCTCACCTACTTCCAGAAGGAACTCCAACACCCCTAATTCCAATCTTAATTTTAATTGAGTCCATTAGTCTATTAATCCGACCAATCGCTCTTGCCGTACGACTAACAGCCAACCTAACCGCCGGACACCTTTTAATTCACCTAATTGCCGCCACAGCGCTGAACCTACTAATAACATCCATACTACTAGCTGCACTGACCCTAACTATTTTAACACTACTAACGCTACTAGAAATTGCAGTAGCAATTATTCAAGCCTATGTTTTTACCCTACTCCTCTCCCTCTACCTACAAGAAAATGTATAA-TGCCCCAACTAAACCCAAAACCCTGACTTATGGTTTTCCTTACCACTTGATTTATATTAATTATTGCTACACACCCAAAAATTGCCTCCCTAAAATTTATAAATAACCCAGCACCCTTTCACCCCACACCAATT-AAACCATGACCTTGGCCACAAATCTAA---------------------ATGGCCCACCAACTACGAAAATCCCACCCCCTACTAAAACTTGTAAACCACTCATTAATTGACCTCCCCACCCCATCCAACATCTCCTATTGATGAAACTTCGGATCACTACTAGGATTCACCCTTTTAATCCAAATCCTATCAGGAGTTTTACTAATAATACACTTCCTAGCAGATGACACTATGGCCTTCGCATCTGTTGCTTACACCTCACGAGAAGTTTGATATGGTTGACTTATCCGAAGCCTCCACGCAAACGGAGCCTCCCTCTTCTTCCTATGCATCTTCTTACACATTGGACGTGGCTTATATTACGGCTCTTACCTACATGAAAATACATGAAACATTGGAGTCATCCTGCTCCTCTTATTAATAGCGACAGCATTCATAGGCTATGTCCTACCCTGAGGACAAATATCATTCTGAGGGGCAACCGTAATTACAAACCTCATGTCAGCCATCCCATATATTGGAGACTCCCTCGTAATCTGAATCTGAGGAGGCCCTTCAGTTAACAGCGCCACCCTAACACGCTTTACTACCTTACACTTTCTACTCCCATTCATTCTACTAGCAGCCATCATCACCCACCTCATATTTCTACATGAACAAGGCTCATTTAACCCCTTAGGACTGCCTCCCAACACCGACAAAGTCCCATTTCACCCATACTTCTCCCTAAAAGACGCTATAGGGATAATACTCGCCGTCACCTTACTTTCAACTTTAGCCCTATATCTCCCAAACCTCTTAGGAGACCCAGAAAACTTCACACCAGCTGACCCCATAAAAACCCCTAACCACATCAAACCCGAATGATACTTCCTATTCGCCTACACAATCCTACGATCCATTCCCAATAAGCTAATAGGAGTATTAGCTATATTTTCATCAATCCTAATTCTCTTCCTAATGCCCATGCTTCACACATCAAAACGACAATCAATAGCCATGCGCCCCCTATCCCAACTTCTATTTTGAACACTCGTCGCCGACTTCTTTGTATTAACCTGGATCGGAGGACAGCCCGTACAAGACCCCTACACACTAATCGGCCAAATAGCCTCCATAACCTACTTCACAATCATCCTTATTCTCATACCACTAACAGGCCAAGTAGAAAACTGAATTATTAAACCTCTACGATACCGAT-------------GTGAACATAAATCGCTGACTTTTCTCCACCAATCACAAAGACATCGGCACCCTATATTTTATCTTCGGAGCCTGAGCAGGAATAGTTGGAACAGCCCTGAGCCTACTCATTCGCACAGAACTAAGCCAACCCGGACCCCTTATAGGAGACGACCAGATTTACAATGTCATTGTCACCGCACATGCCTTCATTATAATTTTCTTCATAGTAATACCCATCATAATCGGGGGATTCGGAAACTGACTACTACCATTAATAATTGGAGCACCTGACATAGCATTCCCCCGAATAAACAACATAAGCTTCTGATTACTCCCACCATCATTTACTTTACTCCTCTTCTCAGCCTTCGTTGAAACTGGAGCTGGAACTGGATGAACAGTCTACCCACCACTAGCAGGAAACCTAGCCCACGCTGGACCCTCAGTAGATCTAACCATTTTCTCCCTTCATCTCGCAGGGGTATCTTCAATTCTAGGGGCAATTAATTTTATTACCACAGCCATAAACATAAAACCCCCAGCAATATCACAATACCAAACACCTCTTTTCGTATGGTCTGTGCTTATTACAGCCGTACTACTCCTACTCTCTCTACCAGTCTTAGCTGCTGGAATCACCATACTCCTAACCGACCGAAACTTAAACACAACCTTTTTCGATCCATCTGGTGGCGGAGACCCAATCCTGTATCAACACCTCTTCTGATTCTTCGGACACCCAGAAGTCTACATCCTCATCCTCCCAGGATTCGGAATAATTTCCCACGTGGTTACCTTTTACTCAGGGAAAAAAGAACCATTTGGGTATATAGGGATAGTATGAGCAATAATATCAATTGGCTTCTTAGGCTTCATTGTCTGAGCTCACCATATATTTACAGTAGGAATAGACGTTGATACCCGAGCATATTTTACTTCAGCCACAATAGTTATTGCTATCCCCACAGGAGTAAAAGTATTCAGCTGATTAGCAACAATTTATGGGGGAATTATAAACTGACAAGCTCCAATGCTCTGAGCACTAGGCTTCATCTTCCTATTTACAGTAGGAGGACTGACCGGAATCGTCCTAGCCAACTCATCATTAGACATTATTCTCCACGACACCTACTATGTAGTAGCCCACTTCCATTATGTGTTATCAATAGGGGCAGTATTCGCAATTATAAGTGGGTTCACTCACT-GATTCCCACTA-TTTACAGGGTTTACTCTACACCCTATATGAACGAAAA-CCCAATTCATAATCATGTTTACAGGAGTAAACCTGACCTTCTTCCCACAACACTTCCTAGGCCTCTCCGGAATACCACGACGATACTCAGACTACCCAGACGCATATGCCTTCTGAAACATGATCTCGTCAATCGGGTCATTAATTTCCATAGTCTCTGTAATCCTCCTAATATTTATTGTGTGAGAAGCATTCTCATCAAAACGTAAAGTTCAAACACCAGAAATAGCAACCACAAATATTGAATGACTCAATAGCTGCCCTCCATCCCACCACACCTATGAAGAGCCAGTATTTGTTCAAACACAAT--------------------------------------------------ATGGCAAACCCGATACATCTAGGGCTCCAGGATGCAATATCCCCACTAATAGAAGAACTCCTATATTTCCATGACCACACATTAATAGTCATTTTCCTAATCAGCATGCTTGTACTCTACACCATCTCCGTCCTCCTACTATCAAAATTATATCACACTAACGCCACAGATGTACAAGAAGTAGAAATAATTTGAACAATTCTACCAGCCATCATCCTAATTACCATTGCCCTCCCATCACTTCGCACTCTCTACTTAATAGACGAAACTAGCAACCCATGCCTAACCATTAAAGTCATTGGCCACCAATGATATTGAACCTACGAGTACACAGACTTTTCACAGCTAGAATTTGACTCTTACATAGTACCGTCACAGGACCTCCTACCAGGACACTTCCGCCTGTTAGAAGTAGATCACCGTATAATTACACCAACAGGTGCAACTACCCGGACACTAATTACAGCAGAAGACGTACTACACTCCTGAGCAGTACCGTCCTTAGGAATTAAAATAGACGCAGTACCAGGACGCCTGAACCAAACCTCAATTACACTAGCTAACCCAGGAATTTTTTATGG-TCAATGCTCCGAAATCTGTGGAGCCAACCACAGCTTCATGCCTATCGTAGTAGAAGCAATCCCAATACAATACTTCCAAAAATGGTTAGAAACAAACGTTTCTTCATATGACACACCAAACACATCCGTTTCACATAGTTAACCCTAGCCCTTGACCCATTATAAGTGCCATAGCTGCCATAATACTAACAACAGGACTGGTACTATGATTTCACCACAATCTCAACCTCCTTCTGCTATTAGGACTAATCTCAACACTAATGATCATGTATCAATGATGACGAGATATTGTTCGAGAAAGTACCTACTTAGGCCACCACACCCCACCAGTTCAAAAAGGGTTACGCTACGGCATAATTCTATTTATTACATCAGAAGTTTTCTTCTTCCTAGGATTCTTCTGAGCATTTTACCACTCTAGCCTAGCCCCAACCCCAGAACTAGGAGGACAATGACCACCAACTGGGATCACCCCACTAGACCCATTTGAAGTCCCTCTCCTAAACACCGCAGTACTTCTGGCCTCAGGGGTCACAGTGACATGAGCACACCACAGCCTAATAGAAGCCAACCGAACGCCCACCATCCACGCCCTTACACTTACCATTATTCTAGGACTGTACTTTACTGCCCTTCAAGCAATAGAATACTATGAAGCCCCATTCACTATCGCAGATAGTACCTACGGATCTACCTTTTTCGTAGCTACAGGCTTCCACGGCCTCCATGTAATCATCGGATCGACATTCCTAATAGTCTGCCTATACCGACAAATTATACATCACTTCACCTCGAACCACCACTTTGGCTTTGAAGCTGCCGCCTGATACTGACACTTTGTAGACGTGGTATGACTATTCCTCTACATCTCAATCTATTGATGAGGCTCCTATCGGCTTCCTTATAGCTGCCCCTTTTGTGCTTTATATTATCCTAATCTTGATCGCAGTAGCATTCCTAACAGCCCTGGAACGAAAAATTATTGGTTACATACAACTGCGAAAAGGCCCCAATACTGTAGGACCCTTAGGCCTCCTACAACCGTTTGCTGATGGTCTCAAACTCATCATCAAAGAATTAACCTTCCCCATTCTTGCTACCCCCACCCTATTCATCCTGTCCCCTGCAGTAGCTTTAATCTTATCCCTTACCGTATGAGCTCCACTCCCAATACCATTCTCCCTAGCCAACCTAAACCTGGGTATACTATTTATATTAGCCATATCCAGCCTAGCAGTCTACTCCCTACTCTGATCTGGCTGAGCATCAAACTCAAAATACGCCCTAATAGGGGCCTTACGAGCCGTTGCCCAAACCATCTCCTATGAGGTAACACTAGCTATCATTATTCTATCAATCGTCCTTCTCAGTGGTGGGTTCTCACTACACACACTAGCTATTACTCAAGAGCCAACATACTTGGCATTAACCACATGACCCTTACTAATAATATGATATACATCAACGCTCGCAGAAACAAACCGAGCCCCCTTCGACTTAACAGAAGGAGAATCAGAGCTAGTTTCAGGATTTAATGTCGAATACAGTGCAGGATTATTTACACTTTTTTTCCTAGCCGAGTACGCCAATATTTTACTAATAAATATTTTAACCACCATCCTATTCCTCAATACATCAACTAACTTCTCCATACAAATACTTTTTACCATCACCCTAATAACCAAATCCATCCTACTAACTATAGGGTTCCTATGAATTCGAGCATCATACCCTCGGTTCCGCTACGACCAGCTAATACATCTTCTATGAAAGAGCTTCCTGCCAGCCACACTAACAATTTGTCTATGACACACATCATTTCCAGTGTCAATGTTTGGCCTGCCAGCGATAA---GGATGCCGCTTTTCCAACCCATCATCCTAACAACACTGACTATCACCACATTCATCTTTTTATCATCCACGCATCTTGTACTAATATGAGTAGCACTAGAGCTAAGCACACTAGCGATTCTCCCCCTAATTGCCACTAAATCACATCCCCGGGCTATCGAGGCATCCACAAAATATTTCCTCACACAAGCAACTGCTTCCGCACTGATTATTTTCTCAGGAACATTAAATTACACCATAACAGGAAACTGCCAAATCACTGAACTAACCAACCCAAACTTAATAATAGTCCTGATATTAGCCATATTCATCAAAATTGGCCTAGTCCCCTTCCATTTTTGAGTTCCCGAAACACTCCAAGGTATAGCTCCAACTGCCGCCATCTTCCTACTAACTTGACAAAAACTAGGCCCATTAATTATATTATATCTAATAAGCCCACTAATCAACTTTGAAATCCTTTCCATAATAGCCATTCTATCCGCCATAACTGCCGGTTGACTTGGATTAAACCAAACCCAAGTTCGAAAGCTAGTGGCTCTATCCTCCATCGCCCAAATAGCTTGGACCCTCGTAATCATTAAATACGCACCATCACTTACAATTCTAGCCTTCTACTTGTATTCACTTACCATCTCCGCCACACTCCTCACCCTAAACAAACTATCAACAACGTCTATTAGCGGCCTTCTACTCTCCTTTTCAAAAACCCCTATTATTTCATTCCTACTAACAATCTCCCTACTGTCCCTGTCAGGCCTCCCCCCTTTAGCCGGCTTCTTACCAAAATGATTAACAATTGATATACTCATAGCAGAAGAAGCTATCTGAATCGCCTTTATAATACTTATAGCATCACTCCTAAGCCTATTCTTCTACCTACGACTATGGTACAACTCCGCATCCACCACCCCCCCCAACACCACAAACACTTCTCGCCTATGACGAAAACCCCTCCCCAAAACAAACCTCACAATTAACCTATTAAGCATAGCTGCCTTTACCCTTATCCTAGCAGCCACTTTAATGAAAGCTATCACAAAACAATA----------------A----TAAACTTGCTCACCATGTTTATACTAGCTATAGCCACTGCAGTGGCTGTAGCCACCCTAAACCTATTAATATCTGAAACAGCCCCAGACCCCGAAAAACTATCACCATATGAGTGCGGATTTGACCCACTAGGCTCAGCCCGACTACCATTCTCCATTCGGTTTTTTATAATTGCCATCTTATTCCTTTTATTTGATCTAGAAATCGCTATTCTACTACCCCTTGCATGAGCCCTACAACTTACAAACCTCATTAAAACAACAACATGAGCTATCATTATCTTTTTATTTATATTTATTGGTTTAGCATACGAGTGATCACAGGGGGGCCTAGAATGAGCAGAATAAATGCTAAAACTCGTAGTACCAACAATAATGTTAATCCCCTCCACCTGCTTCACACATCACAAAACAATCTGACTATCACCAACAGCCTATTCTACAGCCATTATCATTTTAACCTTAATTGTCCTAAACCCAGGAGATATGCTCATAAATACTAATGGCCTCCTCTTGGGCAGCGATCAAATCTCTACACCTCTACTAATCCTATCATGCTGACTTCTCCCACTCATATTCATGGCCAGCCAAAGCTCCATATCCCAAGACCCAAACCCCCAAAAACGACTGTTCATTACCGCCCTAGCCCTTCTTCAACTCGCCCTACTACTTGTATTCATGGCCCTAGACCTAATATTATTTTATACCACGTTCGAAGCAACCCTTATTCCAACACTAATGGTAATTGCTCGTTGAGGCACCCAAACAGAACGCCTGGGGGCCGGAATATATTTCCTACTCTACACCATCACCAGCTCAATACCACTCTTAATAGCCCTCCTATGAATTTATAACATAAAAGGAACTCTATCCATCATACTTCTCCAACTAATATCACCAATAACACTCACATTCTGAACAGACATAATATTATGGCTATCCGTTCTACTCGCCTTCCTAGTAAAAGTCCCAATCTACGGCCTCCACCTATGACTCCCCAAAGCACACGTAGAAGCCCCAATTGCCGGATCCATAGTCCTAGCCGCAGTACTCCTAAAACTTGGAGGCTATGGATTATTACGAGTAACAAATCTATTAACAGAACAAAACAATTCCTCCTACACCCTACCACTAACAGTAGCACTATGAGGATCACTCATAACTGGCATAATCTGTTTACGACAAACAGACTTAAAATCCCTAATCGCCTACTCATCAGTAAGCCACATAGGACTTATAACAAGCTCAATCTTAACCCGAAACCAACTAGCCCTATCTGGCTCAATAACTATAATAATCGCCCACGGCCTAACATCCTCCATACTATTTTGTTTAGCTAACATCACCTATGAACGAACACACTCACGAACACTCCTACTAGCCCAAGGAGTACAACTTACCACCCCAACCATGACCTCCTGATGGCTACTAGCCTGCCTAACAAACATAGCACTCCCTCCAACAATTAATTTCATTGGAGAACTCACCCTTATAATCTCCTTATTTAGTTGATCAGACGTTACCATCCTACTAACAGGGCTAAGCGCATCCATCACTTCAATCTACACCCTCCACATATTCTCGTCCACCCAGCAAGGAACACTCCCCTCACACATCATTACAATAACCCCAACACAAACACGCGAACACCTTCTAATACTTATACACTCCGCACCATCACTTGCTCTCATCATGGCCCCCCAATTAATATGCTCTCAATAGTTGAGCACCCCCACCAACCTCATGTTCACCTGTTCCTTTATCATTTTTGCTATCGGACTCACCTTTCACCACACCCATCTACTCTCGGCCCTGCTCTGCCTAGAAGGCATAATACTTTCAGTTTTTATGCTATTGACAATATGATCCCTTAACTCTAACACCTCCTCCTTTATCCTCCCACTAACAGTACTAACCCTTTCAGCCTGCGAAGCAGGCATTGGTCTTGCCTTGCTAATTGCCTCAGCCCGCACACACAACACAACCAACCTTAAAAACCTTAACCTCCTCCAATGCTAAATGAATCCAGCTATCA-------------TGCTTTTCATACTACCACTAACCATTATATTATCGTCACTATTCCTTCCTTACT-CAAAAATGAACAAA--CCATTCCACGCCAAAGTACTAGCAACAAAACTAGCATTCTTCTCCAGCCTACTTCCATTAGCCTTTTTTCTCTACGACGGATTAGTTGTTACCTCACACGAAGTTTATTGACTAGCCTTCAACGTATGCCCCATTCACATTAGCTTCACACTAGACAAATATTCAGTCCTCTTCCTACCAATCTCACTATTCGTCGCATGATCCATCATAGAATTTACTGTTAAATATATAGAATCAGACCCCAAAATCGACACCTTCTTCCGCTACCTTATTATCTTCACTTTAATGATAATAGTACTAGTAACCGCCGAAAATGTAGTTCAACTCTTCATTGGTTGAGAAGGAGTAGGCATTATATCCTATATACTCATTAACTGATGATCCTACCGATCAACCTCCAACAAAGCAGCCCTCCAGGCGGTGATTTACAACCGATTGGCAGACGTCGGCCTAGCAATCGCCCTAGCATGGATAGTCGTAAACAACCTCTCCCTGGACATTCAAGGCGTACGAGCCTCTCCAGACCTCGCTCTTATCCCCGCATTAGGCTTTATCCTAGCCGCCGCTGGAAAGTCCGCCCAATTTGGCTTCCACCCATGACTACCAGCAGCAATAGAAGGCCCAACCCCTGTATCCGCCCTACTACACTCAAGCACCATAGTAGTAGCAGGCGTATTTCTACTAATCCGAACCTCAAAATTAATTTATAGTAGTGAAACAGCAACTATAATCTGCCTGTTACTAGGAGCACTCACATCCCTGCTAGCGGCAACCTGCGCACTAACCCAAAATGATATAAAAAAAATCATTGCCTACTCTACTACCAGCCAACTAGGGCTGATAATAACAGCAATCGGACTAAAACAACCCGAACTCGCATTTATACATATCGCAACACATGCCTTCTTTAAAGCAATACTATTCCTTTGCGCCGGAGCAATTATCCACAATCTTAACAACGAACAAGACATCCGAAAAATAGGAGGCCTAAAAAAAGCAATGCCGATCACCTCATCCTGCCTAATTATTGGTGCCCTAGCCCTATCAGGAATGCCATTCATATCCGGCTTCTACTCAAAAGACGCCATCATCGAAGCACTTAACACCTCCGACATCAACTTCTTCTCACTAGCTATAACCCTAATCGCCACTACCTTCACTGTACTCTACAACCTACGCATAATTTACTTCGTAGTCCTAGGTAGCCCACGAATACCAACACTCTCTGCACTTCCAGAAGCCCGTCAAACCCTCAACCCAATCCTACGGCTGGCCGTAGGAAGTATTGCAGCAGGACTTCTAATTTCAATCAACATGCTTCCCTCCAACATTCCACAAATAACCATACCACCAGAAATTAAACTCGCTGCCCTGATCGTTACAGCCTTAGGATTCCTGACAGGCGCAGCCTTAATATCCACAGCCCCAACCCTCCAGCCATCCACCAAAGGCACCCAATCCCCCCTGCCCTTCAAAATAACCCACTTCTACTTTATTATCCACCACACACTATCGACCATTACTTTATGAATTAGCCAAAAATTATCAAATCATTTAACAGACCAAACACACTATGAAGCAACAGGACCCAAAATGCTAGCCTACCTCCAAATCCTAATAACTAAAGCCCTGACAAGCCTTCATAAATCCCGAATCAACTCATACCTAAAAATCATTGCCTTAGCTATAATACTTATCCTGCTACTAT-----CCCT---------------------------ACCCT---------------ATGGAAATTGCATTTCTTTTTCTTTGTTGTTTAATATTGGTTGGTGTTGTGTTAGTGGCGGCGGGTGCTACAACACACTATGGGGTGGTAAGTTTGCTCTTTGCTGCGGTATTAAGTAGTGGTTTATTGGTGGTTGGAGGAGGCAGTTTTATGCCCATTGTAGTATTGTTAATCTATCTAGGTGGGTTATTGGTGGTATTTGCTTTTTGTGTTGGGTTCACTGATGATGAGTATTGTGAATTTTGGGGGACGGGAGCATCTAAAGGGGTGGCCGGTGTTTGTGGGGTCGGGTTGATGGTGATGGGTTATCGTATGTATAAGTATACATGG----GTAGGGACCTTGGGGGGCTTTT--TAGATGCTGTGGAGGTTTGGAGTGAGGATATCAGTAATGAACTTCTAGGGGTTGGGTTATTTTATTTGGAAGGTTGGGTTTTTGTTGCATTAAGTGGTTGGGCTTTGTTGATTGTGTTATTTGTAATTATAGGTCTCGTCCGGGGTCATCGTCGAGGGGCACTTCGGTCATTGAGG

*Gavialis gangeticus 2*

ATGACCTTGGCCACAAATCTAAGCTTATTTGATCAATTTTCAATCCCTAAACTCCTAGGAATCCCCATACTAATCCCCGCCATATTAATAACAAGCATGTTATTATATAACCCAGAAGACCGATGATTGTCTAACCCAACAACAACCATCCAATCCTGATTAATTGCAAAAACAACTAAACAAATTATAGCCCCAGTGAATCAACCAGGACACAAATGATCCCTAATATTGATTTCGCTACTAACCTTTCTTCTTATCAATAACCTCCTAGGACTACTCCCATATACTTTTACACCAACAACGCAACTATCTATAAACCTAGCCTTAGCCCTACCCTTATGAATGGCAACAGTATTAATTGGGTTACGAAATAAACCAACCGCCTCCTTAGCTCACCTACTTCCAGAAGGAACTCCAACACCCCTAATTCCAATCTTAATTTTAATTGAGTCCATTAGTCTATTAATCCGACCAATCGCTCTTGCCGTACGACTAACAGCCAACCTAACCGCCGGACACCTTTTAATTCACCTAATTGCCGCCACAGCGCTGAACCTACTAATAACATCCATACTACTAGCTGCACTGACCCTAACTATTTTAACACTACTAACGCTACTAGAAATTGCAGTAGCAATTATTCAAGCCTATGTTTTTACCCTACTCCTCTCCCTCTACCTACAAGAAAATGTATAA-TGCCCCAACTAAACCCAAAACCCTGACTTATGGTTTTCCTTACCACTTGATTTATATTAATTATTGCTACACACCCAAAAATTGCCTCCCTAAAATTTATAAATAACCCAGCACCCTTTCACCCCACACCAATT-AAACCATGACCTTGGCCACAAATCTAA---------------------ATGGCCCACCAACTACGAAAATCCCACCCCCTACTAAAACTTGTAAACCACTCATTAATTGACCTCCCCACCCCATCCAACATCTCCTATTGATGAAACTTCGGATCACTACTAGGATTCACCCTTTTAATCCAAATCCTATCAGGAGTTTTACTAATAATACACTTCCTAGCAGATGACACTATGGCCTTCGCATCTGTTGCTTACACCTCACGAGAAGTTTGATATGGTTGACTTATCCGAAGCCTCCACGCAAACGGAGCCTCCCTCTTCTTCCTATGCATCTTCTTACACATTGGACGTGGCTTATATTACGGCTCTTACCTACATGAAAATACATGAAACATTGGAGTCATCCTGCTCCTCTTATTAATAGCGACAGCATTCATAGGCTATGTCCTACCCTGAGGACAAATATCATTCTGAGGGGCAACCGTAATTACAAACCTCATGTCAGCCATCCCATATATTGGAGACTCCCTCGTAATCTGAATCTGAGGAGGCCCTTCAGTTAACAGCGCCACCCTAACACGCTTTACTACCTTACACTTTCTACTCCCATTCATTCTACTAGCAGCCATCATCACCCACCTCATATTTCTACATGAACAAGGCTCATTTAACCCCTTAGGACTGCCTCCCAACACCGACAAAGTCCCATTTCACCCATACTTCTCCCTAAAAGACGCTATAGGGATAATACTCGCCGTCACCTTACTTTCAACTTTAGCCCTATATCTCCCAAACCTCTTAGGAGACCCAGAAAACTTCACACCAGCTGACCCCATAAAAACCCCTAACCACATCAAACCCGAATGATACTTCCTATTCGCCTACACAATCCTACGATCCATTCCCAATAAGCTAATAGGAGTATTAGCTATATTTTCATCAATCCTAATTCTCTTCCTAATGCCCATGCTTCACACATCAAAACGACAATCAATAGCCATGCGCCCCCTATCCCAACTTCTATTTTGAACACTCGTCGCCGACTTCTTTGTATTAACCTGGATCGGAGGACAGCCCGTACAAGACCCCTACACACTAATCGGCCAAATAGCCTCCATAACCTACTTCACAATCATCCTTATTCTCATACCACTAACAGGCCAAGTAGAAAACTGAATTATTAAACCTCTACGATACCGAT-------------GTGAACATAAATCGCTGACTTTTCTCCACCAATCACAAAGACATCGGCACCCTATATTTTATCTTCGGAGCCTGAGCAGGAATAGTTGGAACAGCCCTGAGCCTACTCATTCGCACAGAACTAAGCCAACCCGGACCCCTTATAGGAGACGACCAGATTTACAATGTCATTGTCACCGCACATGCCTTCATTATAATTTTCTTCATAGTAATACCCATCATAATCGGGGGATTCGGAAACTGACTACTACCATTAATAATTGGAGCACCTGACATAGCATTCCCCCGAATAAACAACATAAGCTTCTGATTACTCCCACCATCATTTACTTTACTCCTCTTCTCAGCCTTCGTTGAAACTGGAGCTGGAACTGGATGAACAGTCTACCCACCACTAGCAGGAAACCTAGCCCACGCTGGACCCTCAGTAGATCTAACCATTTTCTCCCTTCATCTCGCAGGGGTATCTTCAATTCTAGGGGCAATTAATTTTATTACCACAGCCATAAACATAAAACCCCCAGCAATATCACAATACCAAACACCTCTTTTCGTATGGTCTGTGCTTATTACAGCCGTACTACTCCTACTCTCTCTACCAGTCTTAGCTGCTGGAATCACCATACTCCTAACCGACCGAAACTTAAACACAACCTTTTTCGATCCATCTGGTGGCGGAGACCCAATCCTGTATCAACACCTCTTCTGATTCTTCGGACACCCAGAAGTCTACATCCTCATCCTCCCAGGATTCGGAATAATTTCCCACGTGGTTACCTTTTACTCAGGGAAAAAAGAACCATTTGGGTATATAGGGATAGTATGAGCAATAATATCAATTGGCTTCTTAGGCTTCATTGTCTGAGCTCACCATATATTTACAGTAGGAATAGACGTTGATACCCGAGCATATTTTACTTCAGCCACAATAGTTATTGCTATCCCCACAGGAGTAAAAGTATTCAGCTGATTAGCAACAATTTATGGGGGAATTATAAACTGACAAGCTCCAATGCTCTGAGCACTAGGCTTCATCTTCCTATTTACAGTAGGAGGACTGACCGGAATCGTCCTAGCCAACTCATCATTAGACATTATTCTCCACGACACCTACTATGTAGTAGCCCACTTCCATTATGTGTTATCAATAGGGGCAGTATTCGCAATTATAAGTGGGTTCACTCACT-GATTCCCACTA-TTTACAGGGTTTACTCTACACCCTATATGAACGAAAA-CCCAATTCATAATCATGTTTACAGGAGTAAACCTGACCTTCTTCCCACAACACTTCCTAGGCCTCTCCGGAATACCACGACGATACTCAGACTACCCAGACGCATATGCCTTCTGAAACATGATCTCGTCAATCGGGTCATTAATTTCCATAGTCTCTGTAATCCTCCTAATATTTATTGTGTGAGAAGCATTCTCATCAAAACGTAAAGTTCAAACACCAGAAATAGCAACCACAAATATTGAATGACTCAATAGCTGCCCTCCATCCCACCACACCTATGAAGAGCCAGTATTTGTTCAAACACAAT--------------------------------------------------ATGGCAAACCCGATACATCTAGGGCTCCAGGATGCAATATCCCCACTAATAGAAGAACTCCTATATTTCCATGACCACACATTAATAGTCATTTTCCTAATCAGCATGCTTGTACTCTACACCATCTCCGTCCTCCTACTATCAAAATTATATCACACTAACGCCACAGATGTACAAGAAGTAGAAATAATTTGAACAATTCTACCAGCCATCATCCTAATTACCATTGCCCTCCCATCACTTCGCACTCTCTACTTAATAGACGAAACTAGCAACCCATGCCTAACCATTAAAGTCATTGGCCACCAATGATATTGAACCTACGAGTACACAGACTTTTCACAGCTAGAATTTGACTCTTACATAGTACCGTCACAGGACCTCCTACCAGGACACTTCCGCCTGTTAGAAGTAGATCACCGTATAATTACACCAACAGGTGCAACTACCCGGACACTAATTACAGCAGAAGACGTACTACACTCCTGAGCAGTACCGTCCTTAGGAATTAAAATAGACGCAGTACCAGGACGCCTGAACCAAACCTCAATTACACTAGCTAACCCAGGAATTTTTTATGG-TCAATGCTCCGAAATCTGTGGAGCCAACCACAGCTTCATGCCTATCGTAGTAGAAGCAATCCCAATACAATACTTCCAAAAATGGTTAGAAACAAACGTTTCTTCATATGACACACCAAACACATCCGTTTCACATAGTTAACCCTAGCCCTTGACCCATTATAAGTGCCATAGCTGCCATAATACTAACAACAGGACTGGTACTATGATTTCACCACAATCTCAACCTCCTTCTGCTATTAGGACTAATCTCAACACTAATGATCATGTATCAATGATGACGAGATATTGTTCGAGAAAGTACCTACTTAGGCCACCACACCCCACCAGTTCAAAAAGGGTTACGCTACGGCATAATTCTATTTATTACATCAGAAGTTTTCTTCTTCCTAGGATTCTTCTGAGCATTTTACCACTCTAGCCTAGCCCCAACCCCAGAACTAGGAGGACAATGACCACCAACTGGGATCACCCCACTAGACCCATTTGAAGTCCCTCTCCTAAACACCGCAGTACTTCTGGCCTCAGGGGTCACAGTGACATGAGCACACCACAGCCTAATAGAAGCCAACCGAACGCCCACCATCCACGCCCTTACACTTACCATTATTCTAGGACTGTACTTTACTGCCCTTCAAGCAATAGAATACTATGAAGCCCCATTCACTATCGCAGATAGTACCTACGGATCTACCTTTTTCGTAGCTACAGGCTTCCACGGCCTCCATGTAATCATCGGATCGACATTCCTAATAGTCTGCCTATACCGACAAATTATACATCACTTCACCTCGAACCACCACTTTGGCTTTGAAGCTGCCGCCTGATACTGACACTTTGTAGACGTGGTATGACTATTCCTCTACATCTCAATCTATTGATGAGGCTCCTATCGGCTTCCTTATAGCTGCCCCTTTTGTGCTTTATATTATCCTAATCTTGATCGCAGTAGCATTCCTAACAGCCCTGGAACGAAAAATTATTGGTTACATACAACTGCGAAAAGGCCCCAATACTGTAGGACCCTTAGGCCTCCTACAACCGTTTGCTGATGGTCTCAAACTCATCATCAAAGAATTAACCTTCCCCATTCTTGCTACCCCCACCCTATTCATCCTGTCCCCTGCAGTAGCTTTAATCTTATCCCTTACCGTATGAGCTCCACTCCCAATACCATTCTCCCTAGCCAACCTAAACCTGGGTATACTATTTATATTAGCCATATCCAGCCTAGCAGTCTACTCCCTACTCTGATCTGGCTGAGCATCAAACTCAAAATACGCCCTAATAGGGGCCTTACGAGCCGTTGCCCAAACCATCTCCTATGAGGTAACACTAGCTATCATTATTCTATCAATCGTCCTTCTCAGTGGTGGGTTCTCACTACACACACTAGCTATTACTCAAGAGCCAACATACTTGGCATTAACCACATGACCCTTACTAATAATATGATATACATCAACGCTCGCAGAAACAAACCGAGCCCCCTTCGACTTAACAGAAGGAGAATCAGAGCTAGTTTCAGGATTTAATGTCGAATACAGTGCAGGATTATTTACACTTTTTTTCCTAGCCGAGTACGCCAATATTTTACTAATAAATATTTTAACCACCATCCTATTCCTCAATACATCAACTAACTTCTCCATACAAATACTTTTTACCATCACCCTAATAACCAAATCCATCCTACTAACTATAGGGTTCCTATGAATTCGAGCATCATACCCTCGGTTCCGCTACGACCAGCTAATACATCTTCTATGAAAGAGCTTCCTGCCAGCCACACTAACAATTTGTCTATGACACACATCATTTCCAGTGTCAATGTTTGGCCTGCCAGCGATAA---GGATGCCGCTTTTCCAACCCATCATCCTAACAACACTGACTATCACCACATTCATCTTTTTATCATCCACGCATCTTGTACTAATATGAGTAGCACTAGAGCTAAGCACACTAGCGATTCTCCCCCTAATTGCCACTAAATCACATCCCCGGGCTATCGAGGCATCCACAAAATATTTCCTCACACAAGCAACTGCTTCCGCACTGATTATTTTCTCAGGAACATTAAATTACACCATAACAGGAAACTGCCAAATCACTGAACTAACCAACCCAAACTTAATAATAGTCCTGATATTAGCCATATTCATCAAAATTGGCCTAGTCCCCTTCCATTTTTGAGTTCCCGAAACACTCCAAGGTATAGCTCCAACTGCCGCCATCTTCCTACTAACTTGACAAAAACTAGGCCCATTAATTATATTATATCTAATAAGCCCACTAATCAACTTTGAAATCCTTTCCATAATAGCCATTCTATCCGCCATAACTGCCGGTTGACTTGGATTAAACCAAACCCAAGTTCGAAAGCTAGTGGCTCTATCCTCCATCGCCCAAATAGCTTGGACCCTCGTAATCATTAAATACGCACCATCACTTACAATTCTAGCCTTCTACTTGTATTCACTTACCATCTCCGCCACACTCCTCACCCTAAACAAACTATCAACAACGTCTATTAGCGGCCTTCTACTCTCCTTTTCAAAAACCCCTATTATTTCATTCCTACTAACAATCTCCCTACTGTCCCTGTCAGGCCTCCCCCCTTTAGCCGGCTTCTTACCAAAATGATTAACAATTGATATACTCATAGCAGAAGAAGCTATCTGAATCGCCTTTATAATACTTATAGCATCACTCCTAAGCCTATTCTTCTACCTACGACTATGGTACAACTCCGCATCCACCACCCCCCCCAACACCACAAACACTTCTCGCCTATGACGAAAACCCCTCCCCAAAACAAACCTCACAATTAACCTATTAAGCATAGCTGCCTTTACCCTTATCCTAGCAGCCACTTTAATGAAAGCTATCACAAAACAATA----------------A----TAAACTTGCTCACCATGTTTATACTAGCTATAGCCACTGCAGTGGCTGTAGCCACCCTAAACCTATTAATATCTGAAACAGCCCCAGACCCCGAAAAACTATCACCATATGAGTGCGGATTTGACCCACTAGGCTCAGCCCGACTACCATTCTCCATTCGGTTTTTTATAATTGCCATCTTATTCCTTTTATTTGATCTAGAAATCGCTATTCTACTACCCCTTGCATGAGCCCTACAACTTACAAACCTCATTAAAACAACAACATGAGCTATCATTATCTTTTTATTTATATTTATTGGTTTAGCATACGAGTGATCACAGGGGGGCCTAGAATGAGCAGAATAAATGCTAAAACTCGTAGTACCAACAATAATGTTAATCCCCTCCACCTGCTTCACACATCACAAAACAATCTGACTATCACCAACAGCCTATTCTACAGCCATTATCATTTTAACCTTAATTGTCCTAAACCCAGGAGATATGCTCATAAATACTAATGGCCTCCTCTTGGGCAGCGATCAAATCTCTACACCTCTACTAATCCTATCATGCTGACTTCTCCCACTCATATTCATGGCCAGCCAAAGCTCCATATCCCAAGACCCAAACCCCCAAAAACGACTGTTCATTACCGCCCTAGCCCTTCTTCAACTCGCCCTACTACTTGTATTCATGGCCCTAGACCTAATATTATTTTATACCACGTTCGAAGCAACCCTTATTCCAACACTAATGGTAATTGCTCGTTGAGGCACCCAAACAGAACGCCTGGGGGCCGGAATATATTTCCTACTCTACACCATCACCAGCTCAATACCACTCTTAATAGCCCTCCTATGAATTTATAACATAAAAGGAACTCTATCCATCATACTTCTCCAACTAATATCACCAATAACACTCACATTCTGAACAGACATAATATTATGGCTATCCGTTCTACTCGCCTTCCTAGTAAAAGTCCCAATCTACGGCCTCCACCTATGACTCCCCAAAGCACACGTAGAAGCCCCAATTGCCGGATCCATAGTCCTAGCCGCAGTACTCCTAAAACTTGGAGGCTATGGATTATTACGAGTAACAAATCTATTAACAGAACAAAACAATTCCTCCTACACCCTACCACTAACAGTAGCACTATGAGGATCACTCATAACTGGCATAATCTGTTTACGACAAACAGACTTAAAATCCCTAATCGCCTACTCATCAGTAAGCCACATAGGACTTATAACAAGCTCAATCTTAACCCGAAACCAACTAGCCCTATCTGGCTCAATAACTATAATAATCGCCCACGGCCTAACATCCTCCATACTATTTTGTTTAGCTAACATCACCTATGAACGAACACACTCACGAACACTCCTACTAGCCCAAGGAGTACAACTTACCACCCCAACCATGACCTCCTGATGGCTACTAGCCTGCCTAACAAACATAGCACTCCCTCCAACAATTAATTTCATTGGAGAACTCACCCTTATAATCTCCTTATTTAGTTGATCAGACGTTACCATCCTACTAACAGGGCTAAGCGCATCCATCACTTCAATCTACACCCTCCACATATTCTCGTCCACCCAGCAAGGAACACTCCCCTCACACATCATTACAATAACCCCAACACAAACACGCGAACACCTTCTAATACTTATACACTCCGCACCATCACTTGCTCTCATCATGGCCCCCCAATTAATATGCTCTCAATAGTTGAGCACCCCCACCAACCTCATGTTCACCTGTTCCTTTATCATTTTTGCTATCGGACTCACCTTTCACCACACCCATCTACTCTCGGCCCTGCTCTGCCTAGAAGGCATAATACTTTCAGTTTTTATGCTATTGACAATATGATCCCTTAACTCTAACACCTCCTCCTTTATCCTCCCACTAACAGTACTAACCCTTTCAGCCTGCGAAGCAGGCATTGGTCTTGCCTTGCTAATTGCCTCAGCCCGCACACACAACACAACCAACCTTAAAAACCTTAACCTCCTCCAATGCTAAATGAATCCAGCTATCA-------------TGCTTTTCATACTACCACTAACCATTATATTATCGTCACTATTCCTTCCTTACT-CAAAAATGAACAAA--CCATTCCACGCCAAAGTACTAGCAACAAAACTAGCATTCTTCTCCAGCCTACTTCCATTAGCCTTTTTTCTCTACGACGGATTAGTTGTTACCTCACACGAAGTTTATTGACTAGCCTTCAACGTATGCCCCATTCACATTAGCTTCACACTAGACAAATATTCAGTCCTCTTCCTACCAATCTCACTATTCGTCGCATGATCCATCATAGAATTTACTGTTAAATATATAGAATCAGACCCCAAAATCGACACCTTCTTCCGCTACCTTATTATCTTCACTTTAATGATAATAGTACTAGTAACCGCCGAAAATGTAGTTCAACTCTTCATTGGTTGAGAAGGAGTAGGCATTATATCCTATATACTCATTAACTGATGATCCTACCGATCAACCTCCAACAAAGCAGCCCTCCAGGCGGTGATTTACAACCGATTGGCAGACGTCGGCCTAGCAATCGCCCTAGCATGGATAGTCGTAAACAACCTCTCCCTGGACATTCAAGGCGTACGAGCCTCTCCAGACCTCGCTCTTATCCCCGCATTAGGCTTTATCCTAGCCGCCGCTGGAAAGTCCGCCCAATTTGGCTTCCACCCATGACTACCAGCAGCAATAGAAGGCCCAACCCCTGTATCCGCCCTACTACACTCAAGCACCATAGTAGTAGCAGGCGTATTTCTACTAATCCGAACCTCAAAATTAATTTATAGTAGTGAAACAGCAACTATAATCTGCCTGTTACTAGGAGCACTCACATCCCTGCTAGCGGCAACCTGCGCACTAACCCAAAATGATATAAAAAAAATCATTGCCTACTCTACTACCAGCCAACTAGGGCTGATAATAACAGCAATCGGACTAAAACAACCCGAACTCGCATTTATACATATCGCAACACATGCCTTCTTTAAAGCAATACTATTCCTTTGCGCCGGAGCAATTATCCACAATCTTAACAACGAACAAGACATCCGAAAAATAGGAGGCCTAAAAAAAGCAATGCCGATCACCTCATCCTGCCTAATTATTGGTGCCCTAGCCCTATCAGGAATGCCATTCATATCCGGCTTCTACTCAAAAGACGCCATCATCGAAGCACTTAACACCTCCGACATCAACTTCTTCTCACTAGCTATAACCCTAATCGCCACTACCTTCACTGTACTCTACAACCTACGCATAATTTACTTCGTAGTCCTAGGTAGCCCACGAATACCAACACTCTCTGCACTTCCAGAAGCCCGTCAAACCCTCAACCCAATCCTACGGCTGGCCGTAGGAAGTATTGCAGCAGGACTTCTAATTTCAATCAACATGCTTCCCTCCAACATTCCACAAATAACCATACCACCAGAAATTAAACTCGCTGCCCTGATCGTTACAGCCTTAGGATTCCTGACAGGCGCAGCCTTAATATCCACAGCCCCAACCCTCCAGCCATCCACCAAAGGCACCCAATCCCCCCTGCCCTTCAAAATAACCCACTTCTACTTTATTATCCACCACACACTATCGACCATTACTTTATGAATTAGCCAAAAATTATCAAATCATTTAACAGACCAAACACACTATGAAGCAACAGGACCCAAAATGCTAGCCTACCTCCAAATCCTAATAACTAAAGCCCTGACAAGCCTTCATAAATCCCGAATCAACTCATACCTAAAAATCATTGCCTTAGCTATAATACTTATCCTGCTACTAT-----CCCT---------------------------ACCCT---------------ATGGAAATTGCATTTCTTTTTCTTTGTTGTTTAATATTGGTTGGTGTTGTGTTAGTGGCGGCGGGTGCTACAACACACTATGGGGTGGTAAGTTTGCTCTTTGCTGCGGTATTAAGTAGTGGTTTATTGGTGGTTGGAGGAGGCAGTTTTATGCCCATTGTAGTATTGTTAATCTATCTAGGTGGGTTATTGGTGGTATTTGCTTTTTGTGTTGGGTTCACTGATGATGAGTATTGTGAATTTTGGGGGACGGGAGCATCTAAAGGGGTGGCCGGTGTTTGTGGGGTCGGGTTGATGGTGATGGGTTATCGTATGTATAAGTATACATGG----GTAGGGACCTTGGGGGGCTTTT--TAGATGCTGTGGAGGTTTGGAGTGAGGATATCAGTAATGAACTTCTAGGGGTTGGGTTATTTTATTTGGAAGGTTGGGTTTTTGTTGCATTAAGTGGTTGGGCTTTGTTGATTGTGTTATTTGTAATTATAGGTCTCGTCCGGGGTCATCGTCGAGGGGCACTTCGGTCATTGAGG

*Tomistoma schlegelii*

ATGACTATGGCCACAAATCTAAACTTATTTGATCAGTTCTTAATTCCTAAACTTCTTGGAGTACCCATACTAATTCCAGCCTTGTTAATAGCATCTATTTTATTGTACAACCCAGAAGACCGATGATTATCTAACCCAACAACAACCATCCAATACTGGTTAATCACAAAAGTGACCAAACAAATTATAACCCCAGTAAATCAGCCTGGACATAAATGATCATTAATATTAATTTCCCTCCTGACATTTCTTCTTATTAATAATCTTCTAGGCCTACTCCCATACACCTTTACACCAACGACACAGTTATCTATAAACCTAGCCTTAGCCCTACCTCTATGGATAGCAACAGTGTTAACTGGTCTACGAAATAAACCAAGCGCCTCCTTAGCCCATCTACTCCCAGAAGGAACTCCAACACCACTAATTCCAATCTTAATTTTGATCGAATCCATCAGCCTGCTAATCCGACCAATCGCTCTTGCCGTACGACTAACAGCCAATCTCACTGCCGGACACCTTTTAATTCACCTAATTGCCGCTACAGCATTAAACCTATTAACAACATCCCCACTACTAGCCGTACTGACCCTAATTATTCTAGTACTACTAATACTACTAGAAATTGCAGTAGCAATAATTCAAGCCTATGTTTTTACCCTGCTTCTATCCCTCTATCTACAAGAAAATGTATAAATGCCTCAACTAAACCCCAAACCTTGACTAATAGTTTTCCTTACCACCTGATTCATACTAATTATTGTTACACACCCCAAAATCGCCTCCTTAAAGCTTCTAAATAGCCCAGTACCTTTTCATCCAACCCCTATT-AAACCATGACTATGGCCACAAATCTAA---------------------ATGGCCCACCAACTACGAAAGTCCCACCCCCTACTAAAACTCATAAACCACTCTCTAATTGACCTCCCCACTCCATCCAACATCTCCTACTGATGAAACTTTGGATCACTTCTAGGGTTTACCCTTTTAATCCAAATCCTATCAGGCATTCTACTAATAATACATTTCCTAGCACATGACACCATAGCCTTCGCATCCGTCGCTTACACATCACGAGAAGTCTGATACGGCTGACTTATCCGAAGCCTTCACGCAAACGGAGCTTCCCTATTCTTCTTTTGCATCTTCTTACACATTGGACGTGGCCTCTACTACGGCTCTTATCTGCACGAAAACACATGGAACATCGGAGTCATTTTACTCCTCCTGCTAATAGCAACAGCTTTCATAGGTTACGTACTACCCTGAGGACAAATATCATTCTGAGGGGCAACCGTAATCACAAACCTCATATCCGCCATCCCATACATTGGAGACTCACTCGTAATTTGAATCTGAGGGGGCCCTTCAATTAACAGTGCCACCTTAACACGCTTTACCACCCTACACTTTCTACTACCATTTATCCTGCTAGCAACCATCATCACCCACCTCATATTTCTACACGAGCAAGGCTCTTTTAACCCCTTAGGCCTTTCTCCCAACGCCGACAAAGTCCCATTCCACCCATACTTCTCCATAAAAGATGCCCTAGGCACAATACTTGCCATCACCCTACTCTCAACCCTAGCCCTATATTTCCCAAACCTCTTGGGTGACCCAGAAAACTTTACTCCAGCTGACCCTATAAAAACCCCAAACCACATCAAACCCGAATGATACTTCCTATTTGCCTACACGATCCTCCGATCTATTCCTAATAAACTAATAGGGGTATTAGCCATATTCTCATCAATCCTTATCCTTTTCCTAATACCAGCACTCCATACATCAAAACGACAATCAATAACAATACGCCCGCTCTCCCAGCTCCTATTCTGAACCCTTATCGCCGACTTTTTTGTACTAACCTGAATCGGAGGACAGCCCGTACAAAACCCATACACCTTAATTGGCCAAATAGCCTCTATCACTTACTTTACAATTATCCTCATCCTCATACCAACAGCGGGCAAAATAGAAAACTGAATTATTAAACCCCTACAATACCGATACCACGGACC-TTGTGAACATTAATCGCTGACTTTTCTCCACCAACCACAAAGACATCGGCACCCTTTACTTTATCTTCGGAGCCTGAGCGGGAATAGTTGGAACAGCCCTAAGCCTCCTTATTCGCACAGAACTAAGTCAACCCGGACCCCTCATAGGAGACGACCAAATCTATAATGTTATTGTTACCGCACATGCCTTTATTATGATTTTCTTCATAGTAATACCTATTATAATTGGGGGATTTGGAAACTGACTACTACCACTAATGATTGGCGCACCCGACATAGCATTCCCACGAATAAATAATATAAGCTTCTGATTACTCCCACCATCATTCACCTTACTGCTTTTCTCCGCTTTCATCGAAACTGGGGCTGGAACCGGATGAACAGTCTATCCGGCCCTAGCAGGAAACCTAGCCCACGCCGGACCCTCCGTAGACTTAACCATTTTCTCCCTTCACCTCGCAGGAGTATCTTCAATCTTAGGGGCAATTAACTTTATTACCACAGCCATTAACATAAAACCCCCAGCAATATCACAATACCAAACACCTCTTTTTGTATGATCTGTATTAATTACAGCTGTGCTTCTCCTACTTTCCCTACCAGTATTAGCTGCAGGAATCACCATACTACTCACCGATCGAAACCTAAATACAACCTTCTTTGACCCCTCAGGCGGCGGAGACCCTATCCTATATCAACATCTCTTCTGATTCTTCGGCCACCCAGAAGTTTATATCCTTATCCTCCCAGGGTTTGGAATAATCTCCCATGTAGTCACCTTCTACTCAGGAAAAAAAGAACCATTCGGGTATATGGGAATAGTGTGAGCCATAATATCAATTGGCTTCCTGGGTTTCATTGTTTGAGCTCATCACATATTTACAGTAGGAATAGACGTTGATACCCGAGCATATTTTACTTCCGCTACAATAGTAATCGCTATCCCCACCGGAGTAAAAGTATTCAGCTGGCTAGCAACAATCTACGGAGGAATTATAAACTGACAAGCCCCCATACTCTGAGCATTAGGCTTTATTTTCTTATTTACAGTAGGAGGACTAACAGGAATCGTCCTAGCCAACTCATCACTAGACATTATTCTCCACGACACTTATTACGTAGTAGCACACTTCCACTACGTACTATCAATAGGGGCAGTATTCGCCATTATAAGTGGATTTACCCACT-GGTTCCCCCTA-TTCACAGGATTTACCCTTCACCCAGCATGAACAAAAG-CCCAATTCGCAATCATATTCACAGGAGTAAATCTAACCTTCTTCCCACAACACTTCTTAGGCCTTTCCGGGATGCCACGACGATATTCAGACTACCCAGACGCATATTCATTCTGAAATATATTATCATCAATTGGGTCGTTAATTTCTATAGTCTCTGTAATTCTCCTCATATTCATTGTATGAGAAGCATTCTCTTCAAAACGTAAAATTGAGACACCAGAAATAACCATCACAAATATTGAGTGGCTCAACAACTGCCCACCATCTCACCACACTTACGAAGAACCAGTATTTGTCCAAGCACGGCCTAAACCAACAAAAACCCCACCTCCTAACAACAAGGA---------CAGGATGGCAAACCCGATACATCTTGGGCTCCAGGATGCAATATCCCCACTAATAGAAGAGCTCCTCTATTTTCATGACCACACACTAATAGTTATTTTCCTAATTAGTATACTCGTACTCTACACCATCTCCGTCCTCCTAATATCAAAACTTTATCACACAAATGCCACAGACGTACAAGAAGTAGAAATAATTTGAACAATCCTACCGGCCATCATCCTAATTACCATCGCCCTCCCATCCCTACGTACCCTATACCTGATAGATGAAACCAGTAACCCCTGTCTAACAATTAAGGTTATTGGACATCAATGATATTGAACTTATGAATATACAGACTTCTCGCAACTAGAGTTTGATTCCTACATAGTACCAGCACAAGACCTTATACCAGGACACTTCCGCCTACTAGAAGTGGATCACCGTATAATTGCTCCAACAAGTGCAACCACCCGGACACTAATCACAGCAGAAGACGTACTACACTCGTGAGCAGTACCATCCCTTGGAATTAAAATAGACGCGGTACCAGGACGCCTAAACCAAACCTCAATTACACTGGCTAGCCCAGGAATTTTTTACGG-TCAGTGTTCTGAAATTTGTGGAGCTAACCACAGCTTTATACCCGTTGTTGTAGAAGCAATCCCAATACAACACTTTCAAAAATGACTAGAAATAAGTGTTTCCTCATATGACACACCAGACACACCCATTCCACATAGTAAACCCCAGCCCTTGGCCAATTATAGGGGCCATAGCCGCCATAATATTAACCACAGGGCTAGTCCTATGGTTTCACTGCAACCTCAAACTACTCCTAGCCATAGGACTAATCTCAACCCTAACAGTTATATATCAGTGATGACGTGATATCGTACGAGAGAGCACCTACTTAGGTCACCACACCCCTCCAGTCCAAAAAGGTCTGCGCTACGGCATAATCTTATTTATTACATCAGAAGTCTTCTTCTTCCTCGGTTTCTTTTGGGCATTTTACCACTCCAGCCTATCCCCAACCCCAGAGCTAGGAGGACAATGACCCCCAACTGGAATTATTCCACTAGATCCATTTGAGGTCCCACTTTTAAACACCGCCGTACTACTGGCCTCAGGAGTTACAGTAACATGAGCACACCACAGCCTAATAGAAGCCAACCGAACACCAACCATCCACGCCCTTATACTCACCATCGTCCTAGGACTGTACTTCACCGCCCTTCAAGCGATAGAATATTATGAAGCCCCATTCACCATCGCAGACAGCAGCTACGGATCCACCTTTTTCGTAGCCACAGGCTTCCACGGCCTACATGTAATTATTGGATCAACATTTCTAATAATTTGCTTATACCGACAAATCATGCATCACTTCACCTCAAATCACCACTTCGGCTTTGAAGCTGCCGCTTGGTACTGACACTTCGTAGACGTAGTATGACTATTCCTCTACATCTCAATCTACTGATGAGGATCCTATCAGCTTCCTTGTAGCTACTCCTTTTGTGCTTTACATTATCCTAATTTTGATCGCAGTCGCATTCCTGACAGCCTTAGAGCGAAAAATTATTGGCTACATGCAACTACGAAAAGGCCCGAATATCGTGGGACCATTAGGCCTTCTACAACCGTTTGCTGATGGCCTCAAACTTATTATTAAAGAATTAACTTTCCCAATTCTTGCCACCCCCACCCTATTCATCATATCCCCCGCAGTAGCTTTAATCTTATCCCTTACAATATGAGCCCCACTTCCTATACCGTTCTCCCTAGCTAACCTAAACCTAGGAATACTGTTTATACTGGCTATATCCAGCCTAGCAGTATACTCATTATTATGGTCCGGCTGAGCATCAAACTCAAAATACGCCCTAATAGGCGCCTTGCGAGCAGTCGCCCAGACCATCTCCTACGAAGTAACCCTAGCTATTATTATCCTTTCAGTCGTTCTCCTCAGCGGCGGATTTTCACTACATACACTAACTATTACCCAAGAACCAACATATCTAGCACTTACCACATGACCTTTATTAATAATATGATATACATCAACGCTTGCAGAAACAAACCGAGCCCCATTTGACTTAACAGAAGGAGAATCAGAACTGGTTTCAGGATTTAATGTTGAATACAGCGCAGGACTCTTCACACTTTTTTTCCTTGCCGAATATGCTAATATTTTATTAATAAACATTTTAACCACAATCCTATTCCTCAATACATCAACCAACCTCCCAGTACAAACACTTTTTACTATAACCCTAATAAGTAAGTCTATCCTACTAACTATAGGATTCCTATGAATTCGAGCATCATACCCTCGATTCCGCTACGACCAACTAATACACCTTCTATGAAAAAGCTTCCTGCCAGCTACCCTAACATTCTGTTTATGACACATATCATTCCCAGTGTCAATATTTGGCCTCCCGGCGATAA---GGATGCCACTATTCCACCCCATCATTTTAACCACGCTAACTATCACCACATTTATTTTCCTCTCCTCCACACACCTTGTGCTAATATGAGTAGCACTAGAACTGAGCACACTGGCAATCCTACCCTTAATCGCCACTAAATCACACCCACGAGCTATCGAGGCATCCACAAAATACTTCCTCACCCAGGCGACCGCTTCCGCATTAATTATTTTCTCAGGAACATTAAACTATACCATGACGGGCAACTGCCAAATTACTGAATTAACTAACCCAAACTTGATAATAACCTTAATATTAGCCGTATTTATCAAAATTGGACTAGTCCCCTTCCACTTTTGAGTCCCTGAAACACTTCAAGGTATAACTCCAACTGCCGCCATCTTCCTTCTAACCTGACAAAAGCTTGGCCCACTAATTATATTATTCTTAATGAGCCCACTAATTAACTTCGAAGTCCTATCCATAATAGCTGTTCTATCCGCCGTAGTCGCCGGCTGACTTGGACTAAACCAAACTCAAGTCCGAAAACTAGTAGCCATATCCTCCATCGCCCAAATGGCTTGAACCCTCGTAATCGTTAAATACGCACCATCACTTATAATCCTAGCTTTCTACTTATATTCTCTCACCATCTCCGCCACACTCCTCACCCTAGATAAGTTATCAATAACATCTATTAGCAACCTCCTCCTTTCATTCTCAAAAGCCCCCATTATTACATTTTTATTAATAATCTCCCTACTGTCCTTATCAGGCCTACCACCTTTAGCCGGCTTCCTGCCAAAATGACTAACAATTGATATACTCGTTGCAGAAGAAGCCATTTGAGTAGCCTTTATAATACTTATAGCCTCGCTCCTAAGCCTATTCTTCTACATACGACTATGATATAACTCCGCATCCACCATCCCACCCAACACTACTAATACCTCCCGGCTATGACGAAAACCCCTCCCCAAAACAAACCTCACAATCAACTTTCTAAGCATGACCGCCTTTATTTTCATACTAGTAGCCACCCTAATGAAGGCCCTCACAAAATAA------------------A----TAAACCTGCTCACCATATTTATACTAGCTATAGCCACTACAGTAGCTGTAGCGGCAGCGAACCTATTAATAGCTGAAATAACCCCAGACCCTGAAAAACTATCACCGTATGAATGTGGATTTGACCCCTTAGGTTCAGCCCGACTGCCCTTCTCCATTCGATTTTTTATAATCGCTATCTTATTCCTACTATTTGATCTAGAAATCGCTATTCTTTTACCCCTCGCATGAGCCCTTCAACTCACAAATCTTATTAAGGCAACTTCATGAGCCATCATTATTTTTTTATTTATATTTATTGGCCTGACATACGAATGGTTACAAGGTGGTCTAGAATGAGCAGAATAGATGCTAAAACTTATAGTACCAACAATAATAGTTATTCCTTCTACCTGTTTTACACACCCAAAAACAGTCTGACTATTACCAACAGCTTATTCTACAGCCATCATCATTTTAACTTTACTTATCCTAAATCCAGGAGACAGCCTCACAAGCACTAATGGTCTCCTCCTAGGAAGTGATCAAGTCTCTACACCTCTACTAATCCTGTCCTGCTGGCTACTCCCACTTATATTTATGGCTAGCCAAAACTCCATATCCCAGGACCCGTATCCCCAAAAACGACTGTTTATTACCACTTTAGCCCTCCTCCAACTAGCTTTACTACTAGTATTTATAGCCTTAGATCTAATATTATTTTACATTGCCTTCGAAGCGACCCTTATTCCAACGCTAATGGTAATTGCTCGCTGAGGCACCCAAACAGAACGGCTAGGAGCCGGATTATATTTTTTATTTTATACTATTACTAGCTCAATACCACTTTTAATAGCCCTTCTATGAGTATATAATATAAAAGGGACCCTATCTATTCTACTTCTACAACTGACACCACCAATAACCCACATATTCTGGACAGACATGATATTATGGCTATCTCTGCTGCTCGCCTTCCTAGTAAAAATTCCAATTTATGGTCTACACCTATGACTTCCCAAAGCACACGTAGAAGCTCCAATCGCCGGATCCATAGTCCTAGCTGCAGTACTCCTAAAACTAGGTGGCTATGGACTATTACGAGTTACAAACCTATTAACAGAACAAACCAACTCCTCCTATGTTTTACCACTAGCAGTAGCACTATGGGGATCACTCATAACTGGTATAATCTGCTTACGACAAACAGACTTAAAATCCCTAATCGCCTACTCATCAGTTAGTCACATAGGTCTTATAACAAGCTCAATCCTAACCCGTAACCAACTAGCCCTATCTGGTTCAATAATTATAATAATCGCCCACGGCCTAACATCCTCTATACTATTTTGCTTAGCCAATGTTACCTACGAGCGAACACACTCACGAACGCTCCTGCTAGCCCAAGGAGTGCAACTCACCACCCCAGCCATAACTTCCTGATGACTCCTAGCCTGCCTGACAAATATAGCACTCCCTCCAACAATTAATTTTATTGGAGAACTCACTCTTACAGTATCCTTATTTAGCTGATCAGATATTACTATTTTACTAACAGGACTAAGTATATTCATTACTTCAGTCTACACCCTCCATATATTCTCGTCCACTCAACAAGGAACACTCCCCTCGCACATTATTACAATAACCCCCACCCAAACACGCGAACACCTCCTAATACTTATACACTCTGCCCCGTCTCTTACTCTGATTATAGCCCCACAACTAATATGCTCTTAA------ACTGCCCCCACCGACCTCTTATTTATTTGTTCTTTTATTATTTTTACCATTGGACTCACCTTCCATCACACCCACTTACTCTCAGCCTTACTATGCCTAGAAGGCATAATATTATCAGTATTTATACTATTAACAATCTGATCCATCAATTTTAATACCTCTTCCTTTATTTTACCCCTAACAGTATTAACCCTCTCAGCCTGTGAAGCAGGCATTGGCCTTGCCCTACTAATTGCCTCAGCTCGCACACACAGTACAACTAACCTTAAAAATCTTAATCTCCTCCAATGCTAAGTGAACCCAGCTATCA-------------TGTTACTTATGTTGCCACTGACTATTATATTATCATCGCTATTTATCCCTTATT-CAAAAGCTAACAAA--TCATCCCACGCTAAACTGTTGGCAACAAAATTAGCGTTCTTCTCCAGCTTACTCCCCCTAGCCTTCTTTATCTATGATGGACTAGTCGTCACCTCATTTGAGGCCCAATGATTGGCCTTCGACGTATGCCCCGTCCATGTCAGCTTCACATTGGACACATACTCAGTCTTCTTCCTACCAATCTCACTCTTCATCGCTTGATCTATTATAGAATTTACCGTTAAATATATAAAATCAGACCCAAAAATTGACACCTTTTTCCGCTACCTTATTATTTTTACCCTAATAATGATAATTCTAGTAACCGCTGAAAATGTATACCAGCTTTTCATTGGCTGAGAGGGCGTAGGTATTATATCCTACATACTCATCAACTGATGATCCTACCGACCAGCCTCCAATAAAGCAGCCCTTCAGGCAGTAATTTACAACCGCCTAGCAGACATCGGCCTAACAGTTGCCCTAGCATGAATAGTTGTAAATAACCTCTCTCTAGATATCCAAGGAGTACAAGCCTCACCAGACCTCGCCATTATTCCCGCGTTCGGGTTCATCCTAGCAGCTGCTGGAAAATCTGCCCAATTCGGCTTCCACCCATGACTGCCAGCAGCAATAGAAGGTCCAACCCCTGTATCCGCCCTACTACACTCAAGTACTATAGTAGTAGCGGGCGTCTTTCTGCTAATTCGAGCCTCAAGCTTTATCCACTGTAATAAAATAACAACCATTGCCTGCCTATTACTAGGAGCACTCACATCCCTGCTCGCAGCAACCTGCGCATTAACTCAAAATGATATAAAAAAAATTATTGCCTACTCTACTACCAGCCAATTAGGACTAATAATAACAGCAATCGGACTAAAACAGCCTGAACTTGCATTCATGCACATCGCAACACATGCCTTCTTTAAAGCAATACTATTTCTATGCGCTGGCGCAATCATCCACAACCTCAATAATGAACAAGATATCCGAAAAATAGGAGGCCTAAAAAAAGCAATACCAATTACTTCTGCCTGCTTAGTTATCGGCGCCCTATCCTTATCAGGAATGCCATTCATGTCTGGATTTTACTCAAAAGACGCCATCATTGAAGCACTTAACACCTCCAACATCAACTCTATCTCACTGGCTATAACCCTAATTGCCACCACCTTCACAGTACTCTACAACTTACGCATAATTTACTATGTGGTATTAGGCACCCCCCGACTACTACCACTTGCTTCACTCCCAGAAACCCGCCAAATCATTAGCCCTATCCTACGATTAGCCGTAGGAAGTATTGCAGCAGGACTCTTAATTTCAACCAACATCCTCCCTTGCAACATCCCACAACTAACCATGCCACCAGAAGTTAAGCTCGCCGCCATAATTATTACAGCCCTAGCACTATTAACTGGTGTAGCCCTAATTCTCACAGCCCCACGACTCCCACTATCCAACAAGGGCACCCAATCTCCCCTCCCTTTCAAAATAGCCAACTTCTACTTCATCCTTCACCACATCCTACCAGCTGCCATCCTATGAATGAGCCAAAAGCTATCAGGTCATCTAACCGATCAAACACACTATGAAGCTCTAGGACCCAAGATATTAACTGTTCTCCAAATCCTGATAACCAAAATTATGACAAATCTTCACAAAGCCCGAATTAACCCATACCTAAAAGTTATTATTTTAGGTATTACATTATTCCTACTGTTATATTACCCCTCAATGACCGAAGGGCCCCCCGCCGATGACCCCGGATAA---------ATGGGAATGACTTTTTTTGCTCTTAGTAGTTTAATATTAGTAGGTGTGGTATTAGTATCAGCGGGGGCCACGACCCATTATGGGGTAATTAGTCTGCTTTTTGCTGCATTATCAAGTGGTGGCCTGCTGGCAGTTGGGGGAGGAAGCTTCATACCAATAGTAGTATTATTAATTTATTTAGGTGGGCTATTGGTAGTATTTACTTTTTGTGTAGGATTTACTGATGATAAATATTGTGAGTTTTGAGGGGTTGGGGCATCTAAGGGGTTGGTTGGTGTTTGTGGGGCTGGGTTGATGGTGGTAGGTTATCGTGCGTATAAGCACATATGG----ATAGGTGTTTTAGGGGGGTTTT--CGGATGCCGTAGAGAATTGGAGTGGTGACATCAGTGATGAGTTGCTAGGGGCCAGCTTGTTCTATCTGGAGGGCTGAGGGTTTGTTGTATTAAGCGGCTGGGCCCTGTTAATTGTGCTATTTACAATTATAAGTATTATCCGGGGTCATCGGCGGGGGGCCCTTCGGTCATTGAGG

*Tomistoma schlegelii 2*

ATGACTATGGCCACAAATCTAAACTTATTTGATCAGTTCTTAATTCCTAAACTTCTTGGAGTACCCATACTAATTCCAGCCTTGTTAATAGCATCTATTTTATTGTACAACCCAGAAGACCGATGATTATCTAACCCAACAACAACCATCCAATACTGGTTAATCACAAAAGTGACCAAACAAATTATAACCCCAGTAAATCAGCCTGGACATAAATGATCATTAATATTAATTTCCCTCCTGACATTTCTTCTTATTAATAATCTTCTAGGCCTACTCCCATACACCTTTACACCAACGACACAGTTATCTATAAACCTAGCCTTAGCCCTACCTCTATGGATAGCAACAGTGTTAACTGGTCTACGAAATAAACCAAGCGCCTCCTTAGCCCATCTACTCCCAGAAGGAACTCCAACACCACTAATTCCAATCTTAATTTTGATCGAATCCATCAGCCTGCTAATCCGACCAATCGCTCTTGCCGTACGACTAACAGCCAATCTCACTGCCGGACACCTTTTAATTCACCTAATTGCCGCTACAGCATTAAACCTATTAACAACATCCCCACTACTAGCCGTACTGACCCTAATTATTCTAGTACTACTAATACTACTAGAAATTGCAGTAGCAATAATTCAAGCCTATGTTTTTACCCTGCTTCTATCCCTCTATCTACAAGAAAATGTATAAATGCCTCAACTAAACCCCAAACCTTGACTAATAGTTTTCCTTACCACCTGATTCATACTAATTATTGTTACACACCCCAAAATCGCCTCCTTAAAGCTTCTAAATAGCCCAGTACCTTTTCATCCAACCCCTATT-AAACCATGACTATGGCCACAAATCTAA---------------------ATGGCCCACCAACTACGAAAGTCCCACCCCCTACTAAAACTCATAAACCACTCTCTAATTGACCTCCCCACTCCATCCAACATCTCCTACTGATGAAACTTTGGATCACTTCTAGGGTTTACCCTTTTAATCCAAATCCTATCAGGCATTCTACTAATAATACATTTCCTAGCACATGACACCATAGCCTTCGCATCCGTCGCTTACACATCACGAGAAGTCTGATACGGCTGACTTATCCGAAGCCTTCACGCAAACGGAGCTTCCCTATTCTTCTTTTGCATCTTCTTACACATTGGACGTGGCCTCTACTACGGCTCTTATCTGCACGAAAACACATGGAACATCGGAGTCATTTTACTCCTCCTGCTAATAGCAACAGCTTTCATAGGTTACGTACTACCCTGAGGACAAATATCATTCTGAGGGGCAACCGTAATCACAAACCTCATATCCGCCATCCCATACATTGGAGACTCACTCGTAATTTGAATCTGAGGGGGCCCTTCAATTAACAGTGCCACCTTAACACGCTTTACCACCCTACACTTTCTACTACCATTTATCCTGCTAGCAACCATCATCACCCACCTCATATTTCTACACGAGCAAGGCTCTTTTAACCCCTTAGGCCTTTCTCCCAACGCCGACAAAGTCCCATTCCACCCATACTTCTCCATAAAAGATGCCCTAGGCACAATACTTGCCATCACCCTACTCTCAACCCTAGCCCTATATTTCCCAAACCTCTTGGGTGACCCAGAAAACTTTACTCCAGCTGACCCTATAAAAACCCCAAACCACATCAAACCCGAATGATACTTCCTATTTGCCTACACGATCCTCCGATCTATTCCTAATAAACTAATAGGGGTATTAGCCATATTCTCATCAATCCTTATCCTTTTCCTAATACCAGCACTCCATACATCAAAACGACAATCAATAACAATACGCCCGCTCTCCCAGCTCCTATTCTGAACCCTTATCGCCGACTTTTTTGTACTAACCTGAATCGGAGGACAGCCCGTACAAAACCCATACACCTTAATTGGCCAAATAGCCTCTATCACTTACTTTACAATTATCCTCATCCTCATACCAACAGCGGGCAAAATAGAAAACTGAATTATTAAACCCCTACAATACCGATACCACGGACC-TTGTGAACATTAATCGCTGACTTTTCTCCACCAACCACAAAGACATCGGCACCCTTTACTTTATCTTCGGAGCCTGAGCGGGAATAGTTGGAACAGCCCTAAGCCTCCTTATTCGCACAGAACTAAGTCAACCCGGACCCCTCATAGGAGACGACCAAATCTATAATGTTATTGTTACCGCACATGCCTTTATTATGATTTTCTTCATAGTAATACCTATTATAATTGGGGGATTTGGAAACTGACTACTACCACTAATGATTGGCGCACCCGACATAGCATTCCCACGAATAAATAATATAAGCTTCTGATTACTCCCACCATCATTCACCTTACTGCTTTTCTCCGCTTTCATCGAAACTGGGGCTGGAACCGGATGAACAGTCTATCCGGCCCTAGCAGGAAACCTAGCCCACGCCGGACCCTCCGTAGACTTAACCATTTTCTCCCTTCACCTCGCAGGAGTATCTTCAATCTTAGGGGCAATTAACTTTATTACCACAGCCATTAACATAAAACCCCCAGCAATATCACAATACCAAACACCTCTTTTTGTATGATCTGTATTAATTACAGCTGTGCTTCTCCTACTTTCCCTACCAGTATTAGCTGCAGGAATCACCATACTACTCACCGATCGAAACCTAAATACAACCTTCTTTGACCCCTCAGGCGGCGGAGACCCTATCCTATATCAACATCTCTTCTGATTCTTCGGCCACCCAGAAGTTTATATCCTTATCCTCCCAGGGTTTGGAATAATCTCCCATGTAGTCACCTTCTACTCAGGAAAAAAAGAACCATTCGGGTATATGGGAATAGTGTGAGCCATAATATCAATTGGCTTCCTGGGTTTCATTGTTTGAGCTCATCACATATTTACAGTAGGAATAGACGTTGATACCCGAGCATATTTTACTTCCGCTACAATAGTAATCGCTATCCCCACCGGAGTAAAAGTATTCAGCTGGCTAGCAACAATCTACGGAGGAATTATAAACTGACAAGCCCCCATACTCTGAGCATTAGGCTTTATTTTCTTATTTACAGTAGGAGGACTAACAGGAATCGTCCTAGCCAACTCATCACTAGACATTATTCTCCACGACACTTATTACGTAGTAGCACACTTCCACTACGTACTATCAATAGGGGCAGTATTCGCCATTATAAGTGGATTTACCCACT-GGTTCCCCCTA-TTCACAGGATTTACCCTTCACCCAGCATGAACAAAAG-CCCAATTCGCAATCATATTCACAGGAGTAAATCTAACCTTCTTCCCACAACACTTCTTAGGCCTTTCCGGGATGCCACGACGATATTCAGACTACCCAGACGCATATTCATTCTGAAATATATTATCATCAATTGGGTCGTTAATTTCTATAGTCTCTGTAATTCTCCTCATATTCATTGTATGAGAAGCATTCTCTTCAAAACGTAAAATTGAGACACCAGAAATAACCATCACAAATATTGAGTGGCTCAACAACTGCCCACCATCTCACCACACTTACGAAGAACCAGTATTTGTCCAAGCACGGCCTAAACCAACAAAAACCCCACCTCCTAACAACAAGGA---------CAGGATGGCAAACCCGATACATCTTGGGCTCCAGGATGCAATATCCCCACTAATAGAAGAGCTCCTCTATTTTCATGACCACACACTAATAGTTATTTTCCTAATTAGTATACTCGTACTCTACACCATCTCCGTCCTCCTAATATCAAAACTTTATCACACAAATGCCACAGACGTACAAGAAGTAGAAATAATTTGAACAATCCTACCGGCCATCATCCTAATTACCATCGCCCTCCCATCCCTACGTACCCTATACCTGATAGATGAAACCAGTAACCCCTGTCTAACAATTAAGGTTATTGGACATCAATGATATTGAACTTATGAATATACAGACTTCTCGCAACTAGAGTTTGATTCCTACATAGTACCAGCACAAGACCTTATACCAGGACACTTCCGCCTACTAGAAGTGGATCACCGTATAATTGCTCCAACAAGTGCAACCACCCGGACACTAATCACAGCAGAAGACGTACTACACTCGTGAGCAGTACCATCCCTTGGAATTAAAATAGACGCGGTACCAGGACGCCTAAACCAAACCTCAATTACACTGGCTAGCCCAGGAATTTTTTACGG-TCAGTGTTCTGAAATTTGTGGAGCTAACCACAGCTTTATACCCGTTGTTGTAGAAGCAATCCCAATACAACACTTTCAAAAATGACTAGAAATAAGTGTTTCCTCATATGACACACCAGACACACCCATTCCACATAGTAAACCCCAGCCCTTGGCCAATTATAGGGGCCATAGCCGCCATAATATTAACCACAGGGCTAGTCCTATGGTTTCACTGCAACCTCAAACTACTCCTAGCCATAGGACTAATCTCAACCCTAACAGTTATATATCAGTGATGACGTGATATCGTACGAGAGAGCACCTACTTAGGTCACCACACCCCTCCAGTCCAAAAAGGTCTGCGCTACGGCATAATCTTATTTATTACATCAGAAGTCTTCTTCTTCCTCGGTTTCTTTTGGGCATTTTACCACTCCAGCCTATCCCCAACCCCAGAGCTAGGAGGACAATGACCCCCAACTGGAATTATTCCACTAGATCCATTTGAGGTCCCACTTTTAAACACCGCCGTACTACTGGCCTCAGGAGTTACAGTAACATGAGCACACCACAGCCTAATAGAAGCCAACCGAACACCAACCATCCACGCCCTTATACTCACCATCGTCCTAGGACTGTACTTCACCGCCCTTCAAGCGATAGAATATTATGAAGCCCCATTCACCATCGCAGACAGCAGCTACGGATCCACCTTTTTCGTAGCCACAGGCTTCCACGGCCTACATGTAATTATTGGATCAACATTTCTAATAATTTGCTTATACCGACAAATCATGCATCACTTCACCTCAAATCACCACTTCGGCTTTGAAGCTGCCGCTTGGTACTGACACTTCGTAGACGTAGTATGACTATTCCTCTACATCTCAATCTACTGATGAGGATCCTATCAGCTTCCTTGTAGCTACTCCTTTTGTGCTTTACATTATCCTAATTTTGATCGCAGTCGCATTCCTGACAGCCTTAGAGCGAAAAATTATTGGCTACATGCAACTACGAAAAGGCCCGAATATCGTGGGACCATTAGGCCTTCTACAACCGTTTGCTGATGGCCTCAAACTTATTATTAAAGAATTAACTTTCCCAATTCTTGCCACCCCCACCCTATTCATCATATCCCCCGCAGTAGCTTTAATCTTATCCCTTACAATATGAGCCCCACTTCCTATACCGTTCTCCCTAGCTAACCTAAACCTAGGAATACTGTTTATACTGGCTATATCCAGCCTAGCAGTATACTCATTATTATGGTCCGGCTGAGCATCAAACTCAAAATACGCCCTAATAGGCGCCTTGCGAGCAGTCGCCCAGACCATCTCCTACGAAGTAACCCTAGCTATTATTATCCTTTCAGTCGTTCTCCTCAGCGGCGGATTTTCACTACATACACTAACTATTACCCAAGAACCAACATATCTAGCACTTACCACATGACCTTTATTAATAATATGATATACATCAACGCTTGCAGAAACAAACCGAGCCCCATTTGACTTAACAGAAGGAGAATCAGAACTGGTTTCAGGATTTAATGTTGAATACAGCGCAGGACTCTTCACACTTTTTTTCCTTGCCGAATATGCTAATATTTTATTAATAAACATTTTAACCACAATCCTATTCCTCAATACATCAACCAACCTCCCAGTACAAACACTTTTTACTATAACCCTAATAAGTAAGTCTATCCTACTAACTATAGGATTCCTATGAATTCGAGCATCATACCCTCGATTCCGCTACGACCAACTAATACACCTTCTATGAAAAAGCTTCCTGCCAGCTACCCTAACATTCTGTTTATGACACATATCATTCCCAGTGTCAATATTTGGCCTCCCGGCGATAA---GGATGCCACTATTCCACCCCATCATTTTAACCACGCTAACTATCACCACATTTATTTTCCTCTCCTCCACACACCTTGTGCTAATATGAGTAGCACTAGAACTGAGCACACTGGCAATCCTACCCTTAATCGCCACTAAATCACACCCACGAGCTATCGAGGCATCCACAAAATACTTCCTCACCCAGGCGACCGCTTCCGCATTAATTATTTTCTCAGGAACATTAAACTATACCATGACGGGCAACTGCCAAATTACTGAATTAACTAACCCAAACTTGATAATAACCTTAATATTAGCCGTATTTATCAAAATTGGACTAGTCCCCTTCCACTTTTGAGTCCCTGAAACACTTCAAGGTATAACTCCAACTGCCGCCATCTTCCTTCTAACCTGACAAAAGCTTGGCCCACTAATTATATTATTCTTAATGAGCCCACTAATTAACTTCGAAGTCCTATCCATAATAGCTGTTCTATCCGCCGTAGTCGCCGGCTGACTTGGACTAAACCAAACTCAAGTCCGAAAACTAGTAGCCATATCCTCCATCGCCCAAATGGCTTGAACCCTCGTAATCGTTAAATACGCACCATCACTTATAATCCTAGCTTTCTACTTATATTCTCTCACCATCTCCGCCACACTCCTCACCCTAGATAAGTTATCAATAACATCTATTAGCAACCTCCTCCTTTCATTCTCAAAAGCCCCCATTATTACATTTTTATTAATAATCTCCCTACTGTCCTTATCAGGCCTACCACCTTTAGCCGGCTTCCTGCCAAAATGACTAACAATTGATATACTCGTTGCAGAAGAAGCCATTTGAGTAGCCTTTATAATACTTATAGCCTCGCTCCTAAGCCTATTCTTCTACATACGACTATGATATAACTCCGCATCCACCATCCCACCCAACACTACTAATACCTCCCGGCTATGACGAAAACCCCTCCCCAAAACAAACCTCACAATCAACTTTCTAAGCATGACCGCCTTTATTTTCATACTAGTAGCCACCCTAATGAAGGCCCTCACAAAATAA------------------A----TAAACCTGCTCACCATATTTATACTAGCTATAGCCACTACAGTAGCTGTAGCGGCAGCGAACCTATTAATAGCTGAAATAACCCCAGACCCTGAAAAACTATCACCGTATGAATGTGGATTTGACCCCTTAGGTTCAGCCCGACTGCCCTTCTCCATTCGATTTTTTATAATCGCTATCTTATTCCTACTATTTGATCTAGAAATCGCTATTCTTTTACCCCTCGCATGAGCCCTTCAACTCACAAATCTTATTAAGGCAACTTCATGAGCCATCATTATTTTTTTATTTATATTTATTGGCCTGACATACGAATGGTTACAAGGTGGTCTAGAATGAGCAGAATAGATGCTAAAACTTATAGTACCAACAATAATAGTTATTCCTTCTACCTGTTTTACACACCCAAAAACAGTCTGACTATTACCAACAGCTTATTCTACAGCCATCATCATTTTAACTTTACTTATCCTAAATCCAGGAGACAGCCTCACAAGCACTAATGGTCTCCTCCTAGGAAGTGATCAAGTCTCTACACCTCTACTAATCCTGTCCTGCTGGCTACTCCCACTTATATTTATGGCTAGCCAAAACTCCATATCCCAGGACCCGTATCCCCAAAAACGACTGTTTATTACCACTTTAGCCCTCCTCCAACTAGCTTTACTACTAGTATTTATAGCCTTAGATCTAATATTATTTTACATTGCCTTCGAAGCGACCCTTATTCCAACGCTAATGGTAATTGCTCGCTGAGGCACCCAAACAGAACGGCTAGGAGCCGGATTATATTTTTTATTTTATACTATTACTAGCTCAATACCACTTTTAATAGCCCTTCTATGAGTATATAATATAAAAGGGACCCTATCTATTCTACTTCTACAACTGACACCACCAATAACCCACATATTCTGGACAGACATGATATTATGGCTATCTCTGCTGCTCGCCTTCCTAGTAAAAATTCCAATTTATGGTCTACACCTATGACTTCCCAAAGCACACGTAGAAGCTCCAATCGCCGGATCCATAGTCCTAGCTGCAGTACTCCTAAAACTAGGTGGCTATGGACTATTACGAGTTACAAACCTATTAACAGAACAAACCAACTCCTCCTATGTTTTACCACTAGCAGTAGCACTATGGGGATCACTCATAACTGGTATAATCTGCTTACGACAAACAGACTTAAAATCCCTAATCGCCTACTCATCAGTTAGTCACATAGGTCTTATAACAAGCTCAATCCTAACCCGTAACCAACTAGCCCTATCTGGTTCAATAATTATAATAATCGCCCACGGCCTAACATCCTCTATACTATTTTGCTTAGCCAATGTTACCTACGAGCGAACACACTCACGAACGCTCCTGCTAGCCCAAGGAGTGCAACTCACCACCCCAGCCATAACTTCCTGATGACTCCTAGCCTGCCTGACAAATATAGCACTCCCTCCAACAATTAATTTTATTGGAGAACTCACTCTTACAGTATCCTTATTTAGCTGATCAGATATTACTATTTTACTAACAGGACTAAGTATATTCATTACTTCAGTCTACACCCTCCATATATTCTCGTCCACTCAACAAGGAACACTCCCCTCGCACATTATTACAATAACCCCCACCCAAACACGCGAACACCTCCTAATACTTATACACTCTGCCCCGTCTCTTACTCTGATTATAGCCCCACAACTAATATGCTCTTAA------ACTGCCCCCACCGACCTCTTATTTATTTGTTCTTTTATTATTTTTACCATTGGACTCACCTTCCATCACACCCACTTACTCTCAGCCTTACTATGCCTAGAAGGCATAATATTATCAGTATTTATACTATTAACAATCTGATCCATCAATTTTAATACCTCTTCCTTTATTTTACCCCTAACAGTATTAACCCTCTCAGCCTGTGAAGCAGGCATTGGCCTTGCCCTACTAATTGCCTCAGCTCGCACACACAGTACAACTAACCTTAAAAATCTTAATCTCCTCCAATGCTAAGTGAACCCAGCTATCA-------------TGTTACTTATGTTGCCACTGACTATTATATTATCATCGCTATTTATCCCTTATT-CAAAAGCTAACAAA--TCATCCCACGCTAAACTGTTGGCAACAAAATTAGCGTTCTTCTCCAGCTTACTCCCCCTAGCCTTCTTTATCTATGATGGACTAGTCGTCACCTCATTTGAGGCCCAATGATTGGCCTTCGACGTATGCCCCGTCCATGTCAGCTTCACATTGGACACATACTCAGTCTTCTTCCTACCAATCTCACTCTTCATCGCTTGATCTATTATAGAATTTACCGTTAAATATATAAAATCAGACCCAAAAATTGACACCTTTTTCCGCTACCTTATTATTTTTACCCTAATAATGATAATTCTAGTAACCGCTGAAAATGTATACCAGCTTTTCATTGGCTGAGAGGGCGTAGGTATTATATCCTACATACTCATCAACTGATGATCCTACCGACCAGCCTCCAATAAAGCAGCCCTTCAGGCAGTAATTTACAACCGCCTAGCAGACATCGGCCTAACAGTTGCCCTAGCATGAATAGTTGTAAATAACCTCTCTCTAGATATCCAAGGAGTACAAGCCTCACCAGACCTCGCCATTATTCCCGCGTTCGGGTTCATCCTAGCAGCTGCTGGAAAATCTGCCCAATTCGGCTTCCACCCATGACTGCCAGCAGCAATAGAAGGTCCAACCCCTGTATCCGCCCTACTACACTCAAGTACTATAGTAGTAGCGGGCGTCTTTCTGCTAATTCGAGCCTCAAGCTTTATCCACTGTAATAAAATAACAACCATTGCCTGCCTATTACTAGGAGCACTCACATCCCTGCTCGCAGCAACCTGCGCATTAACTCAAAATGATATAAAAAAAATTATTGCCTACTCTACTACCAGCCAATTAGGACTAATAATAACAGCAATCGGACTAAAACAGCCTGAACTTGCATTCATGCACATCGCAACACATGCCTTCTTTAAAGCAATACTATTTCTATGCGCTGGCGCAATCATCCACAACCTCAATAATGAACAAGATATCCGAAAAATAGGAGGCCTAAAAAAAGCAATACCAATTACTTCTGCCTGCTTAGTTATCGGCGCCCTATCCTTATCAGGAATGCCATTCATGTCTGGATTTTACTCAAAAGACGCCATCATTGAAGCACTTAACACCTCCAACATCAACTCTATCTCACTGGCTATAACCCTAATTGCCACCACCTTCACAGTACTCTACAACTTACGCATAATTTACTATGTGGTATTAGGCACCCCCCGACTACTACCACTTGCTTCACTCCCAGAAACCCGCCAAATCATTAGCCCTATCCTACGATTAGCCGTAGGAAGTATTGCAGCAGGACTCTTAATTTCAACCAACATCCTCCCTTGCAACATCCCACAACTAACCATGCCACCAGAAGTTAAGCTCGCCGCCATAATTATTACAGCCCTAGCACTATTAACTGGTGTAGCCCTAATTCTCACAGCCCCACGACTCCCACTATCCAACAAGGGCACCCAATCTCCCCTCCCTTTCAAAATAGCCAACTTCTACTTCATCCTTCACCACATCCTACCAGCTGCCATCCTATGAATGAGCCAAAAGCTATCAGGTCATCTAACCGATCAAACACACTATGAAGCTCTAGGACCCAAGATATTAACTGTTCTCCAAATCCTGATAACCAAAATTATGACAAATCTTCACAAAGCCCGAATTAACCCATACCTAAAAGTTATTATTTTAGGTATTACATTATTCCTACTGTTATATTACCCCTCAATGACCGAAGGGCCCCCCGCCGATGACCCCGGATAA---------ATGGGAATGACTTTTTTTGCTCTTAGTAGTTTAATATTAGTAGGTGTGGTATTAGTATCAGCGGGGGCCACGACCCATTATGGGGTAATTAGTCTGCTTTTTGCTGCATTATCAAGTGGTGGCCTGCTGGCAGTTGGGGGAGGAAGCTTCATACCAATAGTAGTATTATTAATTTATTTAGGTGGGCTATTGGTAGTATTTACTTTTTGTGTAGGATTTACTGATGATAAATATTGTGAGTTTTGAGGGGTTGGGGCATCTAAGGGGTTGGTTGGTGTTTGTGGGGCTGGGTTGATGGTGGTAGGTTATCGTGCGTATAAGCACATATGG----ATAGGTGTTTTAGGGGGGTTTT--CGGATGCCGTAGAGAATTGGAGTGGTGACATCAGTGATGAGTTGCTAGGGGCCAGCTTGTTCTATCTGGAGGGCTGAGGGTTTGTTGTATTAAGCGGCTGGGCCCTGTTAATTGTGCTATTTACAATTATAAGTATTATCCGGGGTCATCGGCGGGGGGCCCTTCGGTCATTGAGG

*Tomistoma schlegelii 3*

ATGACTATGGCCACAAATCTAAACTTATTTGATCAGTTCTTAATTCCTAAACTTCTTGGAGTACCCATACTAATTCCAGCCTTGTTAATAGCATCTATTTTATTGTACAACCCAGAAGACCGATGATTATCTAACCCAACAACAACCATCCAATACTGGTTAATCACAAAAGTGACCAAACAAATTATAACCCCAGTAAATCAGCCTGGACATAAATGATCATTAATATTAATTTCCCTCCTGACATTTCTTCTTATTAATAATCTTCTAGGCCTACTCCCATACACCTTTACACCAACGACACAGTTATCTATAAACCTAGCCTTAGCCCTACCTCTATGGATAGCAACAGTGTTAACTGGTCTACGAAATAAACCAAGCGCCTCCTTAGCCCATCTACTCCCAGAAGGAACTCCAACACCACTAATTCCAATCTTAATTTTGATCGAATCCATCAGCCTGCTAATCCGACCAATCGCTCTTGCCGTACGACTAACAGCCAATCTCACTGCCGGACACCTTTTAATTCACCTAATTGCCGCTACAGCATTAAACCTATTAACAACATCCCCACTACTAGCCGTACTGACCCTAATTATTCTAGTACTACTAATACTACTAGAAATTGCAGTAGCAATAATTCAAGCCTATGTTTTTACCCTGCTTCTATCCCTCTATCTACAAGAAAATGTATAAATGCCTCAACTAAACCCCAAACCTTGACTAATAGTTTTCCTTACCACCTGATTCATACTAATTATTGTTACACACCCCAAAATCGCCTCCTTAAAGCTTCTAAATAGCCCAGTACCTTTTCATCCAACCCCTATT-AAACCATGACTATGGCCACAAATCTAA---------------------ATGGCCCACCAACTACGAAAGTCCCACCCCCTACTAAAACTCATAAACCACTCTCTAATTGACCTCCCCACTCCATCCAACATCTCCTACTGATGAAACTTTGGATCACTTCTAGGGTTTACCCTTTTAATCCAAATCCTATCAGGCATTCTACTAATAATACATTTCCTAGCACATGACACCATAGCCTTCGCATCCGTCGCTTACACATCACGAGAAGTCTGATACGGCTGACTTATCCGAAGCCTTCACGCAAACGGAGCTTCCCTATTCTTCTTTTGCATCTTCTTACACATTGGACGTGGCCTCTACTACGGCTCTTATCTGCACGAAAACACATGGAACATCGGAGTCATTTTACTCCTCCTGCTAATAGCAACAGCTTTCATAGGTTACGTACTACCCTGAGGACAAATATCATTCTGAGGGGCAACCGTAATCACAAACCTCATATCCGCCATCCCATACATTGGAGACTCACTCGTAATTTGAATCTGAGGGGGCCCTTCAATTAACAGTGCCACCTTAACACGCTTTACCACCCTACACTTTCTACTACCATTTATCCTGCTAGCAACCATCATCACCCACCTCATATTTCTACACGAGCAAGGCTCTTTTAACCCCTTAGGCCTTTCTCCCAACGCCGACAAAGTCCCATTCCACCCATACTTCTCCATAAAAGATGCCCTAGGCACAATACTTGCCATCACCCTACTCTCAACCCTAGCCCTATATTTCCCAAACCTCTTGGGTGACCCAGAAAACTTTACTCCAGCTGACCCTATAAAAACCCCAAACCACATCAAACCCGAATGATACTTCCTATTTGCCTACACGATCCTCCGATCTATTCCTAATAAACTAATAGGGGTATTAGCCATATTCTCATCAATCCTTATCCTTTTCCTAATACCAGCACTCCATACATCAAAACGACAATCAATAACAATACGCCCGCTCTCCCAGCTCCTATTCTGAACCCTTATCGCCGACTTTTTTGTACTAACCTGAATCGGAGGACAGCCCGTACAAAACCCATACACCTTAATTGGCCAAATAGCCTCTATCACTTACTTTACAATTATCCTCATCCTCATACCAACAGCGGGCAAAATAGAAAACTGAATTATTAAACCCCTACAATACCGATACCACGGACC-TTGTGAACATTAATCGCTGACTTTTCTCCACCAACCACAAAGACATCGGCACCCTTTACTTTATCTTCGGAGCCTGAGCGGGAATAGTTGGAACAGCCCTAAGCCTCCTTATTCGCACAGAACTAAGTCAACCCGGACCCCTCATAGGAGACGACCAAATCTATAATGTTATTGTTACCGCACATGCCTTTATTATGATTTTCTTCATAGTAATACCTATTATAATTGGGGGATTTGGAAACTGACTACTACCACTAATGATTGGCGCACCCGACATAGCATTCCCACGAATAAATAATATAAGCTTCTGATTACTCCCACCATCATTCACCTTACTGCTTTTCTCCGCTTTCATCGAAACTGGGGCTGGAACCGGATGAACAGTCTATCCGGCCCTAGCAGGAAACCTAGCCCACGCCGGACCCTCCGTAGACTTAACCATTTTCTCCCTTCACCTCGCAGGAGTATCTTCAATCTTAGGGGCAATTAACTTTATTACCACAGCCATTAACATAAAACCCCCAGCAATATCACAATACCAAACACCTCTTTTTGTATGATCTGTATTAATTACAGCTGTGCTTCTCCTACTTTCCCTACCAGTATTAGCTGCAGGAATCACCATACTACTCACCGATCGAAACCTAAATACAACCTTCTTTGACCCCTCAGGCGGCGGAGACCCTATCCTATATCAACATCTCTTCTGATTCTTCGGCCACCCAGAAGTTTATATCCTTATCCTCCCAGGGTTTGGAATAATCTCCCATGTAGTCACCTTCTACTCAGGAAAAAAAGAACCATTCGGGTATATGGGAATAGTGTGAGCCATAATATCAATTGGCTTCCTGGGTTTCATTGTTTGAGCTCATCACATATTTACAGTAGGAATAGACGTTGATACCCGAGCATATTTTACTTCCGCTACAATAGTAATCGCTATCCCCACCGGAGTAAAAGTATTCAGCTGGCTAGCAACAATCTACGGAGGAATTATAAACTGACAAGCCCCCATACTCTGAGCATTAGGCTTTATTTTCTTATTTACAGTAGGAGGACTAACAGGAATCGTCCTAGCCAACTCATCACTAGACATTATTCTCCACGACACTTATTACGTAGTAGCACACTTCCACTACGTACTATCAATAGGGGCAGTATTCGCCATTATAAGTGGATTTACCCACT-GGTTCCCCCTA-TTCACAGGATTTACCCTTCACCCAGCATGAACAAAAG-CCCAATTCGCAATCATATTCACAGGAGTAAATCTAACCTTCTTCCCACAACACTTCTTAGGCCTTTCCGGGATGCCACGACGATATTCAGACTACCCAGACGCATATTCATTCTGAAATATATTATCATCAATTGGGTCGTTAATTTCTATAGTCTCTGTAATTCTCCTCATATTCATTGTATGAGAAGCATTCTCTTCAAAACGTAAAATTGAGACACCAGAAATAACCATCACAAATATTGAGTGGCTCAACAACTGCCCACCATCTCACCACACTTACGAAGAACCAGTATTTGTCCAAGCACGGCCTAAACCAACAAAAACCCCACCTCCTAACAACAAGGA---------CAGGATGGCAAACCCGATACATCTTGGGCTCCAGGATGCAATATCCCCACTAATAGAAGAGCTCCTCTATTTTCATGACCACACACTAATAGTTATTTTCCTAATTAGTATACTCGTACTCTACACCATCTCCGTCCTCCTAATATCAAAACTTTATCACACAAATGCCACAGACGTACAAGAAGTAGAAATAATTTGAACAATCCTACCGGCCATCATCCTAATTACCATCGCCCTCCCATCCCTACGTACCCTATACCTGATAGATGAAACCAGTAACCCCTGTCTAACAATTAAGGTTATTGGACATCAATGATATTGAACTTATGAATATACAGACTTCTCGCAACTAGAGTTTGATTCCTACATAGTACCAGCACAAGACCTTATACCAGGACACTTCCGCCTACTAGAAGTGGATCACCGTATAATTGCTCCAACAAGTGCAACCACCCGGACACTAATCACAGCAGAAGACGTACTACACTCGTGAGCAGTACCATCCCTTGGAATTAAAATAGACGCGGTACCAGGACGCCTAAACCAAACCTCAATTACACTGGCTAGCCCAGGAATTTTTTACGG-TCAGTGTTCTGAAATTTGTGGAGCTAACCACAGCTTTATACCCGTTGTTGTAGAAGCAATCCCAATACAACACTTTCAAAAATGACTAGAAATAAGTGTTTCCTCATATGACACACCAGACACACCCATTCCACATAGTAAACCCCAGCCCTTGGCCAATTATAGGGGCCATAGCCGCCATAATATTAACCACAGGGCTAGTCCTATGGTTTCACTGCAACCTCAAACTACTCCTAGCCATAGGACTAATCTCAACCCTAACAGTTATATATCAGTGATGACGTGATATCGTACGAGAGAGCACCTACTTAGGTCACCACACCCCTCCAGTCCAAAAAGGTCTGCGCTACGGCATAATCTTATTTATTACATCAGAAGTCTTCTTCTTCCTCGGTTTCTTTTGGGCATTTTACCACTCCAGCCTATCCCCAACCCCAGAGCTAGGAGGACAATGACCCCCAACTGGAATTATTCCACTAGATCCATTTGAGGTCCCACTTTTAAACACCGCCGTACTACTGGCCTCAGGAGTTACAGTAACATGAGCACACCACAGCCTAATAGAAGCCAACCGAACACCAACCATCCACGCCCTTATACTCACCATCGTCCTAGGACTGTACTTCACCGCCCTTCAAGCGATAGAATATTATGAAGCCCCATTCACCATCGCAGACAGCAGCTACGGATCCACCTTTTTCGTAGCCACAGGCTTCCACGGCCTACATGTAATTATTGGATCAACATTTCTAATAATTTGCTTATACCGACAAATCATGCATCACTTCACCTCAAATCACCACTTCGGCTTTGAAGCTGCCGCTTGGTACTGACACTTCGTAGACGTAGTATGACTATTCCTCTACATCTCAATCTACTGATGAGGATCCTATCAGCTTCCTTGTAGCTACTCCTTTTGTGCTTTACATTATCCTAATTTTGATCGCAGTCGCATTCCTGACAGCCTTAGAGCGAAAAATTATTGGCTACATGCAACTACGAAAAGGCCCGAATATCGTGGGACCATTAGGCCTTCTACAACCGTTTGCTGATGGCCTCAAACTTATTATTAAAGAATTAACTTTCCCAATTCTTGCCACCCCCACCCTATTCATCATATCCCCCGCAGTAGCTTTAATCTTATCCCTTACAATATGAGCCCCACTTCCTATACCGTTCTCCCTAGCTAACCTAAACCTAGGAATACTGTTTATACTGGCTATATCCAGCCTAGCAGTATACTCATTATTATGGTCCGGCTGAGCATCAAACTCAAAATACGCCCTAATAGGCGCCTTGCGAGCAGTCGCCCAGACCATCTCCTACGAAGTAACCCTAGCTATTATTATCCTTTCAGTCGTTCTCCTCAGCGGCGGATTTTCACTACATACACTAACTATTACCCAAGAACCAACATATCTAGCACTTACCACATGACCTTTATTAATAATATGATATACATCAACGCTTGCAGAAACAAACCGAGCCCCATTTGACTTAACAGAAGGAGAATCAGAACTGGTTTCAGGATTTAATGTTGAATACAGCGCAGGACTCTTCACACTTTTTTTCCTTGCCGAATATGCTAATATTTTATTAATAAACATTTTAACCACAATCCTATTCCTCAATACATCAACCAACCTCCCAGTACAAACACTTTTTACTATAACCCTAATAAGTAAGTCTATCCTACTAACTATAGGATTCCTATGAATTCGAGCATCATACCCTCGATTCCGCTACGACCAACTAATACACCTTCTATGAAAAAGCTTCCTGCCAGCTACCCTAACATTCTGTTTATGACACATATCATTCCCAGTGTCAATATTTGGCCTCCCGGCGATAA---GGATGCCACTATTCCACCCCATCATTTTAACCACGCTAACTATCACCACATTTATTTTCCTCTCCTCCACACACCTTGTGCTAATATGAGTAGCACTAGAACTGAGCACACTGGCAATCCTACCCTTAATCGCCACTAAATCACACCCACGAGCTATCGAGGCATCCACAAAATACTTCCTCACCCAGGCGACCGCTTCCGCATTAATTATTTTCTCAGGAACATTAAACTATACCATGACGGGCAACTGCCAAATTACTGAATTAACTAACCCAAACTTGATAATAACCTTAATATTAGCCGTATTTATCAAAATTGGACTAGTCCCCTTCCACTTTTGAGTCCCTGAAACACTTCAAGGTATAACTCCAACTGCCGCCATCTTCCTTCTAACCTGACAAAAGCTTGGCCCACTAATTATATTATTCTTAATGAGCCCACTAATTAACTTCGAAGTCCTATCCATAATAGCTGTTCTATCCGCCGTAGTCGCCGGCTGACTTGGACTAAACCAAACTCAAGTCCGAAAACTAGTAGCCATATCCTCCATCGCCCAAATGGCTTGAACCCTCGTAATCGTTAAATACGCACCATCACTTATAATCCTAGCTTTCTACTTATATTCTCTCACCATCTCCGCCACACTCCTCACCCTAGATAAGTTATCAATAACATCTATTAGCAACCTCCTCCTTTCATTCTCAAAAGCCCCCATTATTACATTTTTATTAATAATCTCCCTACTGTCCTTATCAGGCCTACCACCTTTAGCCGGCTTCCTGCCAAAATGACTAACAATTGATATACTCGTTGCAGAAGAAGCCATTTGAGTAGCCTTTATAATACTTATAGCCTCGCTCCTAAGCCTATTCTTCTACATACGACTATGATATAACTCCGCATCCACCATCCCACCCAACACTACTAATACCTCCCGGCTATGACGAAAACCCCTCCCCAAAACAAACCTCACAATCAACTTTCTAAGCATGACCGCCTTTATTTTCATACTAGTAGCCACCCTAATGAAGGCCCTCACAAAATAA------------------A----TAAACCTGCTCACCATATTTATACTAGCTATAGCCACTACAGTAGCTGTAGCGGCAGCGAACCTATTAATAGCTGAAATAACCCCAGACCCTGAAAAACTATCACCGTATGAATGTGGATTTGACCCCTTAGGTTCAGCCCGACTGCCCTTCTCCATTCGATTTTTTATAATCGCTATCTTATTCCTACTATTTGATCTAGAAATCGCTATTCTTTTACCCCTCGCATGAGCCCTTCAACTCACAAATCTTATTAAGGCAACTTCATGAGCCATCATTATTTTTTTATTTATATTTATTGGCCTGACATACGAATGGTTACAAGGTGGTCTAGAATGAGCAGAATAGATGCTAAAACTTATAGTACCAACAATAATAGTTATTCCTTCTACCTGTTTTACACACCCAAAAACAGTCTGACTATTACCAACAGCTTATTCTACAGCCATCATCATTTTAACTTTACTTATCCTAAATCCAGGAGACAGCCTCACAAGCACTAATGGTCTCCTCCTAGGAAGTGATCAAGTCTCTACACCTCTACTAATCCTGTCCTGCTGGCTACTCCCACTTATATTTATGGCTAGCCAAAACTCCATATCCCAGGACCCGTATCCCCAAAAACGACTGTTTATTACCACTTTAGCCCTCCTCCAACTAGCTTTACTACTAGTATTTATAGCCTTAGATCTAATATTATTTTACATTGCCTTCGAAGCGACCCTTATTCCAACGCTAATGGTAATTGCTCGCTGAGGCACCCAAACAGAACGGCTAGGAGCCGGATTATATTTTTTATTTTATACTATTACTAGCTCAATACCACTTTTAATAGCCCTTCTATGAGTATATAATATAAAAGGGACCCTATCTATTCTACTTCTACAACTGACACCACCAATAACCCACATATTCTGGACAGACATGATATTATGGCTATCTCTGCTGCTCGCCTTCCTAGTAAAAATTCCAATTTATGGTCTACACCTATGACTTCCCAAAGCACACGTAGAAGCTCCAATCGCCGGATCCATAGTCCTAGCTGCAGTACTCCTAAAACTAGGTGGCTATGGACTATTACGAGTTACAAACCTATTAACAGAACAAACCAACTCCTCCTATGTTTTACCACTAGCAGTAGCACTATGGGGATCACTCATAACTGGTATAATCTGCTTACGACAAACAGACTTAAAATCCCTAATCGCCTACTCATCAGTTAGTCACATAGGTCTTATAACAAGCTCAATCCTAACCCGTAACCAACTAGCCCTATCTGGTTCAATAATTATAATAATCGCCCACGGCCTAACATCCTCTATACTATTTTGCTTAGCCAATGTTACCTACGAGCGAACACACTCACGAACGCTCCTGCTAGCCCAAGGAGTGCAACTCACCACCCCAGCCATAACTTCCTGATGACTCCTAGCCTGCCTGACAAATATAGCACTCCCTCCAACAATTAATTTTATTGGAGAACTCACTCTTACAGTATCCTTATTTAGCTGATCAGATATTACTATTTTACTAACAGGACTAAGTATATTCATTACTTCAGTCTACACCCTCCATATATTCTCGTCCACTCAACAAGGAACACTCCCCTCGCACATTATTACAATAACCCCCACCCAAACACGCGAACACCTCCTAATACTTATACACTCTGCCCCGTCTCTTACTCTGATTATAGCCCCACAACTAATATGCTCTTAA------ACTGCCCCCACCGACCTCTTATTTATTTGTTCTTTTATTATTTTTACCATTGGACTCACCTTCCATCACACCCACTTACTCTCAGCCTTACTATGCCTAGAAGGCATAATATTATCAGTATTTATACTATTAACAATCTGATCCATCAATTTTAATACCTCTTCCTTTATTTTACCCCTAACAGTATTAACCCTCTCAGCCTGTGAAGCAGGCATTGGCCTTGCCCTACTAATTGCCTCAGCTCGCACACACAGTACAACTAACCTTAAAAATCTTAATCTCCTCCAATGCTAAGTGAACCCAGCTATCA-------------TGTTACTTATGTTGCCACTGACTATTATATTATCATCGCTATTTATCCCTTATT-CAAAAGCTAACAAA--TCATCCCACGCTAAACTGTTGGCAACAAAATTAGCGTTCTTCTCCAGCTTACTCCCCCTAGCCTTCTTTATCTATGATGGACTAGTCGTCACCTCATTTGAGGCCCAATGATTGGCCTTCGACGTATGCCCCGTCCATGTCAGCTTCACATTGGACACATACTCAGTCTTCTTCCTACCAATCTCACTCTTCATCGCTTGATCTATTATAGAATTTACCGTTAAATATATAAAATCAGACCCAAAAATTGACACCTTTTTCCGCTACCTTATTATTTTTACCCTAATAATGATAATTCTAGTAACCGCTGAAAATGTATACCAGCTTTTCATTGGCTGAGAGGGCGTAGGTATTATATCCTACATACTCATCAACTGATGATCCTACCGACCAGCCTCCAATAAAGCAGCCCTTCAGGCAGTAATTTACAACCGCCTAGCAGACATCGGCCTAACAGTTGCCCTAGCATGAATAGTTGTAAATAACCTCTCTCTAGATATCCAAGGAGTACAAGCCTCACCAGACCTCGCCATTATTCCCGCGTTCGGGTTCATCCTAGCAGCTGCTGGAAAATCTGCCCAATTCGGCTTCCACCCATGACTGCCAGCAGCAATAGAAGGTCCAACCCCTGTATCCGCCCTACTACACTCAAGTACTATAGTAGTAGCGGGCGTCTTTCTGCTAATTCGAGCCTCAAGCTTTATCCACTGTAATAAAATAACAACCATTGCCTGCCTATTACTAGGAGCACTCACATCCCTGCTCGCAGCAACCTGCGCATTAACTCAAAATGATATAAAAAAAATTATTGCCTACTCTACTACCAGCCAATTAGGACTAATAATAACAGCAATCGGACTAAAACAGCCTGAACTTGCATTCATGCACATCGCAACACATGCCTTCTTTAAAGCAATACTATTTCTATGCGCTGGCGCAATCATCCACAACCTCAATAATGAACAAGATATCCGAAAAATAGGAGGCCTAAAAAAAGCAATACCAATTACTTCTGCCTGCTTAGTTATCGGCGCCCTATCCTTATCAGGAATGCCATTCATGTCTGGATTTTACTCAAAAGACGCCATCATTGAAGCACTTAACACCTCCAACATCAACTCTATCTCACTGGCTATAACCCTAATTGCCACCACCTTCACAGTACTCTACAACTTACGCATAATTTACTATGTGGTATTAGGCACCCCCCGACTACTACCACTTGCTTCACTCCCAGAAACCCGCCAAATCATTAGCCCTATCCTACGATTAGCCGTAGGAAGTATTGCAGCAGGACTCTTAATTTCAACCAACATCCTCCCTTGCAACATCCCACAACTAACCATGCCACCAGAAGTTAAGCTCGCCGCCATAATTATTACAGCCCTAGCACTATTAACTGGTGTAGCCCTAATTCTCACAGCCCCACGACTCCCACTATCCAACAAGGGCACCCAATCTCCCCTCCCTTTCAAAATAGCCAACTTCTACTTCATCCTTCACCACATCCTACCAGCTGCCATCCTATGAATGAGCCAAAAGCTATCAGGTCATCTAACCGATCAAACACACTATGAAGCTCTAGGACCCAAGATATTAACTGTTCTCCAAATCCTGATAACCAAAATTATGACAAATCTTCACAAAGCCCGAATTAACCCATACCTAAAAGTTATTATTTTAGGTATTACATTATTCCTACTGTTATATTACCCCTCAATGACCGAAGGGCCCCCCGCCGATGACCCCGGATAA---------ATGGGAATGACTTTTTTTGCTCTTAGTAGTTTAATATTAGTAGGTGTGGTATTAGTATCAGCGGGGGCCACGACCCATTATGGGGTAATTAGTCTGCTTTTTGCTGCATTATCAAGTGGTGGCCTGCTGGCAGTTGGGGGAGGAAGCTTCATACCAATAGTAGTATTATTAATTTATTTAGGTGGGCTATTGGTAGTATTTACTTTTTGTGTAGGATTTACTGATGATAAATATTGTGAGTTTTGAGGGGTTGGGGCATCTAAGGGGTTGGTTGGTGTTTGTGGGGCTGGGTTGATGGTGGTAGGTTATCGTGCGTATAAGCACATATGG----ATAGGTGTTTTAGGGGGGTTTT--CGGATGCCGTAGAGAATTGGAGTGGTGACATCAGTGATGAGTTGCTAGGGGCCAGCTTGTTCTATCTGGAGGGCTGAGGGTTTGTTGTATTAAGCGGCTGGGCCCTGTTAATTGTGCTATTTACAATTATAAGTATTATCCGGGGTCATCGGCGGGGGGCCCTTCGGTCATTGAGG

*Alligator mississippiensis*

------------------ATAAACCTATTTGACCAATTCCTAACCCCAAGCCTCCTAGGCATCTCCTTGCTTATACCCGCCCTACTAATAACCACTATCCTACTTCTAAACCCAAAAAATCAATGATTGTCGCACCCCACAACAACAATCAAATCCTGATTTATTAACCAAGCCGCCAAACAAATTATGACCCCAATTAACCCCACCGGGCACAAACACTCCTTAATCCTCATCTCCTTACTAATTCTCCTCTCTCTCACTAACCTGCTTGGCCTGCTTCCATATACCTTCACCCCTACAACACAACTATCCATAAACATAGCCATCGCCCTCCCCCTCTGACTAGTGACAGTATTAATTGGGTTGCGAACTCAACCAACAACCTCCCTAGCCCACCTCCTACCAGAAGGGACTCCTATGCTCCTAATCCCAATCCTAATTTTAATCGAAACAATTAGCCTGCTAATTCGACCAATTGCCCTGGGCGTCCGACTAACGGCTAACCTAACTGCAGGCCACCTGCTAATTCAACTAATCTCAATCGCCACATTAAACCTCTGATTCATGATACCCCCACTCAGCCTATTAACCTCAACAGTCTTAATCCTCCTATTACTACTAGAATTCGCTGTAGCTATAATCCAAGCATACGTCTTCGTCCTCTTATTATCCCTATATTTACAAGAAAATTCATAAATGCCCCAACTAAACCCAGAGCCTTGACTAACAACCCTTCTAATCACATGAATTTCCTTCATCGCCTTCCTTCAACCCAAGATTACCTCCCCCGCACCTGTAAACGACCCAACTACCCGCAAACCCCCAACCATT-AAAACATGACCCTGACCGTGAACACAAACCTGTTTGATCAATTCCTAAATGACCCACCAACTACGAAAATCCCACCCAATCATTAAACTCATCAACCGCTCCCTAATTGACCTACCAACACCCTCAAACATCTCCGCTTGATGAAACTTTGGATCACTACTAGGCCTAACCCTATTAATTCAGATTCTAACAGGATTCTTCTTAATAATGCACTTTTCATCAAGCGATACTCTAGCATTTTCATCTGTATCCTACACCTCCCGCGAAGTCTGATTTGGATGACTCATCCGCAACCTCCACACAAATGGGGCCTCCCTGTTCTTTATATTTATCTTTCTTCACATCGGACGAGGCCTATACTACACATCATATCTTCACGAAAGCACATGAAATATTGGAGTAATCATACTTCTACTCCTAATAGCCACAGCATTTATAGGCTATGTTCTCCCATGAGGACAAATATCATTCTGAGGAGCAACCGTAATCACGAATCTACTTTCTGCCACACCCTACGTTGGAAGCACTGTCGTGCCATGAATTTGAGGCGGCCCCTCTGTTGACAACGCAACACTTACACGCTTCACTGCCCTACACTTCCTCCTTCCATTCGCCCTATTGGCTTCACTCATCACCCACCTGATCTTCCTCCATGAACGAGGATCATTTAACCCCCTAGGAATTAGCCCAAATGCTGACAAAATCCCATTCCACCCCTACTTCACCATAAAAGACGCCCTAGGAGCAGCACTAGCTGCCTCCTCACTACTCATCTTAGCTCTCTACCTACCAGCCCTATTAGGGGACCCTGAAAACTTCACCCCAGCAAATTCCATAATTACCCCAACACACATCAAACCCGAATGGTACTTCCTATTTGCTTATGCCATTCTACGATCTATTCCAAATAAGTTAGGAGGAGTACTAGCAATATTCTCATCCATTTTAGTCCTATTCCTAATACCCGCCCTACACACAGCAAAACAACAACCAATATCAATACGCCCTATATCTCAGCTTCTATTTTGAGCCCTTACCCTGGACTTCCTCTTACTCACATGAATCGGAGGCCAACCAGTAAACCCCCCATATATTTTAATTGGCCAAACTGCCTCCCTATTCTACTTCATCATCATCCTAATCCTCATACCAATAGCAGGCCTCTTAGAGAACAAAATAGTTGAACCCACCTATGTTACCCCTAAG-------TGTGAACTTCCACCGTTGACTCTTCTCTACTAACCACAAAGACATTGGCACCCTTTACTTCATTTTCGGAACTTGAGCCGGAATGGTGGGAACAGCACTTAGCCTCCTTATTCGGACAGAATTAAGCCAGCCCGGACCTCTATTAGGTGATGACCAAATTTATAACGTAATTGTCACCGCCCATGCCTTCATTATAATCTTTTTTATAGTAATACCAATTATAATTGGAGGGTTTGGAAACTGGCTATTACCCCTAATAATCGGAGCCCCAGACATGGCATTCCCCCGAATAAACAACATAAGTTTCTGATTACTCCCCCCATCTTTCACACTACTACTCTCCTCAGCCTGCATCGAAGCAGGTGCTGGAACAGGGTGAACCGTCTACCCTCCCCTAGCCGGAAACCTAGCCCACGCCGGGCCATCCGTAGATTTAACTATCTTCTCTCTACACTTAGCCGGAGTATCTTCCATCCTCGGAGCAATTAACTTTATTACAACAGCAATCAACATAAAACCCCCAGCAATATCCCAATACCAAACACCACTATTTGTGTGATCCGTCCTAATTACAGCTGTACTTCTCCTACTATCCCTACCAGTACTAGCTGCTGGAATTACAATACTACTCACAGACCGCAACTTAAACACAACCTTCTTTGACCCCGCAGGGGGAGGAGATCCCATCCTATACCAACACCTCTTCTGATTCTTTGGCCACCCAGAAGTATATATCCTAATTCTTCCAGGGTTCGGAATAATTTCCCACGTAGTAGCCTTTTATTCAGGCAAAAAAGAACCATTCGGCTATATAGGAATAGCATGAGCCATATTATCCATTGGATTCTTAGGGTTCATCGTCTGAGCCCACCACATATTTACAGTCGGAATAGACGTAGACACCCGAGCATACTTTACCACCGCCACAATAATCATTGCCGTTCCCACCGGGGTAAAAGTATTTAGCTGACTAGCCACCATCTATGGCGGCATTGTTAACTGACAAGCCCCAATACTCTGAGCACTAGGCTTCATCTTCTTATTTACCGTCGGGGGCCTAACTGGGATTGTCCTAGCCAACTCCTCACTAGACATTGTCCTCCACGACACTTATTATGTAGTGGCCCACTTCCACTACGTACTCTCAATAGGAGCAGTCTTCGCTATCATGAGCGGATTCACCCACT-GATTCCCACTC-TTTACAGGATTTACCCTTCACCCAACATGAACTAAAA-TCCAATTTGTAATTATATTTACCGGAGTAAATTTTACCTTCTTCCCACAACACTTCCTAGGACTATCTGGGATACCTCGACGATACTCGGACTACCCAGACGCATACACCCTCTGAAACCTAACATCATCAATTGGATCCTTAATTTCCATGGTTGCAGTTATCCTTCTAATATTTATTATCTGAGAAGCATTCACATCAAAACGAAAAGTGACAGCACTCGAAATAACAATAACCAACATTGAGTGACTTAACAACTGCCCCCCATCTCATCACACCTACGAAGAGCCCGTATTCGCTGTAGTACA-----------------------------------------------------TAGCCAACCCGACACACCTAGGATTCCAAGATGCAATATCTCCCTTAATAGAAGAGCTACTATATTTTCACGACCACACATTAATAATCCTCTTCCTTATTAGCTCCCTCGTATTTTACATAATCTTCGCCCTATTATTCCCTAAACTATACTACCCAAACACCTCAGACGTTCAAGAAGTAGAAGTAATCTGAACCGTCCTACCAGCCATTGTCCTCATCTCAATTGCCCTACCATCACTACGCACCCTATACCTCATAGACGAAACCAATAACCCCTGCCTGACTATTAAAGTAACCGGACACCAATGATACTGATCTTACGAATACACCGACTTCTCAACACTCGAATTTGACTCCTACATAATTCCCACACAAGATCTTCCCCAAGGACACTTCCGCCTACTAGAAGTTGACCACCGCATAATCACCCCAACTAACTCAACCATCCGAGTACTAATTACAGCAGAAGATGTACTACACTCATGGGCAATCCCATCCATTGGGACAAAAATGGACGCAGTCCCAGGACGCTTAAACCAAGTTATAATTACACTTGCCAATCCTGGAGTATTCTACGG-CCAATGCTCTGAGATCTGCGGGGCAAACCACAGCTTCATACCTATCACCATAGAAACCATCCCACTAAACCACTTCCAACTCTGATTAGAAGATTCTATTCTCTCCTATGTCACACCAAACACACCTCTTCCATATAGTCAACCCAAGTCCCTGACCCCTCGCCGGAGCTATAGCCGCCATACTACTAACAACAGGCCTAACTCTTTGATTTCATTACAACTCCAACCTACTGCTACTATTAGGTCTAGTTACCACCATACTAGTAATATATCAATGATGACGAGACGTTGTCCGAGAAAGCACCTACCTGGGACACCACACACCCGCAGTACAAAAAGGATTACGCTATGGTATAATCCTCTTTATTACATCAGAAGTTTTCTTCTTCCTAGGCTTTTTCTGAGCATTCTACCACTCAAGCTTATCTCCTACCCCTGAATTAGGAGGACAATGACCCCCAACCGGGATTACCCCCCTTGACCCATTTGAAGTCCCACTCCTCAACACAGCTGTCCTACTCGCCTCCGGAGTAACAGTAACATGAGCCCACCATAATTTAATAACCGCCAACCGAATACAAGCAATTCATGCCCTAACACTCACCGTACTCCTGGGCTTTTACTTCACCGCCCTCCAAGCCATAGAATACTATGAAGCCCCCTTTACTATTGCAGACAGCACCTACGGATCAACATTCTTCGTTGCAACTGGCTTCCACGGCCTCCACGTCATCATTGGCTCAACATTCCTCATGATCTGCCTTTATCGACAAGTAAAATTCCACTTCACATCTAACCACCATTTCGGATTCGAAGCTGCTGCCTGATACTGACACTTTGTAGACGTCGTCTGACTTTTCCTCTATATCTCGATCTACTGATGAGGATCGTATAGACTCCCTCACTATTGTACCCCCAGCATTACTAATTATCTCCATCTTAATAGCAGTTGCATTTTTAACAGCGCTAGAACGAAAAATTATAGGCCACATACAACTACGAAAAGGACCAAACATTGTTGGCCCCCTTGGCCTACTTCAACCATTTGCCGATGGACTTAAACTTATTACCAAAGAGTTAACCCTTCCCCTGCTTGCCACCCCTACCCTTTTCATCCTGGCCCCAACAGCTGCCTTAATACTTGCTCTCGCCATGTGGTCCCCCCTCCCCATACCATCTCCGCTCGCAGACCTAAACCTTGGATTATTACTCTTACTCGCAATATCAAGCCTTATGGTTTATTCGTTCCTATGATCAGGATGATCATCAAATTCTAAATATGCCCTAATGGGCGCCATACGGGCAGTTGCTCAAACCATCTCCTATGAAGTAACACTAGCCATTATTGTCCTATCTATTGTCCTACTGAGTGGAGGATTCTCACTTCACACCCTTACTGTCACACAAGAACCCCTATACCTTGCGCTAGCTACATGGCCCTCAATAATAATATGGTATACTTCCACACTAGCAGAAACAAACCGCGCCCCCTTTGACTTAACAGAAGGAGAATCGGAACTAGTATCCGGATTTAATGTAGAATACAGTGCAAGCCCTTTCGCACTTTTTTTCCTAGCTGAATATGCCAACATTATATTAATAAATACACTCACCACCACCCTGTTTCTCAGCCCATCAACCCCCACCTTCCTCCCAGCACTATTCACCATTGCCCTAATAAGCAAGGCTCTCCTACTAACCATAAGTTTCTTATGGGTTCGAGCATCTTACCCCCGATTTCGTTACGACCAGCTTATGCACCTCCTATGAAAAAACTTCCTACCCATAACACTAACCCTCTGCCTATGACACTCATCAGTGCCAATATCAATGTTTGGACTACCACCAATGACTTAGATGCCCCTCTCCCAACCAATTATCCTAGCAACACTAACCATTACAACACTAATTTTTCTACTATCAACCCATCTGGTCCTAATCTGAGTTGCACTAGAACTTAACACACTAGCGATCCTCCCCCTAATTGCTCACAAATCTCACCCACGAGCCATCGAAGCCTCCACAAAATATTTTCTCACCCAGGCAATAGCATCTGCATTAATTATCTTTTCAGGAACACTAAATTACGAAATGACAGGAAGCTGCCAAATTGTAGAATTAACAAACTTAACTTCAATAATTGTGCTAACCCTTGCCCTATTTATTAAAGTGGGATTAGTACCATTTCACTTCTGAGTACCAGAAGTTCTCCAAGGAATATCCACAACTGCCGCAATCTTCCTATTAACCTGACAAAAACTAGGACCATTAATTATACTATTCCTAATTAGCCCCCTTATCAACTTTGAATTAACCTCTGTAGTAGCTACTTTATCTTCCCTTGTTGCAGGCTGAATGGGACTAAACCAAACTCAAGTACGAAAATTAATAGCACTATCGTCCATTGCCCAAATAGCATGAATTATCGTAATTATTAAATACGCACCATCACTAGCCATCCTAACCTTCTACATCTACTCCACCACCATCTCCGCTACACTGTTAACACTAGACAAAATATCAACAACCTCCATTAAATACCTCATTATTTCTTTTTCAAAATCCCCAATCACCACCACCATCCTGATAATTTCCCTCCTATCACTATCCGGCCTCCCACCCCTAGCCGGCTTTATACCAAAATGATTAACAATCAACCAACTCCTCGCAGAAAAAGCAATTTGAATTGCACTATTAATACTAATTACATCCCTTCTAAGTCTATTCTTCTATCTCCGACTATGATACAACTCCTCATCAACTATGCCACCAAGCACTACCAACACAACCCGCCTCTGACGAAAATCTACCCCCCAAAGTAACTTTACCATCAACCTCCTCACCATAGCGACCACCACTCTCCTACTATCAACCACACTAATGAAAGCAATTACTAAACAAGAATACTCTCTAGGCTAAA----TTAACCTATTTATCATACTCACAATATCCTCAATCACCGTTTCAATCGTAGTCGCCCTAAACCTACTGACTGCCAAAACATCACCCGACCCAGAAAAATTATCCCCTTACGAATGTGGATTCGACCCCCTCGGCTCTGCTCGCTTACCCCTATCAATCCGGTTCTTTATAGTAGGCATCCTATTCCTGCTTTTTGACCTTGAAATTGCCATCCTACTACCACTCACATGAGCCATCCACACCCTCAACCCCTTAAAAACTATTACATGAGCCATCATCATCTTCCTATTCCTATTCATTGGACTAGCATACGAATGACTCCAGGGTGGCCTAGAATGAGCAGAATAAATGCTAAAACTTTTATTAGCCACATTCATGTTAATCCCTACGACCCCCCTGCTCCCAAATAAAATTACTTGACTAACTCCAACAACCTACTCAATTATCGTAACAGCCCTGGCCCTACTGGTCCTCAACCCCTCAGACATCTTTATAAACACCAGCGGCCCAGCCCTAGGCAGCGACCAACTCTCAACCCCCCTAATAATTCTATCCTGCTGACTTCTTCCCTTAATACTTATAGCCAGCCAAAACTCCATACTTAAAACCCCTGCCCCCCAAAACCACACATTTATTACAATTCTTGCAATACTTCAATTCACCCTACTCATAACCTTTATAGCCCTAGACCTCATATTATTTTACATTTCCTTTGAAGCCACCTTAATCCCCACCCTCATCATTATCTCACGGTGAGGAGCCCAAGCAGATCGCCTAAACGCAGGCATTTACTTCCTGTTTTATACCATTACTAGCTCAATTCCACTAATAATTGGCATTCTAATAATTTACAACCTAAAAGGCACCCTATCCATCCTCACCCTACAACTAACCCCAATAACAGGCCTCATCTCCTGAACAGACACCCTACTATGACTATCAATTCTACTAGCTTTCCTAGTAAAAATCCCCCTCTATGGTTTACATCTATGACTCCCCAAGGCTCATGTAGAAGCTCCCATTGCAGGCTCTATAGTCCTCGCTGCAGTGCTTCTTAAACTAGGCGGTTATGGCCTACTACGAGTAGTAATTTTACTCACTGAGCAAATCAACACAATCTACCCCCCAATTTTAGGCCTAGCGCTCTGGGGGGCACTTATGACAAGCCTCATCTGCTTGCGACAAACGGACCTAAAATCCCTAATCGCTTACTCATCGGTTAGCCATATGGCCTTAGTAACAGCTGCAATTCTCACCCGAAACCAACTAACCCCGATAGGGTCAATAATCCTAATGATTGCCCATGGACTAACATCTTCTATATTATTCTGTTTAGCAAACTTCAGCTATGAACGCACCCACACCCGCACACTTCTCGCAATACAAGGAATACAACTTACCACACCTACTTTAACATCATGGTGATTTCTAGCTAGCATAATAAATATAGCCCTGCCCCCAACAATTAACTTTGTAGGTGAACTAGCTCTCATTACCTCACTTTTTAACTGATCAGAGACGACACTACTATTAACAGGGCTCAACTCTACCATTACTGCAATCTACACGCTCTACATATTCTCGTCAACCCAACAAGGAACACTACCAATCCACACCCTCATCCTCTCACCCACCCAAACCCGCGAACACCTCTTAATACTATTACACACACTACCATCAATTATCCTCATCACAAACCCACGACTTATTTACCATGACTA---AATCTCCCCGACGGGCACCACGTTCATATTCTCCTTCATCATCTGTACCATCGGCCTAATTATATACCATACACACCTGCTTTCGACACTACTTTGCCTTGAGGGAATAATACTGTCAATTTTTATAGCCCTAACAATATTATCGCTCGACTTGCACACCTCCTCATTCATCCTACCATTAACAGTCCTAACCCTATCCGCTTGTGAAGCAGGAGTTGGCCTTGCCTTATTAGTCGCCTCTGCTCGAACTCATAATACGGCAAGCCTCAAAAACCTAAACCTGCTACAATGCTAAAT----GCAACAGCCGACCCTATCTCTTACCTTCTTCACCCTACCTGCTTTTATCCTTTTACTATCT---------CTACTTCATATAACCGCCCAC---CCGGTCGGTTTTAAAAAAATGATAATTAAACTAGCATTTTTCACAAGCCTCCCTCCCCTTATTTTATTAACTTACAACAACATAGTTCCCCTCTCATTCACTTGACATTGACTCAGCATAGGAACCTGCCCCATCTACTTAAGCCTTAAACTCGACACCTTCTCAATCTTCTTCGTCCCCACAGCACTGTTTGTCACATGATCAATTATAGAATTCACCGAATTATATATAGATTCAGACCTAAAAATTATTAGCTTCTTTAATCACCTCTTAATCTTTATCCTAATAATAATTATCTTAGTAACTGCCAACAACCTCTTCCAGCTATTCATTGGCTGAGAAGGAGTAGGCATCATATCATTTAAACTCATCAACTGATGGTCTTTCCGAGCAAACTCCAACAAAGCAGCCCTCCAGGCCATTACCTACAATCGTCTAGCAGACGTCGGAATACTCGCTGCTATATCATGAATGATCCTAAACAACATCAGTCTGGACATCCAAGACATACCCGTATCTACCAACCACTCACTCATCCCTGCCATTGGCCTCGTCCTAGCAGCAACTGGAAAATCGGCCCAATTCGGTTTCCACCCATGACTCCCGGCAGCAATAGAAGGCCCGACACCAGTCTCAGCTCTACTCCACTCAAGCACCATAGTAGTAGCAGGCATTTTCCTGCTAATCCGAACCTCCCACGTAGTCTATAGCAGTCAAGCAGCAACTACGGCTTGCCTACTCCTGGGAGCAATTACCTCCCTATTCGCAGCCTCTTGCGCCCTCACCCAAAATGACATAAAAAAAATTATCGCATTCTCAACATCGAGCCAACTCGGACTAATAATAGCCACAATTGGATTAAAACAACCCGAACTTGCATTCCTACACATTTCAACACACGCCTTTTTTAAAGCAATACTCTTTCTGTGTGCAGGATCAATTATCCACAACCTCAATAACGAACAAGACATTCGAAAAATAGGGGGCCTTAAAAAAGCAATACCTATCACCACCTCTTGTCTTACTATCGGAGCACTAGCTCTCACTGGCATGCCCTTCCTCTCAGGATTCTTTTCCAAAGACGCTATTATTGAAGCACTAAACACCTCTTATATTAACGCCTGTGCCCTTACCCTTGTACTACTTGCTACTTCCTTCACCGCAGTGTATAGCCTTCGTATAATTTACTTCACCCTATTAAACCCTAACCGACTAACAACCACAAATCCCATCAACGAAAACCCAAAAATTGTAAACCCCATCCTACGCCTAGCCGCCGGAAGCATTATAGCTGGCCTACTAATTTCATCCTCTATATTACCATCCAACACCCCCCAACTAACCATATCGAACACAGCCAAGCTCGCAGCCCTTATTATCACAATGATCGGACTACTAATTGCAGCAACCCTAACCCATGCCACCAATAAATTCCCGCCCATTACCAACGACACCCAACCGCCCTTCCTAACTAAACTAACCTACTTCAACCACCTATTCCACCACTTCTTCCCAACCACAACCCTGCGAATGAGCCAAAAACTATCAACCCACCTAGCTGACCAAACATGATATGAAGCCCTCGGGCCAAAAATAATAACCCACCTACAAACCCTAATAGCCAAAATCCTCACACCATATCATAAAGGAAAAATAAAACAATATTTCAAAATCTTCATATTAACTATTATTATAATTATCTTTTTCA---CCTCCTTTATATCACCCTAA---------------------------------ATGGGATTTACGTT---GTTTCTTTGTTGTTTGATATTAGCATGTGCGATGATGGTGGCGGCAAGTTCAATAATTCATTTTGCCGTGGTAAGTTTACTTTTTGTAGTTGTATTCGGTAGTGGTTTGTTAATTATTGAGGGTGGAAGTTTTATGCCTTTAGTGGTGTTATTAGTTTATTTAGGGGGGTTATTGGTGGTGTTTGCTTTTTGTGTTGGGTTTACTGATGATCCATTTGGGGGGTTTTGAGGAGTGGGGGTGTCTAAGTGGGTCGTATTTTTTTGATGGGCGGGGTTCTTTCGTGGGGTTTATTCACGATATTGGCGTTTGTGGTGTTGGGGAAAGACTAGTGCGGGGGAGTTTTGGGGCG---AGGTAGTTAG------------TAGTGAGTTGTTAGGGGTTAGTCAGATGTATTCTGATGGGTGGGCGTTCATTGTGGTGTGCGGGTGAGCTTTACTGGTGGTGTTATTCGTAATTACAGGGTTTGTTCGAGGGTATCGTCAGGGGGCGTTTCGCTCTTTTAGG

*Alligator mississippiensis 2*

------------------ATAAACCTATTTGACCAATTCCTAACCCCAAGCCTCCTAGGCATCTCCTTGCTTATACCCGCCCTACTAATAACCACTATCCTACTTCTAAACCCAAAAAATCAATGATTGTCGCACCCCACAACAACAATCAAATCCTGATTTATTAACCAAGCCGCCAAACAAATTATGACCCCAATTAACCCCACCGGGCACAAACACTCCTTAATCCTCATCTCCTTACTAATTCTCCTCTCTCTCACTAACCTGCTTGGCCTGCTTCCATATACCTTCACCCCTACAACACAACTATCCATAAACATAGCCATCGCCCTCCCCCTCTGACTAGTGACAGTATTAATTGGGTTGCGAACTCAACCAACAACCTCCCTAGCCCACCTCCTACCAGAAGGGACTCCTATGCTCCTAATCCCAATCCTAATTTTAATCGAAACAATTAGCCTGCTAATTCGACCAATTGCCCTGGGCGTCCGACTAACGGCTAACCTAACTGCAGGCCACCTGCTAATTCAACTAATCTCAATCGCCACATTAAACCTCTGATTCATGATACCCCCACTCAGCCTATTAACCTCAACAGTCTTAATCCTCCTATTACTACTAGAATTCGCTGTAGCTATAATCCAAGCATACGTCTTCGTCCTCTTATTATCCCTATATTTACAAGAAAATTCATAAATGCCCCAACTAAACCCAGAGCCTTGACTAACAACCCTTCTAATCACATGAATTTCCTTCATCGCCTTCCTTCAACCCAAGATTACCTCCCCCGCACCTGTAAACGACCCAACTACCCGCAAACCCCCAACCATT-AAAACATGACCCTGACCGTGAACACAAACCTGTTTGATCAATTCCTAAATGACCCACCAACTACGAAAATCCCACCCAATCATTAAACTCATCAACCGCTCCCTAATTGACCTACCAACACCCTCAAACATCTCCGCTTGATGAAACTTTGGATCACTACTAGGCCTAACCCTATTAATTCAGATTCTAACAGGATTCTTCTTAATAATGCACTTTTCATCAAGCGATACTCTAGCATTTTCATCTGTATCCTACACCTCCCGCGAAGTCTGATTTGGATGACTCATCCGCAACCTCCACACAAATGGGGCCTCCCTGTTCTTTATATTTATCTTTCTTCACATCGGACGAGGCCTATACTACACATCATATCTTCACGAAAGCACATGAAATATTGGAGTAATCATACTTCTACTCCTAATAGCCACAGCATTTATAGGCTATGTTCTCCCATGAGGACAAATATCATTCTGAGGAGCAACCGTAATCACGAATCTACTTTCTGCCACACCCTACGTTGGAAGCACTGTCGTGCCATGAATTTGAGGCGGCCCCTCTGTTGACAACGCAACACTTACACGCTTCACTGCCCTACACTTCCTCCTTCCATTCGCCCTATTGGCTTCACTCATCACCCACCTGATCTTCCTCCATGAACGAGGATCATTTAACCCCCTAGGAATTAGCCCAAATGCTGACAAAATCCCATTCCACCCCTACTTCACCATAAAAGACGCCCTAGGAGCAGCACTAGCTGCCTCCTCACTACTCATCTTAGCTCTCTACCTACCAGCCCTATTAGGGGACCCTGAAAACTTCACCCCAGCAAATTCCATAATTACCCCAACACACATCAAACCCGAATGGTACTTCCTATTTGCTTATGCCATTCTACGATCTATTCCAAATAAGTTAGGAGGAGTACTAGCAATATTCTCATCCATTTTAGTCCTATTCCTAATACCCGCCCTACACACAGCAAAACAACAACCAATATCAATACGCCCTATATCTCAGCTTCTATTTTGAGCCCTTACCCTGGACTTCCTCTTACTCACATGAATCGGAGGCCAACCAGTAAACCCCCCATATATTTTAATTGGCCAAACTGCCTCCCTATTCTACTTCATCATCATCCTAATCCTCATACCAATAGCAGGCCTCTTAGAGAACAAAATAGTTGAACCCACCTATGTTACCCCTAAG-------TGTGAACTTCCACCGTTGACTCTTCTCTACTAACCACAAAGACATTGGCACCCTTTACTTCATTTTCGGAACTTGAGCCGGAATGGTGGGAACAGCACTTAGCCTCCTTATTCGGACAGAATTAAGCCAGCCCGGACCTCTATTAGGTGATGACCAAATTTATAACGTAATTGTCACCGCCCATGCCTTCATTATAATCTTTTTTATAGTAATACCAATTATAATTGGAGGGTTTGGAAACTGGCTATTACCCCTAATAATCGGAGCCCCAGACATGGCATTCCCCCGAATAAACAACATAAGTTTCTGATTACTCCCCCCATCTTTCACACTACTACTCTCCTCAGCCTGCATCGAAGCAGGTGCTGGAACAGGGTGAACCGTCTACCCTCCCCTAGCCGGAAACCTAGCCCACGCCGGGCCATCCGTAGATTTAACTATCTTCTCTCTACACTTAGCCGGAGTATCTTCCATCCTCGGAGCAATTAACTTTATTACAACAGCAATCAACATAAAACCCCCAGCAATATCCCAATACCAAACACCACTATTTGTGTGATCCGTCCTAATTACAGCTGTACTTCTCCTACTATCCCTACCAGTACTAGCTGCTGGAATTACAATACTACTCACAGACCGCAACTTAAACACAACCTTCTTTGACCCCGCAGGGGGAGGAGATCCCATCCTATACCAACACCTCTTCTGATTCTTTGGCCACCCAGAAGTATATATCCTAATTCTTCCAGGGTTCGGAATAATTTCCCACGTAGTAGCCTTTTATTCAGGCAAAAAAGAACCATTCGGCTATATAGGAATAGCATGAGCCATATTATCCATTGGATTCTTAGGGTTCATCGTCTGAGCCCACCACATATTTACAGTCGGAATAGACGTAGACACCCGAGCATACTTTACCACCGCCACAATAATCATTGCCGTTCCCACCGGGGTAAAAGTATTTAGCTGACTAGCCACCATCTATGGCGGCATTGTTAACTGACAAGCCCCAATACTCTGAGCACTAGGCTTCATCTTCTTATTTACCGTCGGGGGCCTAACTGGGATTGTCCTAGCCAACTCCTCACTAGACATTGTCCTCCACGACACTTATTATGTAGTGGCCCACTTCCACTACGTACTCTCAATAGGAGCAGTCTTCGCTATCATGAGCGGATTCACCCACT-GATTCCCACTC-TTTACAGGATTTACCCTTCACCCAACATGAACTAAAA-TCCAATTTGTAATTATATTTACCGGAGTAAATTTTACCTTCTTCCCACAACACTTCCTAGGACTATCTGGGATACCTCGACGATACTCGGACTACCCAGACGCATACACCCTCTGAAACCTAACATCATCAATTGGATCCTTAATTTCCATGGTTGCAGTTATCCTTCTAATATTTATTATCTGAGAAGCATTCACATCAAAACGAAAAGTGACAGCACTCGAAATAACAATAACCAACATTGAGTGACTTAACAACTGCCCCCCATCTCATCACACCTACGAAGAGCCCGTATTCGCTGTAGTACA-----------------------------------------------------TAGCCAACCCGACACACCTAGGATTCCAAGATGCAATATCTCCCTTAATAGAAGAGCTACTATATTTTCACGACCACACATTAATAATCCTCTTCCTTATTAGCTCCCTCGTATTTTACATAATCTTCGCCCTATTATTCCCTAAACTATACTACCCAAACACCTCAGACGTTCAAGAAGTAGAAGTAATCTGAACCGTCCTACCAGCCATTGTCCTCATCTCAATTGCCCTACCATCACTACGCACCCTATACCTCATAGACGAAACCAATAACCCCTGCCTGACTATTAAAGTAACCGGACACCAATGATACTGATCTTACGAATACACCGACTTCTCAACACTCGAATTTGACTCCTACATAATTCCCACACAAGATCTTCCCCAAGGACACTTCCGCCTACTAGAAGTTGACCACCGCATAATCACCCCAACTAACTCAACCATCCGAGTACTAATTACAGCAGAAGATGTACTACACTCATGGGCAATCCCATCCATTGGGACAAAAATGGACGCAGTCCCAGGACGCTTAAACCAAGTTATAATTACACTTGCCAATCCTGGAGTATTCTACGG-CCAATGCTCTGAGATCTGCGGGGCAAACCACAGCTTCATACCTATCACCATAGAAACCATCCCACTAAACCACTTCCAACTCTGATTAGAAGATTCTATTCTCTCCTATGTCACACCAAACACACCTCTTCCATATAGTCAACCCAAGTCCCTGACCCCTCGCCGGAGCTATAGCCGCCATACTACTAACAACAGGCCTAACTCTTTGATTTCATTACAACTCCAACCTACTGCTACTATTAGGTCTAGTTACCACCATACTAGTAATATATCAATGATGACGAGACGTTGTCCGAGAAAGCACCTACCTGGGACACCACACACCCGCAGTACAAAAAGGATTACGCTATGGTATAATCCTCTTTATTACATCAGAAGTTTTCTTCTTCCTAGGCTTTTTCTGAGCATTCTACCACTCAAGCTTATCTCCTACCCCTGAATTAGGAGGACAATGACCCCCAACCGGGATTACCCCCCTTGACCCATTTGAAGTCCCACTCCTCAACACAGCTGTCCTACTCGCCTCCGGAGTAACAGTAACATGAGCCCACCATAATTTAATAACCGCCAACCGAATACAAGCAATTCATGCCCTAACACTCACCGTACTCCTGGGCTTTTACTTCACCGCCCTCCAAGCCATAGAATACTATGAAGCCCCCTTTACTATTGCAGACAGCACCTACGGATCAACATTCTTCGTTGCAACTGGCTTCCACGGCCTCCACGTCATCATTGGCTCAACATTCCTCATGATCTGCCTTTATCGACAAGTAAAATTCCACTTCACATCTAACCACCATTTCGGATTCGAAGCTGCTGCCTGATACTGACACTTTGTAGACGTCGTCTGACTTTTCCTCTATATCTCGATCTACTGATGAGGATCGTATAGACTCCCTCACTATTGTACCCCCAGCATTACTAATTATCTCCATCTTAATAGCAGTTGCATTTTTAACAGCGCTAGAACGAAAAATTATAGGCCACATACAACTACGAAAAGGACCAAACATTGTTGGCCCCCTTGGCCTACTTCAACCATTTGCCGATGGACTTAAACTTATTACCAAAGAGTTAACCCTTCCCCTGCTTGCCACCCCTACCCTTTTCATCCTGGCCCCAACAGCTGCCTTAATACTTGCTCTCGCCATGTGGTCCCCCCTCCCCATACCATCTCCGCTCGCAGACCTAAACCTTGGATTATTACTCTTACTCGCAATATCAAGCCTTATGGTTTATTCGTTCCTATGATCAGGATGATCATCAAATTCTAAATATGCCCTAATGGGCGCCATACGGGCAGTTGCTCAAACCATCTCCTATGAAGTAACACTAGCCATTATTGTCCTATCTATTGTCCTACTGAGTGGAGGATTCTCACTTCACACCCTTACTGTCACACAAGAACCCCTATACCTTGCGCTAGCTACATGGCCCTCAATAATAATATGGTATACTTCCACACTAGCAGAAACAAACCGCGCCCCCTTTGACTTAACAGAAGGAGAATCGGAACTAGTATCCGGATTTAATGTAGAATACAGTGCAAGCCCTTTCGCACTTTTTTTCCTAGCTGAATATGCCAACATTATATTAATAAATACACTCACCACCACCCTGTTTCTCAGCCCATCAACCCCCACCTTCCTCCCAGCACTATTCACCATTGCCCTAATAAGCAAGGCTCTCCTACTAACCATAAGTTTCTTATGGGTTCGAGCATCTTACCCCCGATTTCGTTACGACCAGCTTATGCACCTCCTATGAAAAAACTTCCTACCCATAACACTAACCCTCTGCCTATGACACTCATCAGTGCCAATATCAATGTTTGGACTACCACCAATGACTTAGATGCCCCTCTCCCAACCAATTATCCTAGCAACACTAACCATTACAACACTAATTTTTCTACTATCAACCCATCTGGTCCTAATCTGAGTTGCACTAGAACTTAACACACTAGCGATCCTCCCCCTAATTGCTCACAAATCTCACCCACGAGCCATCGAAGCCTCCACAAAATATTTTCTCACCCAGGCAATAGCATCTGCATTAATTATCTTTTCAGGAACACTAAATTACGAAATGACAGGAAGCTGCCAAATTGTAGAATTAACAAACTTAACTTCAATAATTGTGCTAACCCTTGCCCTATTTATTAAAGTGGGATTAGTACCATTTCACTTCTGAGTACCAGAAGTTCTCCAAGGAATATCCACAACTGCCGCAATCTTCCTATTAACCTGACAAAAACTAGGACCATTAATTATACTATTCCTAATTAGCCCCCTTATCAACTTTGAATTAACCTCTGTAGTAGCTACTTTATCTTCCCTTGTTGCAGGCTGAATGGGACTAAACCAAACTCAAGTACGAAAATTAATAGCACTATCGTCCATTGCCCAAATAGCATGAATTATCGTAATTATTAAATACGCACCATCACTAGCCATCCTAACCTTCTACATCTACTCCACCACCATCTCCGCTACACTGTTAACACTAGACAAAATATCAACAACCTCCATTAAATACCTCATTATTTCTTTTTCAAAATCCCCAATCACCACCACCATCCTGATAATTTCCCTCCTATCACTATCCGGCCTCCCACCCCTAGCCGGCTTTATACCAAAATGATTAACAATCAACCAACTCCTCGCAGAAAAAGCAATTTGAATTGCACTATTAATACTAATTACATCCCTTCTAAGTCTATTCTTCTATCTCCGACTATGATACAACTCCTCATCAACTATGCCACCAAGCACTACCAACACAACCCGCCTCTGACGAAAATCTACCCCCCAAAGTAACTTTACCATCAACCTCCTCACCATAGCGACCACCACTCTCCTACTATCAACCACACTAATGAAAGCAATTACTAAACAAGAATACTCTCTAGGCTAAA----TTAACCTATTTATCATACTCACAATATCCTCAATCACCGTTTCAATCGTAGTCGCCCTAAACCTACTGACTGCCAAAACATCACCCGACCCAGAAAAATTATCCCCTTACGAATGTGGATTCGACCCCCTCGGCTCTGCTCGCTTACCCCTATCAATCCGGTTCTTTATAGTAGGCATCCTATTCCTGCTTTTTGACCTTGAAATTGCCATCCTACTACCACTCACATGAGCCATCCACACCCTCAACCCCTTAAAAACTATTACATGAGCCATCATCATCTTCCTATTCCTATTCATTGGACTAGCATACGAATGACTCCAGGGTGGCCTAGAATGAGCAGAATAAATGCTAAAACTTTTATTAGCCACATTCATGTTAATCCCTACGACCCCCCTGCTCCCAAATAAAATTACTTGACTAACTCCAACAACCTACTCAATTATCGTAACAGCCCTGGCCCTACTGGTCCTCAACCCCTCAGACATCTTTATAAACACCAGCGGCCCAGCCCTAGGCAGCGACCAACTCTCAACCCCCCTAATAATTCTATCCTGCTGACTTCTTCCCTTAATACTTATAGCCAGCCAAAACTCCATACTTAAAACCCCTGCCCCCCAAAACCACACATTTATTACAATTCTTGCAATACTTCAATTCACCCTACTCATAACCTTTATAGCCCTAGACCTCATATTATTTTACATTTCCTTTGAAGCCACCTTAATCCCCACCCTCATCATTATCTCACGGTGAGGAGCCCAAGCAGATCGCCTAAACGCAGGCATTTACTTCCTGTTTTATACCATTACTAGCTCAATTCCACTAATAATTGGCATTCTAATAATTTACAACCTAAAAGGCACCCTATCCATCCTCACCCTACAACTAACCCCAATAACAGGCCTCATCTCCTGAACAGACACCCTACTATGACTATCAATTCTACTAGCTTTCCTAGTAAAAATCCCCCTCTATGGTTTACATCTATGACTCCCCAAGGCTCATGTAGAAGCTCCCATTGCAGGCTCTATAGTCCTCGCTGCAGTGCTTCTTAAACTAGGCGGTTATGGCCTACTACGAGTAGTAATTTTACTCACTGAGCAAATCAACACAATCTACCCCCCAATTTTAGGCCTAGCGCTCTGGGGGGCACTTATGACAAGCCTCATCTGCTTGCGACAAACGGACCTAAAATCCCTAATCGCTTACTCATCGGTTAGCCATATGGCCTTAGTAACAGCTGCAATTCTCACCCGAAACCAACTAACCCCGATAGGGTCAATAATCCTAATGATTGCCCATGGACTAACATCTTCTATATTATTCTGTTTAGCAAACTTCAGCTATGAACGCACCCACACCCGCACACTTCTCGCAATACAAGGAATACAACTTACCACACCTACTTTAACATCATGGTGATTTCTAGCTAGCATAATAAATATAGCCCTGCCCCCAACAATTAACTTTGTAGGTGAACTAGCTCTCATTACCTCACTTTTTAACTGATCAGAGACGACACTACTATTAACAGGGCTCAACTCTACCATTACTGCAATCTACACGCTCTACATATTCTCGTCAACCCAACAAGGAACACTACCAATCCACACCCTCATCCTCTCACCCACCCAAACCCGCGAACACCTCTTAATACTATTACACACACTACCATCAATTATCCTCATCACAAACCCACGACTTATTTACCATGACTA---AATCTCCCCGACGGGCACCACGTTCATATTCTCCTTCATCATCTGTACCATCGGCCTAATTATATACCATACACACCTGCTTTCGACACTACTTTGCCTTGAGGGAATAATACTGTCAATTTTTATAGCCCTAACAATATTATCGCTCGACTTGCACACCTCCTCATTCATCCTACCATTAACAGTCCTAACCCTATCCGCTTGTGAAGCAGGAGTTGGCCTTGCCTTATTAGTCGCCTCTGCTCGAACTCATAATACGGCAAGCCTCAAAAACCTAAACCTGCTACAATGCTAAAT----GCAACAGCCGACCCTATCTCTTACCTTCTTCACCCTACCTGCTTTTATCCTTTTACTATCT---------CTACTTCATATAACCGCCCAC---CCGGTCGGTTTTAAAAAAATGATAATTAAACTAGCATTTTTCACAAGCCTCCCTCCCCTTATTTTATTAACTTACAACAACATAGTTCCCCTCTCATTCACTTGACATTGACTCAGCATAGGAACCTGCCCCATCTACTTAAGCCTTAAACTCGACACCTTCTCAATCTTCTTCGTCCCCACAGCACTGTTTGTCACATGATCAATTATAGAATTCACCGAATTATATATAGATTCAGACCTAAAAATTATTAGCTTCTTTAATCACCTCTTAATCTTTATCCTAATAATAATTATCTTAGTAACTGCCAACAACCTCTTCCAGCTATTCATTGGCTGAGAAGGAGTAGGCATCATATCATTTAAACTCATCAACTGATGGTCTTTCCGAGCAAACTCCAACAAAGCAGCCCTCCAGGCCATTACCTACAATCGTCTAGCAGACGTCGGAATACTCGCTGCTATATCATGAATGATCCTAAACAACATCAGTCTGGACATCCAAGACATACCCGTATCTACCAACCACTCACTCATCCCTGCCATTGGCCTCGTCCTAGCAGCAACTGGAAAATCGGCCCAATTCGGTTTCCACCCATGACTCCCGGCAGCAATAGAAGGCCCGACACCAGTCTCAGCTCTACTCCACTCAAGCACCATAGTAGTAGCAGGCATTTTCCTGCTAATCCGAACCTCCCACGTAGTCTATAGCAGTCAAGCAGCAACTACGGCTTGCCTACTCCTGGGAGCAATTACCTCCCTATTCGCAGCCTCTTGCGCCCTCACCCAAAATGACATAAAAAAAATTATCGCATTCTCAACATCGAGCCAACTCGGACTAATAATAGCCACAATTGGATTAAAACAACCCGAACTTGCATTCCTACACATTTCAACACACGCCTTTTTTAAAGCAATACTCTTTCTGTGTGCAGGATCAATTATCCACAACCTCAATAACGAACAAGACATTCGAAAAATAGGGGGCCTTAAAAAAGCAATACCTATCACCACCTCTTGTCTTACTATCGGAGCACTAGCTCTCACTGGCATGCCCTTCCTCTCAGGATTCTTTTCCAAAGACGCTATTATTGAAGCACTAAACACCTCTTATATTAACGCCTGTGCCCTTACCCTTGTACTACTTGCTACTTCCTTCACCGCAGTGTATAGCCTTCGTATAATTTACTTCACCCTATTAAACCCTAACCGACTAACAACCACAAATCCCATCAACGAAAACCCAAAAATTGTAAACCCCATCCTACGCCTAGCCGCCGGAAGCATTATAGCTGGCCTACTAATTTCATCCTCTATATTACCATCCAACACCCCCCAACTAACCATATCGAACACAGCCAAGCTCGCAGCCCTTATTATCACAATGATCGGACTACTAATTGCAGCAACCCTAACCCATGCCACCAATAAATTCCCGCCCATTACCAACGACACCCAACCGCCCTTCCTAACTAAACTAACCTACTTCAACCACCTATTCCACCACTTCTTCCCAACCACAACCCTGCGAATGAGCCAAAAACTATCAACCCACCTAGCTGACCAAACATGATATGAAGCCCTCGGGCCAAAAATAATAACCCACCTACAAACCCTAATAGCCAAAATCCTCACACCATATCATAAAGGAAAAATAAAACAATATTTCAAAATCTTCATATTAACTATTATTATAATTATCTTTTTCA---CCTCCTTTATATCACCCTAA---------------------------------ATGGGATTTACGTT---GTTTCTTTGTTGTTTGATATTAGCATGTGCGATGATGGTGGCGGCAAGTTCAATAATTCATTTTGCCGTGGTAAGTTTACTTTTTGTAGTTGTATTCGGTAGTGGTTTGTTAATTATTGAGGGTGGAAGTTTTATGCCTTTAGTGGTGTTATTAGTTTATTTAGGGGGGTTATTGGTGGTGTTTGCTTTTTGTGTTGGGTTTACTGATGATCCATTTGGGGGGTTTTGAGGAGTGGGGGTGTCTAAGTGGGTCGTATTTTTTTGATGGGCGGGGTTCTTTCGTGGGGTTTATTCACGATATTGGCGTTTGTGGTGTTGGGGAAAGACTAGTGCGGGGGAGTTTTGGGGCG---AGGTAGTTAG------------TAGTGAGTTGTTAGGGGTTAGTCAGATGTATTCTGATGGGTGGGCGTTCATTGTGGTGTGCGGGTGAGCTTTACTGGTGGTGTTATTCGTAATTACAGGGTTTGTTCGAGGGTATCGTCAGGGGGCGTTTCGCTCTTTTAGG

*Alligator mississippiensis 3*

------------------ATAAACCTATTTGACCAATTCCTAACCCCAAGCCTCCTAGGCATCTCCTTGCTTATACCCGCCCTACTAATAACCACTATCCTACTTCTAAACCCAAAAAATCAATGATTGTCGCACCCCACAACAACAATCAAATCCTGATTTATTAACCAAGCCGCCAAACAAATTATGACCCCAATTAACCCCACCGGGCACAAACACTCCTTAATCCTCATCTCCTTACTAATTCTCCTCTCTCTCACTAACCTGCTTGGCCTGCTTCCATATACCTTCACCCCTACAACACAACTATCCATAAACATAGCCATCGCCCTCCCCCTCTGACTAGTGACAGTATTAATTGGGTTGCGAACTCAACCAACAACCTCCCTAGCCCACCTCCTACCAGAAGGGACTCCTATGCTCCTAATCCCAATCCTAATTTTAATCGAAACAATTAGCCTGCTAATTCGACCAATTGCCCTGGGCGTCCGACTAACGGCTAACCTAACTGCAGGCCACCTGCTAATTCAACTAATCTCAATCGCCACATTAAACCTCTGATTCATGATACCCCCACTCAGCCTATTAACCTCAACAGTCTTAATCCTCCTATTACTACTAGAATTCGCTGTAGCTATAATCCAAGCATACGTCTTCGTCCTCTTATTATCCCTATATTTACAAGAAAATTCATAAATGCCCCAACTAAACCCAGAGCCTTGACTAACAACCCTTCTAATCACATGAATTTCCTTCATCGCCTTCCTTCAACCCAAGATTACCTCCCCCGCACCTGTAAACGACCCAACTACCCGCAAACCCCCAACCATT-AAAACATGACCCTGACCGTGAACACAAACCTGTTTGATCAATTCCTAAATGACCCACCAACTACGAAAATCCCACCCAATCATTAAACTCATCAACCGCTCCCTAATTGACCTACCAACACCCTCAAACATCTCCGCTTGATGAAACTTTGGATCACTACTAGGCCTAACCCTATTAATTCAGATTCTAACAGGATTCTTCTTAATAATGCACTTTTCATCAAGCGATACTCTAGCATTTTCATCTGTATCCTACACCTCCCGCGAAGTCTGATTTGGATGACTCATCCGCAACCTCCACACAAATGGGGCCTCCCTGTTCTTTATATTTATCTTTCTTCACATCGGACGAGGCCTATACTACACATCATATCTTCACGAAAGCACATGAAATATTGGAGTAATCATACTTCTACTCCTAATAGCCACAGCATTTATAGGCTATGTTCTCCCATGAGGACAAATATCATTCTGAGGAGCAACCGTAATCACGAATCTACTTTCTGCCACACCCTACGTTGGAAGCACTGTCGTGCCATGAATTTGAGGCGGCCCCTCTGTTGACAACGCAACACTTACACGCTTCACTGCCCTACACTTCCTCCTTCCATTCGCCCTATTGGCTTCACTCATCACCCACCTGATCTTCCTCCATGAACGAGGATCATTTAACCCCCTAGGAATTAGCCCAAATGCTGACAAAATCCCATTCCACCCCTACTTCACCATAAAAGACGCCCTAGGAGCAGCACTAGCTGCCTCCTCACTACTCATCTTAGCTCTCTACCTACCAGCCCTATTAGGGGACCCTGAAAACTTCACCCCAGCAAATTCCATAATTACCCCAACACACATCAAACCCGAATGGTACTTCCTATTTGCTTATGCCATTCTACGATCTATTCCAAATAAGTTAGGAGGAGTACTAGCAATATTCTCATCCATTTTAGTCCTATTCCTAATACCCGCCCTACACACAGCAAAACAACAACCAATATCAATACGCCCTATATCTCAGCTTCTATTTTGAGCCCTTACCCTGGACTTCCTCTTACTCACATGAATCGGAGGCCAACCAGTAAACCCCCCATATATTTTAATTGGCCAAACTGCCTCCCTATTCTACTTCATCATCATCCTAATCCTCATACCAATAGCAGGCCTCTTAGAGAACAAAATAGTTGAACCCACCTATGTTACCCCTAAG-------TGTGAACTTCCACCGTTGACTCTTCTCTACTAACCACAAAGACATTGGCACCCTTTACTTCATTTTCGGAACTTGAGCCGGAATGGTGGGAACAGCACTTAGCCTCCTTATTCGGACAGAATTAAGCCAGCCCGGACCTCTATTAGGTGATGACCAAATTTATAACGTAATTGTCACCGCCCATGCCTTCATTATAATCTTTTTTATAGTAATACCAATTATAATTGGAGGGTTTGGAAACTGGCTATTACCCCTAATAATCGGAGCCCCAGACATGGCATTCCCCCGAATAAACAACATAAGTTTCTGATTACTCCCCCCATCTTTCACACTACTACTCTCCTCAGCCTGCATCGAAGCAGGTGCTGGAACAGGGTGAACCGTCTACCCTCCCCTAGCCGGAAACCTAGCCCACGCCGGGCCATCCGTAGATTTAACTATCTTCTCTCTACACTTAGCCGGAGTATCTTCCATCCTCGGAGCAATTAACTTTATTACAACAGCAATCAACATAAAACCCCCAGCAATATCCCAATACCAAACACCACTATTTGTGTGATCCGTCCTAATTACAGCTGTACTTCTCCTACTATCCCTACCAGTACTAGCTGCTGGAATTACAATACTACTCACAGACCGCAACTTAAACACAACCTTCTTTGACCCCGCAGGGGGAGGAGATCCCATCCTATACCAACACCTCTTCTGATTCTTTGGCCACCCAGAAGTATATATCCTAATTCTTCCAGGGTTCGGAATAATTTCCCACGTAGTAGCCTTTTATTCAGGCAAAAAAGAACCATTCGGCTATATAGGAATAGCATGAGCCATATTATCCATTGGATTCTTAGGGTTCATCGTCTGAGCCCACCACATATTTACAGTCGGAATAGACGTAGACACCCGAGCATACTTTACCACCGCCACAATAATCATTGCCGTTCCCACCGGGGTAAAAGTATTTAGCTGACTAGCCACCATCTATGGCGGCATTGTTAACTGACAAGCCCCAATACTCTGAGCACTAGGCTTCATCTTCTTATTTACCGTCGGGGGCCTAACTGGGATTGTCCTAGCCAACTCCTCACTAGACATTGTCCTCCACGACACTTATTATGTAGTGGCCCACTTCCACTACGTACTCTCAATAGGAGCAGTCTTCGCTATCATGAGCGGATTCACCCACT-GATTCCCACTC-TTTACAGGATTTACCCTTCACCCAACATGAACTAAAA-TCCAATTTGTAATTATATTTACCGGAGTAAATTTTACCTTCTTCCCACAACACTTCCTAGGACTATCTGGGATACCTCGACGATACTCGGACTACCCAGACGCATACACCCTCTGAAACCTAACATCATCAATTGGATCCTTAATTTCCATGGTTGCAGTTATCCTTCTAATATTTATTATCTGAGAAGCATTCACATCAAAACGAAAAGTGACAGCACTCGAAATAACAATAACCAACATTGAGTGACTTAACAACTGCCCCCCATCTCATCACACCTACGAAGAGCCCGTATTCGCTGTAGTACA-----------------------------------------------------TAGCCAACCCGACACACCTAGGATTCCAAGATGCAATATCTCCCTTAATAGAAGAGCTACTATATTTTCACGACCACACATTAATAATCCTCTTCCTTATTAGCTCCCTCGTATTTTACATAATCTTCGCCCTATTATTCCCTAAACTATACTACCCAAACACCTCAGACGTTCAAGAAGTAGAAGTAATCTGAACCGTCCTACCAGCCATTGTCCTCATCTCAATTGCCCTACCATCACTACGCACCCTATACCTCATAGACGAAACCAATAACCCCTGCCTGACTATTAAAGTAACCGGACACCAATGATACTGATCTTACGAATACACCGACTTCTCAACACTCGAATTTGACTCCTACATAATTCCCACACAAGATCTTCCCCAAGGACACTTCCGCCTACTAGAAGTTGACCACCGCATAATCACCCCAACTAACTCAACCATCCGAGTACTAATTACAGCAGAAGATGTACTACACTCATGGGCAATCCCATCCATTGGGACAAAAATGGACGCAGTCCCAGGACGCTTAAACCAAGTTATAATTACACTTGCCAATCCTGGAGTATTCTACGG-CCAATGCTCTGAGATCTGCGGGGCAAACCACAGCTTCATACCTATCACCATAGAAACCATCCCACTAAACCACTTCCAACTCTGATTAGAAGATTCTATTCTCTCCTATGTCACACCAAACACACCTCTTCCATATAGTCAACCCAAGTCCCTGACCCCTCGCCGGAGCTATAGCCGCCATACTACTAACAACAGGCCTAACTCTTTGATTTCATTACAACTCCAACCTACTGCTACTATTAGGTCTAGTTACCACCATACTAGTAATATATCAATGATGACGAGACGTTGTCCGAGAAAGCACCTACCTGGGACACCACACACCCGCAGTACAAAAAGGATTACGCTATGGTATAATCCTCTTTATTACATCAGAAGTTTTCTTCTTCCTAGGCTTTTTCTGAGCATTCTACCACTCAAGCTTATCTCCTACCCCTGAATTAGGAGGACAATGACCCCCAACCGGGATTACCCCCCTTGACCCATTTGAAGTCCCACTCCTCAACACAGCTGTCCTACTCGCCTCCGGAGTAACAGTAACATGAGCCCACCATAATTTAATAACCGCCAACCGAATACAAGCAATTCATGCCCTAACACTCACCGTACTCCTGGGCTTTTACTTCACCGCCCTCCAAGCCATAGAATACTATGAAGCCCCCTTTACTATTGCAGACAGCACCTACGGATCAACATTCTTCGTTGCAACTGGCTTCCACGGCCTCCACGTCATCATTGGCTCAACATTCCTCATGATCTGCCTTTATCGACAAGTAAAATTCCACTTCACATCTAACCACCATTTCGGATTCGAAGCTGCTGCCTGATACTGACACTTTGTAGACGTCGTCTGACTTTTCCTCTATATCTCGATCTACTGATGAGGATCGTATAGACTCCCTCACTATTGTACCCCCAGCATTACTAATTATCTCCATCTTAATAGCAGTTGCATTTTTAACAGCGCTAGAACGAAAAATTATAGGCCACATACAACTACGAAAAGGACCAAACATTGTTGGCCCCCTTGGCCTACTTCAACCATTTGCCGATGGACTTAAACTTATTACCAAAGAGTTAACCCTTCCCCTGCTTGCCACCCCTACCCTTTTCATCCTGGCCCCAACAGCTGCCTTAATACTTGCTCTCGCCATGTGGTCCCCCCTCCCCATACCATCTCCGCTCGCAGACCTAAACCTTGGATTATTACTCTTACTCGCAATATCAAGCCTTATGGTTTATTCGTTCCTATGATCAGGATGATCATCAAATTCTAAATATGCCCTAATGGGCGCCATACGGGCAGTTGCTCAAACCATCTCCTATGAAGTAACACTAGCCATTATTGTCCTATCTATTGTCCTACTGAGTGGAGGATTCTCACTTCACACCCTTACTGTCACACAAGAACCCCTATACCTTGCGCTAGCTACATGGCCCTCAATAATAATATGGTATACTTCCACACTAGCAGAAACAAACCGCGCCCCCTTTGACTTAACAGAAGGAGAATCGGAACTAGTATCCGGATTTAATGTAGAATACAGTGCAAGCCCTTTCGCACTTTTTTTCCTAGCTGAATATGCCAACATTATATTAATAAATACACTCACCACCACCCTGTTTCTCAGCCCATCAACCCCCACCTTCCTCCCAGCACTATTCACCATTGCCCTAATAAGCAAGGCTCTCCTACTAACCATAAGTTTCTTATGGGTTCGAGCATCTTACCCCCGATTTCGTTACGACCAGCTTATGCACCTCCTATGAAAAAACTTCCTACCCATAACACTAACCCTCTGCCTATGACACTCATCAGTGCCAATATCAATGTTTGGACTACCACCAATGACTTAGATGCCCCTCTCCCAACCAATTATCCTAGCAACACTAACCATTACAACACTAATTTTTCTACTATCAACCCATCTGGTCCTAATCTGAGTTGCACTAGAACTTAACACACTAGCGATCCTCCCCCTAATTGCTCACAAATCTCACCCACGAGCCATCGAAGCCTCCACAAAATATTTTCTCACCCAGGCAATAGCATCTGCATTAATTATCTTTTCAGGAACACTAAATTACGAAATGACAGGAAGCTGCCAAATTGTAGAATTAACAAACTTAACTTCAATAATTGTGCTAACCCTTGCCCTATTTATTAAAGTGGGATTAGTACCATTTCACTTCTGAGTACCAGAAGTTCTCCAAGGAATATCCACAACTGCCGCAATCTTCCTATTAACCTGACAAAAACTAGGACCATTAATTATACTATTCCTAATTAGCCCCCTTATCAACTTTGAATTAACCTCTGTAGTAGCTACTTTATCTTCCCTTGTTGCAGGCTGAATGGGACTAAACCAAACTCAAGTACGAAAATTAATAGCACTATCGTCCATTGCCCAAATAGCATGAATTATCGTAATTATTAAATACGCACCATCACTAGCCATCCTAACCTTCTACATCTACTCCACCACCATCTCCGCTACACTGTTAACACTAGACAAAATATCAACAACCTCCATTAAATACCTCATTATTTCTTTTTCAAAATCCCCAATCACCACCACCATCCTGATAATTTCCCTCCTATCACTATCCGGCCTCCCACCCCTAGCCGGCTTTATACCAAAATGATTAACAATCAACCAACTCCTCGCAGAAAAAGCAATTTGAATTGCACTATTAATACTAATTACATCCCTTCTAAGTCTATTCTTCTATCTCCGACTATGATACAACTCCTCATCAACTATGCCACCAAGCACTACCAACACAACCCGCCTCTGACGAAAATCTACCCCCCAAAGTAACTTTACCATCAACCTCCTCACCATAGCGACCACCACTCTCCTACTATCAACCACACTAATGAAAGCAATTACTAAACAAGAATACTCTCTAGGCTAAA----TTAACCTATTTATCATACTCACAATATCCTCAATCACCGTTTCAATCGTAGTCGCCCTAAACCTACTGACTGCCAAAACATCACCCGACCCAGAAAAATTATCCCCTTACGAATGTGGATTCGACCCCCTCGGCTCTGCTCGCTTACCCCTATCAATCCGGTTCTTTATAGTAGGCATCCTATTCCTGCTTTTTGACCTTGAAATTGCCATCCTACTACCACTCACATGAGCCATCCACACCCTCAACCCCTTAAAAACTATTACATGAGCCATCATCATCTTCCTATTCCTATTCATTGGACTAGCATACGAATGACTCCAGGGTGGCCTAGAATGAGCAGAATAAATGCTAAAACTTTTATTAGCCACATTCATGTTAATCCCTACGACCCCCCTGCTCCCAAATAAAATTACTTGACTAACTCCAACAACCTACTCAATTATCGTAACAGCCCTGGCCCTACTGGTCCTCAACCCCTCAGACATCTTTATAAACACCAGCGGCCCAGCCCTAGGCAGCGACCAACTCTCAACCCCCCTAATAATTCTATCCTGCTGACTTCTTCCCTTAATACTTATAGCCAGCCAAAACTCCATACTTAAAACCCCTGCCCCCCAAAACCACACATTTATTACAATTCTTGCAATACTTCAATTCACCCTACTCATAACCTTTATAGCCCTAGACCTCATATTATTTTACATTTCCTTTGAAGCCACCTTAATCCCCACCCTCATCATTATCTCACGGTGAGGAGCCCAAGCAGATCGCCTAAACGCAGGCATTTACTTCCTGTTTTATACCATTACTAGCTCAATTCCACTAATAATTGGCATTCTAATAATTTACAACCTAAAAGGCACCCTATCCATCCTCACCCTACAACTAACCCCAATAACAGGCCTCATCTCCTGAACAGACACCCTACTATGACTATCAATTCTACTAGCTTTCCTAGTAAAAATCCCCCTCTATGGTTTACATCTATGACTCCCCAAGGCTCATGTAGAAGCTCCCATTGCAGGCTCTATAGTCCTCGCTGCAGTGCTTCTTAAACTAGGCGGTTATGGCCTACTACGAGTAGTAATTTTACTCACTGAGCAAATCAACACAATCTACCCCCCAATTTTAGGCCTAGCGCTCTGGGGGGCACTTATGACAAGCCTCATCTGCTTGCGACAAACGGACCTAAAATCCCTAATCGCTTACTCATCGGTTAGCCATATGGCCTTAGTAACAGCTGCAATTCTCACCCGAAACCAACTAACCCCGATAGGGTCAATAATCCTAATGATTGCCCATGGACTAACATCTTCTATATTATTCTGTTTAGCAAACTTCAGCTATGAACGCACCCACACCCGCACACTTCTCGCAATACAAGGAATACAACTTACCACACCTACTTTAACATCATGGTGATTTCTAGCTAGCATAATAAATATAGCCCTGCCCCCAACAATTAACTTTGTAGGTGAACTAGCTCTCATTACCTCACTTTTTAACTGATCAGAGACGACACTACTATTAACAGGGCTCAACTCTACCATTACTGCAATCTACACGCTCTACATATTCTCGTCAACCCAACAAGGAACACTACCAATCCACACCCTCATCCTCTCACCCACCCAAACCCGCGAACACCTCTTAATACTATTACACACACTACCATCAATTATCCTCATCACAAACCCACGACTTATTTACCATGACTA---AATCTCCCCGACGGGCACCACGTTCATATTCTCCTTCATCATCTGTACCATCGGCCTAATTATATACCATACACACCTGCTTTCGACACTACTTTGCCTTGAGGGAATAATACTGTCAATTTTTATAGCCCTAACAATATTATCGCTCGACTTGCACACCTCCTCATTCATCCTACCATTAACAGTCCTAACCCTATCCGCTTGTGAAGCAGGAGTTGGCCTTGCCTTATTAGTCGCCTCTGCTCGAACTCATAATACGGCAAGCCTCAAAAACCTAAACCTGCTACAATGCTAAAT----GCAACAGCCGACCCTATCTCTTACCTTCTTCACCCTACCTGCTTTTATCCTTTTACTATCT---------CTACTTCATATAACCGCCCAC---CCGGTCGGTTTTAAAAAAATGATAATTAAACTAGCATTTTTCACAAGCCTCCCTCCCCTTATTTTATTAACTTACAACAACATAGTTCCCCTCTCATTCACTTGACATTGACTCAGCATAGGAACCTGCCCCATCTACTTAAGCCTTAAACTCGACACCTTCTCAATCTTCTTCGTCCCCACAGCACTGTTTGTCACATGATCAATTATAGAATTCACCGAATTATATATAGATTCAGACCTAAAAATTATTAGCTTCTTTAATCACCTCTTAATCTTTATCCTAATAATAATTATCTTAGTAACTGCCAACAACCTCTTCCAGCTATTCATTGGCTGAGAAGGAGTAGGCATCATATCATTTAAACTCATCAACTGATGGTCTTTCCGAGCAAACTCCAACAAAGCAGCCCTCCAGGCCATTACCTACAATCGTCTAGCAGACGTCGGAATACTCGCTGCTATATCATGAATGATCCTAAACAACATCAGTCTGGACATCCAAGACATACCCGTATCTACCAACCACTCACTCATCCCTGCCATTGGCCTCGTCCTAGCAGCAACTGGAAAATCGGCCCAATTCGGTTTCCACCCATGACTCCCGGCAGCAATAGAAGGCCCGACACCAGTCTCAGCTCTACTCCACTCAAGCACCATAGTAGTAGCAGGCATTTTCCTGCTAATCCGAACCTCCCACGTAGTCTATAGCAGTCAAGCAGCAACTACGGCTTGCCTACTCCTGGGAGCAATTACCTCCCTATTCGCAGCCTCTTGCGCCCTCACCCAAAATGACATAAAAAAAATTATCGCATTCTCAACATCGAGCCAACTCGGACTAATAATAGCCACAATTGGATTAAAACAACCCGAACTTGCATTCCTACACATTTCAACACACGCCTTTTTTAAAGCAATACTCTTTCTGTGTGCAGGATCAATTATCCACAACCTCAATAACGAACAAGACATTCGAAAAATAGGGGGCCTTAAAAAAGCAATACCTATCACCACCTCTTGTCTTACTATCGGAGCACTAGCTCTCACTGGCATGCCCTTCCTCTCAGGATTCTTTTCCAAAGACGCTATTATTGAAGCACTAAACACCTCTTATATTAACGCCTGTGCCCTTACCCTTGTACTACTTGCTACTTCCTTCACCGCAGTGTATAGCCTTCGTATAATTTACTTCACCCTATTAAACCCTAACCGACTAACAACCACAAATCCCATCAACGAAAACCCAAAAATTGTAAACCCCATCCTACGCCTAGCCGCCGGAAGCATTATAGCTGGCCTACTAATTTCATCCTCTATATTACCATCCAACACCCCCCAACTAACCATATCGAACACAGCCAAGCTCGCAGCCCTTATTATCACAATGATCGGACTACTAATTGCAGCAACCCTAACCCATGCCACCAATAAATTCCCGCCCATTACCAACGACACCCAACCGCCCTTCCTAACTAAACTAACCTACTTCAACCACCTATTCCACCACTTCTTCCCAACCACAACCCTGCGAATGAGCCAAAAACTATCAACCCACCTAGCTGACCAAACATGATATGAAGCCCTCGGGCCAAAAATAATAACCCACCTACAAACCCTAATAGCCAAAATCCTCACACCATATCATAAAGGAAAAATAAAACAATATTTCAAAATCTTCATATTAACTATTATTATAATTATCTTTTTCA---CCTCCTTTATATCACCCTAA---------------------------------ATGGGATTTACGTT---GTTTCTTTGTTGTTTGATATTAGCATGTGCGATGATGGTGGCGGCAAGTTCAATAATTCATTTTGCCGTGGTAAGTTTACTTTTTGTAGTTGTATTCGGTAGTGGTTTGTTAATTATTGAGGGTGGAAGTTTTATGCCTTTAGTGGTGTTATTAGTTTATTTAGGGGGGTTATTGGTGGTGTTTGCTTTTTGTGTTGGGTTTACTGATGATCCATTTGGGGGGTTTTGAGGAGTGGGGGTGTCTAAGTGGGTCGTATTTTTTTGATGGGCGGGGTTCTTTCGTGGGGTTTATTCACGATATTGGCGTTTGTGGTGTTGGGGAAAGACTAGTGCGGGGGAGTTTTGGGGCG---AGGTAGTTAG------------TAGTGAGTTGTTAGGGGTTAGTCAGATGTATTCTGATGGGTGGGCGTTCATTGTGGTGTGCGGGTGAGCTTTACTGGTGGTGTTATTCGTAATTACAGGGTTTGTTCGAGGGTATCGTCAGGGGGCGTTTCGCTCTTTTAGG

*Alligator sinensis*

------------GTGAACACAAACCTGTTTGATCAATTCCTAATCCCAAGCCTCCTAGGCATCTCCCTATTAATGCCGGCCCTACTAATAACTGCCATTCTCCTTTTAAACCCTAAAAATCAATGACTATCACACCCAACAGTAACAATCAAATCTTGATTTATTAATAAAGCTACAAAACAAATCATGCTTCCTATCAGCCCCTCAGGGCGGAAACAATCCTTAATCCTCATCTCCTTATTAATCCTCCTTCTCTTTACTAACCTGCTTGGCCTACTTCCATACACCTTCACCCCAACAACACAACTATCTATAAACATAGCCCTCGGCCTTCCTCTATGGCTGGCAACAGTATTAATCGGGCTTCGGACCCGCCCAACGGCCTCCCTGGGCCACCTTCTTCCAGGAGGGACCCCCACCCTCCTTATCCCGGGCCTAATCTTGATCGAGACAATTAGCCTACTAATTCGACCAATCGCCCTAGGTGTCCGACTAACAGCAAACCTAACTGCGGGCCACCTACTAATTCAATTAATCTCAATCGCCACATTAAACCTCTGATCCATAATACCCCCACTTAGCCTATTGACCTTGACAGTCCTGATTCTCCTCTTATTACTAGAATTTGCTGTGGCCATAATCCAAGCCTACGTCTTCGTCCTCCTATTATCCCTATACCTTCAAGAAAACACGTAAATGCCACAACTAAACCCAGAACCTTGACTAACAACCTTCCTAATCGTTTGAATCTCCCTTATTGTTATTCTCCAACCCAAAATCGCCTCACTCATACTCACAAGCAGCCCAACCCCTTACAAAGCCATAACTATT-AAAACATGACCCTGACCTTGAACA---------------------TAAATGACCCACCAGCTACGAAAATCCCACCCACTTATTAAACTTATTAACCAAACCCTTATTGACCTCCCAACACCCTCAAACATCTCAGCTTGTTGAAACTTTGGATCACTACTAGGCCTAACCCTTCTAATCCAGATCCTAACAGGAGTCTTCTTAATAATGCACTTCTCATCGGGTGACACCATAGCATTTTCATCTGTCGCCTACACCTCCCGTGAAGTTTGGTTCGGGTGGCTTATTCGCGGCCTCCACATAAACGGGGCCTCTCTCTTCTTCATATTCATCTTCCTCCACATCGGACGAGGCCTATACTACGCATCCTACCTTCACGAGAGCACGTGAAATGTCGGAGTAATTATACTCCTACTCCTGATAGCCACTGCATTCATAGGCTACGTCCTCCCGTGAGGACAAATATCGTTCTGGGGAGCAACCGTAATTACAAATCTACTATCCGCCACACCCTACGTTGGAAGCACTGTTGTACCCTGAATCTGAGGCGGCCCCTCTGTAGACAACGCAACACTCATACGCTTCACCGCCCTACACTTCATTCTCCCTTTTGCCCTATTAGCCTCACTAGTTACCCACCTAATCTTCCTACACGAACGAGGATCCTTCAACCCCCTAGGAGTCAACTCGAATACTGACAAAATCCCATTCCACCCCTACTATACCCTAAAAGACACCCTTGGAGCAGCACTAGCCGCCTCAGCACTACTCACCCTCGCCCTCTATTTACCAACCTTATTAAGCGACCCTGAAAACTTTACCCAAGCAAACTCCATAATTACCCCCACACACATTAAACCAGAATGGTACTTCTTATTCGCCTACGCTATTCTACGATCCACCCCTAACAAACTAGGAGGAGTACTAGCCATGTTTTCATCTATTCTAATCCTACTTCTAATGCCCTTCTTACACACAACTAAACAGCAACCGATATCAACACGCCCCATGTCTCAGCTCCTATTCTGGGCCCTCGTCCTAGACTTCTTCGTACTCACATGAATCGGAGGTCAACCAGTAAACTCCACATACATCTTAATGGGCCAAACCGCCTCCGTGCTCTACTTCGCCATCATCCTCATCCTCATACCCACAATCGGACTCCTGGAAAACAAAATAA---------------------CTAGC-------TGTGAACGCCCACCGTTGATTATTCTCTACTAACCACAAAGACATTGGCACCCTTTACTTCGTCTTTGGAACATGAGCCGGAATAGTGGGAACAGCACTAAGCCTCCTTATTCGAACAGAATTAAGCCAGCCAGGGCCCCTCCTAGGAGACGACCAAATCTACAACGTAATTGTCACCGCCCATGCCTTTATTATAATCTTTTTCATAGTAATACCCATCATGATCGGCGGATTTGGAAACTGACTACTACCCCTGATAATCGGAGCCCCAGATATAGCATTCCCCCGAATAAACAACATAAGCTTTTGATTGCTCCCCCCATCCTTCATACTTCTACTCTCCTCCGCCTGCGTCGAGGCGGGGGCCGGAACAGGGTGAACTGTCTACCCGCCCCTCGCCGGAAATTTAGCCCACGCCGGACCGTCCGTAGATTTGACAATCTTCTCTCTTCATCTCGCCGGAGTATCCTCTATCCTTGGTGCTATTAATTTCATTACAACAGCAATTAACATAAAACCCCCAGCAATATCCCAATACCAAACACCCTTATTTGTATGGTCCGTCCTAATTACAGCCGTGCTCCTTCTACTATCCCTACCAGTACTAGCTGCTGGAATCACCATACTCCTTACAGATCGCAACTTAAATACAACCTTCTTCGACCCCGCGGGCGGAGGAGACCCCATCCTATACCAACACCTTTTCTGATTCTTTGGCCACCCCGAAGTATACATCCTCATCCTCCCTGGATTCGGAATAATTTCCCACGTGGTCGCCTTTTACTCAGGCAAAAAGGAACCATTCGGCTATATAGGAATGGCATGAGCCATACTCTCTATCGGATTCCTAGGATTCATTGTCTGGGCCCACCACATATTTACAGTCGGAATAGACGTCGACACCCGAGCATACTTCACCACCGCCACAATAGTTATCGCTATCCCCACCGGAGTAAAAGTATTTAGCTGACTTGCCACCATCTACGGCGGCATTGTCAACTGACAAGCCCCGATACTCTGAGCACTTGGTTTCATCTTCTTGTTCACAGTAGGGGGCCTCACTGGCATCGTCCTAGCTAACTCCTCACTAGATATTGTTCTCCATGACACCTATTATGTAGTCGCCCACTTCCACTACGTACTGTCAATGGGGGCAGTCTTCGCCATTATAAGTGGATTCACCCACTTGATTCCCTCTTATTTACGGGATTTACCCTTCACCCAACATGAACTAAAAATCCAATTTATAATTATATTTGTGGGGGTAAATTTTACCTTCTTCCCACAACACTTCCTAGGCCTCTCCGGGATACCCCGACGCTATTCGGACTACCCAGACGCATACACCCTCTGAAACCTATTATCATCTATTGGGTCCCTAATCTCAATAACCGCAGTCGTCCTGCTCATATTTATTGTATGAGAAGCATTCTCATCCAAACGAAAAGTAACAGCACTCGAAATGACAACGACCAACATCGAATGGCTCAACAACTGCCCCCCATCCCATCACACCTATGAAGAGCCCGTATTTGCCCTAGTGCAAACCTCCTTTAAAACATACCACCCAGC------------------CTCAAGAATAGCTAACCCGATACACTTAGGATTCCAAGATGCAATATCCCCTCTGATAGAAGAATTACTGTATTTCCACGACCACACGCTGATAATCCTATTTCTAATCAGCTCCCTCGTATTCTACATAATTTCCGCCCTCCTCCTCCCCAAACTCTACCACTCGAGCGCCTCAGACGTCCAAGAAGTAGAAGTAATCTGAACTATCCTGCCCGCTATTGTCCTCATCTCAGTCGCCCTTCCATCACTTCGTACCCTTTACCTCATGGACGAAACCAACAACCCCTGCCTTACTATTAAAGCAACCGGACACCAATGATATTGATCCTATGAATACACCGATTTCTCTGCACTAGAATTCGACTCCTACATAGTACCCACACAAGACCTGCCTCTAGGCCACTTCCGTCTTCTAGAAGTTGACCACTGCATGATTACTCCAACAAACTCAACCATCCGAGTACTAATTACAGCCGAAGATGTGTTGCACTCATGGGCCATCCCGTCCATCGGAACAAAAATAGACGCACGTCCAGGGCGCCTAAACCAGGTCATACTCACACTGGCCAATTCCGGTGTATTTTACGG-CCAATGCTCCGAAATCTGCGGGGCAAACCACAGCTTCATACCCATTGTCATAGAAACTATCCCATTAAACCACTTCCAACTCTGACTAAAAGACTGCATGTCCTCCTATGTCACACCAAACACACTCCTTTCACATAGTCCACCCCAGCCCCTGACCCCTCGCCGGGGCCATAGCCGCCATATTATTAACAACAGGCCTGACCTTCTGATTCCACTATGACTCTAGCCTTATTCTATTGCTCGGCCTAATCACCACTCTATTAGTAATACTCCAATGATGACGAGACATTATCCGAGAAAGCACCTACCTAGGACACCACACACCTGCAGTACAAAAAGGACTACGCTACGGCATAATCCTTTTTATCACATCAGAGGTCTTCTTCTTCCTGGGCTTCTTCTGAGCATTTTATCACTCAAGCCTTTCCCCCACCCCTGAGCTAGGGGGACAGTGACCCCCAGTCGGAATTACCACCCTTGACCCATTTGAAGTTCCCCTCCTAAACACAGCTGTCCTCCTTGCCTCTGGGGTAACAGTAACCTGGGCCCACCACAGCTTGATGGAAGCCAACCGAACACAAGCAATTCAGGCCCTAACACTCACCGTACTCCTTGGCCTATACTTCACCGCCCTTCAAGCCATAGAGTACTACGAAGCCCCCTTTACAATCGCAGACAGCACCTACGGATCAACATTCTTCGTTGCAACCGGCTTCCACGGCCTCCATGTTATTATTGGCTCAACATTTCTCATAGTCTGCCTATATCGACAGACAAAATATCACTTCACATCCAACCACCACTTCGGGTTCGAAGCCGCTGCCTGATATTGACATTTTGTAGATGTCGTCTGACTCTTCCTTTACATCTCAATCTACTGATGAGGTTCATATAGACTTTCTCATCGTCGTGCCCCCCGCTCTACTTATCATCTCCATCCTGATCGCGGTTGCATTTTTAACGGCCCTAGAACGAAAAATTATTGGCTACATGCAACTACGAAAAGGACCAAACATCGTTGGCCCACTTGGCCTACTTCAACCCCTCGCCGACGGATTTAAACTTGTTATTAAAGAACTAACCCTCCCGCTACTTGCAACCCCAGCCCTCTTCATCCTATCCCCAGCAGCAGCCCTAATGCTAGCCCTTACGATATGATCCCCCCTCCCCATGCCATTTCCCCTGGCAGACCTTAACCTAGGACTACTATTCCTACTGGCCATATCAAGCCTCATGGTCTACTCATTATTATGATCCGGGTGATCCTCAAACTCAAAATATGCCTTAATAGGTGCCCTTCGGGCAGTTGCTCAAACTATCTCCTACGAAGTAACACTGGCCATCATTGTATTATCTATTGTCTTACTGACTGGGGGATTTTCGCTACATGCCCTCACCACCACACAAGAACCCCTATACCTGCACTTAGCCACCTGACCATCGATAATAATATGGTATACCTCTACACTAGCAGAAACAAACCGGGCCCCATTCGACCTAACAGAGGGAGAGTCAGAGCTAGTATCCGGATTTAACGTTGAATACGGCGCAAGCCCCTTCGCACTCTTTTTCCTGGCCGAGTACGCCAACATTATATTAATAAACACCCTGACTGTCACCCTATTCTTAAACCCATCTACCCCCACTTCCATCCCAATACTCTTCACCATCGCCCTAATAAGCAAAACCCTTCTACTAACTATAAGCTTTCTATGAATCCGAGCATCCTACCCCCGATTTCGCTATGACCAGCTTATGCACCTTTTATGAAAAAGCTTCCTACCCACAACATTAGCCCTCTGCCTATGACACTCATCACTTCCAATATCAACATTTGGGCTGCCCCCAACAACATAGATGCCCCTCTCCCAGCCAATTATCTTAACGACCCTGACCGCCACAACACTCGTCTTTCTACTATCAACCCACCTTGTACTAATATGAGCCGCACTAGAACTTAGCACACTAGCAATCCTCCCCCTAATCGCTAATAAATCCCACCCCCGAGCTATCGAAGCTTCTACAAAATACTTTTTAATACAAGCGATAGCCTCCACACTAATCATCTTCTCAGGAGCGCTCAACTATGAAATAACAGGAAGTTACCAAATCGCAGAGTTAACGGACTTAACCTCAATAATTGTGCTAACCCTCGCCCTGTTTATTAAAGTGGGACTAGTGCCATTCCACTTCTGAGTACCAGAAGTCCTACAAGGAATACCCACAGCTCCTGCAATCTTTCTACTGACATGGCAGAAGCTAGGCCCACTAGTTATACTCTTCTTAATTAGCCACCTCATCAGCCTTAAACTAATCTTTATAGTGGCCGTCTTATCCTCTCTTATTGCAGGTTGGATAGGACTAAATCAAACTCAAGTACGAAAACTAATAGCATTCTCATCCATCGCCCAAATGGCATGAATTATTGTAATCATTAAATACGCCCCATCCCTGACAATCCTGACTTTTTATATCTATTCCACTACCGTCTCCGCCACACTACTCACACTAGATAAAATATCAACAACCTCCACCAAACACCTCATTACCTCCTTCTCAAAATCCCCGACTGCAGCCACCCTCCTAACTCTCTCCCTTCTCTCGCTATCCGGCCTTCCACCCCTGGCCGGCTTTCTGCCAAAATGGCTAACCGTTAATCAACTTGTCTCAGAAAAAGCAACTTGAATCGCTCTCCTCATACTCATGGCCTCCCTCTTAAGCCTTTTCTTCTATCTTCGGCTATGGTACAACTCCTCATCGACCCTACCGCCAAATACCACAAACACAACCCGCCTCTGACGAAAACTAACCCCTCAAAGCAACCTAACCATTAACCTCCTTGTCCTGGCTGCCACCACCCTTCTGCTATCCGCCACATTAATGAAAGCAATTACCAAACAAGAA-ACCCCCAAAGCTAAAAGAATCAACCTTCTTACCATATTCATAGTAACCTCCATCACCGCCGCAGCCGTAATCACTATAAACCTGCTAATAACTGAAATACTACCTGACTCAGAAAAACTATCCCCCTACGAGTGCGGATTTGACCCCCTCGGCTCTGCTCGCCTACCCCTATCAATTCGATTTTTCATGATCGCCATCTTATTCCTACTTTTCGATCTTGAAATCGCTATCCTGCTCCCACTCACATGAGCCACCCACGCCCTAAACCCCCTAAAAACCGCCACATGGGCCATCATTATCTTCCTATTCTTATTCATCGGATTAACATACGAATGACTCCAGGGCGGCCTAGAATGAGCAGAATAGATGCTAAAACTTCTTCTTCCCACAACCATGCTGATCCCAACAATCAACCTCCTCCCAAACAAAATTACCTGGTTGCCGCCAACAGCCTACTCGATTGTCGTAATGACCCTAGCCCTCCTAATCCTCAACCCATCTGACACTCTTATAGCCACTAGCCGCCTGGCCCTAGGTAGCGATCAATTCTCAACCCCTTTAATAATTCTGTCCTGCTGACTTCTCCCCTTAATACTTATAGCCAGCCAAAGTTCTATATTCAAAAACCCCGCCCCCCAAAATCACATATTTATCACAATCCTTGCAATACTTCAACTTGCTCTACTTATGGCATTTATGGCCCTAGACTTAATACTATTCTACATCTCCTTCGAAGCCACCCTAATCCCCACCCTTGTAATCATCTCCCGCTGAGGGGCTCAAACAGACCGCCTAAACGCAGGTATTTATTTTTTGTTTTACACTATCGCCAGCTCAATCCCGCTGATAATTAGCACCTTGGTAACCTACAACCTAAAAGGGACTTTATCTCTCCCCGCCCTACAACTAATTCCAATAGCAAACCCCCTCTCCTGAACAGACACACTTCTATGACTATCCATACTCCTAGCCTTCCTAGTAAAAATCCCCCTTTACGGCCTCCACCTGTGACTCCCCAAAGCTCACGTCGAAGCCCCCATTGCGGGCTCCATAATCCTAGCTGCAGTACTCCTAAAACTTGGCGGCTACGGCCTGTTACGAGTAGTAAACTTACTCACCGAACAAATAAACACTATCTACCTCCCCTTTTTAACCTTGGCGCTCTGAGGGGCCCTTATGACTGGCCTTATCTGTTTGCGACAAACTGACCTAAAATCCTTAATTGCCTACTCATCAGTAAGTCACATAGCCCTAGTAACGGCTGCAATTCTTGCGCGGAATCAATTAGCCCCAGCAGCCTCAATACTTCTAATAATAGCCCACGGACTGACATCCTCCATGCTATTCTGCTTGGCAAATTTCAACTATGAACGTACTCACACTCGGACACTCCTGGCAATACAAGGTATACAACTCACTACACCTGCCCTTACAACTTGATGATTTTTAGCTAGCGCAATGAACATAGCTCTCCCCCCAACAATTAACCTCATAGGAGAACTAACTATTATTGTCTCACTTTTTAGCTGGCTAGACATCACACTATTGTTAACTGGACTAAGCTCATTCATTACAGCAATCTACACCCTCCACATATTCTCATCCACCCAACAAGGAACACTACCCGCCCACACCACCCTCCTCCCACCTGCCCAAACCCGAGAACACCTCCTAATACTACTTCACTCACTACCATCGATTATTCTCATCGCCAACCCCCAACTCATCTTCCCCCAATAGATAATCACATCCATAAGCGTCATATTTTTATACTCCTTCGTCATCTGCACCATCGGCCTAATCATACACCACACACACCTACTCTCAACACTACTATGTCTTGAGGGTATGATACTGTCAATTTTCATGGCCTTGACAATATCAGCGCTCAGTTCAAACACCTCCTCATTCATCCTGCCACTAACAATTCTAACCCTTTCTGCCTGTGAAGCAGGCGTCGGCCTGGCCCTACTGGTTGCCTCTGCTCGAACACATAACACAGCAAACCTTAAAAACCTAAACCTCCTCCAATGCTAAAT----GCAACAATCAACCCTATTTGTTACACTATTCACCCTACCCCCCTTCATCCTCGTACTATCCTGCTCCCTCCCAGCCCCCAAAACCCTCCAC---CCGGCTGACTTTAAAAGTATAATAACTAAACTGGCATTCTTGCAAAGCCTCCCTCCCCTCCTCTTATTAGTCTATAACAACACAACCGCTCTCTCATTCCAATGACACTGACTCAACGTAGGAACTTGCTCTGTTCACCTGGGCCTTAAAGTTGACACCTTCTCAGTCTTCTTTATCCCAACAGCCTTATTCGTCACATGATCAATCATAGAGTTCACCAAAGCATACATATACTCAGACCCCAAAATCACCAGCTTCTTTAACCACCTCCTAATTTTTATTCTAATGATGATCCTTCTAATCTCCGCTAACAACTTACTCATATTATTCGTGGGCTGAGAGGGAGTAGGCATCTTGTCGTTCAAGCTCATCAACTGATGATCCTTCCGAGCGGACTCTAACAAGGCAGCCCTACAGGCCATCATTTACAACCGCCTAGCAGATATCGGAATACTCGCTAGCATTTCATGAATGGCCCTAAATAGCCTCACCCTCGACGCCCAAGACGTCCCCATATCCCCCGACCACTCACTCATCCTAGCCATAGCCCTTGTCCTAGCAGCAGCTGGAAAATCAGCCCAATTTGGCTTCCATCCCTGGCTCCCAGCAGCCATAGAGGGCCCCACACCAGTCTCAGCCCTACTCCACTCAAGCACCATAGTAGTAGCAGGCATTTTCTTATTAATCCGAACCTCCCACATCATCTACAGCAGCCAAACAGCAACCACAGCCTGCCTGCTCCTAGGAGCAGCAACCTCCCTGCTCACAGCTGCCTGCGCCCTCACCCAAAATGATATGAAGAAGATTATTGCATTCTCAACCTCAAGCCAACTTGGACTAATAATGAGCACAATTGGACTTAAACAGCCCGAACTTGCATTTCTACACATCTCAACACATGCCTTTTTTAAAGCAATACTATTCCTGTGCGCAGGGTCAATTATCCATAGCCTTAACAACGAGCAAGATATTCGAAAGATGGGCGGCCTTAAAAAAGCAATACCCATCACCACCTCTTGCTTGACTATTGGAGCATTAGCTCTCACCGGCATACCCTTCCTCTCAGGATTTTTTTCCAAAGACGCCATTATTGAATCGCTAAATACCTCATACACTAGCGCCTGGGCCCTTACCCTCGTCCTACTCGCCACCTCCTTCACTGCAGTTTATAGCTTCCGCATGATTTATTTTACCCTACTAAACACCAACCGCCTAACACCCATGAACCCCATTAATGAAAACCCAGAAACTGTAAACCCCATCATACGTCTAGCTGTCGGAAGCATTGTAGCCGGGCTATTAATTTCAACCCACATACTACCCTCTAATACCCCCCAACTAACCATGCCTGGCCCAATCAAACTTGCAGCCCTCACCATCACAATAGCTGGCCTACTAGTCGCAATAGCCCTGACCTACGCCACCAACAAATTCCCCCCATCCACCAACGACACTCAACTGCCCTTCCTAACTAAACTGGCCTACTTCAACCTCCTATTCCACCATCTCTTCTCCACCACTGCCCTTTACATAAGCCAGAAACTATCTACCCATCTGACCGACCAAACATGATACGAAACTATCGGACCAAAAACATTAGCCTATCTTCAAACCCTGTTAGCCAAAACTATTACCCCCTATCACAAAGGAAAAATGAAACAGTACTTCAAAACCTTTTTACTAACCATTGCCGTAATTATCTTCTTC-----CTTCTGT-------TCTAA---------------------------------ATGGAGATTACGTT---GTTTTTTTGTTGTTTTATACTAGTATGTGCGATAATGGTAGCGGCAAGTTCAATGATCCACTTTGCTGTAGTGAGCTTGCTTTTTGTGGCTGTGTTCGGTAGTGGTTTGGTAGTAGTTGAGGGTGGAAGTTTTATGCCCCTGGTGGTATTATTAATTTATCTGGGGGGCTTACTGGTGGTATTTGCTTTTTGTGTGGGGTTCACTGATGATCCTTTTATAGGGTTTTGAGGGGTGGGGGTATCCAAGGGGGCTGTGTTTCTTTATTGAGTGGGGTTGTTGATCGGGATCTGTTGTGCGTGGGGGTATTTGTGAGGGGGGATGTGGATTAGTGTGGGAGAGCCTTGGAGTG---AGGTTGTGGT------------CGGTGAGTCATTAGGGGTCAGTGAGATGTATTGTAATGGGTGAGTATTTGTTGTGGTGTGTGGATGAGCCCTGCTAATTGTATTATTCGTAATCACGGGGCTCATTCGTGGCCATCGGCGAGGGGCATTTCGTTCTTTTAGA

*Paleosuchus palpebrosus*

------------ATGAACACAAACCTATTCGACCAATTCCTAATCCCCAACCTCTTGGGAACCTCCCTACTAACACCCGCCCTACTAATAACCATTGCCCTCGTGCTAAACCCCCAAAATCTATGACTCCAACACCCAACAGCAACAATTAAAACCTGACTCATCAACCAAATTACCAAACAAATCATAACCCCAATCAACAAGTCAGGACATAAGCACTCCCTAACCCTTATCTCCATCCTAGTTCTCCTCCTCCTCACAAACCTACTAGGCCTACTCCCATACACATTTACACCAACAACACAACTCTCCATAAACATAGCCCTCGCTATCCCCCTATGACTGATGACTGTACTAACAGGCATGCGAACTCAACCAACAACCTCCTTAGCCCACCTTCTTCCAGAAGGAACCCCCACCCCACTAATCCCAGTCCTCATTATAATTGAAACAATTAGCCTATTAATTCGACCAATCGCATTGGGTGTACGATTAACAGCCAACCTAACCGCAGGCCACCTACTAATTCAATTAATCTCCATTGCTACACTAAACCTCTGACCCATCATACCACCCCTCAGCCTATTAACACTAACAACCCTGATCCTGCTCCTCCTACTAGAATTCGCCGTAGCCATAATCCAAGCATACGTCTTCGTCCTCCTACTCTCCCTATACCTACAAGAAAACACATAAATGCCCCAGCTAAACCCAGAACCCTGACTAACAGTCCTTCTAACCACCTGAATCTTCTTTATCGCCATTCTCCAGCCTAAAATCGCCACACTAACCGCCACAAACAACCCCACCCCACACAAACCACAGACCACC-AAAACATGACACTGGCCATGAACACAAACCTATT--------------ATGACCCACCAACTACGAAAATCCCACCCACTCCTCAAACTCTTCAACCACTCCCTCATTGACCTCCCAACACCCTCAAACATCTCCGCCTGGTGAAACTTTGGATCACTACTGGGCCTGACCCTCCTAATCCAAATTCTAACAGGAGTCTTCCTAATAATACACTTCTCATCAAGTGACACCACGGCCTTCTCATCCGTCGCCTACACCTCCCGCGAAGTCTGATTCGGATGACTTATCCGAAACCTCCACACAAACGGAGCCTCACTCTTCTTCATGTTTATTTTCCTCCACATCGGACGGGGCCTGTACTACGCCTCCTACACCCACGAAAAGACATGAGCTGTAGGAGTTATTATACTCTTCCTACTAATAGCTACAGCATTCATGGGTTATGTCCTCCCCTGAGGACAAATATCATTCTGAGGGGCAACCGTAATTACAAACCTGCTGTCTGCCACCCCATACATCGGAGACACCATCGTGCCATGAATTTGGGGGGGACCCTCCGTAGACAACGCAACACTCACACGCTTTACCGCCCTTCACTTCCTCCTCCCGTTTACCCTCCTAGCCCTGCTCATCACCCACCTGATCTTTCTACACGAACGGGGGTCCCTCAACCCCCTCGGACTAAGCCAAAACGCCGACAAAATCCCATTCCACCCCTACTACACACTAAAAGATGCTCTAGGAGCAACACTAGCCGCCTCCCTACTACTCACCTTCGCCCTATACCTCCCAACCCTACTAGGCGACCCAGAAAACTTCACCCCAGCAAACCCCATAACCACCCCATCACACATCAAACCTGAATGATACTTCCTATTTGCCTATGCCATCCTACGATCTATCCCAAACAAGCTCGGAGGCGTACTAGCCATATTCTCCTCCATCTTTATCTTATTCCTAGTGCCTGCCCTTCACACAGCCAAACAACAGCCCATATCCGTCCGCCCCTTATCCCAACTCCTATTCTGAGCCCTCATCCTAGACTTCTTTGTACTTACATGAATTGGGGGCCAACCAGTAAACCCCCCATTCATCCTAATGGGCCAAATCGCCTCCTCACTCTTCTTCATCACCATCCTCATCCTCATACCCACACTAGGGTCCCTAGAGAACAAAATAACC------TAA-----------------------ATGTTCATCAACCGCTGATTTTTCTCCACAAACCACAAAGACATCGGCACCCTGTACTTCATCTTCGGGGCCTGATCCGGAATAGTAGGCACAGCACTCAGCCTTCTCATCCGAACAGAACTAAGCCAACCAGGACCCCTACTAGGAGACGACCAAATCTACAATGTAATCGTCACTGCCCACGCCTTTATTATAATCTTTTTTATAGTAATACCCGTCATGATCGGCGGATTCGGAAACTGACTCCTGCCCTTAATAATTGGAGCCCCAGACATAGCATTCCCGCGAATAAACAACATAAGCTTCTGACTACTCCCCCCATCCTTCACACTGCTGCTCGCCTCTTCCTGCATTGAAGCGGGGGCCGGAACAGGATGAACCGTCTATCCCCCCCTAGCTGGAAACATAGCCCACGCCGGACCATCAGTAGACCTAACCATCTTCTCCCTACACCTTGCCGGAGTATCTTCCATCCTAGGCGCAATCAACTTCATCACAACAGCCATTAACATAAAACCCCCAGCCATGTCCCAATACCAAACACCCCTATTTGTTTGATCGGTCTTAATCACAGCCGTACTACTCTTACTCTCCCTCCCAGTACTAGCTGCCGGAATTACTATACTACTCACAGACCGAAACCTAAACACAACCTTCTTTGACCCCGCAGGAGGGGGGGACCCCATCCTATACCAGCACCTTTTCTGATTCTTCGGCCACCCCGAAGTCTACATCCTCATCCTACCTGGATTTGGAATAGTCTCACACGTCGTCACCTTCTACTCAAACAAAAAAGAACCATTCGGCTACATAGGAATAGCATGAGCCATAATATCTATCGGATTCCTAGGATTCATCGTATGGGCCCACCACATATTCACAGTCGGAATAGACGTCGACACACGAGCATACTTTACTACTGCCACAATAGTTATTGCCATCCCCACCGGGGTAAAAGTGTTTAGTTGACTAGCAACCATCTACGGGGGCATCGTCAACTGACAAGCCCCAATACTATGAGCACTAGGCTTCATCTTCTTATTCACCGTAGGTGGCCTTACTGGAATCGTCCTAGCCAACTCCTCCCTAGACATCGTTCTCCACGACACCTACTACGTAGTCGCCCACTTCCACTACGTATTATCAATAGGAGCAGTCTTTGCCATCATATGCGGATTTACCCACT-GATTCCCACTA-TTCACAGGATTTACCCTCCACCCAACATGAACAAAAA-TCCAATTCACAATTATATTCTTAGGAGTAAACCTTACCTTCTTCCCACAACACTTCCTAGGGCTATCTGGAATACCTCGACGATACTCTGACTACCCAGACGCATACACCATCTGAAATCTAACATCATCAATTGGATCCCTAATCTCCCTAACCGCTGTCATCCTACTAATGTTCATTGTATGAGAAGCATTTTCTTCCAAACGAAAAACAACCACACCTGAGATAACAACAACCAACATCGAGTGACTTAACAACTCCCCACCACCCCACCACACATACGAAGAACCAGTTTTTACTGTAACACACAAACCAACCGACCTC-----------------------------CTAAACTATGGCCAACCCCACACATCTGGGCTTCCAAGACGCAATATCCCCCCTCATAGAAGAGCTATTATACTTCCATGACCACACTCTAATAATCCTCATCCTAATCAGCTCCCTTGTATTCTACACAATCTCCGCCCTGCTCCTCCAAAAACTCTATCACTCAAACACCTCCGACGTCCAAGAGGTAGAAATAGTATGAACCATCCTACCAGCCATCGTCTTAATTTCAATCGCCCTCCCCTCTCTGCGAGCCCTTTACCTTATAGACGAAACCAACAACCCATGCCTGACTATCAAAGCAACCGGACACCAGTGATACTGATCCTACGAATACACTGACTTCTCCACAGTAGAATTTGACTCCTACATGACACCCCCACAAGATCTCTCTCCAGGTCACCTCCGCCTCCTAGAAGTTGACCACCGCATAATTACCCCAATAAACTCAACAATTCGGATATTAATCACAGCAGAAGATGTCCTGCACTCATGGGCAATTCCATCCATCGGAACAAAAATAGACGCAGTCCCAGGACGCCTAAACCAAGTCATAATTACACTAACCAACCCTGGCGTATTCTACGG-CCAATGCTCCGAAATCTGTGGAGCAAACCACAGCTTCATACCCATCGTAATAGAAACCACCCCATTAAACCACTTTCAACTCTGACTAGAAGACCACACTACCTCATATGTCCCACCAAACCCACCCATTCCATATAGTACACCCAAGCCCCTGACCCCTAGCCGGCGCCATAGCCGCCATATTACTAACAACCGGCCTAACCTTTTGATTTCACTACAACTCTTGCCCCCTCCTCCTACTAGGCCTGACCTCAGCCCTACTTGTAATATTCCAATGATGACGAGACATTGTACGAGAAAGCACCTTCCTAGGACACCACACACCCCCAGTACAAAAAGGATTGCGCTACGGCATAATTCTCTTTATTACATCTGAAGTCTTCTTTTTCCTAGGCTTCTTCTGAGCATTTTACCACTCAAGCCTCTCCCCAACCCCAGAGCTAGGAGGACAATGACCCCCTACCGGAATCACCCCCCTCGACCCCTTTGAAGTCCCCCTCCTTAACACAGCCGTCCTACTCGCCTCCGGAGTAACAGTAACATGGGCCCACCACAGCCTAATAAACACCCACCGCATGCAAGCAATCCACGCTCTCGCACTCACTGTACTTCTCGGATTCTACTTCACCACCCTTCAAGCCATAGAATACTATGAGGCCCCCTTCACTATCGCAGATAGCACCTACGGATCAACATTCTTTGTTGCAACAGGCTTTCATGGCCTCCACGTCATCATCGGCTCAATCTTCCTCACAGTCTGCCTATATCGACAAACAAAATACCACTTCACATCCAACCACCACTTCGGATTCGAAGCCGCCGCCTGATACTGACACTTCGTAGACATAATCTGACTATTCCTCTACATCTCAATCTACTGATGAGGCTCATATCGACTCCCTCACCATCCTCCCCCCAATCTTATTTATTGCCTCCGTCCTAATTGCAGTCGCCTTCTTAACAGCCCTAGAACGAAAAGTTATTGGCTACATTCAATTACGAAAAGGACCAAACATTGTTGGCCCCATCGGCCTGCTCCAACCCCTCGCAGATGGATTCAAACTTATTATTAAAGAACTGACCCTCCCCCTACTTGCCACACCAACCCTATTCATCCTCGCCCCGGCAGCAGCCCTAATACTAGCCCTCACCATGTGGTCCCCCCTTCCAATACCATTCCCCCTCACAGACCTCAACCTAGGCCTGCTTTTTCTACTAGCCATATCAAGCCTCATGGTCTACTCACTTCTCTGATCCGGGTGGGCCTCAAACTCAAAATACGCCCTAATAGGGGCCCTCCGAGCAGTAGCCCAAACAATCTCCTACGAAGTCACACTAGCCATTATTGTACTCTCCATTATCTTGCTCTGCGGGGGGTTCTCACTACACGCCCTCACCACCACACAAGAGCCCCTATATCTTGCACTGCCTGCATGGCCCCTAATAATAACGTGATATATCTCCACACTAGCAGAAACAAACCGCGCCCCATTCGACTTAACAGAGGGCGAATCAGAACTCGTATCTGGGTTCAATGTTGAATACGGCGCAAGCCCCTTCGCACTGTTCTTCCTAGCCGAATACGCCAACATCATGTTAATAAACACCCTGACCGTCATCCTCTTCCTCAGCCCATCACCCCAAACCCACCCTCCAACGCTATTCACCATCACCCTAATAACCAAAACCCTCTTACTGACTACAACCTTTCTATGAGTCCGAGCATCCTACCCACGATTTCGCTATGATCAACTCATACACCTCCTATGAAAAAGCTTCCTCCCCCTAACATTAGCCCTCTGCCTATGACACACATCACTACCAGTCTCAATATTTGGACTTCCCCCAACAACCTAGATGTCCCTCTCATTACCCCTCATCCTAACAACAATGGCCCCAGCAACACTCATCTTCCTCATATCATCCCACCTAGTACTAATGTGAATCGCACTAGAACTCAACACACTGACAATCCTTTACATAATTGCCAACAAATCCCACCCCCGAGCCATTGAAGCCACCATCAAATACTTCCTCACACAAGCACTAGCATCCACATTAATTATCCTTTCCGGAACTATCAACTACGAGATAACAGGAAGCTGGGAAATCACAGAAATAACTAGCCCAACCCCCTTAACCATACTCACCCTCGCCCTATCCATCAAGCTAGGATTAGTCCCATTTCACTTCTGAGTGCCAGAAGTCCTTCAAGGAATACCCACACTCCCCGCAATCTTCCTACTTACATGACAAAAAGTAGGCCCACTAATTATGTTCTTCTTAATCAGCCCCTTTATCAACCATAATTTAGTCTCCACAATGGCCATTATGTCCTCCACTGTTGCAGGGTGGCTGGGGCTCAACCAAACCCAGGTACGAAAGCTAATAGCAATGTCCTCCATCGCCCAAATAGCATGAATAATTGTCGTTATTAAATACGCACCCTCCCTAACAGTCCTCTCTTTCTACGTGTATTCAATAACCGTCTCCGCCGCACTACTAATGCTAAACAAGCTATCTGTAACCACCATAAAATCACTTGCCACTTCCTTCTCAAAAAACCCAATTGCCACCTTACTCCTAATAGCCTCCATCCTCTCACTATCCGGCCTTCCCCCCCTGGCCGGCTTCACACCTAAATGATTAATAATCAACCAGCTTGTTGCAGAAAAGGCAGTCTGAACCGCACTTCTCATACTAGCATCCTCACTCTTGACCCTGTTCTTCTACCTTCGCATGTGATACTACACCATCTCCACCCTGCCCCCAAACACCTCAAACATATCCCGCCCTTGACGAAAACCCACCTCCAAAAGCAACCTCACCGCCAACACCCTCGCTACAGCTGCCTCCACCCTCCTACTATGCACTACACTCATAAAAGCAGCCACCAAAAG------------------AA----TAGACCTCCTCATTATATTCTCAATAGCCTCCATCACTGCAACAATCCTGATTATCTCAAACCTCACAATAGCCGAAGCATCGCCCGACCCTGAAAAACTATCCCCATACGAATGTGGATTTGACCCCCTAGGGTCTGCTCGACTTCCACTATCAATCCGGTTCTTCATGATCGCTATCTTATTCTTACTTTTCGACCTTGAAATCGCCATCTTACTCCCACTCACGTGATCCACCCACACCTTTCACCCAACAAAAACCATCACATGTATTATCGCAATCTTCCTACTACTATTCATCGGTCTGATATACGAATGACTCCATGGCGGCCTAGAATGAGCAGAATAGATGCTAAAAATCCTGCTACCCACGGCCATGGTTGGCCCAA---CAACCCTCACCCCAAACAAGACCATCTGACTATCCCCAACAACTTGCGCCACTGCTGTAATAATTATCCCCCTACTAACCCTAAACCCCTCAGATACTTTAACAAACCCCAGCAACCAAGCCCTCGGTAGCGACCAATATTCAAGCCCCCTGATAATACTATCCTGCTGACTCCTCCCCCTGATGCTCATGGCCAGCCAAAACGCAATATCCACAACCCTGGCCCCCCAAATCCAAATATTCATCTCCATTCTTGCAACACTCCAACTCACCCTGCTCATGGCATTCATGGCCCTAGACTTAATACTATTCTATATTATATTTGAAACCACCCTACTTCCAACCCTAGTAACCATCTCCCGATGAGGCGCCCGACCAGAACGTCTAAATGCCGGAGCCTATTTCCTATTCTACACTATTACCAGCTCAATCCCCCTAATAATTAGCATTCTAATAATCCACAATCTCATCGGCACCTTGTCCCTCCCAATCCTACAACTAACCCCAATAACGAACCACCCCCCCTGAACAAATACACTATTCTGACTCTCAACACTTGTAGCCTTCCTGGTAAAAATCCCAATCTACGGACTACACCTCTGACTCCCCAAAGCTCACGTCGAAGCCCCAATTGCAGGATCAATAGTACTGGCCGCAGTACTTCTAAAGCTGGGAGGATACGGGATGCTACGAGTAACAAACCTGCTCACCGCGCAGGTTAATACTACCTACCTCCCCCTACTAACCCTAGCACTCTGAGGGGCACTAACAACAAGCCTCATCTGCCTACGACAAACAGACCTAAAATCCCTAATCGCCTATTCATCGGTTAGTCACATAGCCCTGGTAACAGCCACGATCCTCGCCCGAGACCACCTTACCCCAACAGGATCCATAATCCTAATAGTAGCCCACGGACTAACCTCCTCCATACTATTCTGTCTAGCAAACTTCAATTATGAACGAACCCACACCCGAACACTCATCACCACACAAGGCATACAACTCACCTCCCCCGCCCTCACAGCTTGGTGGTTCCTAGCCTGCTCAATAAACATAGCCCTCCCCCCAACATTCAACCTAACCGGCGAACTAACCGTTATTACATCGCTATTTAACTGACTAGACATTACAGTTATCCTAACAGGACTGAGTGCCTTCATAACGGCAGTCTACACCTTATACATATTCTCAGCCACCCAACAATGAACACTACCCACTAATGCCACCCTATTACCTCCAACCCAAACCCGAGAACACCTCCTGATACTCCTCCACCTCCTCCCCTCTGCGGGCCTTACCACCAACCCAAAACTCACCTCCGCCCAATA---AATTACCCCCACAAATACACTTTTTTTGTTCTCCTTCTCTCTTTGCATCATTGGCCTAATTACCCACCACAACCACCTCCTCTCAACACTCCTCTGCCTTGAAGGGATGGCACTCTCTATCTTCTTAGCCGTCACAATATCCTCACTCCAATCACACCCCTCATCATTCATCCTCCCCCTAACAATCCTGACCCTCTCTGCCTGCGAAGCAGGCACCGGCCTCGCCCTACTAGTCGCCTCCGCCCGAACACACAACACCGCCAACCTTAAAAACCTAAACCTCCTTCAATGCTAAAT----GTCCCACACGACACTCCTCCAAACATTCTCCGCCATCCCCTTTCTAATCCTATTACTGGCAACCCTTCCCCTAGCCCATAAAACCAACAACACACCAGCCAAATCCAAAACAATAATAGCCAAACTCGCTTTCTTCACAAGCCTCCCCCCTCTCTTTTTAGTGGTGCTAAATAACAGCCCCGCCCTGTCACACCACCTATCTTTATTCCACCTAGAATCCCTCAACCTCCCCATCACCCTAAAAATTGACACTTATTCAACCTTCTTCATCCCAACAGCATTATTCATCACATGGTCAATTATAGAATTCTCCAAATCGTACATAGACTCAGACCCCAAAATTACCAGCTTCTTCAACCACCTCCTAGTATTTATCCTAATAATAATAATCCTAGTAAGCGCTAGCAACCTATTCCACCTCTTTATCGGCTGAGAAGGGGTAGGCATTATATCGTACCTACTCATCAACTGATGATCCTTCCGAGCAAACGCCAACAAAGCAGCGCTCCAGGCTATCATCTACAACCGACTATCAGACATTGGAATCCTCACCACACTGACATGAATAACCACCCACAATCTCACCTTAGACATCACAAACCTCTCC---CCCCCAAACTACCCACTAATCCCAGCCGCAGCCATCATCCTAGCAGCAGCGGGAAAATCAGCCCAAATTGGCCTCCACCCCTGACTCCCAGCAGCAATAGAAGGGCCAACACCAGTTTCCGCCCTACTCCACTCAAGCACCATAGTAGTAGCAGGCGTCTTCTTACTAATCCGAACTTCTCCAATCATCTATAGCAGCCAAACAACAACTACAGCCTGCCTAATTCTAGGAGCAATCACCTCTCTATTCGCAGCCGCATGCGCTTTCGCCCAAAATGACATAAAAAAAATCATCGCATTCTCAACCTCAAGTCAACTTGGACTAATAGTAACCACAATTGGACTTAAACAACCCGAACTCGCATTCCTACACATCCTAATGCACGCCTTTTTCAAAGCAATACTCTTCCTGTGCGCAGGATCAATCATCCACAGCCTCAATAACGAACAAGACTTCCGAAAAATAGGAAACCTCAAAAAAGCAATACCAATTACCACCTCTTGCCTTACCATCGGAGTACTAGCCCTCACCGGCACGCCCTTCCTCTCCGGATTCTACTCCAAAGATGCCATTATTGAATCACTAAACACCTCACATATTAACGCCTGAGCCCTTGCCATCACCCTACTCGCCACCTCCTTCACTGCAATCTATAGCCTCCGCATAGTCTACTTCACCCTACTAAATACCAATCGACTAGCAACCACGAGCCCGATAAACGAAACCCCAAAAACCACCAACCCTATTCTCCGTTTAGCCATTGGAAGTGTAATCACAGGACTATTGACCTTAAATTTCATACTACCAACCCACACCCCCCAACTAACTATACCAACCACAATCAAACTTATAGCCCTAACCATCACAGCAATTGGCATACTCACTGCAATGGCCCTAACCCTCACCACCGACAAATTCCCACCATCCACAAAAGATACCCAACCACCCCCGCTAACAAAACTAATATTCTTCAACCCAATTATCCACCACCTATTCTCCTCCACCACCCTGCACATCAGCCAAAAGCTCTCCACACACCTAACAGACCAACTATGATATGAAACCCTTGGGCCAAAAACAACGGCCAGCCTGCAAACCATGATAACCAAAACACTAACCCCCTACCACAAAGCAAAAATAAAAAAATACCTCAAAACCTTCCTCTGAGCCATCACCATCACCCTCCTCCTC---------TGAA---------GG---------------------------------ATGGAATTTACATT---ATTTTTGGGTTGTCTGGTGTTGGCGTGTGCGGTGATGGTGGGGGCGAATTCTGGGATTCATTTTGCTGTGTTGAGCTTGGTATTTATGGCTGTGTTTGGTAGCTGTTTGGTGGTGATTGAGGGTGGGAGTTTTATGCCGTTGGTGGTGTTGTTGGTTTATTTGGGGGGGTTGTTGGTAGTGTTTGCTTTTTGCGTTGGGTTTACTGACGATCCGTTTG---------GGATGGTGGGGGCTTCTAAGGGGTTTGTGCTTTTTTGTTGGATTGGTCTTCTTGTTGGGGGGGTTTGTATGTGTGGACATTTGTGGGGTTGGTGGGTCGTTGGGGTGAGCGGGTTGGTGGATGTAGAGGTTGTTGGGGGTGGGGCGGCTGATGAGTTGTTGGGGGTGGGTTTAATGTATTCAAGTGGGTGGGGTTTTGTTGTGATTTGTGGTTGGGCCTTGCTGGTTACGTTGCTCGTGATTACGGGGCTTGTTCGGGGTTGTTGTTGGGGGTCACTTCGTCCCTTCAGA

*Paleosuchus palpebrosus 2*

------------ATGAACACAAACCTATTCGACCAATTCCTAATCCCCAACCTCTTGGGAACCTCCCTACTAACACCCGCCCTACTAATAACCATTGCCCTCGTGCTAAACCCCCAAAATCTATGACTCCAACACCCAACAGCAACAATTAAAACCTGACTCATCAACCAAATTACCAAACAAATCATAACCCCAATCAACAAGTCAGGACATAAGCACTCCCTAACCCTTATCTCCATCCTAGTTCTCCTCCTCCTCACAAACCTACTAGGCCTACTCCCATACACATTTACACCAACAACACAACTCTCCATAAACATAGCCCTCGCTATCCCCCTATGACTGATGACTGTACTAACAGGCATGCGAACTCAACCAACAACCTCCTTAGCCCACCTTCTTCCAGAAGGAACCCCCACCCCACTAATCCCAGTCCTCATTATAATTGAAACAATTAGCCTATTAATTCGACCAATCGCATTGGGTGTACGATTAACAGCCAACCTAACCGCAGGCCACCTACTAATTCAATTAATCTCCATTGCTACACTAAACCTCTGACCCATCATACCACCCCTCAGCCTATTAACACTAACAACCCTGATCCTGCTCCTCCTACTAGAATTCGCCGTAGCCATAATCCAAGCATACGTCTTCGTCCTCCTACTCTCCCTATACCTACAAGAAAACACATAAATGCCCCAGCTAAACCCAGAACCCTGACTAACAGTCCTTCTAACCACCTGAATCTTCTTTATCGCCATTCTCCAGCCTAAAATCGCCACACTAACCGCCACAAACAACCCCACCCCACACAAACCACAGACCACC-AAAACATGACACTGGCCATGAACACAAACCTATT--------------ATGACCCACCAACTACGAAAATCCCACCCACTCCTCAAACTCTTCAACCACTCCCTCATTGACCTCCCAACACCCTCAAACATCTCCGCCTGGTGAAACTTTGGATCACTACTGGGCCTGACCCTCCTAATCCAAATTCTAACAGGAGTCTTCCTAATAATACACTTCTCATCAAGTGACACCACGGCCTTCTCATCCGTCGCCTACACCTCCCGCGAAGTCTGATTCGGATGACTTATCCGAAACCTCCACACAAACGGAGCCTCACTCTTCTTCATGTTTATTTTCCTCCACATCGGACGGGGCCTGTACTACGCCTCCTACACCCACGAAAAGACATGAGCTGTAGGAGTTATTATACTCTTCCTACTAATAGCTACAGCATTCATGGGTTATGTCCTCCCCTGAGGACAAATATCATTCTGAGGGGCAACCGTAATTACAAACCTGCTGTCTGCCACCCCATACATCGGAGACACCATCGTGCCATGAATTTGGGGGGGACCCTCCGTAGACAACGCAACACTCACACGCTTTACCGCCCTTCACTTCCTCCTCCCGTTTACCCTCCTAGCCCTGCTCATCACCCACCTGATCTTTCTACACGAACGGGGGTCCCTCAACCCCCTCGGACTAAGCCAAAACGCCGACAAAATCCCATTCCACCCCTACTACACACTAAAAGATGCTCTAGGAGCAACACTAGCCGCCTCCCTACTACTCACCTTCGCCCTATACCTCCCAACCCTACTAGGCGACCCAGAAAACTTCACCCCAGCAAACCCCATAACCACCCCATCACACATCAAACCTGAATGATACTTCCTATTTGCCTATGCCATCCTACGATCTATCCCAAACAAGCTCGGAGGCGTACTAGCCATATTCTCCTCCATCTTTATCTTATTCCTAGTGCCTGCCCTTCACACAGCCAAACAACAGCCCATATCCGTCCGCCCCTTATCCCAACTCCTATTCTGAGCCCTCATCCTAGACTTCTTTGTACTTACATGAATTGGGGGCCAACCAGTAAACCCCCCATTCATCCTAATGGGCCAAATCGCCTCCTCACTCTTCTTCATCACCATCCTCATCCTCATACCCACACTAGGGTCCCTAGAGAACAAAATAACC------TAA-----------------------ATGTTCATCAACCGCTGATTTTTCTCCACAAACCACAAAGACATCGGCACCCTGTACTTCATCTTCGGGGCCTGATCCGGAATAGTAGGCACAGCACTCAGCCTTCTCATCCGAACAGAACTAAGCCAACCAGGACCCCTACTAGGAGACGACCAAATCTACAATGTAATCGTCACTGCCCACGCCTTTATTATAATCTTTTTTATAGTAATACCCGTCATGATCGGCGGATTCGGAAACTGACTCCTGCCCTTAATAATTGGAGCCCCAGACATAGCATTCCCGCGAATAAACAACATAAGCTTCTGACTACTCCCCCCATCCTTCACACTGCTGCTCGCCTCTTCCTGCATTGAAGCGGGGGCCGGAACAGGATGAACCGTCTATCCCCCCCTAGCTGGAAACATAGCCCACGCCGGACCATCAGTAGACCTAACCATCTTCTCCCTACACCTTGCCGGAGTATCTTCCATCCTAGGCGCAATCAACTTCATCACAACAGCCATTAACATAAAACCCCCAGCCATGTCCCAATACCAAACACCCCTATTTGTTTGATCGGTCTTAATCACAGCCGTACTACTCTTACTCTCCCTCCCAGTACTAGCTGCCGGAATTACTATACTACTCACAGACCGAAACCTAAACACAACCTTCTTTGACCCCGCAGGAGGGGGGGACCCCATCCTATACCAGCACCTTTTCTGATTCTTCGGCCACCCCGAAGTCTACATCCTCATCCTACCTGGATTTGGAATAGTCTCACACGTCGTCACCTTCTACTCAAACAAAAAAGAACCATTCGGCTACATAGGAATAGCATGAGCCATAATATCTATCGGATTCCTAGGATTCATCGTATGGGCCCACCACATATTCACAGTCGGAATAGACGTCGACACACGAGCATACTTTACTACTGCCACAATAGTTATTGCCATCCCCACCGGGGTAAAAGTGTTTAGTTGACTAGCAACCATCTACGGGGGCATCGTCAACTGACAAGCCCCAATACTATGAGCACTAGGCTTCATCTTCTTATTCACCGTAGGTGGCCTTACTGGAATCGTCCTAGCCAACTCCTCCCTAGACATCGTTCTCCACGACACCTACTACGTAGTCGCCCACTTCCACTACGTATTATCAATAGGAGCAGTCTTTGCCATCATATGCGGATTTACCCACT-GATTCCCACTA-TTCACAGGATTTACCCTCCACCCAACATGAACAAAAA-TCCAATTCACAATTATATTCTTAGGAGTAAACCTTACCTTCTTCCCACAACACTTCCTAGGGCTATCTGGAATACCTCGACGATACTCTGACTACCCAGACGCATACACCATCTGAAATCTAACATCATCAATTGGATCCCTAATCTCCCTAACCGCTGTCATCCTACTAATGTTCATTGTATGAGAAGCATTTTCTTCCAAACGAAAAACAACCACACCTGAGATAACAACAACCAACATCGAGTGACTTAACAACTCCCCACCACCCCACCACACATACGAAGAACCAGTTTTTACTGTAACACACAAACCAACCGACCTC-----------------------------CTAAACTATGGCCAACCCCACACATCTGGGCTTCCAAGACGCAATATCCCCCCTCATAGAAGAGCTATTATACTTCCATGACCACACTCTAATAATCCTCATCCTAATCAGCTCCCTTGTATTCTACACAATCTCCGCCCTGCTCCTCCAAAAACTCTATCACTCAAACACCTCCGACGTCCAAGAGGTAGAAATAGTATGAACCATCCTACCAGCCATCGTCTTAATTTCAATCGCCCTCCCCTCTCTGCGAGCCCTTTACCTTATAGACGAAACCAACAACCCATGCCTGACTATCAAAGCAACCGGACACCAGTGATACTGATCCTACGAATACACTGACTTCTCCACAGTAGAATTTGACTCCTACATGACACCCCCACAAGATCTCTCTCCAGGTCACCTCCGCCTCCTAGAAGTTGACCACCGCATAATTACCCCAATAAACTCAACAATTCGGATATTAATCACAGCAGAAGATGTCCTGCACTCATGGGCAATTCCATCCATCGGAACAAAAATAGACGCAGTCCCAGGACGCCTAAACCAAGTCATAATTACACTAACCAACCCTGGCGTATTCTACGG-CCAATGCTCCGAAATCTGTGGAGCAAACCACAGCTTCATACCCATCGTAATAGAAACCACCCCATTAAACCACTTTCAACTCTGACTAGAAGACCACACTACCTCATATGTCCCACCAAACCCACCCATTCCATATAGTACACCCAAGCCCCTGACCCCTAGCCGGCGCCATAGCCGCCATATTACTAACAACCGGCCTAACCTTTTGATTTCACTACAACTCTTGCCCCCTCCTCCTACTAGGCCTGACCTCAGCCCTACTTGTAATATTCCAATGATGACGAGACATTGTACGAGAAAGCACCTTCCTAGGACACCACACACCCCCAGTACAAAAAGGATTGCGCTACGGCATAATTCTCTTTATTACATCTGAAGTCTTCTTTTTCCTAGGCTTCTTCTGAGCATTTTACCACTCAAGCCTCTCCCCAACCCCAGAGCTAGGAGGACAATGACCCCCTACCGGAATCACCCCCCTCGACCCCTTTGAAGTCCCCCTCCTTAACACAGCCGTCCTACTCGCCTCCGGAGTAACAGTAACATGGGCCCACCACAGCCTAATAAACACCCACCGCATGCAAGCAATCCACGCTCTCGCACTCACTGTACTTCTCGGATTCTACTTCACCACCCTTCAAGCCATAGAATACTATGAGGCCCCCTTCACTATCGCAGATAGCACCTACGGATCAACATTCTTTGTTGCAACAGGCTTTCATGGCCTCCACGTCATCATCGGCTCAATCTTCCTCACAGTCTGCCTATATCGACAAACAAAATACCACTTCACATCCAACCACCACTTCGGATTCGAAGCCGCCGCCTGATACTGACACTTCGTAGACATAATCTGACTATTCCTCTACATCTCAATCTACTGATGAGGCTCATATCGACTCCCTCACCATCCTCCCCCCAATCTTATTTATTGCCTCCGTCCTAATTGCAGTCGCCTTCTTAACAGCCCTAGAACGAAAAGTTATTGGCTACATTCAATTACGAAAAGGACCAAACATTGTTGGCCCCATCGGCCTGCTCCAACCCCTCGCAGATGGATTCAAACTTATTATTAAAGAACTGACCCTCCCCCTACTTGCCACACCAACCCTATTCATCCTCGCCCCGGCAGCAGCCCTAATACTAGCCCTCACCATGTGGTCCCCCCTTCCAATACCATTCCCCCTCACAGACCTCAACCTAGGCCTGCTTTTTCTACTAGCCATATCAAGCCTCATGGTCTACTCACTTCTCTGATCCGGGTGGGCCTCAAACTCAAAATACGCCCTAATAGGGGCCCTCCGAGCAGTAGCCCAAACAATCTCCTACGAAGTCACACTAGCCATTATTGTACTCTCCATTATCTTGCTCTGCGGGGGGTTCTCACTACACGCCCTCACCACCACACAAGAGCCCCTATATCTTGCACTGCCTGCATGGCCCCTAATAATAACGTGATATATCTCCACACTAGCAGAAACAAACCGCGCCCCATTCGACTTAACAGAGGGCGAATCAGAACTCGTATCTGGGTTCAATGTTGAATACGGCGCAAGCCCCTTCGCACTGTTCTTCCTAGCCGAATACGCCAACATCATGTTAATAAACACCCTGACCGTCATCCTCTTCCTCAGCCCATCACCCCAAACCCACCCTCCAACGCTATTCACCATCACCCTAATAACCAAAACCCTCTTACTGACTACAACCTTTCTATGAGTCCGAGCATCCTACCCACGATTTCGCTATGATCAACTCATACACCTCCTATGAAAAAGCTTCCTCCCCCTAACATTAGCCCTCTGCCTATGACACACATCACTACCAGTCTCAATATTTGGACTTCCCCCAACAACCTAGATGTCCCTCTCATTACCCCTCATCCTAACAACAATGGCCCCAGCAACACTCATCTTCCTCATATCATCCCACCTAGTACTAATGTGAATCGCACTAGAACTCAACACACTGACAATCCTTTACATAATTGCCAACAAATCCCACCCCCGAGCCATTGAAGCCACCATCAAATACTTCCTCACACAAGCACTAGCATCCACATTAATTATCCTTTCCGGAACTATCAACTACGAGATAACAGGAAGCTGGGAAATCACAGAAATAACTAGCCCAACCCCCTTAACCATACTCACCCTCGCCCTATCCATCAAGCTAGGATTAGTCCCATTTCACTTCTGAGTGCCAGAAGTCCTTCAAGGAATACCCACACTCCCCGCAATCTTCCTACTTACATGACAAAAAGTAGGCCCACTAATTATGTTCTTCTTAATCAGCCCCTTTATCAACCATAATTTAGTCTCCACAATGGCCATTATGTCCTCCACTGTTGCAGGGTGGCTGGGGCTCAACCAAACCCAGGTACGAAAGCTAATAGCAATGTCCTCCATCGCCCAAATAGCATGAATAATTGTCGTTATTAAATACGCACCCTCCCTAACAGTCCTCTCTTTCTACGTGTATTCAATAACCGTCTCCGCCGCACTACTAATGCTAAACAAGCTATCTGTAACCACCATAAAATCACTTGCCACTTCCTTCTCAAAAAACCCAATTGCCACCTTACTCCTAATAGCCTCCATCCTCTCACTATCCGGCCTTCCCCCCCTGGCCGGCTTCACACCTAAATGATTAATAATCAACCAGCTTGTTGCAGAAAAGGCAGTCTGAACCGCACTTCTCATACTAGCATCCTCACTCTTGACCCTGTTCTTCTACCTTCGCATGTGATACTACACCATCTCCACCCTGCCCCCAAACACCTCAAACATATCCCGCCCTTGACGAAAACCCACCTCCAAAAGCAACCTCACCGCCAACACCCTCGCTACAGCTGCCTCCACCCTCCTACTATGCACTACACTCATAAAAGCAGCCACCAAAAG------------------AA----TAGACCTCCTCATTATATTCTCAATAGCCTCCATCACTGCAACAATCCTGATTATCTCAAACCTCACAATAGCCGAAGCATCGCCCGACCCTGAAAAACTATCCCCATACGAATGTGGATTTGACCCCCTAGGGTCTGCTCGACTTCCACTATCAATCCGGTTCTTCATGATCGCTATCTTATTCTTACTTTTCGACCTTGAAATCGCCATCTTACTCCCACTCACGTGATCCACCCACACCTTTCACCCAACAAAAACCATCACATGTATTATCGCAATCTTCCTACTACTATTCATCGGTCTGATATACGAATGACTCCATGGCGGCCTAGAATGAGCAGAATAGATGCTAAAAATCCTGCTACCCACGGCCATGGTTGGCCCAA---CAACCCTCACCCCAAACAAGACCATCTGACTATCCCCAACAACTTGCGCCACTGCTGTAATAATTATCCCCCTACTAACCCTAAACCCCTCAGATACTTTAACAAACCCCAGCAACCAAGCCCTCGGTAGCGACCAATATTCAAGCCCCCTGATAATACTATCCTGCTGACTCCTCCCCCTGATGCTCATGGCCAGCCAAAACGCAATATCCACAACCCTGGCCCCCCAAATCCAAATATTCATCTCCATTCTTGCAACACTCCAACTCACCCTGCTCATGGCATTCATGGCCCTAGACTTAATACTATTCTATATTATATTTGAAACCACCCTACTTCCAACCCTAGTAACCATCTCCCGATGAGGCGCCCGACCAGAACGTCTAAATGCCGGAGCCTATTTCCTATTCTACACTATTACCAGCTCAATCCCCCTAATAATTAGCATTCTAATAATCCACAATCTCATCGGCACCTTGTCCCTCCCAATCCTACAACTAACCCCAATAACGAACCACCCCCCCTGAACAAATACACTATTCTGACTCTCAACACTTGTAGCCTTCCTGGTAAAAATCCCAATCTACGGACTACACCTCTGACTCCCCAAAGCTCACGTCGAAGCCCCAATTGCAGGATCAATAGTACTGGCCGCAGTACTTCTAAAGCTGGGAGGATACGGGATGCTACGAGTAACAAACCTGCTCACCGCGCAGGTTAATACTACCTACCTCCCCCTACTAACCCTAGCACTCTGAGGGGCACTAACAACAAGCCTCATCTGCCTACGACAAACAGACCTAAAATCCCTAATCGCCTATTCATCGGTTAGTCACATAGCCCTGGTAACAGCCACGATCCTCGCCCGAGACCACCTTACCCCAACAGGATCCATAATCCTAATAGTAGCCCACGGACTAACCTCCTCCATACTATTCTGTCTAGCAAACTTCAATTATGAACGAACCCACACCCGAACACTCATCACCACACAAGGCATACAACTCACCTCCCCCGCCCTCACAGCTTGGTGGTTCCTAGCCTGCTCAATAAACATAGCCCTCCCCCCAACATTCAACCTAACCGGCGAACTAACCGTTATTACATCGCTATTTAACTGACTAGACATTACAGTTATCCTAACAGGACTGAGTGCCTTCATAACGGCAGTCTACACCTTATACATATTCTCAGCCACCCAACAATGAACACTACCCACTAATGCCACCCTATTACCTCCAACCCAAACCCGAGAACACCTCCTGATACTCCTCCACCTCCTCCCCTCTGCGGGCCTTACCACCAACCCAAAACTCACCTCCGCCCAATA---AATTACCCCCACAAATACACTTTTTTTGTTCTCCTTCTCTCTTTGCATCATTGGCCTAATTACCCACCACAACCACCTCCTCTCAACACTCCTCTGCCTTGAAGGGATGGCACTCTCTATCTTCTTAGCCGTCACAATATCCTCACTCCAATCACACCCCTCATCATTCATCCTCCCCCTAACAATCCTGACCCTCTCTGCCTGCGAAGCAGGCACCGGCCTCGCCCTACTAGTCGCCTCCGCCCGAACACACAACACCGCCAACCTTAAAAACCTAAACCTCCTTCAATGCTAAAT----GTCCCACACGACACTCCTCCAAACATTCTCCGCCATCCCCTTTCTAATCCTATTACTGGCAACCCTTCCCCTAGCCCATAAAACCAACAACACACCAGCCAAATCCAAAACAATAATAGCCAAACTCGCTTTCTTCACAAGCCTCCCCCCTCTCTTTTTAGTGGTGCTAAATAACAGCCCCGCCCTGTCACACCACCTATCTTTATTCCACCTAGAATCCCTCAACCTCCCCATCACCCTAAAAATTGACACTTATTCAACCTTCTTCATCCCAACAGCATTATTCATCACATGGTCAATTATAGAATTCTCCAAATCGTACATAGACTCAGACCCCAAAATTACCAGCTTCTTCAACCACCTCCTAGTATTTATCCTAATAATAATAATCCTAGTAAGCGCTAGCAACCTATTCCACCTCTTTATCGGCTGAGAAGGGGTAGGCATTATATCGTACCTACTCATCAACTGATGATCCTTCCGAGCAAACGCCAACAAAGCAGCGCTCCAGGCTATCATCTACAACCGACTATCAGACATTGGAATCCTCACCACACTGACATGAATAACCACCCACAATCTCACCTTAGACATCACAAACCTCTCC---CCCCCAAACTACCCACTAATCCCAGCCGCAGCCATCATCCTAGCAGCAGCGGGAAAATCAGCCCAAATTGGCCTCCACCCCTGACTCCCAGCAGCAATAGAAGGGCCAACACCAGTTTCCGCCCTACTCCACTCAAGCACCATAGTAGTAGCAGGCGTCTTCTTACTAATCCGAACTTCTCCAATCATCTATAGCAGCCAAACAACAACTACAGCCTGCCTAATTCTAGGAGCAATCACCTCTCTATTCGCAGCCGCATGCGCTTTCGCCCAAAATGACATAAAAAAAATCATCGCATTCTCAACCTCAAGTCAACTTGGACTAATAGTAACCACAATTGGACTTAAACAACCCGAACTCGCATTCCTACACATCCTAATGCACGCCTTTTTCAAAGCAATACTCTTCCTGTGCGCAGGATCAATCATCCACAGCCTCAATAACGAACAAGACTTCCGAAAAATAGGAAACCTCAAAAAAGCAATACCAATTACCACCTCTTGCCTTACCATCGGAGTACTAGCCCTCACCGGCACGCCCTTCCTCTCCGGATTCTACTCCAAAGATGCCATTATTGAATCACTAAACACCTCACATATTAACGCCTGAGCCCTTGCCATCACCCTACTCGCCACCTCCTTCACTGCAATCTATAGCCTCCGCATAGTCTACTTCACCCTACTAAATACCAATCGACTAGCAACCACGAGCCCGATAAACGAAACCCCAAAAACCACCAACCCTATTCTCCGTTTAGCCATTGGAAGTGTAATCACAGGACTATTGACCTTAAATTTCATACTACCAACCCACACCCCCCAACTAACTATACCAACCACAATCAAACTTATAGCCCTAACCATCACAGCAATTGGCATACTCACTGCAATGGCCCTAACCCTCACCACCGACAAATTCCCACCATCCACAAAAGATACCCAACCACCCCCGCTAACAAAACTAATATTCTTCAACCCAATTATCCACCACCTATTCTCCTCCACCACCCTGCACATCAGCCAAAAGCTCTCCACACACCTAACAGACCAACTATGATATGAAACCCTTGGGCCAAAAACAACGGCCAGCCTGCAAACCATGATAACCAAAACACTAACCCCCTACCACAAAGCAAAAATAAAAAAATACCTCAAAACCTTCCTCTGAGCCATCACCATCACCCTCCTCCTC---------TGAA---------GG---------------------------------ATGGAATTTACATT---ATTTTTGGGTTGTCTGGTGTTGGCGTGTGCGGTGATGGTGGGGGCGAATTCTGGGATTCATTTTGCTGTGTTGAGCTTGGTATTTATGGCTGTGTTTGGTAGCTGTTTGGTGGTGATTGAGGGTGGGAGTTTTATGCCGTTGGTGGTGTTGTTGGTTTATTTGGGGGGGTTGTTGGTAGTGTTTGCTTTTTGCGTTGGGTTTACTGACGATCCGTTTG---------GGATGGTGGGGGCTTCTAAGGGGTTTGTGCTTTTTTGTTGGATTGGTCTTCTTGTTGGGGGGGTTTGTATGTGTGGACATTTGTGGGGTTGGTGGGTCGTTGGGGTGAGCGGGTTGGTGGATGTAGAGGTTGTTGGGGGTGGGGCGGCTGATGAGTTGTTGGGGGTGGGTTTAATGTATTCAAGTGGGTGGGGTTTTGTTGTGATTTGTGGTTGGGCCTTGCTGGTTACGTTGCTCGTGATTACGGGGCTTGTTCGGGGTTGTTGTTGGGGGTCACTTCGTCCCTTCAGA

*Paleosuchus palpebrosus 3*

------------ATGAACACAAACCTATTCGACCAATTCCTAATCCCCAACCTCTTGGGAACCTCCCTACTAACACCCGCCCTACTAATAACCATTGCCCTCGTGCTAAACCCCCAAAATCTATGACTCCAACACCCAACAGCAACAATTAAAACCTGACTCATCAACCAAATTACCAAACAAATCATAACCCCAATCAACAAGTCAGGACATAAGCACTCCCTAACCCTTATCTCCATCCTAGTTCTCCTCCTCCTCACAAACCTACTAGGCCTACTCCCATACACATTTACACCAACAACACAACTCTCCATAAACATAGCCCTCGCTATCCCCCTATGACTGATGACTGTACTAACAGGCATGCGAACTCAACCAACAACCTCCTTAGCCCACCTTCTTCCAGAAGGAACCCCCACCCCACTAATCCCAGTCCTCATTATAATTGAAACAATTAGCCTATTAATTCGACCAATCGCATTGGGTGTACGATTAACAGCCAACCTAACCGCAGGCCACCTACTAATTCAATTAATCTCCATTGCTACACTAAACCTCTGACCCATCATACCACCCCTCAGCCTATTAACACTAACAACCCTGATCCTGCTCCTCCTACTAGAATTCGCCGTAGCCATAATCCAAGCATACGTCTTCGTCCTCCTACTCTCCCTATACCTACAAGAAAACACATAAATGCCCCAGCTAAACCCAGAACCCTGACTAACAGTCCTTCTAACCACCTGAATCTTCTTTATCGCCATTCTCCAGCCTAAAATCGCCACACTAACCGCCACAAACAACCCCACCCCACACAAACCACAGACCACC-AAAACATGACACTGGCCATGAACACAAACCTATT--------------ATGACCCACCAACTACGAAAATCCCACCCACTCCTCAAACTCTTCAACCACTCCCTCATTGACCTCCCAACACCCTCAAACATCTCCGCCTGGTGAAACTTTGGATCACTACTGGGCCTGACCCTCCTAATCCAAATTCTAACAGGAGTCTTCCTAATAATACACTTCTCATCAAGTGACACCACGGCCTTCTCATCCGTCGCCTACACCTCCCGCGAAGTCTGATTCGGATGACTTATCCGAAACCTCCACACAAACGGAGCCTCACTCTTCTTCATGTTTATTTTCCTCCACATCGGACGGGGCCTGTACTACGCCTCCTACACCCACGAAAAGACATGAGCTGTAGGAGTTATTATACTCTTCCTACTAATAGCTACAGCATTCATGGGTTATGTCCTCCCCTGAGGACAAATATCATTCTGAGGGGCAACCGTAATTACAAACCTGCTGTCTGCCACCCCATACATCGGAGACACCATCGTGCCATGAATTTGGGGGGGACCCTCCGTAGACAACGCAACACTCACACGCTTTACCGCCCTTCACTTCCTCCTCCCGTTTACCCTCCTAGCCCTGCTCATCACCCACCTGATCTTTCTACACGAACGGGGGTCCCTCAACCCCCTCGGACTAAGCCAAAACGCCGACAAAATCCCATTCCACCCCTACTACACACTAAAAGATGCTCTAGGAGCAACACTAGCCGCCTCCCTACTACTCACCTTCGCCCTATACCTCCCAACCCTACTAGGCGACCCAGAAAACTTCACCCCAGCAAACCCCATAACCACCCCATCACACATCAAACCTGAATGATACTTCCTATTTGCCTATGCCATCCTACGATCTATCCCAAACAAGCTCGGAGGCGTACTAGCCATATTCTCCTCCATCTTTATCTTATTCCTAGTGCCTGCCCTTCACACAGCCAAACAACAGCCCATATCCGTCCGCCCCTTATCCCAACTCCTATTCTGAGCCCTCATCCTAGACTTCTTTGTACTTACATGAATTGGGGGCCAACCAGTAAACCCCCCATTCATCCTAATGGGCCAAATCGCCTCCTCACTCTTCTTCATCACCATCCTCATCCTCATACCCACACTAGGGTCCCTAGAGAACAAAATAACC------TAA-----------------------ATGTTCATCAACCGCTGATTTTTCTCCACAAACCACAAAGACATCGGCACCCTGTACTTCATCTTCGGGGCCTGATCCGGAATAGTAGGCACAGCACTCAGCCTTCTCATCCGAACAGAACTAAGCCAACCAGGACCCCTACTAGGAGACGACCAAATCTACAATGTAATCGTCACTGCCCACGCCTTTATTATAATCTTTTTTATAGTAATACCCGTCATGATCGGCGGATTCGGAAACTGACTCCTGCCCTTAATAATTGGAGCCCCAGACATAGCATTCCCGCGAATAAACAACATAAGCTTCTGACTACTCCCCCCATCCTTCACACTGCTGCTCGCCTCTTCCTGCATTGAAGCGGGGGCCGGAACAGGATGAACCGTCTATCCCCCCCTAGCTGGAAACATAGCCCACGCCGGACCATCAGTAGACCTAACCATCTTCTCCCTACACCTTGCCGGAGTATCTTCCATCCTAGGCGCAATCAACTTCATCACAACAGCCATTAACATAAAACCCCCAGCCATGTCCCAATACCAAACACCCCTATTTGTTTGATCGGTCTTAATCACAGCCGTACTACTCTTACTCTCCCTCCCAGTACTAGCTGCCGGAATTACTATACTACTCACAGACCGAAACCTAAACACAACCTTCTTTGACCCCGCAGGAGGGGGGGACCCCATCCTATACCAGCACCTTTTCTGATTCTTCGGCCACCCCGAAGTCTACATCCTCATCCTACCTGGATTTGGAATAGTCTCACACGTCGTCACCTTCTACTCAAACAAAAAAGAACCATTCGGCTACATAGGAATAGCATGAGCCATAATATCTATCGGATTCCTAGGATTCATCGTATGGGCCCACCACATATTCACAGTCGGAATAGACGTCGACACACGAGCATACTTTACTACTGCCACAATAGTTATTGCCATCCCCACCGGGGTAAAAGTGTTTAGTTGACTAGCAACCATCTACGGGGGCATCGTCAACTGACAAGCCCCAATACTATGAGCACTAGGCTTCATCTTCTTATTCACCGTAGGTGGCCTTACTGGAATCGTCCTAGCCAACTCCTCCCTAGACATCGTTCTCCACGACACCTACTACGTAGTCGCCCACTTCCACTACGTATTATCAATAGGAGCAGTCTTTGCCATCATATGCGGATTTACCCACT-GATTCCCACTA-TTCACAGGATTTACCCTCCACCCAACATGAACAAAAA-TCCAATTCACAATTATATTCTTAGGAGTAAACCTTACCTTCTTCCCACAACACTTCCTAGGGCTATCTGGAATACCTCGACGATACTCTGACTACCCAGACGCATACACCATCTGAAATCTAACATCATCAATTGGATCCCTAATCTCCCTAACCGCTGTCATCCTACTAATGTTCATTGTATGAGAAGCATTTTCTTCCAAACGAAAAACAACCACACCTGAGATAACAACAACCAACATCGAGTGACTTAACAACTCCCCACCACCCCACCACACATACGAAGAACCAGTTTTTACTGTAACACACAAACCAACCGACCTC-----------------------------CTAAACTATGGCCAACCCCACACATCTGGGCTTCCAAGACGCAATATCCCCCCTCATAGAAGAGCTATTATACTTCCATGACCACACTCTAATAATCCTCATCCTAATCAGCTCCCTTGTATTCTACACAATCTCCGCCCTGCTCCTCCAAAAACTCTATCACTCAAACACCTCCGACGTCCAAGAGGTAGAAATAGTATGAACCATCCTACCAGCCATCGTCTTAATTTCAATCGCCCTCCCCTCTCTGCGAGCCCTTTACCTTATAGACGAAACCAACAACCCATGCCTGACTATCAAAGCAACCGGACACCAGTGATACTGATCCTACGAATACACTGACTTCTCCACAGTAGAATTTGACTCCTACATGACACCCCCACAAGATCTCTCTCCAGGTCACCTCCGCCTCCTAGAAGTTGACCACCGCATAATTACCCCAATAAACTCAACAATTCGGATATTAATCACAGCAGAAGATGTCCTGCACTCATGGGCAATTCCATCCATCGGAACAAAAATAGACGCAGTCCCAGGACGCCTAAACCAAGTCATAATTACACTAACCAACCCTGGCGTATTCTACGG-CCAATGCTCCGAAATCTGTGGAGCAAACCACAGCTTCATACCCATCGTAATAGAAACCACCCCATTAAACCACTTTCAACTCTGACTAGAAGACCACACTACCTCATATGTCCCACCAAACCCACCCATTCCATATAGTACACCCAAGCCCCTGACCCCTAGCCGGCGCCATAGCCGCCATATTACTAACAACCGGCCTAACCTTTTGATTTCACTACAACTCTTGCCCCCTCCTCCTACTAGGCCTGACCTCAGCCCTACTTGTAATATTCCAATGATGACGAGACATTGTACGAGAAAGCACCTTCCTAGGACACCACACACCCCCAGTACAAAAAGGATTGCGCTACGGCATAATTCTCTTTATTACATCTGAAGTCTTCTTTTTCCTAGGCTTCTTCTGAGCATTTTACCACTCAAGCCTCTCCCCAACCCCAGAGCTAGGAGGACAATGACCCCCTACCGGAATCACCCCCCTCGACCCCTTTGAAGTCCCCCTCCTTAACACAGCCGTCCTACTCGCCTCCGGAGTAACAGTAACATGGGCCCACCACAGCCTAATAAACACCCACCGCATGCAAGCAATCCACGCTCTCGCACTCACTGTACTTCTCGGATTCTACTTCACCACCCTTCAAGCCATAGAATACTATGAGGCCCCCTTCACTATCGCAGATAGCACCTACGGATCAACATTCTTTGTTGCAACAGGCTTTCATGGCCTCCACGTCATCATCGGCTCAATCTTCCTCACAGTCTGCCTATATCGACAAACAAAATACCACTTCACATCCAACCACCACTTCGGATTCGAAGCCGCCGCCTGATACTGACACTTCGTAGACATAATCTGACTATTCCTCTACATCTCAATCTACTGATGAGGCTCATATCGACTCCCTCACCATCCTCCCCCCAATCTTATTTATTGCCTCCGTCCTAATTGCAGTCGCCTTCTTAACAGCCCTAGAACGAAAAGTTATTGGCTACATTCAATTACGAAAAGGACCAAACATTGTTGGCCCCATCGGCCTGCTCCAACCCCTCGCAGATGGATTCAAACTTATTATTAAAGAACTGACCCTCCCCCTACTTGCCACACCAACCCTATTCATCCTCGCCCCGGCAGCAGCCCTAATACTAGCCCTCACCATGTGGTCCCCCCTTCCAATACCATTCCCCCTCACAGACCTCAACCTAGGCCTGCTTTTTCTACTAGCCATATCAAGCCTCATGGTCTACTCACTTCTCTGATCCGGGTGGGCCTCAAACTCAAAATACGCCCTAATAGGGGCCCTCCGAGCAGTAGCCCAAACAATCTCCTACGAAGTCACACTAGCCATTATTGTACTCTCCATTATCTTGCTCTGCGGGGGGTTCTCACTACACGCCCTCACCACCACACAAGAGCCCCTATATCTTGCACTGCCTGCATGGCCCCTAATAATAACGTGATATATCTCCACACTAGCAGAAACAAACCGCGCCCCATTCGACTTAACAGAGGGCGAATCAGAACTCGTATCTGGGTTCAATGTTGAATACGGCGCAAGCCCCTTCGCACTGTTCTTCCTAGCCGAATACGCCAACATCATGTTAATAAACACCCTGACCGTCATCCTCTTCCTCAGCCCATCACCCCAAACCCACCCTCCAACGCTATTCACCATCACCCTAATAACCAAAACCCTCTTACTGACTACAACCTTTCTATGAGTCCGAGCATCCTACCCACGATTTCGCTATGATCAACTCATACACCTCCTATGAAAAAGCTTCCTCCCCCTAACATTAGCCCTCTGCCTATGACACACATCACTACCAGTCTCAATATTTGGACTTCCCCCAACAACCTAGATGTCCCTCTCATTACCCCTCATCCTAACAACAATGGCCCCAGCAACACTCATCTTCCTCATATCATCCCACCTAGTACTAATGTGAATCGCACTAGAACTCAACACACTGACAATCCTTTACATAATTGCCAACAAATCCCACCCCCGAGCCATTGAAGCCACCATCAAATACTTCCTCACACAAGCACTAGCATCCACATTAATTATCCTTTCCGGAACTATCAACTACGAGATAACAGGAAGCTGGGAAATCACAGAAATAACTAGCCCAACCCCCTTAACCATACTCACCCTCGCCCTATCCATCAAGCTAGGATTAGTCCCATTTCACTTCTGAGTGCCAGAAGTCCTTCAAGGAATACCCACACTCCCCGCAATCTTCCTACTTACATGACAAAAAGTAGGCCCACTAATTATGTTCTTCTTAATCAGCCCCTTTATCAACCATAATTTAGTCTCCACAATGGCCATTATGTCCTCCACTGTTGCAGGGTGGCTGGGGCTCAACCAAACCCAGGTACGAAAGCTAATAGCAATGTCCTCCATCGCCCAAATAGCATGAATAATTGTCGTTATTAAATACGCACCCTCCCTAACAGTCCTCTCTTTCTACGTGTATTCAATAACCGTCTCCGCCGCACTACTAATGCTAAACAAGCTATCTGTAACCACCATAAAATCACTTGCCACTTCCTTCTCAAAAAACCCAATTGCCACCTTACTCCTAATAGCCTCCATCCTCTCACTATCCGGCCTTCCCCCCCTGGCCGGCTTCACACCTAAATGATTAATAATCAACCAGCTTGTTGCAGAAAAGGCAGTCTGAACCGCACTTCTCATACTAGCATCCTCACTCTTGACCCTGTTCTTCTACCTTCGCATGTGATACTACACCATCTCCACCCTGCCCCCAAACACCTCAAACATATCCCGCCCTTGACGAAAACCCACCTCCAAAAGCAACCTCACCGCCAACACCCTCGCTACAGCTGCCTCCACCCTCCTACTATGCACTACACTCATAAAAGCAGCCACCAAAAG------------------AA----TAGACCTCCTCATTATATTCTCAATAGCCTCCATCACTGCAACAATCCTGATTATCTCAAACCTCACAATAGCCGAAGCATCGCCCGACCCTGAAAAACTATCCCCATACGAATGTGGATTTGACCCCCTAGGGTCTGCTCGACTTCCACTATCAATCCGGTTCTTCATGATCGCTATCTTATTCTTACTTTTCGACCTTGAAATCGCCATCTTACTCCCACTCACGTGATCCACCCACACCTTTCACCCAACAAAAACCATCACATGTATTATCGCAATCTTCCTACTACTATTCATCGGTCTGATATACGAATGACTCCATGGCGGCCTAGAATGAGCAGAATAGATGCTAAAAATCCTGCTACCCACGGCCATGGTTGGCCCAA---CAACCCTCACCCCAAACAAGACCATCTGACTATCCCCAACAACTTGCGCCACTGCTGTAATAATTATCCCCCTACTAACCCTAAACCCCTCAGATACTTTAACAAACCCCAGCAACCAAGCCCTCGGTAGCGACCAATATTCAAGCCCCCTGATAATACTATCCTGCTGACTCCTCCCCCTGATGCTCATGGCCAGCCAAAACGCAATATCCACAACCCTGGCCCCCCAAATCCAAATATTCATCTCCATTCTTGCAACACTCCAACTCACCCTGCTCATGGCATTCATGGCCCTAGACTTAATACTATTCTATATTATATTTGAAACCACCCTACTTCCAACCCTAGTAACCATCTCCCGATGAGGCGCCCGACCAGAACGTCTAAATGCCGGAGCCTATTTCCTATTCTACACTATTACCAGCTCAATCCCCCTAATAATTAGCATTCTAATAATCCACAATCTCATCGGCACCTTGTCCCTCCCAATCCTACAACTAACCCCAATAACGAACCACCCCCCCTGAACAAATACACTATTCTGACTCTCAACACTTGTAGCCTTCCTGGTAAAAATCCCAATCTACGGACTACACCTCTGACTCCCCAAAGCTCACGTCGAAGCCCCAATTGCAGGATCAATAGTACTGGCCGCAGTACTTCTAAAGCTGGGAGGATACGGGATGCTACGAGTAACAAACCTGCTCACCGCGCAGGTTAATACTACCTACCTCCCCCTACTAACCCTAGCACTCTGAGGGGCACTAACAACAAGCCTCATCTGCCTACGACAAACAGACCTAAAATCCCTAATCGCCTATTCATCGGTTAGTCACATAGCCCTGGTAACAGCCACGATCCTCGCCCGAGACCACCTTACCCCAACAGGATCCATAATCCTAATAGTAGCCCACGGACTAACCTCCTCCATACTATTCTGTCTAGCAAACTTCAATTATGAACGAACCCACACCCGAACACTCATCACCACACAAGGCATACAACTCACCTCCCCCGCCCTCACAGCTTGGTGGTTCCTAGCCTGCTCAATAAACATAGCCCTCCCCCCAACATTCAACCTAACCGGCGAACTAACCGTTATTACATCGCTATTTAACTGACTAGACATTACAGTTATCCTAACAGGACTGAGTGCCTTCATAACGGCAGTCTACACCTTATACATATTCTCAGCCACCCAACAATGAACACTACCCACTAATGCCACCCTATTACCTCCAACCCAAACCCGAGAACACCTCCTGATACTCCTCCACCTCCTCCCCTCTGCGGGCCTTACCACCAACCCAAAACTCACCTCCGCCCAATA---AATTACCCCCACAAATACACTTTTTTTGTTCTCCTTCTCTCTTTGCATCATTGGCCTAATTACCCACCACAACCACCTCCTCTCAACACTCCTCTGCCTTGAAGGGATGGCACTCTCTATCTTCTTAGCCGTCACAATATCCTCACTCCAATCACACCCCTCATCATTCATCCTCCCCCTAACAATCCTGACCCTCTCTGCCTGCGAAGCAGGCACCGGCCTCGCCCTACTAGTCGCCTCCGCCCGAACACACAACACCGCCAACCTTAAAAACCTAAACCTCCTTCAATGCTAAAT----GTCCCACACGACACTCCTCCAAACATTCTCCGCCATCCCCTTTCTAATCCTATTACTGGCAACCCTTCCCCTAGCCCATAAAACCAACAACACACCAGCCAAATCCAAAACAATAATAGCCAAACTCGCTTTCTTCACAAGCCTCCCCCCTCTCTTTTTAGTGGTGCTAAATAACAGCCCCGCCCTGTCACACCACCTATCTTTATTCCACCTAGAATCCCTCAACCTCCCCATCACCCTAAAAATTGACACTTATTCAACCTTCTTCATCCCAACAGCATTATTCATCACATGGTCAATTATAGAATTCTCCAAATCGTACATAGACTCAGACCCCAAAATTACCAGCTTCTTCAACCACCTCCTAGTATTTATCCTAATAATAATAATCCTAGTAAGCGCTAGCAACCTATTCCACCTCTTTATCGGCTGAGAAGGGGTAGGCATTATATCGTACCTACTCATCAACTGATGATCCTTCCGAGCAAACGCCAACAAAGCAGCGCTCCAGGCTATCATCTACAACCGACTATCAGACATTGGAATCCTCACCACACTGACATGAATAACCACCCACAATCTCACCTTAGACATCACAAACCTCTCC---CCCCCAAACTACCCACTAATCCCAGCCGCAGCCATCATCCTAGCAGCAGCGGGAAAATCAGCCCAAATTGGCCTCCACCCCTGACTCCCAGCAGCAATAGAAGGGCCAACACCAGTTTCCGCCCTACTCCACTCAAGCACCATAGTAGTAGCAGGCGTCTTCTTACTAATCCGAACTTCTCCAATCATCTATAGCAGCCAAACAACAACTACAGCCTGCCTAATTCTAGGAGCAATCACCTCTCTATTCGCAGCCGCATGCGCTTTCGCCCAAAATGACATAAAAAAAATCATCGCATTCTCAACCTCAAGTCAACTTGGACTAATAGTAACCACAATTGGACTTAAACAACCCGAACTCGCATTCCTACACATCCTAATGCACGCCTTTTTCAAAGCAATACTCTTCCTGTGCGCAGGATCAATCATCCACAGCCTCAATAACGAACAAGACTTCCGAAAAATAGGAAACCTCAAAAAAGCAATACCAATTACCACCTCTTGCCTTACCATCGGAGTACTAGCCCTCACCGGCACGCCCTTCCTCTCCGGATTCTACTCCAAAGATGCCATTATTGAATCACTAAACACCTCACATATTAACGCCTGAGCCCTTGCCATCACCCTACTCGCCACCTCCTTCACTGCAATCTATAGCCTCCGCATAGTCTACTTCACCCTACTAAATACCAATCGACTAGCAACCACGAGCCCGATAAACGAAACCCCAAAAACCACCAACCCTATTCTCCGTTTAGCCATTGGAAGTGTAATCACAGGACTATTGACCTTAAATTTCATACTACCAACCCACACCCCCCAACTAACTATACCAACCACAATCAAACTTATAGCCCTAACCATCACAGCAATTGGCATACTCACTGCAATGGCCCTAACCCTCACCACCGACAAATTCCCACCATCCACAAAAGATACCCAACCACCCCCGCTAACAAAACTAATATTCTTCAACCCAATTATCCACCACCTATTCTCCTCCACCACCCTGCACATCAGCCAAAAGCTCTCCACACACCTAACAGACCAACTATGATATGAAACCCTTGGGCCAAAAACAACGGCCAGCCTGCAAACCATGATAACCAAAACACTAACCCCCTACCACAAAGCAAAAATAAAAAAATACCTCAAAACCTTCCTCTGAGCCATCACCATCACCCTCCTCCTC---------TGAA---------GG---------------------------------ATGGAATTTACATT---ATTTTTGGGTTGTCTGGTGTTGGCGTGTGCGGTGATGGTGGGGGCGAATTCTGGGATTCATTTTGCTGTGTTGAGCTTGGTATTTATGGCTGTGTTTGGTAGCTGTTTGGTGGTGATTGAGGGTGGGAGTTTTATGCCGTTGGTGGTGTTGTTGGTTTATTTGGGGGGGTTGTTGGTAGTGTTTGCTTTTTGCGTTGGGTTTACTGACGATCCGTTTG---------GGATGGTGGGGGCTTCTAAGGGGTTTGTGCTTTTTTGTTGGATTGGTCTTCTTGTTGGGGGGGTTTGTATGTGTGGACATTTGTGGGGTTGGTGGGTCGTTGGGGTGAGCGGGTTGGTGGATGTAGAGGTTGTTGGGGGTGGGGCGGCTGATGAGTTGTTGGGGGTGGGTTTAATGTATTCAAGTGGGTGGGGTTTTGTTGTGATTTGTGGTTGGGCCTTGCTGGTTACGTTGCTCGTGATTACGGGGCTTGTTCGGGGTTGTTGTTGGGGGTCACTTCGTCCCTTCAGA

*Paleosuchus palpebrosus 4*

------------ATGAACACAAACCTATTCGACCAATTCCTAATCCCCAACCTCTTGGGAACCTCCCTACTAACACCCGCCCTACTAATAACCATTGCCCTCGTGCTAAACCCCCAAAATCTATGACTCCAACACCCAACAGCAACAATTAAAACCTGACTCATCAACCAAATTACCAAACAAATCATAACCCCAATCAACAAGTCAGGACATAAGCACTCCCTAACCCTTATCTCCATCCTAGTTCTCCTCCTCCTCACAAACCTACTAGGCCTACTCCCATACACATTTACACCAACAACACAACTCTCCATAAACATAGCCCTCGCTATCCCCCTATGACTGATGACTGTACTAACAGGCATGCGAACTCAACCAACAACCTCCTTAGCCCACCTTCTTCCAGAAGGAACCCCCACCCCACTAATCCCAGTCCTCATTATAATTGAAACAATTAGCCTATTAATTCGACCAATCGCATTGGGTGTACGATTAACAGCCAACCTAACCGCAGGCCACCTACTAATTCAATTAATCTCCATTGCTACACTAAACCTCTGACCCATCATACCACCCCTCAGCCTATTAACACTAACAACCCTGATCCTGCTCCTCCTACTAGAATTCGCCGTAGCCATAATCCAAGCATACGTCTTCGTCCTCCTACTCTCCCTATACCTACAAGAAAACACATAAATGCCCCAGCTAAACCCAGAACCCTGACTAACAGTCCTTCTAACCACCTGAATCTTCTTTATCGCCATTCTCCAGCCTAAAATCGCCACACTAACCGCCACAAACAACCCCACCCCACACAAACCACAGACCACC-AAAACATGACACTGGCCATGAACACAAACCTATT--------------ATGACCCACCAACTACGAAAATCCCACCCACTCCTCAAACTCTTCAACCACTCCCTCATTGACCTCCCAACACCCTCAAACATCTCCGCCTGGTGAAACTTTGGATCACTACTGGGCCTGACCCTCCTAATCCAAATTCTAACAGGAGTCTTCCTAATAATACACTTCTCATCAAGTGACACCACGGCCTTCTCATCCGTCGCCTACACCTCCCGCGAAGTCTGATTCGGATGACTTATCCGAAACCTCCACACAAACGGAGCCTCACTCTTCTTCATGTTTATTTTCCTCCACATCGGACGGGGCCTGTACTACGCCTCCTACACCCACGAAAAGACATGAGCTGTAGGAGTTATTATACTCTTCCTACTAATAGCTACAGCATTCATGGGTTATGTCCTCCCCTGAGGACAAATATCATTCTGAGGGGCAACCGTAATTACAAACCTGCTGTCTGCCACCCCATACATCGGAGACACCATCGTGCCATGAATTTGGGGGGGACCCTCCGTAGACAACGCAACACTCACACGCTTTACCGCCCTTCACTTCCTCCTCCCGTTTACCCTCCTAGCCCTGCTCATCACCCACCTGATCTTTCTACACGAACGGGGGTCCCTCAACCCCCTCGGACTAAGCCAAAACGCCGACAAAATCCCATTCCACCCCTACTACACACTAAAAGATGCTCTAGGAGCAACACTAGCCGCCTCCCTACTACTCACCTTCGCCCTATACCTCCCAACCCTACTAGGCGACCCAGAAAACTTCACCCCAGCAAACCCCATAACCACCCCATCACACATCAAACCTGAATGATACTTCCTATTTGCCTATGCCATCCTACGATCTATCCCAAACAAGCTCGGAGGCGTACTAGCCATATTCTCCTCCATCTTTATCTTATTCCTAGTGCCTGCCCTTCACACAGCCAAACAACAGCCCATATCCGTCCGCCCCTTATCCCAACTCCTATTCTGAGCCCTCATCCTAGACTTCTTTGTACTTACATGAATTGGGGGCCAACCAGTAAACCCCCCATTCATCCTAATGGGCCAAATCGCCTCCTCACTCTTCTTCATCACCATCCTCATCCTCATACCCACACTAGGGTCCCTAGAGAACAAAATAACC------TAA-----------------------ATGTTCATCAACCGCTGATTTTTCTCCACAAACCACAAAGACATCGGCACCCTGTACTTCATCTTCGGGGCCTGATCCGGAATAGTAGGCACAGCACTCAGCCTTCTCATCCGAACAGAACTAAGCCAACCAGGACCCCTACTAGGAGACGACCAAATCTACAATGTAATCGTCACTGCCCACGCCTTTATTATAATCTTTTTTATAGTAATACCCGTCATGATCGGCGGATTCGGAAACTGACTCCTGCCCTTAATAATTGGAGCCCCAGACATAGCATTCCCGCGAATAAACAACATAAGCTTCTGACTACTCCCCCCATCCTTCACACTGCTGCTCGCCTCTTCCTGCATTGAAGCGGGGGCCGGAACAGGATGAACCGTCTATCCCCCCCTAGCTGGAAACATAGCCCACGCCGGACCATCAGTAGACCTAACCATCTTCTCCCTACACCTTGCCGGAGTATCTTCCATCCTAGGCGCAATCAACTTCATCACAACAGCCATTAACATAAAACCCCCAGCCATGTCCCAATACCAAACACCCCTATTTGTTTGATCGGTCTTAATCACAGCCGTACTACTCTTACTCTCCCTCCCAGTACTAGCTGCCGGAATTACTATACTACTCACAGACCGAAACCTAAACACAACCTTCTTTGACCCCGCAGGAGGGGGGGACCCCATCCTATACCAGCACCTTTTCTGATTCTTCGGCCACCCCGAAGTCTACATCCTCATCCTACCTGGATTTGGAATAGTCTCACACGTCGTCACCTTCTACTCAAACAAAAAAGAACCATTCGGCTACATAGGAATAGCATGAGCCATAATATCTATCGGATTCCTAGGATTCATCGTATGGGCCCACCACATATTCACAGTCGGAATAGACGTCGACACACGAGCATACTTTACTACTGCCACAATAGTTATTGCCATCCCCACCGGGGTAAAAGTGTTTAGTTGACTAGCAACCATCTACGGGGGCATCGTCAACTGACAAGCCCCAATACTATGAGCACTAGGCTTCATCTTCTTATTCACCGTAGGTGGCCTTACTGGAATCGTCCTAGCCAACTCCTCCCTAGACATCGTTCTCCACGACACCTACTACGTAGTCGCCCACTTCCACTACGTATTATCAATAGGAGCAGTCTTTGCCATCATATGCGGATTTACCCACT-GATTCCCACTA-TTCACAGGATTTACCCTCCACCCAACATGAACAAAAA-TCCAATTCACAATTATATTCTTAGGAGTAAACCTTACCTTCTTCCCACAACACTTCCTAGGGCTATCTGGAATACCTCGACGATACTCTGACTACCCAGACGCATACACCATCTGAAATCTAACATCATCAATTGGATCCCTAATCTCCCTAACCGCTGTCATCCTACTAATGTTCATTGTATGAGAAGCATTTTCTTCCAAACGAAAAACAACCACACCTGAGATAACAACAACCAACATCGAGTGACTTAACAACTCCCCACCACCCCACCACACATACGAAGAACCAGTTTTTACTGTAACACACAAACCAACCGACCTC-----------------------------CTAAACTATGGCCAACCCCACACATCTGGGCTTCCAAGACGCAATATCCCCCCTCATAGAAGAGCTATTATACTTCCATGACCACACTCTAATAATCCTCATCCTAATCAGCTCCCTTGTATTCTACACAATCTCCGCCCTGCTCCTCCAAAAACTCTATCACTCAAACACCTCCGACGTCCAAGAGGTAGAAATAGTATGAACCATCCTACCAGCCATCGTCTTAATTTCAATCGCCCTCCCCTCTCTGCGAGCCCTTTACCTTATAGACGAAACCAACAACCCATGCCTGACTATCAAAGCAACCGGACACCAGTGATACTGATCCTACGAATACACTGACTTCTCCACAGTAGAATTTGACTCCTACATGACACCCCCACAAGATCTCTCTCCAGGTCACCTCCGCCTCCTAGAAGTTGACCACCGCATAATTACCCCAATAAACTCAACAATTCGGATATTAATCACAGCAGAAGATGTCCTGCACTCATGGGCAATTCCATCCATCGGAACAAAAATAGACGCAGTCCCAGGACGCCTAAACCAAGTCATAATTACACTAACCAACCCTGGCGTATTCTACGG-CCAATGCTCCGAAATCTGTGGAGCAAACCACAGCTTCATACCCATCGTAATAGAAACCACCCCATTAAACCACTTTCAACTCTGACTAGAAGACCACACTACCTCATATGTCCCACCAAACCCACCCATTCCATATAGTACACCCAAGCCCCTGACCCCTAGCCGGCGCCATAGCCGCCATATTACTAACAACCGGCCTAACCTTTTGATTTCACTACAACTCTTGCCCCCTCCTCCTACTAGGCCTGACCTCAGCCCTACTTGTAATATTCCAATGATGACGAGACATTGTACGAGAAAGCACCTTCCTAGGACACCACACACCCCCAGTACAAAAAGGATTGCGCTACGGCATAATTCTCTTTATTACATCTGAAGTCTTCTTTTTCCTAGGCTTCTTCTGAGCATTTTACCACTCAAGCCTCTCCCCAACCCCAGAGCTAGGAGGACAATGACCCCCTACCGGAATCACCCCCCTCGACCCCTTTGAAGTCCCCCTCCTTAACACAGCCGTCCTACTCGCCTCCGGAGTAACAGTAACATGGGCCCACCACAGCCTAATAAACACCCACCGCATGCAAGCAATCCACGCTCTCGCACTCACTGTACTTCTCGGATTCTACTTCACCACCCTTCAAGCCATAGAATACTATGAGGCCCCCTTCACTATCGCAGATAGCACCTACGGATCAACATTCTTTGTTGCAACAGGCTTTCATGGCCTCCACGTCATCATCGGCTCAATCTTCCTCACAGTCTGCCTATATCGACAAACAAAATACCACTTCACATCCAACCACCACTTCGGATTCGAAGCCGCCGCCTGATACTGACACTTCGTAGACATAATCTGACTATTCCTCTACATCTCAATCTACTGATGAGGCTCATATCGACTCCCTCACCATCCTCCCCCCAATCTTATTTATTGCCTCCGTCCTAATTGCAGTCGCCTTCTTAACAGCCCTAGAACGAAAAGTTATTGGCTACATTCAATTACGAAAAGGACCAAACATTGTTGGCCCCATCGGCCTGCTCCAACCCCTCGCAGATGGATTCAAACTTATTATTAAAGAACTGACCCTCCCCCTACTTGCCACACCAACCCTATTCATCCTCGCCCCGGCAGCAGCCCTAATACTAGCCCTCACCATGTGGTCCCCCCTTCCAATACCATTCCCCCTCACAGACCTCAACCTAGGCCTGCTTTTTCTACTAGCCATATCAAGCCTCATGGTCTACTCACTTCTCTGATCCGGGTGGGCCTCAAACTCAAAATACGCCCTAATAGGGGCCCTCCGAGCAGTAGCCCAAACAATCTCCTACGAAGTCACACTAGCCATTATTGTACTCTCCATTATCTTGCTCTGCGGGGGGTTCTCACTACACGCCCTCACCACCACACAAGAGCCCCTATATCTTGCACTGCCTGCATGGCCCCTAATAATAACGTGATATATCTCCACACTAGCAGAAACAAACCGCGCCCCATTCGACTTAACAGAGGGCGAATCAGAACTCGTATCTGGGTTCAATGTTGAATACGGCGCAAGCCCCTTCGCACTGTTCTTCCTAGCCGAATACGCCAACATCATGTTAATAAACACCCTGACCGTCATCCTCTTCCTCAGCCCATCACCCCAAACCCACCCTCCAACGCTATTCACCATCACCCTAATAACCAAAACCCTCTTACTGACTACAACCTTTCTATGAGTCCGAGCATCCTACCCACGATTTCGCTATGATCAACTCATACACCTCCTATGAAAAAGCTTCCTCCCCCTAACATTAGCCCTCTGCCTATGACACACATCACTACCAGTCTCAATATTTGGACTTCCCCCAACAACCTAGATGTCCCTCTCATTACCCCTCATCCTAACAACAATGGCCCCAGCAACACTCATCTTCCTCATATCATCCCACCTAGTACTAATGTGAATCGCACTAGAACTCAACACACTGACAATCCTTTACATAATTGCCAACAAATCCCACCCCCGAGCCATTGAAGCCACCATCAAATACTTCCTCACACAAGCACTAGCATCCACATTAATTATCCTTTCCGGAACTATCAACTACGAGATAACAGGAAGCTGGGAAATCACAGAAATAACTAGCCCAACCCCCTTAACCATACTCACCCTCGCCCTATCCATCAAGCTAGGATTAGTCCCATTTCACTTCTGAGTGCCAGAAGTCCTTCAAGGAATACCCACACTCCCCGCAATCTTCCTACTTACATGACAAAAAGTAGGCCCACTAATTATGTTCTTCTTAATCAGCCCCTTTATCAACCATAATTTAGTCTCCACAATGGCCATTATGTCCTCCACTGTTGCAGGGTGGCTGGGGCTCAACCAAACCCAGGTACGAAAGCTAATAGCAATGTCCTCCATCGCCCAAATAGCATGAATAATTGTCGTTATTAAATACGCACCCTCCCTAACAGTCCTCTCTTTCTACGTGTATTCAATAACCGTCTCCGCCGCACTACTAATGCTAAACAAGCTATCTGTAACCACCATAAAATCACTTGCCACTTCCTTCTCAAAAAACCCAATTGCCACCTTACTCCTAATAGCCTCCATCCTCTCACTATCCGGCCTTCCCCCCCTGGCCGGCTTCACACCTAAATGATTAATAATCAACCAGCTTGTTGCAGAAAAGGCAGTCTGAACCGCACTTCTCATACTAGCATCCTCACTCTTGACCCTGTTCTTCTACCTTCGCATGTGATACTACACCATCTCCACCCTGCCCCCAAACACCTCAAACATATCCCGCCCTTGACGAAAACCCACCTCCAAAAGCAACCTCACCGCCAACACCCTCGCTACAGCTGCCTCCACCCTCCTACTATGCACTACACTCATAAAAGCAGCCACCAAAAG------------------AA----TAGACCTCCTCATTATATTCTCAATAGCCTCCATCACTGCAACAATCCTGATTATCTCAAACCTCACAATAGCCGAAGCATCGCCCGACCCTGAAAAACTATCCCCATACGAATGTGGATTTGACCCCCTAGGGTCTGCTCGACTTCCACTATCAATCCGGTTCTTCATGATCGCTATCTTATTCTTACTTTTCGACCTTGAAATCGCCATCTTACTCCCACTCACGTGATCCACCCACACCTTTCACCCAACAAAAACCATCACATGTATTATCGCAATCTTCCTACTACTATTCATCGGTCTGATATACGAATGACTCCATGGCGGCCTAGAATGAGCAGAATAGATGCTAAAAATCCTGCTACCCACGGCCATGGTTGGCCCAA---CAACCCTCACCCCAAACAAGACCATCTGACTATCCCCAACAACTTGCGCCACTGCTGTAATAATTATCCCCCTACTAACCCTAAACCCCTCAGATACTTTAACAAACCCCAGCAACCAAGCCCTCGGTAGCGACCAATATTCAAGCCCCCTGATAATACTATCCTGCTGACTCCTCCCCCTGATGCTCATGGCCAGCCAAAACGCAATATCCACAACCCTGGCCCCCCAAATCCAAATATTCATCTCCATTCTTGCAACACTCCAACTCACCCTGCTCATGGCATTCATGGCCCTAGACTTAATACTATTCTATATTATATTTGAAACCACCCTACTTCCAACCCTAGTAACCATCTCCCGATGAGGCGCCCGACCAGAACGTCTAAATGCCGGAGCCTATTTCCTATTCTACACTATTACCAGCTCAATCCCCCTAATAATTAGCATTCTAATAATCCACAATCTCATCGGCACCTTGTCCCTCCCAATCCTACAACTAACCCCAATAACGAACCACCCCCCCTGAACAAATACACTATTCTGACTCTCAACACTTGTAGCCTTCCTGGTAAAAATCCCAATCTACGGACTACACCTCTGACTCCCCAAAGCTCACGTCGAAGCCCCAATTGCAGGATCAATAGTACTGGCCGCAGTACTTCTAAAGCTGGGAGGATACGGGATGCTACGAGTAACAAACCTGCTCACCGCGCAGGTTAATACTACCTACCTCCCCCTACTAACCCTAGCACTCTGAGGGGCACTAACAACAAGCCTCATCTGCCTACGACAAACAGACCTAAAATCCCTAATCGCCTATTCATCGGTTAGTCACATAGCCCTGGTAACAGCCACGATCCTCGCCCGAGACCACCTTACCCCAACAGGATCCATAATCCTAATAGTAGCCCACGGACTAACCTCCTCCATACTATTCTGTCTAGCAAACTTCAATTATGAACGAACCCACACCCGAACACTCATCACCACACAAGGCATACAACTCACCTCCCCCGCCCTCACAGCTTGGTGGTTCCTAGCCTGCTCAATAAACATAGCCCTCCCCCCAACATTCAACCTAACCGGCGAACTAACCGTTATTACATCGCTATTTAACTGACTAGACATTACAGTTATCCTAACAGGACTGAGTGCCTTCATAACGGCAGTCTACACCTTATACATATTCTCAGCCACCCAACAATGAACACTACCCACTAATGCCACCCTATTACCTCCAACCCAAACCCGAGAACACCTCCTGATACTCCTCCACCTCCTCCCCTCTGCGGGCCTTACCACCAACCCAAAACTCACCTCCGCCCAATA---AATTACCCCCACAAATACACTTTTTTTGTTCTCCTTCTCTCTTTGCATCATTGGCCTAATTACCCACCACAACCACCTCCTCTCAACACTCCTCTGCCTTGAAGGGATGGCACTCTCTATCTTCTTAGCCGTCACAATATCCTCACTCCAATCACACCCCTCATCATTCATCCTCCCCCTAACAATCCTGACCCTCTCTGCCTGCGAAGCAGGCACCGGCCTCGCCCTACTAGTCGCCTCCGCCCGAACACACAACACCGCCAACCTTAAAAACCTAAACCTCCTTCAATGCTAAAT----GTCCCACACGACACTCCTCCAAACATTCTCCGCCATCCCCTTTCTAATCCTATTACTGGCAACCCTTCCCCTAGCCCATAAAACCAACAACACACCAGCCAAATCCAAAACAATAATAGCCAAACTCGCTTTCTTCACAAGCCTCCCCCCTCTCTTTTTAGTGGTGCTAAATAACAGCCCCGCCCTGTCACACCACCTATCTTTATTCCACCTAGAATCCCTCAACCTCCCCATCACCCTAAAAATTGACACTTATTCAACCTTCTTCATCCCAACAGCATTATTCATCACATGGTCAATTATAGAATTCTCCAAATCGTACATAGACTCAGACCCCAAAATTACCAGCTTCTTCAACCACCTCCTAGTATTTATCCTAATAATAATAATCCTAGTAAGCGCTAGCAACCTATTCCACCTCTTTATCGGCTGAGAAGGGGTAGGCATTATATCGTACCTACTCATCAACTGATGATCCTTCCGAGCAAACGCCAACAAAGCAGCGCTCCAGGCTATCATCTACAACCGACTATCAGACATTGGAATCCTCACCACACTGACATGAATAACCACCCACAATCTCACCTTAGACATCACAAACCTCTCC---CCCCCAAACTACCCACTAATCCCAGCCGCAGCCATCATCCTAGCAGCAGCGGGAAAATCAGCCCAAATTGGCCTCCACCCCTGACTCCCAGCAGCAATAGAAGGGCCAACACCAGTTTCCGCCCTACTCCACTCAAGCACCATAGTAGTAGCAGGCGTCTTCTTACTAATCCGAACTTCTCCAATCATCTATAGCAGCCAAACAACAACTACAGCCTGCCTAATTCTAGGAGCAATCACCTCTCTATTCGCAGCCGCATGCGCTTTCGCCCAAAATGACATAAAAAAAATCATCGCATTCTCAACCTCAAGTCAACTTGGACTAATAGTAACCACAATTGGACTTAAACAACCCGAACTCGCATTCCTACACATCCTAATGCACGCCTTTTTCAAAGCAATACTCTTCCTGTGCGCAGGATCAATCATCCACAGCCTCAATAACGAACAAGACTTCCGAAAAATAGGAAACCTCAAAAAAGCAATACCAATTACCACCTCTTGCCTTACCATCGGAGTACTAGCCCTCACCGGCACGCCCTTCCTCTCCGGATTCTACTCCAAAGATGCCATTATTGAATCACTAAACACCTCACATATTAACGCCTGAGCCCTTGCCATCACCCTACTCGCCACCTCCTTCACTGCAATCTATAGCCTCCGCATAGTCTACTTCACCCTACTAAATACCAATCGACTAGCAACCACGAGCCCGATAAACGAAACCCCAAAAACCACCAACCCTATTCTCCGTTTAGCCATTGGAAGTGTAATCACAGGACTATTGACCTTAAATTTCATACTACCAACCCACACCCCCCAACTAACTATACCAACCACAATCAAACTTATAGCCCTAACCATCACAGCAATTGGCATACTCACTGCAATGGCCCTAACCCTCACCACCGACAAATTCCCACCATCCACAAAAGATACCCAACCACCCCCGCTAACAAAACTAATATTCTTCAACCCAATTATCCACCACCTATTCTCCTCCACCACCCTGCACATCAGCCAAAAGCTCTCCACACACCTAACAGACCAACTATGATATGAAACCCTTGGGCCAAAAACAACGGCCAGCCTGCAAACCATGATAACCAAAACACTAACCCCCTACCACAAAGCAAAAATAAAAAAATACCTCAAAACCTTCCTCTGAGCCATCACCATCACCCTCCTCCTC---------TGAA---------GG---------------------------------ATGGAATTTACATT---ATTTTTGGGTTGTCTGGTGTTGGCGTGTGCGGTGATGGTGGGGGCGAATTCTGGGATTCATTTTGCTGTGTTGAGCTTGGTATTTATGGCTGTGTTTGGTAGCTGTTTGGTGGTGATTGAGGGTGGGAGTTTTATGCCGTTGGTGGTGTTGTTGGTTTATTTGGGGGGGTTGTTGGTAGTGTTTGCTTTTTGCGTTGGGTTTACTGACGATCCGTTTG---------GGATGGTGGGGGCTTCTAAGGGGTTTGTGCTTTTTTGTTGGATTGGTCTTCTTGTTGGGGGGGTTTGTATGTGTGGACATTTGTGGGGTTGGTGGGTCGTTGGGGTGAGCGGGTTGGTGGATGTAGAGGTTGTTGGGGGTGGGGCGGCTGATGAGTTGTTGGGGGTGGGTTTAATGTATTCAAGTGGGTGGGGTTTTGTTGTGATTTGTGGTTGGGCCTTGCTGGTTACGTTGCTCGTGATTACGGGGCTTGTTCGGGGTTGTTGTTGGGGGTCACTTCGTCCCTTCAGA

*Paleosuchus trigonatus*

------------GTGAACACAAACCTATTCGACCAATTCCTAATCCCTAGCCTCCTCGGGGCCTCCCTATTAGCACCCGCCCTACTAATAACCCTCATCCTCACACCAACCCCCAAAAACCTATGACTCCCCCACCCAGCAGCAACAATTAAAATATGACTCATCAGCCAAATTACCAAACAAATCATAGCCCCAATCAATAAATCAGGGCACAAACACTCACTAACCCTTATCTCCCTCCTAGTCTTCCTCCTCCTCACAAACCTACTAGGCCTACTCCCATACACATTCACCCCAACAACACAACTCTCCATAAACATAGCCCTCGCCATCCCCCTATGACTGATAACTGTATTAACAGGCATACGAACCCAACCAACAACCTCCTTGGCCCACCTCCTCCCAGAGGGAACCCCCACCCCGCTAATCCCAATCCTCATTATAATTGAAACAATCAGCCTACTAATTCGACCAATTGCACTAGGCGTACGACTAACAGCCAACCTAACCGCAGGCCACCTACTAATCCAACTAATCTCCATTGCCACACTAAACCTGTGACCCATCATACCACCCCTCAGCCTATTAACACTAACAACCCTAATCCTACTCCTCCTATTAGAATTCGCCGTAGCCATAATCCAAGCATACGTCTTCGTCCTCCTACTCTCCCTATACCTACAAGAAAACACATAAATGCCCCAACTAAGCCCAGAACCCTGACTAACAGTCCTTCTAACCGTCTGAACCTTCTTTATCATTATCCTACAGCCTAAAATCGCCACACTAACCGCCACAAACAACCCCACCCCACACAAACCCCAAACCACT-AAAACATGGCCCTGACCGTGAACACAAACCTATT--------------ATGACCCACCAGCTACGAAAATCCCACCCACTCCTCAAACTCTTCAACCACTCCCTCATTGACCTCCCAACACCCTCAAACATTTCCGCCTGATGAAACTTTGGATCACTGCTGGGCCTAGCCCTCCTAATCCAAATCCTAACAGGGGTCTTCCTAATAATACACTTCTCATCAAGCGACACTGCGGCTTTCCCGTCCGTCGCCTACACCTCCCGCGAAGTCTGATTCGGATGACTTATCCGAAGCCTCCACACAAACGGGGCCTCCCTCTTCTTCATATTTATTTTCCTCCACATCGGACGAGGCCTATACTACGCCTCTTACGCCCACGAAAACACATGAAATGTAGGAGTCATAATGCTCTTCTTACTAATAGCCACGGCATTCATAGGCTATGTCCTCCCCTGAGGACAAATATCATTCTGAGGAGCAACCGTAATCACCAACCTGCTGTCTGCTATCCCATACATCGGAGACACCATTGTACCGTGAATCTGGGGGGGACCCTCCGTAGACAATGCAACACTCACACGCTTCACCGCCCTCCACTTCCTCCTCCCATTCGCCCTCCTGGCCCTGCTCATCACCCACCTGATCTTTTTACACGAACGAGGGTCTTTCAACCCCCTCGGACTAGACCTAAACGCCGACAAAATCCCATTCCACCCCTATTACACACTAAAAGACGCCCTAGGAGCAACTCTAGCCGCCTCCCTACTACTCACCCTCGCCTTATACCTTCCGACCCTACTAAGCGACCCGGAAAACTTCACCCCAGCAAACCCCATAACCACCCCATCACACATCAAGCCCGAATGATACTTCCTATTCGCCTATGCCATCCTACGATCCATCCCAAACAAACTTGGAGGCGTACTAGCCATATTCTTCTCCATCTTTATTTTATTCCTAGTACCCGCCATTCACACAACCAAACAACAACCAATATCCGTCCGCCCCCCATCCCAACTCCTATTCTGAGCCCTCATCTTAGACTTCTTCGTACTCACATGAATCGGGGGCCAACCAGTAAACCCCCCATACACCCTAGTGGGCCAAATCGCCTCCTTACTTTTCTTCACCGTCATCCTCATCCTCATACCCACACTAGGGTCCTTAGAAAACAAAATAGCCAAACCTTAA-----------------------ATGTTCATCAACCGCTGATTTTTCTCCACAAACCACAAAGACATCGGCACCCTATACTTCATCTTCGGGGCTTGATCTGGAATAGTAGGCACAGCACTCAGCCTCCTCATCCGGACAGAACTAAGCCAACCAGGACCCCTACTAGGAGACGACCAAATCTACAATGTAATCGTCACCGCCCACGCCTTCGTCATAATCTTCTTTATAGTAATACCCGTCATGATCGGCGGATTTGGAAACTGACTCCTCCCCCTAATAATTGGAGCCCCAGACATAGCATTCCCACGAATAAACAACATAAGCTTCTGACTACTCCCCCCATCCTTCACACTACTGCTCGCCTCTTCCTGTATTGAGGCGGGGGCCGGAACAGGGTGAACCGTCTATCCCCCCCTAGCTGGAAACCTAGCCCACGCCGGACCATCCGTAGACCTTACCATTTTCTCCCTACACCTTGCCGGAGTATCCTCCATCCTAGGCGCAATCAACTTCATCACAACAGCCATTAACATAAAACCCCCAGCCATATCCCAATACCAAACCCCCCTATTTGTCTGATCAGTCTTAATCACGGCCGTACTCCTCCTACTCTCCCTCCCAGTACTAGCTGCTGGAATCACTATACTACTCACAGACCGAAACCTGAACACAACCTTCTTTGACCCAGCAGGGGGAGGAGACCCCATCCTATACCAGCACCTTTTCTGATTCTTCGGCCACCCCGAAGTCTACATCCTTATCCTACCCGGATTCGGAATAGTCTCACACGTTGTCACCTTCTACTCAAACAAAAAAGAACCCTTCGGCTACATGGGAATAGCATGGGCTATAATATCCATCGGATTCCTGGGGTTCATCGTCTGAGCCCACCACATATTCACAGTCGGAATAGACGTCGACACACGAGCATACTTTACCACCGCTACAATAATCATTGCCATCCCTACCGGAGTAAAAGTATTTAGTTGACTAGCGACCATCTACGGCGGCATTATTAACTGACAAGCCCCAATACTGTGGGCACTAGGTTTCATCTTCTTATTCACCGTAGGGGGCCTCACTGGAATTGTCCTAGCCAACTCCTCACTAGATATCGTCCTCCACGACACCTACTACGTAGTCGCCCACTTCCACTATGTATTATCAATAGGAGCAGTTTTCGCTATTATAAGCGGATTCACCCATT-GATTCCCCCTA-TTCACAGGGTTTACCCTCCACCCAACATGAACAAAAA-TTCAATTCACAATCATATTCTTAGGAGTAAACCTCACCTTCTTCCCACAACACTTCCTAGGACTATCTGGAATACCTCGACGATACTCCGACTACCCAGACGCATACACCATCTGAAATCTAATATCATCAATTGGATCTTTAATTTCCCTAGCCGCTGTTATCCTACTAATATTCATTGTATGAGAAGCATTTTCTTCCAAGCGAAAAACAACCACACCTGAAATAACAACAACCAACATCGAGTGACTTAACAACTCCCCACCACCCCACCACACATACGAAGAACCAGTCTTCGTTACAACACACAAACCAGCCGAG--------------------------------------TATGGCCAACCCCACACATCTGGGCTTCCAAGACGCAATATCCCCCCTCATGGAAGAACTATTATATTTCCACGACCACACCCTGATAATCCTCATCATAATCAGCTCCCTTGTATTTTACACAATCTCTGCCCTGCTCCTCCAAAAACTCTATCACTCAAACACCTCTGACGTCCAAGAAATAGAAATGGTATGAACTATCCTACCAGCCATCGTCTTAATTTCAATCGCCCTCCCCTCTCTACGAACCCTCTACCTCATAGACGAAACCAACAACCCATGCCTAACTATCAAAGCAACCGGACACCAATGATACTGGTCCTACGAATACACTGACTTCTCCACAGTGGAGTTTGACTCCTACATAACACCCCCACAAGACCTCCCACCAGGCCACTTCCGCCTTCTAGAAGTTGACCACCGCATAATTACCCCAACAAACTCAACCATCCGGATACTGATCACAGCAGAAGATGTCCTACACTCATGAGCAATCCCATCCATCGGAACAAAAATAGACGCAGTCCCCGGACGCCTAAACCAAGTCATAGTTACACTAACCAACCCTGGCGTATTCTACGG-CCAATGCTCTGAAATCTGCGGGGCAAATCATAGCTTTATACCCATCGTAATAGAAACTACCCCATTAAACCACTTCCAACTCTGATTAGAAAACCACACTACCTCATATGTCCCACCAAACCCACCCCTTCCATATAGTTCACCCAAGCCCATGACCCCTGGCCGGCGCCATAGCCGCCATATTATTAACAGCCGGCCTAACCTTTTGGTTTCACTACAGTTCTTACCCCCTTCTCCTACTAGGCCTAGCCTCAACCCTACTTGTAATATTCCAATGATGGCGGGACGTTGTACGAGAAAGTACCTTCCTGGGACACCATACACCCCCGGTACAAAAAGGATTACGCTACGGCATAATCCTCTTCATTACATCCGAAATCTTCTTTTTCCTGGGCTTCTTCTGAGCATTTTACCACTCAAGCCTCTCTCCAACCCCAGAGCTAGGGGGACAATGACCCCCTACCGGAATCACCCCCCTCGACCCTTTTGAAGTCCCCCTCCTCAACACAACCGTCCTACTCGCCTCCGGAGTAACAGTGACATGAGCCCACCACAGCCTAATAAACACCCACCGCATACAAGCAATCCACGCTCTCACACTCACCGTACTTCTCGGATTCTACTTCACCGCCCTTCAAGCCATAGAGTACTACGAGGCCCCCTTCACTATCGCAGATAGCACCTACGGATCAACATTCTTTGTTGCAACGGGCTTCCATGGCCTCCACGTCATCATCGGCTCAATCTTCCTCATAATCTGCCTATATCGACAAACAAAATACCACTTCACATCCAATCACCACTTCGGATTCGAAGCCGCCGCCTGATACTGACACTTCGTAGACATAATTTGACTATTCCTCTACATCTCAATCTACTGATGAGGCTCATATTGACTCCATCACCATCCTCCCCCCAATCTTATTTATAACCTCCGTCCTGATTGCAGTGGCCTTTTTAACAGTCCTAGAACGAAAAATTATTGGCTACATTCAACTACGAAAAGGACCAAACACTGTCGGCCCCATCGGCCTGCTTCAACCCCTCGCAGACGGATTTAAACTCATCATCAAAGAACTAACCCTCCCCCTACTTGCCACACCAACCCTGTTCATTCTCGCCCCAGCAGCAGCTCTAATGCTAGCCCTCACCATATGATCCCCCCTCCCAATACCATTCCCCCTCACAGACCTCAACCTAGGCCTGCTTTTTCTACTGGCCATATCAAGCCTTATGGTCTACTCACTCCTCTGATCCGGGTGGGCCTCAAACTCAAAATACGCCCTAATGGGGGCCCTACGAGCAGTAGCCCAAACAATCTCCTACGAAGTCACACTAGCCATTATTGTACTGTCCATTGTCTTACTTTGCGGAGGATTCTCACTACAGGCCCTCACCACCACACAAGAACCCCTATACCTCGCACTACCCGCATGGCCATTAATAATAACATGATATATCTCCACACTAGCAGAAACAAACCGCGCCCCATTCGACTTAACAGAGGGTGAATCAGAGCTCGTATCCGGATTTAATGTTGAATACGGCGCAAGCCCTTTCGCACTATTCTTCCTAGCCGAATACGCCAACATCATGCTAATAAACACCCTAACTGTCATCCTCTTCCTCAGCCCCTCACCCCCAACTCACCTTCCAACACTATTCGCCCTCACCTTAATAACCAAAACCCTTTTACTAACTATAACCTTTTTATGAGTCCGAGCATCCTACCCGCGATTTCGCTACGACCAGCTTATACACCTCCTATGAAAAAGCTTCCTTCCCTTAACACTAGCCCTCTGCCTATGACACACATCACTACCAGTCTCAATATTTGGCCTTCCCCCAACAACCTAGATGTCCCTCTCATTACCCCTGATCCTAACAACAATAACCCCAGCAACACTCATCTTCCTCCTATCATCCCACCTGGCACTAATATGAATCGCGCTAGAACTCAACACACTGACAATCCTTTACATAATTGCCAACAAATCCCACCCCCGAGCCATTGAAGCTACCATCAAATACTTCCTCACACAAGCACTAGCATCCACATTAATTATCCTTTCCGGAGCTATTAACTACGAAACAACAGGAAGCTGGGAGATCACAGAAATAACCAACCCAACCCCCCTAACCATGCTCACCCTCGCCCTATCAATCAAATTAGGATTAGTCCCCTTTCACTTCTGAGTGCCAGAAGTCCTTCAAGGAATACCCACACTCCCAGCAATCTTCCTACTCACATGACAAAAAGTAGGCCCACTAATCATGTTCTTCCTAATTAGCCCCCTTGTCAACCACAACCTAATCTCCACAATAGCCATCCTATCCTCCACTATTGCAGGGTGACTAGCACTCAACCAAACCCAAGTGCGTAAATTAATAGCAATATCCTCCATCGCCCAAATAGCATGAATAATTGTTATTATCAAGTACGCACCCTCCTTAACAGTCCTCTCTTTCTACGTATACTCCATAACCGTCTCCGCCGCATTACTAGCACTAAACAAGCTATCTGTAACCACCATAAAATCACTTACCACCTCCTTCTCAAAAAACCCAATTGCCACTTTACTCCTAATAGCCTCTATCCTCTCACTATCAGGCCTTCCCCCCCTAGCCGGCTTCACACCTAAATGATTAATAATCAACCAACTCATTGCAGAAAAAGCAACCTGAACCGCACTTCTTATACTAGCGTCCTCACTCTTGACCCTGTTCTTCTACCTCCGCATATGATACTACACCATCTCCACCATGCCCCCAAACACCTCCAACATATCCCGCCCTTGACGAAAATCCACCCCCAAAAGCAACCTCACTGCCAACACCCTCGCCACAGCTGCCTCCACCCTTCTACTCTGTACCACACTCATAAAAGCAGCCACCAAAAG------------------AA----TAGACCTTCTCATTATATTCTCAATAGCCTCCATCACCGCAACAATCCTAATTATCTCAAACCTCATAATGGCCGAAACATCACCCGACCCTGAAAAACTATCCCCCTACGAGTGTGGATTTGACCCCCTAGGATCCGCTCGACTCCCCCTATCAATCCGATTCTTCATAATTGCCATCTTATTCCTACTTTTCGACCTTGAAATCGCCATCTTACTCCCCCTCGCATGATCCATCCACACCCTTCACCCAACAAAAACCATCATATGCACTATCGCAATCTTCCTACTACTATTCATTGGTCTGATATACGAATGACTCCAGGGCGGCCTAGAATGAGCAGAATAGATGTTAAAAATTCTCCTACCCACAGCTATAATCACCCCAATAACAACCTTTACCTCGGACAAAACCATCTGACTATCCCCAACAACCTATACCACCGCTATAATAATCATTCCCCTACTAACCCTAAACCCCACAGATTCCCTAACAAACCCAAACAGTCAAGCCCTGGGCAGCGACCGATATTCAAGCCCCCTAATAATACTATCCTGCTGACTCTTCCCCCTAATACTCATAGCCAGCCAAAACTCAATATCCACAACCCCCACCCCCCAAACCCAAACACTCATCTCCATTCTCGCAATACTCCAACTCGCCCTACTCATGGCATTCATAGCCCTAGACCTGATACTATTCTATATTATATTTGAAGCCACCTTAATTCCAACCCTGGTAACCATCTCTCGATGGGGCGCCCAACCAAAGCGTCTAAACGCTGGGACTTACTTTCTATTCTACACTATTACTAGCTCAATCCCCCTCATAATTGGCATTCTAACAATCCACAACCTCAGCGGTACCACATCCCTTCCAATCCTACAACTAACCCCAATAACGAACCACCCATCCTGAACAAATACACTATTCTGATTCTCAACACTCCTGGCCTTCCTAGTAAAAATCCCAATCTACGGACTACACCTCTGACTCCCAAAAGCCCACGTCGAGGCTCCAATTGCAGGATCAATAGTGCTAGCTGCAGTACTCCTAAAACTAGGAGGATATGGAATGCTACGAGTAACAAACCTACTCACCGCACAAATTAACACCACCTACCTCCCCCTACTAACCCTGGCGCTTTGAGGGGCACTAACAACAGCCCTCATCTGTCTTCGACAAACAGACCTAAAATCCATAATCGCCTATTCATCAATCAGCCACATAGCTCTGGTAACAGCCACAGTCCTTATCCGAGACCCAATAACCCCAACAGGATCTATAATCCTAATAGTAGCCCATGGCCTAACCTCCTCTATGCTATTTTGCCTGGCAAACTTCAGCTACGAACGAACCCACACCCGAACACTCATCGCCATACAAGGCACACAACTCACCTCCCCCACCCTCACAGCTTGATGGTTCCTAGCCTGCTCAATAAATATAGCCCTCCCCCCAACAGTCAACCTAACAGGCGAACTAACCCTTATCACATCACTATTTAGTTGATCAGACATTACAGTCCTCCTAACAGGGCTAAGTGCTTTCATAACGGCAGTCTACACCTTACACATATTCTCAGCCACCCAACAAGGAACACTACCCCACAATGCTATCCTACTATACCCCACCAAAACCCGAGAACACCTCCTGATACTCCTCCACCTCCTCCCCTCAGCGGGCCTTTCCACCAACCCAAAACTCACCTCCACCCAATA---AATCACACCTACAAGCACGTTTTTCTTATTCTCCTTCTCTCTTTGCATCATTGGCCTAATTACCCACCACAGCCACCTCCTTTCAACTCTCCTCTGCCTTGAGGGCATAGCCCTCTCCATCTTCTTGGCCCTCACAATATCCTCACTCCAATCACACCCCTCATCATTCATCCTCCCCCTAACAATCCTAACCCTCTCTGCCTGCGAAGCGGGCACCGGCCTCGCCCTACTAGTCGCCTCTGCCCGAACACACAACACTGCTAACCTTAAAAGCCTAAACCTCCTTCAATGTTAAAT----GCCCTACACGACACCCCTCCAAGCACTCTTCACCATCCCCTTCCTAGTCCTACTATTGGCCATCCTCCCCCTAGCCCATAAAACCAACAACATATTAATTAAATCTAAGACGGTAATAGTCAAACTCGCTTTCCTCACAAGCCTCCCCCCTCTCTTCCTAGTTGTACTAAACAACAATGCCACCCTATCACACCACTTATGTCTATTCCACCTAGAACCCCTCAACCTCCCAATTGCCCTAAAAATTGACACCTACTCAGCCTTCTTCATCCCAACAGCACTATTCATCACATGATCGATTATAGAATTCTCTAAATCGTACATAGACTCAGACCCAAAAACCACCAGCTTCTTCAACCACCTCCTAGTATTTATCCTAATAATGATAATCCTAGTAAGCGCTAGCAACCTATTTCACCTCTTTATTGGGTGAGAAGGAGTAGGCATTATATCATACCTCCTCATCAATTGATGATCCTTCCGAGCAGGCGCCAACAAAGCAGCACTCCAAGCCATCATCTACAACCGACTATCAGACATCGGAATCCTCGCCGCCCTAGCATGAATAACCACCCACAGCCTCAGCCTGGATATCACAAACCTCTCCGCCCTCCCAAACTACCCACTAATCCTAACCACAGCCATCATCCTCGCAGCAGCAGGAAAATCGGCCCAAATCGGCCTTCACCCCTGACTCCCAGCAGCAATAGAAGGACCAACACCGGTTTCAGCCCTACTCCACTCAAGCACCATGGTAGTAGCAGGCATCTTCCTACTAATCCGAACTTCTCCCATCATCTACAACAGCCAAACAGCAACCACAGCCTGCCTAATCCTAGGAGCAATCACCTCCCTATTCGCAGCCGCATGTGCCCTCACCCAAAACGACATAAAAAAAATCATCGCATTCTCAACCTCCAGTCAACTTGGACTAATAATAACCACAGTCGGGCTTAAACAACCAGAACTCGCATTCCTACACATCCTAATACACGCTTTTTTCAAAGCAATACTCTTCCTGTGCGCAGGGTCAATCATCCACAACCTCAACAACGAACAAGACATCCAAAAAATAGGAAACCTCAAAAAAACAATACCAATTACCACCTCCTGCCTTATCATCGGAGTACTAGCCCTCACCGGCACACCCTTCCTCTCCGGATTCTATTCCAAAGACGCCATCATTGAATCGCTAAACACCTCACATGCTAATGCCTGAGCCCTCGCCATCACCCTACTTGCCACCTCCTTCACTGCAGTCTACAGCCTCCGCATGGCCTATTTCACCCTACTAAACACCAACCGACTAACAACCACGAACCCAGTAAACGAAACCCCAAAAACCACCAACCCCATCCTCCGTCTAGCCATCGGAAGCGTGATCACCGGACTACTAGCCTCAAACTTCATACTACCAGCCCACACCCCCCAATTAACCATACCAACCACAATCAAACTCACAGCCCTAGCCATCACAATGATCGGCATACTCGCCGCAATAGCCCTAATCCTCGCCACCAACAAATTTCCGCCATCCACAAAAGACACCCAACCACCCCCACTAATAAAACTAATATTCTTCAACCCAATCATCCACCACTTATTCTCCTCCACCGTCCTGCACATTAGCCAAAAACTCTCCACACACCTAACCGACCAACTGTGATATGAAGCCCTTGGACCAAAAACAACAGCTAACCTACAAACCACAATAACCAAAACACTAACCCCCTACCACAAAGCAAAAATAAAAAAATACCTTAAAACTTTCCTCTGAGCCATCACCATCATCCTCCTTCTC---------TGAA---------GG---------------------------------ATGGAGTTTACACT---GTTTTTGGGTTGTTTGGTGCTGGCGTGTGCGGTAATGGTGGGGGCGAATTCTGGGATTCATTTTGCTGTGTTGAGTTTGGTATTTATGGCCGTGTTTGGCAGCTGCTTGGTGGTGGTTGAGGGTGGGAGTTTTATGCCGTTGGTGGTGTTGCTGGTTTACTTGGGGGGGTTGTTGGTGGTGTTTGCTTTTTGTGTGGGGTTTACTGATGACCCGTTTG---------GGGCAGTGGGGGTTTCTAAGGGGTTTGTGCTTTTTTGTTGAGTGGGGCTTCTTGTTGGAGGGGTTTGGGTGTGTGGGCATTTGTGAGGTTGGTGGGTCGTTGGGGTGAGTGGGTTAGTGGATGTGGAGGTTGTTGGGGGTGGGGTGGCTGATGAGTTGTTGGGGGTTGGTTTAATGTATTCAAGTGGGTGGGGTTTTGTTGTGATTTGTGGTTGGGCTTTGTTGGTTACGTTGCTTGTAATTACAGGGTTAGTTCGAGGTTGTTGTTGAGGATCGCTTCGTCCCTTCAGA

*Caiman crocodilus*

------------ATGAACACTAACCTATTTGACCAATTCATAATCCCCAACCTCATAGGCACCCCCCTACTCATACCTGCCCTATTAATCATTCCCCTCCTCCTCCTAAACCCAAAAAACCAATGACTATCAAACCCAGCAACAACAATAAAATCTTGATACATTACCCAAATCACCAAACAAATCATAACCCCAATCAATAAACCCGGACACATACACTCCGTTACCCTCATCTCCTTACTAATCCTCCTCTCTTTCACAAACCTATTAGGACTACTCCCATATACATTTACCCCAACAACACAACTATCTATAAACATAGCCCTTGCCCTCCCCCTATGGGGAATAACCGTACTAATTGGACTACGAACCCAACCAACAACCTCCCTTGCTCACCTCCTCCCAGAAGGAACCCCAACCCCATTAATCCCCATCCTCATCCTAATTGAAACAATCAGCCTACTAATCCGACCTGTTGCCCTAGCCGTACGACTAACAGCAAATCTAACCGCAGGCCACTTACTTATTCAATTACTCTCTATAGCCACAATAAACCTATGGTCTATTATACCCCCTCTCAGCCTACTAACACTAACAACCCTAACCCTCCTCCTTCTCCTAGAGTTCGCCGTAGCCATAATCCAAGCATACGTTTTTGTCCTATTACTCTCCCTATACCTACAAGAAAACACATAAATGCCCCAACTAAATCCAGAACCCTGACTAGTTATCCTTCTAATCACTTGAACTTTTTTTACTACGATCCTCCAACCCCAAACAACCTCATTAATCCCAACAAATGACCCT-CTACACATAACCTACAAACCACCCAAAACATGACCCTGACCATGAACACTAACCTATT--------------ATGACCCACCAACTACGAAAATCACACCCACTCCTCAAACTTGTCAACCACTCACTAATTGACCTACCAACCCCATCAAACATCTCTACCTGATGAAACTTCGGATCATTACTAGGCCTGACCCTTATAATCCAAATCCTCACAGGAGTATTCCTAATAATACACTTCTCACCCAGCGATACCACAGCTTTCTCATCCGTCGCCTACACCTCCCGCGAGGTCTGATTTGGATGACTTATCCGAAGCTTCCACACAAACGGAGCCTCCATCTTCTTCATATTTATTTTCCTACATATCGGACGAGGCCTATACTACGCCTCCTACTTACACGAAAACACATGAAACGTTGGAGTAATCATACTATTCCTACTAATAGCCACAGCATTCATAGGCTATGTCCTCCCATGAGGACAAATATCATTCTGAGGAGCAGCCGTAATCACCAACCTACTATCCGCCATCCCATACATTGGAGACACCATCGTACCATGAATCTGAGGTGGACCCTCAGTAAACAACGCAACACTCACACGCTTTACTGCCCTTCACTTCCTCCTCCCATTCATCATCCTAGCCCTACTCATCACCCACCTAATCTTCCTACACGAACGAGGATCCTTCAATCCCGCTGGATTATCCAAAAACACCGACAAAATCCCATTTCACCCCTACTACACCATAAAAGATGTCCTAGGAGCAGTACTAGCCGCATCCATATTACTCACCCTTGCCCTCTACCTCCCAGCTCTACTAGGAGACCCTGAAAACTTTACCCCAGCAAATCCAATAGCTACCCCATCACATATTAAGCCTGAATGATACTTCCTATTTGCCTATGCTATCTTACGATCCATCCCTAACAAACTAGGCGGCGTACTAGCCATATTTTCCTCCATCTTTATCCTACTCCTAATCCCCTTCCTTCACACAACCACCCAACAACCCATATCTCTACGCCCCCTATCACAACTACTATTCTGAACCCTTATCTTAAATTTCCTCGCTCTCACATGAATCGGAGGAAAACCAGTAAACTCCCCATACATTCTACTAGGACAAATCACCTCACTACTCTATTTCATCACCATCCTCATCCTCATACCCCTGCTAGGAGCCCTAGAAAATAAAACAACCAAACCACCAT---------TTATT-------TATGTTTATCCACCGCTGATTCTTCTCCACAAACCACAAAGACATTGGCACCCTCTACTTTATCTTCGGAACCTGGGCCGGAATAGTAGGAACAGCACTTAGCCTCCTCATCCGAACAGAACTAAGCCAGCCCGGACCCCTCCTGGGAGACGACCAAATCTACAACGTAATTGTTACCGCCCACGCTTTCATTATAATCTTTTTTATAGTAATACCCGTCATGATCGGCGGATTTGGAAACTGACTCCTCCCCTTAATAATTGGAGCCCCAGACATGGCATTCCCACGAATAAATAACATAAGCTTCTGACTTCTACCCCCATCTTTCACACTACTGCTTGCCTCCTCTTGCATTGAAGCAGGAGCTGGAACAGGATGAACCGTCTACCCCCCTCTAGCCGGAAACCTAGCTCACGCCGGACCATCCGTAGACTTAACAATCTTCTCACTACATCTTGCCGGAGTATCCTCTATCCTAGGAGCAATCAACTTCATTACAACAGCCATCAACATAAAACCCCCAGCCATATCCCAATACCAAACACCACTATTTGTTTGATCTGTCTTAATCACAGCCGTACTCCTCTTGCTAGCCCTACCGGTACTAGCCGCTGGAATTACTATACTACTCACAGACCGAAACTTAAACACAACTTTCTTTGACCCTGCGGGAGGAGGAGACCCTATCCTATACCAACACCTTTTCTGATTCTTTGGCCACCCCGAAGTTTATATTCTCATCCTACCAGGATTCGGAATCATCTCACACGTGGTCACTTACTACTCAGGTAAAAAAGAACCATTCGGCTATATAGGAATAGCTTGAGCTATAATATCTATTGGATTCCTAGGATTCATCGTCTGAGCTCACCACATGTTTACCGTTGGAATAGACGTTGACACTCGAGCATACTTCACCACAGCCACAATAATCATCGCCATCCCAACCGGTGTAAAAGTATTTAGCTGACTAGCAACCATTTATGGGGGCATCATTAACTGACAAGCTCCAATATTATGAGCACTAGGCTTCATTTTCCTATTCACCGTAGGGGGCCTCACCGGAATTGTCCTAGCCAACTCCTCACTAGACGTTGTTCTTCACGACACATACTACGTAGTCGCCCACTTCCACTACGTATTATCAATAGGAGCAGTTTTCGCCATTATAAGCGGGTTTACTCATT-GATTTCCCCTA-TTCACAGGATTTACCCTCCACCCAACATGAACAAAAA-TCCAATTCATAATCATATTTATCGGAGTAAACTTCACCTTCTTCCCACAACACTTCCTCGGACTATCCGGAATGCCACGACGATACTCTGACTACCCAGACGCGTACACCATCTGAAACCTAACATCCTCAATCGGATCCTTAATCTCCTTAACCGCTGTTATCCTCATAATATTCATTGTATGAGAAGCATTCTCATCTAAACGAAAAGTAACCATACCTGAAATAACAATCACCAACATTGAATGACTTAACAATTGCCCACCACCCTATCACACCTACGAAGAACCAGTATTTACTATAACACAAAAACGCCCTGACAT-----------------------------------ATATGGCTAACCCAATACATCTAGGCTTCCAAGATGCAATATCCCCACTCATAGAAGAACTACTATATTTCCACGACCATACCATAATAATCCTCATCCTAATCAGCTCCCTTGTATTCTACACAATATCTGCCCTACTCCTTACTAAACTCTACTACTCAAACACCTCAGACGTCCAAGAAATCGAAGTAATCTGAACCATTCTACCAGCAATTATTCTAATTTCAATTGCCCTCCCATCTCTACGCACCCTCTATCTTATAGATGAAACCAATAACCCCTGCCTAACCATCAAAACAACCGGACACCAATGATATTGATCTTACGAGTACACTGACTTCTCCACATTAGAATTTGACTCTTACATAACACCCCCACAAGACCTCCTACCCGGTCATCTTCGCCTCCTAGAAGTAGACCACCGCATAGTCACCCCAATAAACTCAACTATTCGAGTGTTAGTCACAGCAGAAGATGTTCTCCACTCATGAGCTATCCCTTCTATCGGAACAAAAATAGACGCAGTCCCTGGACGCCTAAACCAAACCATAATTACACTAACCAACCCTGGCGTATTTTACGG-CCAATGTTCTGAGATTTGCGGAGCAAATCACAGCTTCATACCAATTGTTATAGAAACAACCCCACTAAACCACTTCCAACTCTGATTAGAAAACCGCTCCACCTCATATGTCCCACCAAACCCACCCATTCCATATAGTCCACCCAAGCCCATGACCCCTAACCGGAGCCATAGCCGCCATAATCCTAACAACGGGCCTAACCCTATGATTTCACTACAACTCTTATCCAGCCCTACTACTCGGCCTAATCTGCACATTACTAGTAATATTCCAATGATGACGAGATATTGTACGAGAAAGCACCTTCCTAGGACACCACACACCCACAGTACAAAAAGGATTACGCCTTGGAATAATCCTTTTTATTACATCTGAAATCTTCTTCTTCCTAGGCTTCTTCTGAGCATTCTACCACTCAAGCCTATCCCCAACCCCCGAACTAGGAGGACAATGACCCCCCACCGGAATTACCCCTATTGACCCCTTCGAGGTTCCCCTCCTAAATACTGCCGTCCTATTAGCATCCGGGGTAACAGTAACATGGGCCCACCATAGCCTAATGAACGCCAACCGCGTACAAACAATCCACGCCCTCACACTCACCGTACTCCTAGGAGTTTACTTCACCGCCCTTCAAACCATAGAATATTACGAAGCCCCATTCACTATTGCAGATAGCACCTACGGATCAACATTCTTCGTTGCTACTGGCTTCCACGGCCTACACGTCATCATCGGCTCAATCTTCCTCATAACTTGCCTATACCGACAAGTAAAATACCACTTCACTTCTAACCACCACTTCGGATTTGAAGCTGCCGCTTGATACTGACACTTCGTAGACATAATCTGGCTCTTCCTTTATATTTCAATCTACTGATGAGGCTCATATTAACCTTATAGCCATCACACCTCAAGCACTATTCATTATCTCCATTCTAATTGCAGTAGCGTTTTTAACAGCCCTAGAACGAAAAATTATTGGCCACATTCAACTACGAAAAGGGCCAAACACCGTTGGCCCTATTGGCTTATTCCAATCCTTTGCAGACGGATTTAAACTCATTATTAAAGAACTAACCCTCCCTATAATCGCCACCCCAACCCTATTCATATTAGCCCCAGCTACAGCATTAACCCTATCTCTCACCATATGGGCCCCAATCCCAATACCATTTCCCCTCGTAAACCTAAACCTAGGCCTACTTTTCCTACTAGCTATGTCAAGCCTCATAGTCTATTCACTACTATGATCTGGGTGATCCTCAAACTCAAAATACGCCCTGATAGGCGCCATACGAGCAGTTGCCCAAACAATCTCCTATGAGGTCACACTAGCCATTATTGTACTATCCATTGTACTGCTAAGCGGGGGATTCTCATTACAAACCCTCATCACCACGCAAGAACCCCTACCCCTCGCACCAACCACATGACCACTAATAATGATATGATACACATCCACACTAGCAGAAACAAACCGTGCCCCATTCGACCTAACAGAGGGCGAATCAGAACTCGTATCTGGATTCAATGTTGAATACGGCGCAAGCCCATTCACACTATTTTTCCTAGCCGAATATGCTAACATTATATTAATAAATACCCTAACTATCACCCTATTCCTCAACCCATCACCTCTAACCTTTATCCCAACACTATTTACCAGCATCATAATAACCGAAGCTCTTATATTTACCATAAGCTTCCTATGAATCCGAGCATCTTACCCACGATTCCGCTACGACCAACTCATATACCTACTATGAAAAAGCTTCCTGCCCCTAACACTAGCTTTCTGCCTATGGCACATGTCACTACCAATATCAATATTTGGCCTCCCTCCTGGGGCCTAGATGTCCCTCTCTCTCCCCCTCATCCTAATAACAATAACCCCAGCAACACTTATTTTTCTCCTATCAACCCACCTGACCCTAATATGAGCCGCACTAGAACTTAACACACTAACAGTCCTCTACCTAATCGCCAACAAATCCCACCCCCGAGCCATCGAGGCCACCATCAAATATTTCATCACACAAGCACTAGCGTCCGCACTCATAATCTTTTCCGGGGCCCTTAATTATGAAATAACAGGATCCTGAAAAATTACAGAAATAACAAATCCAATTGCCTTAACCACACTCACCCTCGCCTTATCTATCAAATTAGGGTTAGTGCCATTCCACTTCTGAGTGCCAGAAGTCCTACAAGGCATACCCACACTCCCATCAATTTTCCTACTAACATGACAAAAACTAGGCCCACTAGTTATATTCTTCCAAACTGCCCCCCTCATTAACCACAACCTAATCTCCTCAATAGCCATCTTATCATCTATTGTAGCAGGGTGACTAGGAATCAACCAAGCCCAAGTACGAAAACTAGTAGCCCTGTCCTCCATTGCCCAAATAGCATGAATAGTAATTATCATCAAATATGCACCATCTCTAGCAGTTCTAACCTTCTACATCTACTCTGTAGCCGTTTCTGCCGCACTAATCACGCTAGATAAACTCTCCGTAACAACTATAAAATCACTTACCATCTCCTTCTCAAAAAACCCGACCTCAACCCTCCTCTTAATGGCCTCCGTCCTATCATTATCTGGCCTCCCGCCCCTGGCCGGTTTTACACCTAAATGACTAACAATTAGCCAACTCATCGCAGAAAAAGCAATTTGAACTTCCTTTATTATGTTAACATCCACACTCTTAACCTTATTCTTCTACGTCCGACTATGATACTCCTCTATTTCCACCATATCCCCAAACACTGCTAACATAACCCGCCTTTGACGAAAAACTCCAAATCAAGGAAGCTTCCCCGCCAGCACTCTCGCCACAGCTGCCTCCATCCTCTTACTATGTACCGTATTTATAAAAGCAATTTCCAAAGATTAT---TTTATCTTTTAAA----TTAACTTACTTATTATACTCCTAATAACACTCATCACCACCGCAGCTCTAATTACCCTAAATCTTATAACAACCAAAACATCCCCGGACCAAGAAAAACTCTCCCCCTATGAATGCGGATTTGACCCACTCGGATCTGCCCGCCTTCCCCTATCAATTCGATTCTTCATAGTCGCCATCCTATTTCTACTCTTCGACCTTGAAGTCGCCATCCTACTCCCACTCACATGATCTATCCACACCATTAACCCCATAAAAACTATTACATGCACCATCACCGTATTTCTACTCCTACTTATCGGTCTAAGTTATGAATGACTCCAAGGCGGCCTAGAGTGGGCAGAATAAATGTTAAAAATCCTTACCCCCACGGCCACGTCCACCCTCATCACCTCCTTCATCCCGAACAAGGCCACCTGACTCTCATCAACATCATGTACAACCATCACAACAACTATCTCCGTCCTAATCCTTAATCCATCCGACCCCGCAATAGACCCAAGCAACTCATCCCTAGGCAGCGACGGCTATTCAACCCCCCTCATACTACTATCTTGTTGACTCCTCCCCATAATATTCATAGCCAGCCAAAACTTAATATCCAACCTCACTCCTCGACAAATCAAAACATTTATCGCCATCACCCTCGCACTCCAACTCGCCCTACTCATAGCATTCATAGCCATAGACCTAATACTATTCTACATCACATTCGAAGCTACACTAATCCCAACTCTTGTAATCATCTCCCGATGGGGCAGCCAACTAGCACGGCTAAACGCTGGAACCTACTTCCTATTCTACACTATTACCACCTCCATCCCCCTAATACTCAGCATCTTAGCCGTCCAAAACGTAAAAGGGACTACCTCCATGCCCGTCTTACAATTAATCCCAACAACAAGCGAAAGCCCATGAACTAATACACTACTCTGACTCTCAACACTTCTAGCCTTCCTAGCAAAAACCCCAATCTATGGCCTACACCTTTGACTACCAAAAGCCCATGTAGAAGCCCCAATTGCAGGATCAATAGTACTTGCCGCAGTGCTCCTAAAACTAGGCGGCTATGGTATACTACGAGTAACAAACCTATTAACCACACAAGACAACACCACTTATATACCACTACTATGCCTATCCCTTTGGGGAACACTAGCAGCTGGCCTCATCTGCCTACGACAAGGGGACTTAAAATCACTTATTGCCTACTCCTCAATCGGCCACATAGCACTAGTAACAGCCGCCATCCTCACCCGAGACCAACTAGCCCCAACAGGGGCTATAATCCTAATAGTAGCCCACGGTCTAACCTCATCCATATTATTCTGCCTGGCAAACTTCAACTACGAACGAGCCGGCACCCGAACACTCATCGCCCTACAAGGCCTACAACTTACCTCACCCCTCCTTACAGCCTGATGATTTCTAGCCTGCTCAACAAACATAGCCCTACCACCAACAATTAACCTAAACGGAGAACTAACCCTTATTACATCGCTATTTAGCTGACTAGACATCACAGTCTTCATAGCCGGATTAAAGACCTTCGCTACAACAACTTACACCCTCTACATATTTTCATCCACCCAACAAGCAACCCTACCACCTAATATTAAACCCTCGTCCCCCTCCCAAACTCGAGAACACTTCCTAATACTCCTCCACCTATTACCATCAGCCGGACTTGCAACCAACCCAAAATTAACCGCCCCCCAATA---AATCACACCCGTAACAACACTTTTTTTCCTAGCTTTCATAATTTGCACCACAGGCTTAATTATCCACCACAACCACCTTCTCTCAACGCTTCTCTGCCTCGAAGGCATAACCTTATCAGTCCTCCTAGCCCTCACAGTACTATCACTTCACTCACACCCATCATTATTCATCTTACCCTTAACAGTCTTAACCATTTCCGCTCGCGAAGCAGGAGTCGGCCTCGCCCTACTTATCGCCTCCACCCGAACACACAACACCACAAACCTTAAAAACCTAAACCTCCTCCAATGTTAAAT----GCCTAATCCCCCCCTCATCCTAACCACCTTTATCCTTCCCTTTCTACTCCTACTAACAGCCACCCTA------------AAAACTAGCAATACATCAATCAAATTCAAAACAACAATAACCAAATTAGCCTTCTTCACAAGCCTCCCCCCTCTTCTCTTAACCTTACCAAACAACAACACAACCTTGTCAAACCACTTTTACTTACTACACTTAGAATCTTTCAACCTACCCATCGCCCTAAAAATTGACACATACTCAGCCTTCTTCATTCCAACAGCACTATTCATTACATGATCAATTATAGAATTCTCCGAATCCTACATATCCTCAGACCCAAAAATTACCTCTTTCTTTAACCACCTACTAGTATTTATCCTAATAATAATAATCCTAGTAAGCGCTAACTACCTATTCAATCTCTTCATCGGCTGAGAAGGAGTTGGCATCCTGTCATACCTATTAATTAACTGATGGTCCTTCCGAGCAAGCTCCAACAAAGCAGCCCTCCAAGCCATCATTTACAACCGATTTTCAGACATCGGAATCCTAACCGCCCTATCATGAATAGCCACACATAACCTCACCCTAGACATTACAAACCTTTCCCCTTCTCCAAACTACCCCCTTATCCTGCCAGCCGCTATCATTTTTGCAGCAGCAGGAAAATCAGCCCAAATCGGACTCCACCCATGACTACCACCAGCTATAGAAGGCCCAACACCAGTCTCAGCCCTACTCCACTCAAGCACTATAGTAGTAGCCGGCATTTTCCTACTAATCCGAACTTCCCCGATCATCTACAGCTACCAAGAAACCACCACCGCCTGCCTAATCTTGGGGGCAATTACTTCCACATTTGCAGCCACCTGTGCCATCACCCAAAACGACATAAAAAAAATTATTGCCTTCTCAACCTCAAGCCAACTAGGACTGATAATAACCACAATCGGACTTAAACAACCCGAACTAACATTCCTACACATCCTAACGCACGCTTTCTTTAAAGCAATACTATTCCTATGCGCAGGATCAATTATCCACAACCTAAACAATGAACAAGACATCCGAAAAATAGGAAACCTAAAAAAAACAATACCCATTACCACCTCCTGTCTCACCACTGGAACGCTAGCCCTCACTGGTATACCCTTCTTATCTGGATTCTACTCCAAAGACGCCATTATTGAAACCCTAAATACCTCCCACATCAACATCTGGCCCCTCGCCCTCACCCTATTCGCCACTTCCGTCACCGCAGCCTACAGCCTTCGTATAGTCTACTTCACCCTACTAGGAACCAACCGATCATTAACAATAAACCCAATTGAAGAAAACCAAAAAACCATAAACCCAATCCTCCGTCTGGCCGTCGGAAGTATAATTGCCGGACTATTAATCTCGAACCTCATACTACCATCCCATACCCCTCAACTAACCATACCAACCACAATCAAATTCACAGCCTTAGCCATCACAACAGCCGGCCTTCTAACCGCAATAGCCTTAATATTCACCACAGATAAATTCCCATCAACCACAAAAGACCTCCAACCACCATCACTAACAAAACTAATATTCTTCAACCTAATTATTCACCACCTATTTTCTTCCACTGCACTACACTCCAGCCAAAAACTATCTACCCCCCTAACCGACCAAACATGATATGAAGCTATCGGACCAAAAACAACAGCTCGCCTACAAATCCTAATAACTACAACATTAACCCCCTACCACAAAGCAAAAATAAAAAAATACCTCATAGCCTTTCTATGAACTATCACCATCACTCTTCTCCTC---------TGAA---------GG---------------------------------ATGGAATTTACATT---GTTTTTGGGTTGTTTGGTGTTGATGTGTGCGGTAATGGTTGGGGCAAATTCTGGGGTTCATTTTGCTGTGCTGAGTTTAGTTTTTATGGCTATGTTGGGTAGTGGTTTGGTGGTGATTGAGGGTGGGAGTTTTATGCCTTTAGTGGTGTTGTTGGTTTGTTTGGGGGGGTTGTTGGTTGTGTTTGCTTTTTGTGTGGGGTTTACTGATGATCCGTTTG---------GTGGGGTTGGGGTCTCTAAGGAGTTTGTGTTTTTTTGTTTGGTTGGGTTGGTCGTGGTGGTAGGTTGTGTGTGTGGGTACTTGCGAGATTGTTGGGTTGTTGGGGTGAGTGGAATGGTGGGTGTAGAAGTTGTTGGAAGTGGTGCAGCTAATGAGTTGTTGGGAGTTGGGTTGATGTATTCAAGTGGGTGGGGGTTTGTTTTGGTGTGTAGTTGGGCTTTGTTAGTTGCGTTGTTCGTGATTACGGGGATTGTTCGTGGTCATTGTTTAGGGTCGCTTCGTTCCTTCAGA
